# Sources of SARS-CoV-2 and Other Microorganisms in Dental Aerosols

**DOI:** 10.1177/00220345211015948

**Published:** 2021-05-12

**Authors:** A.P. Meethil, S. Saraswat, P.P. Chaudhary, S.M. Dabdoub, P.S. Kumar

**Affiliations:** 1 Division of Periodontology, College of Dentistry, The Ohio State University, Columbus, OH, USA; 2 Laboratory of Clinical Immunology and Microbiology, National Institute of Allergy and Infectious Diseases, National Institutes of Health, Rockville, MD, USA; 3 James Cancer Center, The Ohio State University, Columbus, OH, USA

**Keywords:** ultrasonics, dental implants, irrigant, saliva, microbiota, DNA sequence analysis

## Abstract

On March 16, 2020, 198,000 dentists in the United States closed their doors to patients, fueled by concerns that aerosols generated during dental procedures are potential vehicles for transmission of respiratory pathogens through saliva. Our knowledge of these aerosol constituents is sparse and gleaned from case reports and poorly controlled studies. Therefore, we tracked the origins of microbiota in aerosols generated during ultrasonic scaling, implant osteotomy, and restorative procedures by combining reverse transcriptase quantitative polymerase chain reaction (to identify and quantify SARS-CoV-2) and 16S sequencing (to characterize the entire microbiome) with fine-scale enumeration and source tracking. Linear discriminant analysis of Bray-Curtis dissimilarity distances revealed significant class separation between the salivary microbiome and aerosol microbiota deposited on the operator, patient, assistant, or the environment (*P* < 0.01, analysis of similarities). We also discovered that 78% of the microbiota in condensate could be traced to the dental irrigant, while saliva contributed to a median of 0% of aerosol microbiota. We also identified low copy numbers of SARS-CoV-2 virus in the saliva of several asymptomatic patients but none in aerosols generated from these patients. Together, the bacterial and viral data encourage us to conclude that when infection control measures are used, such as preoperative mouth rinses and intraoral high-volume evacuation, dental treatment is not a factor in increasing the risk for transmission of SARS-CoV-2 in asymptomatic patients and that standard infection control practices are sufficiently capable of protecting personnel and patients from exposure to potential pathogens. This information is of immediate urgency, not only for safe resumption of dental treatment during the ongoing COVID-19 pandemic, but also to inform evidence-based selection of personal protection equipment and infection control practices at a time when resources are stretched and personal protection equipment needs to be prioritized.

## Introduction

On March 11, 2020, the World Health Organization declared coronavirus disease 2019 (COVID-19) a pandemic. On March 16, the American Dental Association responded with a recommendation that dental offices refrain from providing nonemergency services, resulting in 198,000 dentists closing their doors to patients in the United States alone. Nine months later, restrictions on the use of key instruments and procedures are still in place, fueling concern in providers and patients alike (Zhao et al. 2020). This is despite the fact that 3.5 billion individuals experience caries, periodontitis, or oral cancer (Kassebaum et al. 2014)—diseases that not only impede essential functions, such as speech and mastication (National Institute of Dental and Craniofacial Research 2014), but can also influence the course and outcomes of diabetes, atherosclerosis, and rheumatoid arthritis (D’Aiuto et al. 2005; de Pablo et al. 2009; Tonetti et al. 2013). Indeed, emerging evidence suggests that untreated periodontitis might increase the risk for developing severe forms of COVID-19 (Gupta and Sahni 2020; Pitones-Rubio et al. 2020).

Regulators and health authorities based this guidance largely on literature that aerosols generated during medical procedures, such as intubation/extubation, bronchoscopy, ventilation, and airway suctioning, can transmit infections (Davies et al. 2009; Judson and Munster 2019; Centers for Disease Control and Prevention 2020). Although microorganisms have been identified in dental aerosols (Grenier 1995; Harrel et al. 1998), our recent review found no information regarding their origins (Kumar and Subramanian 2020). Therefore, there is an urgent need to inform infection control science by definitively identifying the source of bacteria and viruses in aerosol-generating dental procedures (AGDPs).

We combined a rigorous clinical study with microbiomics and robust ecologic statistics to test the hypothesis that microorganisms from AGDPs are of nonsalivary origin. This enabled us to fill a critical gap in our knowledge and provide urgently needed information for appropriately apportioning our already stretched resources and guiding best practices for infection control during the COVID-19 pandemic and beyond.

## Methods

### Ethics Statement

This study was approved by the institutional review board of The Ohio State University (protocol 2020H0155) and carried out according to the approved guidelines and in accordance with the STROBE guidelines (Strengthening the Reporting of Observational Studies in Epidemiology) for human observational investigations.

### Participant Selection and Recruitment

Informed consent was obtained from 28 individuals seeking dental treatment in the College of Dentistry of The Ohio State University between May 4 and July 10, 2020. Exclusion criteria were age <18 y, current pregnancy, requirement for antibiotics prior to dental therapy, self-reported HIV or COVID-19 history, COVID-19–like symptoms since January 2020, and antibiotic therapy within 3 mo of sample collection. The sample size was based on previous evidence from in vitro and clinical studies on aerosol spread (King et al. 1997; Muzzin et al. 1999; Nulty et al. 2020; Allison et al. 2021) during scaling and restorative procedures.

### Operating Conditions

The AGDPs were dental implants, restorative procedures with high-speed handpieces, and ultrasonic scaling and were delivered by 5 operators and assistants in 2 enclosed operatories measuring 10.5 × 10 × 12 ft, with 6-exchange/min ventilation. Since aerosols are generated during normal physiologic activity, the operator, assistant, and sample collector wore N95 masks during the entire period to reduce microorganisms from extraneous sources. High-volume intraoral evacuators (mean suction capacity, 7.1 L/min; range, 6.6 to 7.4 L/min) were used throughout the AGDPs. Magnetostrictive ultrasonic scalers were used at a water flow of 19.3 mL/min. Implant osteotomies were performed at a flow rate of 30 mL/min. A high-speed handpiece with a single coolant port and flow rate of 23 mL/min was used for restorative procedures. Twenty-three individuals used a preprocedural rinse of 1% hydrogen peroxide (30 mL for 1 min), and 5 who underwent scaling did not use the rinse.

### Sample Collection

Prior to the procedure, 1 mL of unstimulated saliva and irrigant from the dental (or implant) unit was collected in tubes containing RNA stabilizer (RNA*Later*; Oragene). At 30 min following the procedure, condensate was collected from the face shields of the operator and assistant, the patient’s chest, and an area 6 ft distant from the site of operation (“environment”).

### Virus Identification

Investigators conducting the analyses were blinded to the source of samples. An RNA extraction–free dual-plexed reverse transcriptase quantitative polymerase chain reaction method for SARS-CoV-2 detection (SalivaDirect version 5) was used according to the developers’ instructions (Vogels et al. 2020). Briefly, 50 µL of homogenized saliva was mixed with 2 µL of 50-mg/mL proteinase K, and 5 µL was used in a 20-µL reaction containing FAM-labeled primers and probes targeting SARS-CoV-2 N1 and N2 and amplified for 44 cycles in triplicate reactions. RNA from TRIzol-inactivated virus (obtained from Dr. Wang of The Ohio State University) was used as positive control and to generate standard curves.

### Sequencing and Analysis Pipeline

DNA was isolated from 100 µL of the sample with a Qiagen MiniAmp Kit, after a 90-min incubation with lysozyme (2 mg/mL; Thermo Fisher Scientific). V1 to V3 and V4 to V5 (Kumar et al. 2011) regions of the 16S rDNA were sequenced on the Miseq system (2 × 250 bp). Negative and positive controls (defined culture mixture) were used in all runs. Amplicon sequence variants (ASVs) were inferred with the DADA2 version 1.16 pipeline (Callahan et al. 2016). Sequences were truncated on the basis of quality plots and filtered, dereplicated, and denoised with standard parameters, following which chimeras were identified and removed. Paired ends of denoised sequences were then merged. To be retained in the data set, the sequence had to be detected at least once in at least 5% of the samples. ASVs were assigned taxonomic identity with naive Bayes classifiers (QIIME2 q2-feature-classifier; Caporaso et al. 2010) trained for each primer pair by extracting the corresponding hypervariable region from the SILVA database (as of November 12, 2020; Quast et al. 2013).

### Controls

We used multiple internal controls in experimental design as well as analysis to validate these results. As a first level of control, we chose the same irrigant (dental unit water line) that passed through 2 different devices operating on 2 different principles (high-speed handpiece and ultrasonic scaler). We then selected a second therapeutic option, where a different irrigant source (saline) flows through a similar type of device (implant handpiece). By doing so, we controlled for the type of device as well as the type of coolant. In an effort to separate environmental bacteria found normally in water and aerosols from reagent contaminants, we subtracted the sequences found in our negative controls from samples. The negative controls were sterile Petri dishes opened by the sample collector in the clinic environment before the patient, operator, or assistant entered it and subjected to DNA isolation and polymerase chain reaction amplification in the same manner as the samples. It is well established that the hypervariable region that is sequenced can be a source of bias in determining the composition of the microbiome (Kumar et al. 2011). To overcome this, we used 2 primer pairs targeting 2 hypervariable regions and inducted them into separate sequence analysis pipelines.

### Statistical Analysis

ASVs were used to compute alpha diversity (within group) and beta diversity (between group). As an initial step, the negative controls were used to rarefy each data set. However, the sequencing depth still varied among the samples by a magnitude of 100 in some cases, especially between saliva and environmental condensate. Since current evidence does not support rarefying the microbiome to compensate for sequencing effort (McMurdie and Holmes 2014), we used cumulative sum scaling normalization from the Bioconductor package metagenomeSeq. Linear discriminant analysis (LDA) was performed with the MASS package for R. The input for LDA was a matrix of variance-stabilized (arc-sin square root transformed) relative abundances of ASVs (Shchipkova et al. 2010). MASS:lda provided singular value decomposition values, which were used to calculate the percentage variance explained in each dimension. The Dunn test with joint ranking was used to test significance of LDA clustering. SourceTracker version 0.9.5 (Knights et al. 2011) was applied to identify the source of microorganisms. Saliva and irrigant were designated as possible sources, and the operator, assistant, patient, and ambient environment were set as targets. Data sets were filtered to remove ASVs that were not present in at least 1% of samples. The following default parameters were used for analysis: rarefaction depth, 1,000; burn-in, 100; restart, 10; and alpha (0.001) and beta (0.01) Dirichlet hyperparameters.

## Results

**Figure 1.**
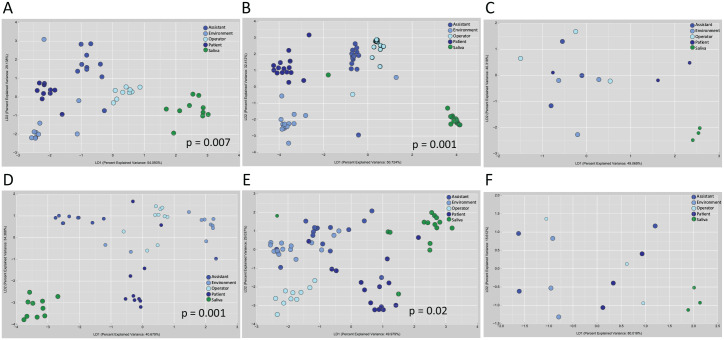
Differences in beta diversity between saliva and aerosol deposited on the operator, assistant, patient and the clinic environment. Linear discriminant analysis of Bray-Curtis dissimilarity distances are shown. Two hypervariable regions of the 16S rRNA gene (V1 to V3 and V4 to V5) were sequenced and analyzed separately. Amplicon sequence variants (ASVs) generated from V1-V3 (**A**–**C**), and V4-V5 regions (**D**–**F**) are shown. Samples were acquired during (A, D) implant surgery, (B, E) ultrasonic scaling, and (C, F) restorative procedures. Saliva demonstrated significant clustering from all aerosol samples irrespective of procedure (*P* < 0.05, Dunn test for joint ranking).

A total of 4,500,063 classifiable sequences representing 22,013 ASVs were used for microbial analysis. We began by characterizing the microbiota in saliva and the aerosol deposited on the operator, assistant, patient, and environment with an increasingly granular top-down approach. We found significant group separation between the salivary and aerosol microbiomes irrespective of the type of aerosol-generating procedure (*P* < 0.001, Dunn method for joint ranking of Bray-Curtis dissimilarity distances; Fig. 1, Appendix Table 1). By contrast, no such clustering was demonstrable among the aerosol deposited on the operator, assistant, patient, and environment. We then explored the abundances of individual taxa in the condensate generated from each procedure. Irrespective of the procedure or the area of deposition, 70% of the microbial abundance was attributable to 2 ASVs that mapped to uncultured *Vulcaniibacteria*. These ASVs were also the predominant taxa in the irrigants. Salivary bacteria, when present, accounted for 0.1% to 1.2% of the microbial abundance of aerosol.

**Figure 2.**
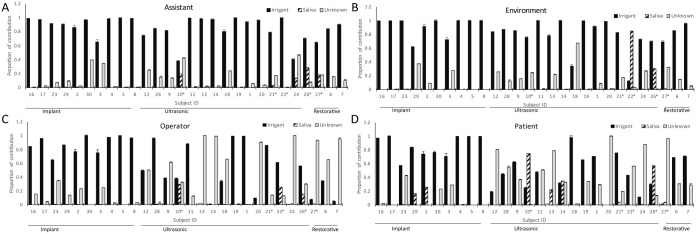
Source of microorganisms in aerosols generated during dental procedures. The relative contributions of saliva, irrigant fluid and unknown sources to the microbial composition of the condensate deposited on the assistant (**A**), environment (**B**), operator (**C**), and patient (**D**) are shown. The proportions were estimated using SourceTracker. For each subject, irrigant and saliva were designated as potential sources, and the operator, assistant, patient and the ambient environment were set as targets. Datasets were filtered to remove taxa that were not present in at least 1% of samples. Default parameters (rarefaction depth 1000, burn-in 100, restart 10, alpha [0.001] and beta [0.01] dirichlet hyperparameter) were used for analysis.

Source-tracking analysis revealed that, irrespective of the AGDP, microbiota from irrigants contributed to a median 78% of the microbiota in condensate (range, 2.5% to 100%; Fig. 2), while saliva contributed to a median 0% (range, 0% to 82%). On average, 20% of the microbiota could not be attributed to either source (range, 0% to 90%). Salivary bacteria were detectable in the condensate in only 8 participants out of the 28. Of these, 5 had not used a preprocedural rinse. In these 8 cases, the patient’s chest was the most frequent site of deposition (*P* < 0.05, chi-square test). LDA and SourceTracker revealed similar trends for ASVs generated from V1 to V3 and V4 to V5, indicating that our findings were robust against sequencing targets (Fig. 1).

We then investigated if aerosol dispersion of SARS-CoV-2 virus followed the same pattern, and it was identified in the saliva of 19 participants, with a viral load between 27 and 912 copies/mL. The virus was undetectable in the condensate on the operator, assistant, patient, or environment in any of these cases (Table).

**Table.** Levels of SARS-CoV-2 Virus in 28 Asymptomatic Patients with Noncontributory COVID-19 History and in Aerosols Generated during Dental Procedures on Them.

			Saliva		Operator		Assistant		Environment		Patient	
ID	Procedure	Periodontal Status	Average Ct	Viral Load	Average Ct	Viral Load	Average Ct	Viral Load	Average Ct	Viral Load	Average Ct	Viral Load
S1	Ultrasonic	Generalized moderate periodontitis	40.00	ND	NA	NA	NA	NA	NA	NA	NA	NA
S10^a^	Ultrasonic	Periodontal health	36.61	109.4	40	ND	40	ND	40	ND	40	ND
S11	Ultrasonic	Periodontal health	37.41	62.2	40	ND	40	ND	40	ND	40	ND
S12	Drilling	Periodontal health	38.45	29.9	40	ND	40	ND	40	ND	40	ND
S13	Ultrasonic	Periodontal health	37.01	82.2	40	ND	40	ND	40	ND	40	ND
S14	Ultrasonic	Generalized moderate periodontitis	35.01	338.2	40	ND	40	ND	40	ND	40	ND
S16	Implant	Periodontal health	40.00	ND	NA	NA	NA	NA	NA	NA	NA	NA
S17	Implant	Periodontal health	40.00	ND	NA	NA	NA	NA	NA	NA	NA	NA
S18	Ultrasonic	Periodontal health	37.12	76.3	40	ND	40	ND	40	ND	40	ND
S19	Ultrasonic	Generalized moderate periodontitis	37.14	75.4	40	ND	40	ND	40	ND	40	ND
S2	Implant	Periodontal health	40.00	ND	NA	NA	NA	NA	NA	NA	NA	NA
S20	Ultrasonic	Generalized moderate periodontitis	35.81	192.6	40	ND	40	ND	40	ND	40	ND
S21^a^	Ultrasonic	Generalized moderate periodontitis	36.30	136.1	40	ND	40	ND	40	ND	40	ND
S22^a^	Ultrasonic	Generalized moderate periodontitis	33.60	912.5	40	ND	40	ND	40	ND	40	ND
S23	Implant	Periodontal health	36.07	160.4	40	ND	40	ND	40	ND	40	ND
S24	Ultrasonic	Periodontal health	37.27	68.7	40	ND	40	ND	40	ND	40	ND
S26^a^	Ultrasonic	Generalized moderate periodontitis	35.09	318.6	40	ND	40	ND	40	ND	40	ND
S27^a^	Ultrasonic	Periodontal health	36.92	88.0	40	ND	40	ND	40	ND	40	ND
S28	Drilling	Periodontal health	37.83	46.2	40	ND	40	ND	40	ND	40	ND
S29	Implant	Periodontal health	40.00	ND	NA	NA	NA	NA	NA	NA	NA	NA
S3	Implant	Periodontal health	37.02	81.9	40	ND	40	ND	40	ND	40	ND
S30	Implant	Periodontal health	38.56	27.7	40	ND	40	ND	40	ND	40	ND
S4	Implant	Periodontal health	40.00	ND	NA	NA	NA	NA	NA	NA	NA	NA
S5	Implant	Periodontal health	40.00	ND	NA	NA	NA	NA	NA	NA	NA	NA
S6	Implant	Periodontal health	40.00	ND	NA	NA	NA	NA	NA	NA	NA	NA
S7	Ultrasonic	Periodontal health	40.00	ND	NA	NA	NA	NA	NA	NA	NA	NA
S8	Implant	Periodontal health	36.57	112.3	40	ND	40	ND	40	ND	40	ND
S9	Drilling	Periodontal health	37.53	57.2	40	ND	40	ND	40	ND	40	ND

NA, aerosol sample not analyzed since the virus was not identified in the saliva samples; ND, not detected by reverse transcriptase quantitative polymerase chain reaction at a detection threshold of 40 reaction cycles.

a Patient did not use a mouth rinse.

## Discussion

The impetus for this study came from our recent review of the literature on aerosols generated during dental procedures (Kumar and Subramanian 2020). We found that while several investigations measured the radius of contamination following AGDPs and demonstrated the presence of microbiota in these contaminated areas (Grenier 1995; Harrel et al. 1998; Rivera-Hidalgo et al. 1999; Petti and Vitali 2017; Kobza et al. 2018; Jain et al. 2020), few characterized the types of microorganisms, and none identified their source. Understanding the sources of microbial bioload in aerosols is of immediate urgency, not only for infection control in dental operatories during the COVID-19 pandemic, but also to inform best practices in aerosol reduction, mitigation, and abatement in the long term. To the best of our knowledge, this study sets the stage for future work on risk of disease transmission among dental health care workers and patients.

We deliberately elected to employ a passive air-sampling methodology over other aerosol-harvesting methods (Greco and Lai 2008; Verreault et al. 2008; O’Neil et al. 2017), since dental procedures generate a variety of airborne particles, ranging from spatter and particulate matter to aerosolized microorganisms, any of which could pose a health hazard to patient and personnel. Our method allowed us to collect everything that was generated during the AGDP as well as everything that settled on the surfaces during the following 30 min. This method has been widely used in similar studies, since it is reflective of events that occur in a treatment session (Bentley et al. 1994; Bennett et al. 2000; Hallier et al. 2010).

This study was conducted at the height of the COVID-19 pandemic, and patients were inducted into the study based on presentation for treatment to the dental clinics. We did not do a COVID-19 test before starting the study because the primers and probes had not been optimized when we began our recruitment. An extensive review of the literature revealed wide heterogeneity in sample size, with a minimum of 3 (Bentley et al. 1994) and a maximum of 20 (Serban et al. 2013; Singh et al. 2016). As a result, we included 15 participants for the ultrasonic group. During the same time frame, 10 patients who presented for implant therapy consented to participate. Thus, we did an interim analysis to see if we could detect differences among the aerosols deposited on the various surfaces with 10 participants, and since there were significant differences, we used this as our endpoint. Three studies used 3 subjects/replicates per group for restorative procedures and to estimate SARS-CoV-2 spread (van Doremalen et al. 2020; Allison et al. 2021) and therefore we included the restorative data from 3 subjects for completeness.

With all the procedural controls described in the methods, we used an emergent bioinformatics approach to characterize microbial assemblages: ASVs. Bioinformatics has traditionally sought to reduce large 16S sequence data sets into manageable operational units, either by assigning them a taxonomic identity by comparison with a database or by identifying taxon-independent operational taxonomic units with a sequence similarity threshold (Huse et al. 2010). Both these approaches fail to capture the immense diversity of environmental bacteria, many of which have not been assigned definitive taxonomic identities or entered into curated databases (Callahan et al. 2017). The decision to use ASVs over operational taxonomic units was driven by the need for a finer scale resolution of bacterial identities than could be provided by traditional methods. This allowed us to exploit the immense sensitivity built into the 16S rRNA gene and overcome the limitations imposed by incomplete reference databases to better discriminate ecologic patterns (Prodan et al. 2020).

Our key discovery was that the irrigant fluid contributes to the majority of the bioload in dental aerosols. Although this is the first time that it is being reported, this is not entirely surprising, since we estimated that the irrigant flow dilutes the saliva by 20- to 200-fold (assuming a salivary flow rate of 0.1 to 1.0 mL/min; Iorgulescu 2009). This discovery lends credence to previous studies on the transmission of legionellosis, a waterborne infection, and pneumonia, from dental unit water lines (Ricci et al. 2012; Petti and Vitali 2017). It also validates recent reports of extremely low COVID-19 transmission among dental personnel (Estrich et al. 2020).

One surprising finding was the discovery of microorganisms in the implant irrigant, which was sterile saline. To confirm that this was not an artifact, we sequenced the saline before and after it passed through the implant handpiece and discovered a diverse microbiome in reusable handpieces. However, since these handpieces were sterilized by autoclaving, we anticipate that these organisms are not viable.

The salivary SARS-CoV-2 load in our study is consistent with previous reports of 10^2^ to 10^6^ copies/mL (Chau et al. 2020); however, none was detected in any aerosol. Interestingly, participant 22 demonstrated the highest salivary viral load, and while bacteria of salivary origin could be identified on the operator and environment, the virus was not detectable in the same samples. This was true of those individuals with periodontitis and those who were periodontally healthy. The 20- to 200-fold dilution factor and our discovery that the irrigant, not the saliva, is the primary and predominant source of airborne microbiota might explain the absence of SARS-CoV-2 in aerosol. Preprocedural mouth rinsing serves to further reduce potential salivary contaminants in aerosol.

In summary, we find sufficient evidence to reject our null hypothesis that saliva is a potential source of disease transmission during AGDPs. Instead, we identify, for the first time, that the irrigant used in dental equipment is the primary and major source of microorganisms during AGDPs. We also demonstrate that high-volume intraoral evacuators are highly effective in reducing salivary contamination of the surrounding environment and that preprocedural mouth rinses consistently reduce salivary microbial bioloads. Within the limitations of a small sample size, we conclude that the risk for transmission of SARS-CoV-2 and other respiratory pathogens from aerosolized saliva in dental operatories is moderately low and that current infection control practices are adequately robust to protect personnel and patients alike.

## Author Contributions

A.P. Meethil, S. Saraswat, P.P. Chaudhary, S.M. Dabdoub, P.S. Kumar, contributed to conception, design, and data acquisition, drafted and critically revised manuscript. All authors gave final approval and agree to be accountable for all aspects of the work.

## Supplemental Material

sj-pdf-1-jdr-10.1177_00220345211015948 – Supplemental material for Sources of SARS-CoV-2 and Other Microorganisms in Dental Aerosols Click here for additional data file. Supplemental material, sj-pdf-1-jdr-10.1177_00220345211015948 for Sources of SARS-CoV-2 and Other Microorganisms in Dental Aerosols by A.P. Meethil, S. Saraswat, P.P. Chaudhary, S.M. Dabdoub and P.S. Kumar in Journal of Dental Research
