# Corrigendum to “Sources of SARS-CoV-2 and Other Microorganisms in Dental Aerosols”

**DOI:** 10.1177/00220345211064910

**Published:** 2021-12-20

Meethil AP, Saraswat S, Chaudhary PP, Dabdoub SM, Kumar PS. 2021. Sources of SARS-CoV-2 and other microorganisms in dental aerosols. J Dent Res. 100(8):817–823.

Since the original publication of this article, it has been brought to our attention that there were minor inaccuracies in the original article. Specifically, the ventilation rate and the primer set used for SARS-CoV-2 detection were incorrect. Corrected and more complete methods for these protocols are given below.

**Operating conditions:** The aerosol generating procedures (AGPs) included in the study were osteotomies for dental implants, tooth preparation for restorations, and scaling and root planing using ultrasonic scalers. All treatment was delivered in two enclosed dental operatories measuring 10.5 × 10 × 12 feet each, with a ventilation system that allowed 6 air exchanges per hour. Five operators and assistants carried out the clinical procedures, and at any given time, no more than 3 individuals occupied the room. Operators and assistants wore N95 masks with single-use face shields, and high-volume intra-oral evacuators were used during AGP.

**Virus identification:** We used an RNA-extraction free, dual-plexed RT-qPCR method for SARS-CoV-2 detection (SalivaDirect^TM^ V.5) as per the developers’ instructions. 50 µl of saliva were mixed with 2.5 µl of proteinase K (ThermoFisher Scientific) and vortexed for 1 min at 5000 rpm, following which proteinase K was deactivated by heating for 5 min at 95°C. 5 µl of the proteinase-treated sample were used in a 20 µl reaction containing primers and probes for the N1 target and amplified for 44 cycles in a Bio-Rad CFX96 thermocycler using the cycling conditions specified in the protocol. RNA from trizol-inactivated virus (obtained from Dr. Wang, Food Animal Health Research Program of The Ohio State University) was used as positive control and to generate standard curves, and the master mix without template was used as no-template control. All samples were run in triplicate, and the Ct values averaged. Samples with inconsistent Ct values (±3 Ct) between replicates were repeated. Based on the developer’s protocol, Ct values of ≥40 were considered negative for presence of the virus. Virus RNA copies were quantified using the formula X = E_amp_^(b-Ct)^, where b = 35.75, and E_amp_ = 2.022 (R² = 0.9969) based on a 5-fold dilution standard curve of RNA.

