# Supplementary material for: Sources of SARS-CoV-2 and Other Microorganisms in Dental Aerosols
Source: J Dent Res. 2021 May 12;100(8):817–23. doi: 10.1177/00220345211015948 (PMC8258727; doi:10.1177/00220345211015948)
Supplement: sj-pdf-1-jdr-10.1177_00220345211015948 – Supplemental material for Sources of SARS-CoV-2 and Other Microorganisms in Dental Aerosols [file sj-pdf-1-jdr-10.1177_00220345211015948.pdf]

Supplemental Table 1: **Relative abundances of taxa in condensate deposited on the assistant, environment, operator and patient.** ASVs were assigned taxonomic identity using Naive Bayes classifiers (QIIME2 q2-feature-classifier) trained for each primer pair by extracting the corresponding hypervariable region from the SILVA database (as of November 12,2020).

Ultrasonic																	
Assistant			Environment			Operator			Patient			Saliva			Waterline		
Taxon ID	Mean relative abundance	Std dev	Taxon ID	Mean relative abundance	Std dev	Taxon ID	Mean relative abundance	Std dev	Taxon ID	Mean relative abundance	Std dev	Taxon ID	Mean relative abundance	Std dev	Taxon ID	Mean relative abundance	Std dev
Uncultured_Vulcanibacterium	0.886134445	0.036795863	Uncultured_Vulcanibacterium	0.9156728	0.035253608	Uncultured_Vulcanibacterium	0.415571933	0.026406008	Uncultured_Vulcanibacterium	0.518221148	0.031632344	ASV_346	0.018358832	0.003283972	Uncultured_Vulcanibacterium	0.082205311	0.007802935
Uncultured_Vulcanibacterium	0.750443747	0.028657492	Uncultured_Vulcanibacterium	0.817254182	0.036760755	Uncultured_Vulcanibacterium	0.382017117	0.02596621	Uncultured_Vulcanibacterium	0.455260158	0.025621996	ASV_305	0.015936627	0.002928262	Uncultured_Vulcanibacterium	0.079733722	0.006895084
Schlegelella_uncultured_bacterium	0.080155871	0.00846083	Schlegelella_uncultured_bacterium	0.061203972	0.006385497	Treponema_denticola	0.04047585	0.008442465	Treponema_denticola	0.029695733	0.007318144	Solobacterium_moorei	0.010110376	0.000703207	C1-B045_uncultured_gamma	0.075177958	0.009765081
Rhodococcus_erythropolis	0.011025795	0.00078122	Rhodococcus_erythropolis	0.00979544	0.000717273	Schlegelella_uncultured_bacterium	0.029519012	0.003370783	ASV_256	0.028777057	0.006694022	Rothia_aeria	0.008824588	0.000759255	ASV_144	0.065698339	0.00853037
Brevundimonas_diminuta	0.007043422	0.000602043	Acetobacteraceae_uncultured_alpha_proteobacterium	0.008146528	0.000743928	Streptococcus_mutans	0.016280223	0.003658637	ASV_292	0.020701415	0.004336799	Treponema_denticola	0.006779841	0.000884068	Denitratisoma_sp.	0.059145663	0.003697241
Acetobacteraceae_uncultured_alpha_proteobacterium	0.005271268	0.000489914	ASV_292	0.007789915	0.001751498	Rhodococcus_erythropolis	0.012448189	0.001538966	Schlegelella_uncultured_bacterium	0.015355605	0.00149017	Filifactor_sp.	0.00622236	0.00099116	ASV_164	0.053197757	0.007454065
ASV_363	0.005173774	0.000692659	ASV_256	0.006989718	0.001656132	Actinomyces_israelii	0.009298507	0.001413016	Rhodococcus_erythropolis	0.014835867	0.00160692	Leptotrichia_wadei	0.006028458	0.000841248	Desulfobacterota_uncultured_Desulfuromonadales	0.034102015	0.00043196
ASV_1208	0.004793763	0.000499576	Brevundimonas_diminuta	0.006452333	0.000761022	Cryptobacterium_sp.	0.008344624	0.001491323	Streptococcus_mutans	0.014217671	0.002173958	Prevotella_micans	0.005433324	0.001178305	ASV_211	0.015368769	0.001758182
ASV_366	0.004538279	0.000604158	Rothia_aeria	0.006090623	0.000995602	Brevundimonas_diminuta	0.007956799	0.000622528	Rothia_aeria	0.012013436	0.001392753	Prevotella_nigrescens	0.005331793	0.000613381	Thioclava_uncultured_bacterium	0.013839385	0.003092483
Lactobacillus_coryniformis	0.004455369	0.001150371	ASV_366	0.005831407	0.001430518	ASV_476	0.006874881	0.001503497	Prevotella_nigrescens	0.008332498	0.001482595	Streptococcus_mutans	0.004902567	0.000962101	Obscuribacteraceae_sp.	0.010063183	0.000953937
ASV_720	0.004061493	0.000320978	ASV_363	0.005813664	0.001425943	Jonquetella_anthropi	0.00559539	0.001053243	Acetobacteraceae_uncultured_alpha_proteobacterium	0.007265158	0.000704404	Butyrivibrio_Eubacterium_sp.	0.00453547	0.000669285	ASV_800	0.00982428	0.001423236
Corynebacterium_propinquum	0.003460364	0.00082635	Chloroplast_Cinnamomum_camphora	0.005687618	0.001468537	ASV_256	0.004891857	0.00072013	Brevundimonas_diminuta	0.007228823	0.000727233	ASV_256	0.003887228	0.000446074	ASV_621	0.008571966	0.001607594
ASV_709	0.003458785	0.000391414	ASV_709	0.005625651	0.00082394	ASV_305	0.00484635	0.000931337	ASV_305	0.005633951	0.001182345	Prevotella_baroniae	0.003770441	0.000458296	ASV_897	0.007963823	0.000919408
ASV_292	0.00319165	0.000490525	ASV_3352	0.005074642	0.001088725	ASV_292	0.004644388	0.00071115	Treponema_maltophilum	0.004634951	0.001168461	ASV_292	0.003600664	0.000423034	Singulisphaera_Isosphaera_sp.	0.0064566	0.000789967
Dehalococcoidia_RBG-13-46-9_sp.	0.003116031	0.000804556	ASV_720	0.00498194	0.000559284	Rothia_aeria	0.004469627	0.00030223	ASV_709	0.004599968	0.000567874	Peptococcus_sp.	0.003381966	0.000528795	Simkaniaceae_uncultured_Candidatus	0.006228528	0.00084428
Methylopilaceae_sp.	0.003072669	0.00079336	Corynebacterium_mycetoides	0.003961084	0.001022747	ASV_678	0.00403631	0.000794641	ASV_346	0.004397119	0.000712263	Clostridia_vadinBB60_group_Clostridiales_bacterium	0.002830708	0.000456943	ASV_1021	0.006093786	0.000689847
Lawsonella_uncultured_bacterium	0.002934037	0.000489093	Lactobacillales_sp.	0.003466926	0.000895156	Acetobacteraceae_uncultured_alpha_proteobacterium	0.003888	0.000416494	ASV_513	0.004321703	0.001115859	Campylobacter_concisus	0.002793493	0.000705321	Edaphobaculum_uncultured_Chitinophagaceae	0.00515461	0.000494469
ASV_6154	0.002788204	0.000719911	Oscillospiraceae_NK4A214_group_metagenome	0.003189571	0.000823544	Lawsonella_uncultured_bacterium	0.003652846	0.000535819	Leptotrichia_wadei	0.004270808	0.000750833	Eubacterium_minutum	0.001883587	0.000451974	Legionellaceae_uncultured_Legionella_sp.	0.005132602	0.001148163
ASV_8581	0.002765402	0.000714024	ASV_6187	0.003157574	0.000615922	ASV_709	0.00340454	0.000293831	ASV_366	0.004026096	0.000710979	Actinomycetaceae_F0332unidentified	0.001820979	0.000187315	ASV_413	0.004792157	0.001022215
Isosphaera_sp.	0.002765402	0.000714024	Cellvibrio_uncultured_Cellvibrio	0.003061199	0.000445219	ASV_346	0.003385169	0.000567575	ASV_363	0.003967545	0.000715065	ASV_1443	0.001786477	0.000461266	Amb-16S-1323_uncultured_bacterium	0.003710124	0.00045913
Chloroplast_Melosira_varians	0.00275594	0.000489066	Dermatophilaceae_uncultured__bacterium	0.002999667	0.000774511	ASV_513	0.003350551	0.000865109	Actinomyces_israelii	0.003946671	0.000549472	Lachnospiraceae_uncultured_Eubacterium_sp.	0.001694054	0.000437403	ASV_366	0.002700288	0.000388215
alphaproteobacteria_uncultured__bacterium	0.002690092	0.000673213	Lactobacillales_P5D1-392_sp.	0.002881383	0.00074397	Solobacterium_moorei	0.002905535	0.000381757	Ruminococcaceae_uncultured__rumen	0.003719392	0.000960343	Streptococcus_anginosus	0.001618166	0.000180998	Schlegelella_uncultured_bacterium	0.002609428	0.000311951
ASV_3442	0.002643948	0.000682664	Alphaproteobacteria_uncultured_Lacibacterium_aquatile	0.002616157	0.000675489	Amb-16S-1323_uncultured_bacterium	0.002807111	0.000724793	ASV_4644	0.003616076	0.000933667	Pyramidobacter_piscolens	0.001496715	0.000365028	ASV_363	0.002312312	0.000347958
Steroidobacteraceae_uncultured__metagenome	0.002611768	0.000674356	Sandaracinaceae_uncultured_Myxococcales	0.002555272	0.000659768	ASV_720	0.002720444	0.000250626	Prevotella_baroniae	0.00361135	0.000932447	Leptotrichia_hofstadii	0.001481313	0.000280683	oc32_uncultured_bacterium	0.001561783	0.000292797

Citricoccus_sp.	0.002601006	0.000671577	Leptolyngbyaceae_uncultured_cyanobacterium	0.002496186	0.000644512	Prevotella_nigrescens	0.002637057	0.000334738	ASV_476	0.003520237	0.000693262	Bacteroides_pyogenes	0.001434998	0.000328178	Acetobacteraceae_uncultured_alpha_proteobacterium	0.001480797	0.000343843
Tepidimonas_sp.	0.002480314	0.000457271	Ramlibacter_sp.	0.002496186	0.000644512	Aliterella_uncultured_bacterium	0.002479416	0.000640182	ASV_720	0.003370846	0.000423533	Bulleidia_extructa	0.001419954	0.000271275	Rhodococcus_erythropolis	0.001351634	0.000164574
Chloroplast_Chlorella_heliozoae	0.002470407	0.000637856	Paracaedibacteraceae_sp.	0.002479218	0.000640131	Leptotrichia_hofstadii	0.002384264	0.000615614	Singulisphaerauncultured_eubacterium	0.002996177	0.00077361	ASV_1665	0.001398113	0.000360991	ASV_2649	0.001134037	0.000148396
Bacteroidales_RF16_groupuncultured_Porphyromonadaceae	0.002458135	0.000634688	Oligoflexales_uncultured__soil	0.002418553	0.000279379	ASV_3099	0.002377818	0.00061395	Oligoflexales_uncultured__soil	0.00294806	0.000718494	Metamycoplasma_salivarium	0.001356566	0.000252289	ASV_2622	0.001091854	0.000264889
Segetibacter_sp.	0.002344996	0.000605475	Hungateiclostridiaceae_UCG-012_uncultured_bacterium	0.002406865	0.00062145	ASV_363	0.002232152	0.000271	Rubinisphaeraceae_SH-PL14__uncultured_planctomycete	0.002867448	0.000520039	ASV_2779	0.001322375	0.000341436	Obscuribacteraceae_metagenome	0.001008228	0.000210553
Prevotella_veroralis	0.002251196	0.000581256	Perlucidibaca_uncultured_bacterium	0.002406865	0.00062145	Treponema_parvum	0.002138649	0.000552197	Actinomycetaceae_F0332unidentified	0.002754181	0.000331748	Stomatobaculum_uncultured_bacterium	0.00132044	0.000340936	ASV_3132	0.000985834	0.000181115
ASV_256	0.002122553	0.00033149	Anaerococcus_hydrogenalis	0.002399808	0.000619628	ASV_2080	0.00212594	0.000370931	Phaselicystis_sp.	0.00269919	0.000696928	Corynebacterium_durum	0.001296741	0.000166695	Brevundimonas_diminuta	0.000967385	0.000199384
Cellvibrio_uncultured_Cellvibrio	0.002088866	0.000276145	DEV114_uncultured_verrucomicrobium	0.002302219	0.00059443	Corynebacterium_propinquum	0.002071803	0.000390073	Georgenia_uncultured_bacterium	0.002628505	0.000678677	Candidatus_SaccharimonasTM7_phylum	0.001284211	0.000236835	ASV_709	0.000915606	0.000135565
ASV_7220	0.002056404	0.000530961	Actinoplanes_sp.	0.002282556	0.000589353	Tannerella_forsythia	0.002063287	0.000377252	ASV_4892	0.002551415	0.000658773	Eubacterium_nodatum	0.001259587	0.000179254	ASV_720	0.000893788	0.000144098
Methylomonadaceae_uncultured__metagenome	0.002056404	0.000530961	Selenomonadaceae_uncultured__rumen	0.002223767	0.000574174	Anaerovoracaceae_Family_XIII_UCG-006Peptostreptococcaceae_bacterium	0.002021996	0.000522077	ASV_6651	0.002479595	0.000640229	Alloscardovia_omnicolens	0.001249089	0.000276022	ASV_1862	0.000832841	9.38732E-05
KaistiaRhizobiales_(rhizobacteria)	0.001997235	0.000515684	Caenarcaniphilales_microbial_mat	0.002197572	0.000567411	Actinomycetaceae_F0332unidentified	0.0019313	0.000442289	Treponema_parvum	0.002475749	0.000639236	ASV_476	0.001186567	0.000237311	Gammaproteobacteria_PLTA13_sp.	0.000822791	0.000212444
Parabacteroides_merdae	0.001997235	0.000515684	Hydrogenedensaceae_uncultured_bacterium	0.002197572	0.000567411	Metamycoplasma_salivarium	0.001731925	0.000251827	Treponema_lecithinolyticum	0.002435891	0.000336919	Treponema_lecithinolyticum	0.001111763	0.000158216	ASV_3215	0.000815507	0.000141628
ASV_6633	0.00196802	0.000508141	Planctomycetes_uncultured_Planctomycetaceae	0.002197572	0.000567411	Mogibacterium_timidum	0.001678516	0.000433391	Symbiobacterium_sp.	0.002396784	0.000618847	Porphyromonas_catoniae	0.001111726	0.000142989	ASV_1642	0.000813114	0.000209945
Rikenella_uncultured_bacterium	0.00196802	0.000508141	Prevotellaceae_UCG-003_uncultured_Bacteroidales	0.002197572	0.000567411	Anaerolineaceae_bacterium	0.00166467	0.000355919	Bryobacter_uncultured_bacterium	0.002169646	0.00056002	Anaerovoracaceae_Family_XIII_UCG-004Eubacterium_sp.	0.001055212	0.000171982	ASV_2180	0.000749911	0.000134353
Gallicola_Peptostreptococcaceae_bacterium	0.001966725	0.000507806	ASV_1837	0.001935231	0.000431438	Cellvibrio_uncultured_Cellvibrio	0.001640209	0.00016761	Weeksellaceae_uncultured_Wautersiella_sp.	0.00209937	0.000542055	Megasphaera_micronuciformis	0.001038875	0.000268236	Saccharimonadalesuncultured_organism	0.000748065	0.00019315
Periglandula_ipomoeae	0.001875997	0.00048438	Acinetobacter_kyonggiensis	0.001922153	0.000496298	Leptotrichia_wadei	0.001632748	0.000302853	Anaerovoracaceae_Family_XIII_UCG-006Peptostreptococcaceae_bacterium	0.00209932	0.000542042	Anaerovoracaceae_Family_XIII_UCG-005Eubacterium_sp.	0.000957227	0.000148604	ASV_10537	0.000719942	0.000185888
ASV_3443	0.001844571	0.000429017	Burkholderiales_sp.	0.001922153	0.000496298	Candidatus_Aquirestis_uncultured_bacterium	0.00161352	0.000416609	ASV_678	0.002096948	0.000376884	Parvimonas_Peptostreptococcaceae_bacterium	0.00090065	0.000232547	Terrimicrobiaceae_FukuN18_freshwater_group_sp.	0.000719942	0.000185888
Sharpea_uncultured_bacterium	0.001823056	0.000470711	Phycisphaeraceae_uncultured__metagenome	0.001922153	0.000496298	Allobranchiibius_sp.	0.001571059	0.000405646	Fretibacteriumuncultured_Fretibacterium	0.002005212	0.000517744	ASV_678	0.000838831	0.000142312	Rothia_aeria	0.000710934	6.91453E-05
Acinetobacter_towneri	0.001754106	0.000452908	Saprospiraceae_OLB8_sp.	0.001922153	0.000496298	ASV_4513	0.001528598	0.000394682	Chloroflexia_sp.	0.0019994	0.000516243	ASV_513	0.000739905	0.000191043	Candidatus_Obscuribacter_uncultured_bacterium	0.000668518	0.000172611
ASV_7457	0.001722017	0.000444623	Gaiellales_sp.	0.001919846	0.000495702	Roseomonas_lacus	0.00145705	0.000376209	Pyramidobacter_piscolens	0.001985939	0.000512767	Simonsiella_muelleri	0.000705163	0.000182072	ASV_1760	0.000666753	0.000172155
Saprospiraceae_OLB8_metagenome	0.001715818	0.000443022	Bdellovibrio_uncultured_Bdellovibrionales	0.001888679	0.000487655	Gammaproteobacteria_EV818SWSAP88uncultured_gamma	0.001443675	0.000372755	Plantibacter_sp.	0.001807026	0.00032167	Neisseria_bacilliformis	0.000658212	0.00016995	ASV_4280	0.000614747	0.000158727
ASV_1837	0.001694231	0.000299405	ASV_5944	0.001876303	0.000484459	ASV_366	0.001436765	0.000185702	Lawsonella_uncultured_bacterium	0.001738559	0.000233559	Actinomycetaceae_F0332_uncultured_bacterium	0.000628416	0.000105076	Leptospirauncultured_Spirochaetales	0.000579595	8.35946E-05
Phaeodactylibacter_metagenome	0.001689968	0.000436348	ASV_2889	0.001835577	0.000473944	Rhizobiales_A0839_uncultured_sludge	0.00137558	0.000250707	Eggerthellaceae_DNF00809_uncultured_bacterium	0.001730129	0.000446717	Filifactor_alocis	0.000610327	0.000157586	Paracaedibacteraceae_uncultured_Acetobacteraceae	0.000565669	0.000146055
Lactobacillus_sanfranciscensis	0.001688397	0.000435942	ASV_5749	0.001817238	0.000469209	Butyrivibrio_Eubacterium_sp.	0.00136668	0.000247345	SelenomonasSchwartzia_sp.	0.001715651	0.000442979	Prevotellauncultured_Prevotella	0.000609263	0.000157311	ASV_4670	0.000552917	0.000142763
Chlamydiaceae_uncultured_organism	0.001608579	0.000415333	Hylemonella_sp.	0.001813326	0.000431238	Shuttleworthia_satelles	0.001360607	0.000258496	ASV_5494	0.001685752	0.000435259	Leptotrichia_shahii	0.000600258	0.000125734	Meiothermus_uncultured_Deinococci	0.000539944	6.96706E-05
ASV_6292	0.001598754	0.000358438	Occallatibacter_sp.	0.00180681	0.000466516	ASV_3100	0.001350084	0.00034859	Mogibacterium_timidum	0.001679456	0.000433634	ASV_366	0.000580381	7.07856E-05	ASV_4800	0.000536655	0.000138564
Oscillospirales_UCG-010_metagenome	0.001593774	0.000288744	ASV_9280	0.00177758	0.000458969	Dehalococcoidia_RBG-13-46-9uncultured_sludge	0.001324591	0.000342008	ASV_5701	0.001627623	0.00042025	Prevotella_multiformis	0.000553341	0.000142872	Chloroflexi_OLB14_uncultured_bacterium	0.000521326	8.0358E-05
Lactobacillus_delbrueckii	0.001565475	0.000404204	ASV_10043	0.001681884	0.000434261	Oligoflexales_uncultured__soil	0.001286439	0.000136824	Selenomonadaceae_uncultured__sp.	0.001623628	0.000419219	Parvimonas_sp.	0.000520537	5.98127E-05	ASV_13441	0.000514245	0.000132777
Bacillus_coagulans	0.001560603	0.000402946	ASV_9484	0.001674341	0.000432313	Defluviitaleaceae_UCG-011_uncultured_rumen	0.001227689	0.000316988	Fibrobacteraceae_uncultured_microbial_mat	0.001557632	0.000402179	ASV_363	0.000498584	7.39388E-05	ASV_5985	0.000511178	0.000103489
Rothia_aeria	0.001560603	0.000402946	Mangroviflexus_sp.	0.001674341	0.000432313	Fretibacteriumuncultured_Fretibacterium	0.001211901	0.000312911	ASV_3639	0.001528799	0.000263167	Eggerthia_catenaformis	0.00047084	7.63442E-05	Candidatus_Bealeia_uncultured_Alphaproteobacteria	0.000506074	9.09926E-05

Coraliomargarita_sp.	0.001558016	0.000402278	Steroidobacter_sp.	0.001600768	0.000413317	uncultured_Actinomyces	0.00120731	0.000311726	bacterium_CS910	0.00149955	0.000387182	Treponema_medium	0.000461118	0.00011906	Streptococcus_mutans	0.000498174	9.44211E-05
Leptolyngbya_PCC-6306_sp.	0.00154486	0.000398881	Brochothrix_sp.	0.001599147	0.000412898	ASV_1901	0.001189176	0.000198014	Pantoea_sp.	0.00149955	0.000387182	Cardiobacterium_sp.	0.000452033	5.03825E-05	Oligoflexus_uncultured_delta	0.000473253	7.56976E-05
ASV_5700	0.001488333	0.000384286	Caulobacter_metagenome	0.001569694	0.000405293	Spongiibacteraceae_sp.	0.001169536	0.000301973	Hephaestia_uncultured_bacterium	0.00144643	0.000373467	Alysiella_uncultured_bacterium	0.000432222	0.000111599	ASV_2510	0.000469312	9.4777E-05
ASV_4770	0.001476015	0.000381105	ASV_7514	0.001561749	0.000403242	Belnapia_sp.	0.001136212	0.000293369	Acetitomaculum__uncultured_bacterium	0.001395106	0.000360215	Anaerovoracaceae_uncultured__bacterium	0.000413479	0.00010676	ASV_4487	0.000468478	0.00012096
Myroides_uncultured_bacterium	0.00146886	0.000379258	Terrabacter_sp.	0.001561749	0.000403242	Hymenobacter_uncultured_Bacteroidetes	0.001136212	0.000293369	Oscillospiraceae_UCG-005_metagenome	0.001395106	0.000360215	Anaerovoracaceae_Family_XIII_UCG-001_sp.	0.000406076	6.34502E-05	ASV_14376	0.00046282	0.00011095
Cytophaga_aurantiaca	0.001441071	0.000372083	Acidobacteriae_Elev-16S-1166_uncultured_bacterium	0.001559875	0.000402758	Candidatus_Aquirestis_sp.	0.001131072	0.000158661	Arcticibacter_uncultured_bacterium	0.001375752	0.000355218	Actinomyces_israelii	0.000368543	6.74058E-05	Carnobacteriaceae_uncultured__bacterium	0.00046282	0.00011095
Mitochondriauncultured_Rickettsiales	0.001413453	0.000364952	Haliangium_uncultured_Kofleriaceae	0.001559875	0.000402758	Treponema_maltophilum	0.001095248	0.000282792	ASV_3568	0.001352817	0.000218943	Rhodospirillales_uncultured__sp.	0.000327906	8.4665E-05	Marinoscillum_metagenome	0.00046282	0.00011095
Prevotella_nigrescens	0.001399803	0.000221396	Lactobacillus_gasseri	0.001559875	0.000402758	Geobacteraceae_sp.	0.001087568	0.000280809	Tepidimonas_sp.	0.001265576	0.000032677	Mobiluncus_uncultured_bacterium	0.00030493	7.87326E-05	Gammaproteobacteria_R7C24uncultured_gamma	0.000442098	0.000114149
ASV_8991	0.001394014	0.000359933	ASV_5741	0.001557459	0.000402134	SelenomonasSchwartzia_sp.	0.001062844	0.000274425	Anaerovoracaceae_Family_XIII_UCG-007Peptostreptococcaceae_bacterium	0.001259592	0.000325225	Mogibacterium_timidum	0.000292504	5.60655E-05	Solobacterium_moorei	0.000439897	5.1088E-05
Methyloversatilis_sp.	0.001394014	0.000359933	Sphingomonas_panaciterrae	0.001528839	0.000394745	Aliterella_cyanobacterium_OU_20	0.001032337	0.000266548	Solobacterium_moorei	0.001258003	0.000198204	Howardella_sp.	0.000291227	5.04405E-05	Acinetobacter_baumannii	0.000423861	0.00010944
Corynebacterium_aurimucosum	0.001387203	0.000358174	Ammoniphilus_oxalaticus	0.001525447	0.000393869	Lechevalieria_sp.	0.001032337	0.000266548	ASV_6298	0.001239797	0.000320114	Prevotella_loescheii	0.000290032	5.54034E-05	Carnobacteriaceae_sp.	0.000422819	0.000109171
Psychroglaciecola_uncultured_bacterium	0.001382701	0.000357012	ASV_1592	0.001524029	0.000221662	Prevotella_histicola	0.001019065	0.000263121	Burkholderiales_SC-I-84_metagenome	0.001239797	0.000320114	Capnocytophaga_gingivalis	0.000282971	7.30628E-05	Sphingobacteriaceae_bacterium	0.000422819	0.000109171
Hungateiclostridiaceae_uncultured_bacterium	0.001382023	0.000356837	PHOS-HE36_sp.	0.001497262	0.000386591	Sandaracinobacter_uncultured_bacterium	0.00100254	0.000258855	ASV_3531	0.001219843	0.000245951	Peptoanaerobacter_stomatis	0.000282065	7.28289E-05	ASV_7533	0.000419713	7.92243E-05
Actinoplanes_garbadinensis	0.001370936	0.000353974	Chitinophagaceae_uncultured__bacterium	0.001480427	0.000278335	Anaerovoracaceae_Family_XIII_UCG-007Peptostreptococcaceae_bacterium	0.000998036	0.000257692	Anaerolineaceae_bacterium	0.001214197	0.000264709	Jonquetella_anthropi	0.000241726	6.24134E-05	Alcaligenaceae_sp.	0.000411396	0.000106222
Azorhizobium_sp.	0.001313198	0.000339066	ASV_10583	0.001465048	0.000378274	Candidatus_Limnoluna_uncultured_bacterium	0.000990115	0.000138791	ASV_5801	0.001203783	0.000310815	Haemophilus_parahaemolyticus	0.000238546	6.15923E-05	Anaerolineae_A4b_wastewater__metagenome	0.000411396	0.000106222
ASV_2180	0.001304726	0.000336879	Bacteroidales_M2PB4-65_termite_group_sp.	0.001465048	0.000378274	Gracilibacteria_marine__metagenome	0.00098241	0.000253657	Prevotella_fusca	0.001203783	0.000310815	Parvimonas_micra	0.000237168	6.12365E-05	ASV_15366	0.000411396	0.000106222
ASV_2386	0.001290056	0.000226707	Caryophanon_sp.	0.001444284	0.000372913	Eggerthia_catenaformis	0.000975771	0.000251943	Candidatus_Nostocoida_uncultured_bacterium	0.00119964	0.000309746	Gemella_asaccharolytica	0.00023566	6.08472E-05	ASV_15373	0.000411396	0.000106222
Blyi10_sp.	0.001273012	0.00032869	Lachnospiraceae_UCG-008_metagenome	0.001444284	0.000372913	Selenomonas_sputigena	0.000960285	0.000247945	Leptotrichia_hofstadii	0.0011886	0.000306895	Johnsonella_ignava	0.000209751	3.31628E-05	Sphingomonas_uncultured_bacterium	0.000411396	0.000106222
Candidatus_Saccharimonas	0.001268962	0.000275837	ASV_14404	0.001440692	0.000371985	Nitrospirillum_sp.	0.000937559	0.000242077	ASV_11492	0.001168224	0.000301634	Veillonellaceae_sp.	0.000203442	5.25285E-05	Fusobacterium_necrophorum	0.000410418	0.000105969
ASV_2061	0.001244417	0.000268139	Methyloligellaceae_sp.	0.001440692	0.000371985	Chitinophagales_37-13_uncultured_Sphingobacteriales	0.000927214	0.000239406	Peptococcus_sp.	0.001128642	0.000024922	ASV_17842	0.000192286	4.9648E-05	Armatimonadalesuncultured_Armatimonadetes	0.000401553	0.000103681
SpirochaetaSpirochaeta_sp.	0.001235203	0.000318928	ASV_4520	0.001419137	0.000338044	Oligoflexia_0319-6G20_uncultured_Geobacter	0.000927214	0.000239406	ASV_10035	0.00112495	0.000290461	Prevotella_salivae	0.000190837	4.92739E-05	ASV_9089	0.000392976	0.000101466
Hydrogenedensaceae_sp.	0.001232241	0.000318163	ASV_3021	0.00138677	0.000358062	Gardnerella_vaginalis	0.000912897	0.000235709	Aspergillus_nidulans	0.001118517	0.00002888	Lachnoanaerobaculum_uncultured_Lachnospiraceae	0.000188044	4.85528E-05	ASV_9092	0.000392976	0.000101466
Pedobacter_rivuli	0.001232241	0.000318163	ASV_2080	0.001362928	0.000351906	Sphingomonas_daechungensis	0.000891682	0.000230231	Eubacterium_brachy_group_Eubacterium_sp.	0.001100333	0.000284105	Lentimicrobium_unidentified	0.000188044	4.85528E-05	Oligoflexales_uncultured__soil	0.000381242	5.82351E-05
Streptococcus_mutans	0.001230012	0.000317588	Candidatus_Paracaedibacter_metagenome	0.001360402	0.000351254	ASV_1161	0.000872066	0.000169206	Anaerolineae_1-20_sp.	0.001070872	0.000276498	Prevotella_enoeca	0.000183098	4.72757E-05	Chitinophagaceae_uncultured_Haliscomenobacter	0.000379244	9.79204E-05
Mitochondria_Eukaryota_sp.	0.001207463	0.000131065	Kitasatospora_sp.	0.001333185	0.000344227	ASV_4520	0.000868868	0.000224341	Bacteroidetes_BD2-2_uncultured_Cytophaga	0.001070872	0.000276498	Xanthomonadaceae_Xanthomonas_sp.	0.000180314	4.14876E-05	Corynebacterium_amycolatum	0.000378211	6.96921E-05
Helcobacillus_massiliensis	0.001184684	0.000305884	ASV_2433	0.001326146	0.000342409	Exiguobacterium_undae	0.000868868	0.000224341	Desulfosporosinus_uncultured_bacterium	0.001070872	0.000276498	Treponema_parvum	0.000176916	3.59263E-05	ASV_292	0.000377625	6.67425E-05
ASV_6194	0.001175088	0.000303406	Chloroplast_uncultured_Cyanobacterium	0.001320361	0.000340916	Burkholderiaceae_sp.	0.000849939	0.000219453	Megamonasuncultured_organism	0.001047738	0.000204095	Anaerovoracaceae_Family_XIII_UCG-007Peptostreptococcaceae_bacterium	0.000175073	3.68751E-05	Micavibrionales_uncultured__bacterium	0.000374032	9.65746E-05
ASV_1592	0.00116933	0.000160917	Roseomonas_uncultured_bacterium	0.001319894	0.000340795	Colidextribacter_sp.	0.000849221	0.000219268	ASV_3026	0.001046165	0.000162815	Prevotella_buccae	0.000165055	4.2617E-05	Microlunatus_sp.	0.000374032	9.65746E-05
Candidatus_Curtissbacteria_uncultured_Microgenomates	0.001159756	0.000299448	ASV_6422	0.001312527	0.000338893	Pedobacter_namyangjuensis	0.000842066	0.000217421	ASV_10606	0.001044596	0.000269714	Johnsonella_Firmicutes_oral	0.000146516	3.78303E-05	Meiothermus_uncultured_bacterium	0.00036846	7.7114E-05
ASV_144	0.001148011	0.000296415	Fimbriimonadaceae_metagenome	0.001312527	0.000338893	Candidatus_Latescibacter_uncultured_Latescibacteria	0.000825052	0.000213028	BBMC-4_uncultured_bacterium	0.001033165	0.000266762	Leptotrichiaceae_uncultured_Leptotrichia-like_sp.	0.000143459	3.7041E-05	Acinetobacter_towneri	0.000368415	9.51243E-05
ASV_1161	0.00113	0.00013	Methylobacterium_isbiliense	0.00131	0.00033	Ferruginibacter_uncultured_Chitinophaga	0.00080	0.00020	Pseudopropionibacterium_sp.	0.00102	0.00019	Catonella_sp.	0.00013	3.43952	ASV_4143	0.00036	7.60862

	7098	2004		2527	8893		2032	7084		9259	8927		3212	E-05		3955	E-05
Rhodanobacteraceae_uncultured_Lysobacter_sp.	0.00113227	0.000292351	Moheibacter_uncultured_bacterium	0.001298027	0.000335149	ASV_2061	0.000799419	0.000110032	Meiothermus_sp.	0.001005103	0.000259516	Haemophilus_sp.	0.000125685	3.24517E-05	Peptostreptococcus_sp.	0.000359971	9.29441E-05
ASV_2103	0.001128861	0.000198698	ASV_3161	0.001248093	0.000322256	Solirubrobacter_uncultured_Solirubrobacter	0.000795284	0.000205341	Nosocomiicoccus_uncultured_bacterium	0.001005103	0.000259516	Oribacterium_sp.	0.000125685	3.24517E-05	Cellvibrio_uncultured_Cellvibrio	0.000355788	8.61825E-05
Actinomycetaceae_F0332unidentified	0.001125598	0.000290628	ASV_4673	0.001220663	0.000285838	Corynebacterium_kroppenstedtii	0.000794755	0.000205205	Actinomycetaceae_uncultured__bacterium	0.001004383	0.000259331	Centipeda_sp.	0.000122965	3.17494E-05	ASV_256	0.000348855	5.95781E-05
Fusobacterium_periodonticum	0.001125598	0.000290628	ASV_12731	0.001201346	0.000310186	Sphingomonas_astaxanthinifaciens	0.000794755	0.000205205	ASV_5066	0.000987239	0.000214477	Anaeroglobus_geminatus	0.000121273	3.13126E-05	Butyrivibrio_Eubacterium_sp.	0.000346829	6.50469E-05
Gammaproteobacteria_uncultured__sp.	0.001116249	0.000288214	ASV_3436	0.001201346	0.000310186	Scardovia_wiggsiae	0.000790065	0.000141799	Porphyromonas_catoniae	0.000979695	0.000158816	Anaerovoracaceae_Family_XIII_UCG-006Peptostreptococcaceae_bacterium	0.000121107	1.88601E-05	Armatimonadales_uncultured_eubacterium	0.000343854	8.87827E-05
ASV_2850	0.001112231	0.000205309	Lactobacillus_perolens	0.001166691	0.000301238	ASV_4746	0.000777374	0.000200717	ASV_3630	0.000978699	0.000144436	Treponema_vincentii	0.000117213	3.02643E-05	ASV_1592	0.000341136	3.7687E-05
Leptospira_sp.	0.001077164	0.000278123	ASV_2103	0.001151109	0.000297215	Planctomycetes_WD2101_soil_group_uncultured_planctomycete	0.000764299	0.000197341	ASV_13022	0.00097352	0.000251362	Alloprevotella_rava	0.000111321	2.8743E-05	ASV_5620	0.000323273	3.96421E-05
Tetrodontophora_bielanensis	0.001077164	0.000278123	CK-2C2-2_uncultured_organism	0.001151109	0.000297215	Howardella_sp.	0.000757247	0.000111217	Cloacibacterium_sp.	0.00097352	0.000251362	Prevotella_marshii	0.000107876	2.28032E-05	Myxococcaceae_P3OB-42_metagenome	0.000319293	8.24411E-05
Pedosphaeraceae_uncultured_bacterium	0.001075434	0.000277676	Silanimonas_uncultured_bacterium	0.001111883	0.000287087	Legionella_norrlandica	0.000755501	0.00019507	ASV_2465	0.000964885	0.000169882	Actinomycetaceae_uncultured__bacterium	0.000105959	2.73585E-05	Sediminibacterium_metagenome	0.00031742	8.19575E-05
Woesearchaeales_GW2011_GWC1_47_15_sp.	0.001075434	0.000277676	ASV_15347	0.001109416	0.00028645	Eubacterium_sp.	0.000735196	0.000189827	Anoxybacillus_sp.	0.000964243	0.000248966	Solobacterium_uncultured_organism	0.000100654	2.59888E-05	ASV_2692	0.000312319	8.06404E-05
ASV_13432	0.001072386	0.000276889	Jeotgalibaca_uncultured_bacterium	0.001103323	0.000284877	Macrococcus_sp.	0.000735196	0.000189827	Fibrobacteraceae_uncultured_Juncus_effusus	0.000964243	0.000248966	uncultured_Capnocytophaga	9.52353E-05	2.45896E-05	Rhizobiales_sp.	0.000308983	7.97791E-05
Demequina_sp.	0.001066011	0.000275243	Microcoleus_PCC-7113_Microcoleus_sp.	0.001103323	0.000284877	Deinococcus_antarcticus	0.000725426	0.000187304	Paenibacillus_metagenome	0.000964243	0.000248966	ASV_2285	0.000094264	2.43389E-05	Bacteroides_uncultured_bacterium	0.000308547	7.96665E-05
Gordonia_sp.	0.001040402	0.000268631	Curtobacterium_sp.	0.001079914	0.000278833	Desulfitobacteriales_TC1uncultured_Firmicutes	0.000724022	0.000186942	Caenarcaniphilales_uncultured_Firmicutes	0.000951098	0.000245572	Selenomonadaceae_uncultured_Selenomonas_sp.	0.000087382	2.25619E-05	Caulobacteraceae_PMMR1Caulobacter_sp.	0.000308547	7.96665E-05
PeredibacterPeredibacter_starrii	0.001040402	0.000268631	Eubacterium_coprostanoligenes_group_gut__metagenome	0.001079914	0.000278833	Propionivibrio_uncultured_soil	0.000724022	0.000186942	Gaiella_uncultured_actinobacterium	0.000951098	0.000245572	Slackia_exigua	0.000082871	1.23839E-05	Christensenellaceae_R-7_group_uncultured_rumen	0.000308547	7.96665E-05
Solobacterium_moorei	0.001019399	0.000151768	Bacillus_infernus	0.001069473	0.000276137	ASV_2850	0.000714965	0.000110087	Sumerlaea_metagenome	0.000933323	0.000240983	Oribacterium_uncultured_organism	8.19765E-05	2.11662E-05	Polyangia_sp.	0.000308547	7.96665E-05
Anaerolineaceae_uncultured_toluene-degrading_methanogenic	0.001015443	0.000262186	Phycisphaera_metagenome	0.001069473	0.000276137	ASV_8590	0.000695087	0.000155466	Citrifermentans_uncultured_delta	0.00093007	0.000240143	Capnocytophaga_haemolytica	8.13769E-05	2.10114E-05	Uliginosibacterium_sp.	0.000308547	7.96665E-05
ASV_8760	0.001010101	0.000260807	ASV_8124	0.001056847	0.000272877	Leptotrichia_shahii	0.000688795	0.000153061	Leptotrichia_shahii	0.000925256	0.000196385	Mycoplasma_spermatophilum	8.13769E-05	2.10114E-05	Blastocatellaceae_JGI_0001001-H03_sp.	0.000307813	7.9477E-05
Anaerolineae_uncultured__bacterium	0.00100994	0.000260765	ASV_5802	0.001048139	0.000206509	Acidimicrobiia_uncultured_bacterium_YC-ZSS-LKJ199	0.00066836	0.00017257	ASV_3228	0.000910581	0.000171671	ASV_2084	8.05837E-05	2.08066E-05	SAR324_clade(Marine_group_B)_uncultured_soil	0.000294732	7.60995E-05
CL500-3_uncultured_bacterium	0.00100994	0.000260765	ASV_13525	0.001046463	0.000270196	ASV_13011	0.000662296	0.000171004	ASV_1208	0.00089973	0.000232309	Prevotellaunidentified	7.95153E-05	2.05308E-05	ASV_4673	0.000292721	7.55802E-05
ASV_5948	0.001004128	0.000259265	ASV_2061	0.001027613	0.000195457	Empedobacter_sp.	0.00065494	0.000169105	ASV_7512	0.000883889	0.000228219	Actinomyces_dentalis	7.94691E-05	2.05188E-05	Saccharomonospora_sp.	0.000292721	7.55802E-05
Chloroplast_Mychonastes_jurisii	0.00098574	0.000176115	Acidimicrobiia_IMCC26256_metagenome	0.000999889	0.00025817	Eubacterium_brachy_group__sp.	0.000629584	0.000162558	ASV_13900	0.000876168	0.000226226	Lawsonella_uncultured_bacterium	6.98516E-05	1.07245E-05	ASV_5322	0.000290713	7.50618E-05
ASV_12760	0.00097924	0.000252839	Lactobacillus_iners	0.000972898	0.000251201	Eubacterium_brachy_group_Eubacterium_sp.	0.000622153	0.000160639	Jatrophihabitans_uncultured_actinobacterium	0.000876168	0.000226226	Saccharimonadales_TM7auncultured_Candidatus	6.79638E-05	1.75482E-05	Pyrinomonadaceae_RB41_metagenome	0.000290713	7.50618E-05
Chitinophagales_uncultured__bacterium	0.00097924	0.000252839	ASV_16467	0.000970739	0.000250644	ASV_5922	0.000617326	0.000159393	Solirubrobacteraceae_uncultured__metagenome	0.000876168	0.000226226	Gemella_sp.	6.77622E-05	1.74961E-05	Tyzzerella_uncultured_Firmicutes	0.000290713	7.50618E-05
Rhodoplanes_sp.	0.00097924	0.000252839	Candidatus_Nomurabacteria_Parcubacteria_bacterium	0.000970739	0.000250644	Oscillospiraceae_UCG-007_uncultured_rumen	0.000617326	0.000159393	Roseburia_Lachnospiraceae_bacterium	0.000875923	0.000226162	Anaerolineaceae_bacterium	6.62243E-05	1.7099E-05	Luteococcus_sp.	0.000290001	7.48803E-05
Thermoleophilia_uncultured_Conexibacter	0.00097924	0.000252839	Glutamicibacter_uncultured_bacterium	0.000970739	0.000250644	Gracilibacteria_Gracilibacteria_bacterium	0.000611924	0.000157998	Corynebacterium_amycolatum	0.000871941	0.000225134	Treponema_refringens	6.59321E-05	1.70236E-05	ASV_1777	0.000281144	4.40594E-05
Candidatus_Limnoluna_uncultured_bacterium	0.000969311	0.000219544	Roseimarinus_metagenome	0.000961076	0.000248149	Candidatus_Solibacter_sp.	0.000601524	0.000155313	Sphingobacteriales_NS11-12_marine_groupuncultured_Sphingobacterium	0.000871941	0.000225134	Prevotella_sp.	6.36122E-05	1.64246E-05	Acidimicrobiia_IMCC26256uncultured_Aciditerrimonas	0.000276459	7.13814E-05
Ferritrophicum_uncultured_Rhodocyclaceae	0.000965147	0.0002492	Candidatus_Flaviluna_uncultured_actinobacterium	0.000960461	0.00024799	Winkia_neuii	0.000601524	0.000155313	Kiritimatiellae_WCHB1-41uncultured_prokaryote	0.00087184	0.000225108	Johnsonella_uncultured_bacterium	6.28427E-05	1.62259E-05	Cellvibrio_uncultured_gamma	0.000276459	7.13814E-05
Roseiflexaceae_uncultured__bacterium	0.000950683	0.000245465	Candidatus_Hamiltonella_sp.	0.000959923	0.000247851	Treponema_lecithinolyticum	0.000596229	0.000153946	ASV_12772	0.000859845	0.000222011	ASV_1071	6.10327E-05	1.57586E-05	ASV_11581	0.000267702	6.91204E-05
Corynebacterium_amycolatum	0.000949137	0.000218207	Lenti-02_metagenome	0.000959923	0.000247851	ASV_13883	0.000596066	0.000153904	Prevotellaceae_UCG-003_sp.	0.000859845	0.000222011	Porphyromonas_uenonis	6.10327E-05	1.57586E-05	Peptococcus_sp.	0.000257249	5.28396E-05
Treponema_stenostreptum	0.00093	0.00024219	Methylobacterium_hispanicum	0.00095	0.00024	Stomatobaculum_sp.	0.00059	0.00015	Actinomyces_dentalis	0.00085	0.00017	Fusobacterium_sp.	5.82547	1.50413	ASV_18970	0.00025	6.63886

	7998			9923	7851		5386	3728		1614	7421		E-05	E-05		7122	E-05
ASV_17824	0.000921801	0.000238008	Pseudoxanthomonas_metagenome	0.000959923	0.000247851	Cytophagales_sp.	0.000594462	0.000153489	Chloroplast_Melosira_varians	0.00083307	0.000149641	Lachnospiraceae_uncultured__sp.	5.12805E-05	1.32406E-05	Bacteriovoracaceae_uncultured_delta	0.000257122	6.63886E-05
ASV_17828	0.000921801	0.000238008	Actinobacteria_PeM15uncultured_actinobacterium	0.000941817	0.000243176	Lachnospiraceae_NK3A20_group_uncultured_Lachnospiraceae	0.000594462	0.000153489	Alistipes_gut__metagenome	0.000826532	0.00021341	Leptotrichia_goodfellowii	5.12805E-05	1.32406E-05	CCM19a_uncultured_bacterium	0.000257122	6.63886E-05
Nitrosomonadaceae_GOUTA6_uncultured_bacterium	0.000921801	0.000238008	Phycicoccus_sp.	0.000941817	0.000243176	Gemmobacter_sp.	0.000594455	0.000153488	Bdellovibrio__uncultured_soil	0.000826532	0.00021341	Corynebacterium_amycolatum	5.03271E-05	1.29944E-05	Luteibacter_sp.	0.000257122	6.63886E-05
Flavobacterium_unidentified	0.000915893	0.000169163	BSV13_uncultured_bacterium	0.000934117	0.000241188	Slackia_exigua	0.000575945	9.63012E-05	ASV_1592	0.000826296	0.000123333	Streptococcus_sp.	5.03271E-05	1.29944E-05	Olsenella_uncultured_rumen	0.000257122	6.63886E-05
Ralstonia_sp.	0.000913315	0.000199202	Eubacterium_coprostanoligenes_group_human_gut	0.000934117	0.000241188	Azospirillaceae_sp.	0.000571598	0.000147586	Bacteroidales_M2PB4-65_termite_group_metagenome	0.000813812	0.000210125	Cardiobacterium_valvarum	4.85456E-05	1.25344E-05	Runella_slithyformis	0.000257122	6.63886E-05
ASV_164	0.000902009	0.000232898	Sphingomonadaceae_uncultured_alpha_proteobacterium	0.000908619	0.000234604	Kiloniella_sp.	0.000571598	0.000147586	oligoflexales_uncultured__uncultured_soil	0.000803536	0.000207472	Firmicutes_sp.	0.000047132	1.21694E-05	ASV_12313	0.000245393	6.33602E-05
Hydrogenedensaceae_metagenome	0.000902009	0.000232898	Faecalibacterium_metagenome	0.000867546	0.000214378	Sphingomonas_phyllosphaerae	0.000571598	0.000147586	Absconditabacteriales_(SR1)_uncultured_organism	0.00079976	0.000206497	ASV_6	4.59079E-05	8.18652E-06	ASV_4520	0.000245393	6.33602E-05
Rubrobacter_uncultured_Rubrobacter	0.000902009	0.000232898	Ketobacter_uncultured_soil	0.000840942	0.00021713	ASV_10003	0.000562535	0.000145246	Flavihumibacter_uncultured_bacterium	0.00079976	0.000206497	Blvii28_wastewater-sludge_group_sp.	4.39548E-05	1.13491E-05	ASV_4506	0.00024422	6.30573E-05
Coriobacteriia_OPB41_uncultured_soil	0.000889136	0.000193113	Rhizobiales_Incertae_Sedis_uncultured__bacterium	0.000840942	0.00021713	ASV_10004	0.000562535	0.000145246	Campylobacter_concisus	0.000785423	0.000142681	Tannerella_forsythia	3.71885E-05	9.60203E-06	Nannocystis_exedens	0.000243934	6.29835E-05
Fimbriimonadaceae_sp.	0.000881316	0.000227555	Rickettsiales_SM2D12uncultured_Bradyrhizobium	0.000840942	0.00021713	Elusimicrobiota_Lineage_IIa_metagenome	0.000561423	0.000144959	ASV_14881	0.000778816	0.000201089	Erysipelotrichaceae_UCG-006_uncultured_bacterium	0.000036629	9.45757E-06	ASV_2303	0.000242613	4.30837E-05
Bergeyella_uncultured_bacterium	0.000867002	0.000223859	ASV_15928	0.000839933	0.00021687	ASV_7817	0.00055802	0.00014408	Inquilinus_sp.	0.000778816	0.000201089	Anaerovoracaceae_sp.	3.18061E-05	8.2123E-06	ASV_2862	0.000236951	5.30332E-05
Xanthobacteraceae_uncultured__sp.	0.000867002	0.000223859	Enterococcus_sp.	0.000839933	0.00021687	Pseudoxanthomonas_mexicana	0.00055802	0.00014408	Oligoflexus_uncultured_delta	0.000777407	0.000141704	Prevotella_oris	3.18061E-05	8.2123E-06	Thiothrix_sp.	0.00022589	5.83245E-05
Larkinella_metagenome	0.00086334	0.000222913	Holophagaceae_uncultured_bacterium	0.00083717	0.000216156	Rhodomicrobium_uncultured_bacterium	0.000548734	0.000141683	Ignavibacteria_sp.	0.000773861	0.00019981	Prevotellaceae_uncultured_Prevotella	3.18061E-05	8.2123E-06	Prevotella_nigrescens	0.000225648	3.12314E-05
Oligoflexales_uncultured__soil	0.000861703	0.000153221	Marmoricola_sp.	0.000832062	0.000214837	ASV_1592	0.000547572	8.20965E-05	Johnsonella_sp.	0.000773861	0.00019981	Fretibacteriumuncultured_Fretibacterium	3.05547E-05	5.38078E-06	ASV_3526	0.000223085	5.76003E-05
Nosocomiicoccus_ampullae	0.000855368	0.000220855	Prevotellaceae_sp.	0.000832062	0.000214837	ASV_8922	0.000535147	0.000138174	Spirochaetaceae_uncultured__metagenome	0.000773861	0.00019981	Anaerococcus_octavius	2.92539E-05	7.55332E-06	Chloroplast_uncultured_diatom	0.00022231	5.74002E-05
ASV_3100	0.000846203	0.000218489	Paenibacillus_taihuensis	0.000800384	0.000206658	ASV_14482	0.000534688	0.000138056	bacteriap25_uncultured_bacterium	0.000773156	0.000199628	Prevotella_oulorum	2.92539E-05	7.55332E-06	Mycoplasmataceae_genomosp.	0.00022231	5.74002E-05
Chryseobacterium_taklimakanense	0.000846203	0.000218489	Candidatus_Xiphinematobacter_uncultured_Candidatus	0.000799574	0.000206449	Bifidobacteriaceae_uncultured__sp.	0.000534688	0.000138056	Chamaesiphon_PCC-7430_sp.	0.000773156	0.000199628	Peptoniphilus_lacrimalis	2.91273E-05	7.52064E-06	Elusimicrobia_MVP-88_sp.	0.000222194	5.73702E-05
Oligoflexus_uncultured_delta	0.000844913	0.000148722	ASV_2510	0.000777054	0.000144461	Magnetospirillaceaeuncultured_proteobacterium	0.000534688	0.000138056	ASV_3256	0.000752584	0.000194316	Olsenella_genomosp.	2.84848E-05	7.35474E-06	ASV_13612	0.000221049	5.70746E-05
Pseudoalteromonas_sp.	0.000844198	0.000217971	ASV_5779	0.000761952	0.000178642	Pseudorhodoplanes_sp.	0.000534688	0.000138056	Cupriavidus_sp.	0.000723215	0.000186733	Stomatobaculum_Eubacterium_sp.	2.64897E-05	6.83961E-06	Acidovorax_sp.	0.000211613	5.46382E-05
WOR-1_sp.	0.000823469	0.000212619	Streptococcus_mutans	0.000746269	0.000192686	Rhizobiales_A0839__uncultured_bacterium	0.000534688	0.000138056	Meiothermus_uncultured_bacterium	0.000723182	0.000186725	Granulicatella_sp.	2.04941E-05	5.29155E-06	ASV_5801	0.00021141	5.45858E-05
ASV_13403	0.000820008	0.000211725	Saccharofermentans_uncultured_microorganism	0.000732524	0.000189137	Solirubrobacteraceae_uncultured_Phytoplasma_sp.	0.000534688	0.000138056	Aeromonas__metagenome	0.000713323	0.000184179	Porphyromonas_sp.	1.94182E-05	5.01376E-06	ASV_20250	0.000205698	5.3111E-05
ASV_13405	0.000820008	0.000211725	Rhodospirillaceae_uncultured__bacterium	0.000729182	0.000188274	Aminobacter_sp.	0.000529836	0.000136803	Legionellaceae_uncultured__bacterium	0.00069979	0.000180685	ASV_9	1.85943E-05	4.80103E-06	ASV_2249	0.000205698	5.3111E-05
Elusimicrobiota_Lineage_IIauncultured_actinobacterium	0.000820008	0.000211725	ASV_2850	0.00072643	0.000136026	Spirosoma_uncultured_Bacteroidetes	0.000529836	0.000136803	ASV_14382	0.00069584	0.000179665	ASV_2888	1.46516E-05	3.78303E-06	Azospirillaceae_uncultured__metagenome	0.000205698	5.3111E-05
Planctopirus_uncultured_planctomycete	0.000820008	0.000211725	Anaerobiospirillum_succiniciproducens	0.000720807	0.000186112	ASV_5304	0.000525871	0.000135779	Chloroflexi_KD4-96uncultured_Chloroflexi	0.000687876	0.000177609	Catonella_unidentified	1.46516E-05	3.78303E-06	Chloroplast_Guillardia_theta	0.000205698	5.3111E-05
Stenotrophomonas_nitritireducens	0.000820008	0.000211725	ASV_10613	0.000720807	0.000186112	ASV_3349	0.000525033	0.000135563	Edaphobaculum_uncultured_Chitinophaga	0.000687876	0.000177609	Eubacterium_brachy_group_Eubacterium_sp.	1.46516E-05	3.78303E-06	Cyclobacteriaceae_sp.	0.000205698	5.3111E-05
Herpetosiphon_sp.	0.000798316	0.000206124	Enterococcus_cecorum	0.000719942	0.000185888	Roseomonas_metagenome	0.000525033	0.000135563	Flavobacterium_lacicola	0.000687876	0.000177609	Saccharimonadales_TM7a_uncultured_bacterium	1.46516E-05	3.78303E-06	Elusimicrobiota_Lineage_IIc_uncultured_bacterium	0.000205698	5.3111E-05
PRD18C08_uncultured_bacterium	0.000798316	0.000206124	ASV_8228	0.000714325	0.000184438	Eubacterium_brachy_group__uncultured_bacterium	0.000514596	0.000132868	Metamycoplasma_salivarium	0.000683246	9.28502E-05	Anaerococcusuncultured_organism	1.46269E-05	3.77665E-06	Blastocatella_uncultured_bacterium	0.000205209	5.29847E-05
Candidatus_Limnoluna_sp.	0.000797333	0.00020587	Microtrichales_uncultured_Iamiaceae	0.000713915	0.000184332	Flavobacterium_rivuli	0.000514596	0.000132868	ASV_15912	0.000681464	0.000175953	009E01-B-SD-P15__uncultured_bacterium	0	0	Gaiellales_uncultured_Rubrobacteria	0.000205209	5.29847E-05
Chloroplast_Taxus_mairei	0.000797321	0.000205867	Rikenellaceae_uncultured__sp.	0.000713915	0.000184332	Paracaedibacteraceae_uncultured__metagenome	0.000514596	0.000132868	Eubacterium_brachy_group_uncultured_Firmicutes	0.000681464	0.000175953	1013-28-CG33__uncultured_soil	0	0	ASV_8157	0.000201033	5.19065E-05
Anaerovorax_sp.	0.000783392	0.000202271	Saprospiraceae_uncultured_Devosia	0.000713915	0.000184332	Saccharimonadales_TM7aTM7_phylum	0.000514596	0.000132868	Bilophila_sp.	0.000671993	0.000173508	Abditibacterium_sp.	0	0	Polymorphobacter_uncultured_Sphingomonas	0.000201033	5.19065E-05

Chloroplast_uncultured_Cryptomonadaceae	0.000783392	0.000202271	Flaviaesturariibacter_sp.	0.000694927	0.000179429	ASV_2386	0.000514195	0.000106352	Chelativoransuncultured_Alphaproteobacteria	0.000642828	0.000165977	Absconditabacteriales_(SR1)__metagenome	0	0	Babeliales_UBA12411_uncultured_bacterium	0.000200776	5.18401E-05
Myroides_sp.	0.000783392	0.000202271	Cloacibacterium_sp.	0.000693385	0.000179031	Cutibacterium_granulosum	0.000511551	7.49032E-05	Advenella_sp.	0.000639423	0.000165098	Absconditabacteriales_(SR1)__uncultured_bacterium	0	0	Clostridia_UCG-014unidentified_rumen	0.000200776	5.18401E-05
Rhizobacter_sp.	0.000783392	0.000202271	Desulfobulbus_uncultured_Desulfobulbus	0.000693385	0.000179031	Methylacidiphilaceae_uncultured_candidate_division	0.000509533	0.000131561	Filifactor_alocis	0.000639423	0.000165098	Absconditabacteriales_(SR1)_candidate_division	0	0	Corynebacterium_glutamicum	0.000200776	5.18401E-05
Leeia_uncultured_bacterium	0.000771844	0.000122807	Anaeromusa-Anaeroarcus_sp.	0.000666593	0.000172114	ASV_2160	0.000508553	6.22522E-05	ASV_8997	0.000063466	0.000163869	Absconditabacteriales_(SR1)_sp.	0	0	Steroidobacteraceae_uncultured_Hypsibius_dujardini	0.000200776	5.18401E-05
Acholeplasma_laidlawii	0.000768167	0.00019834	ASV_17312	0.000666593	0.000172114	ASV_9358	0.000503667	0.000130046	Aquabacterium_sp.	0.000619899	0.000160057	Absconditabacteriales_(SR1)_SR1_bacterium	0	0	SBR1031anaerobic_bacterium	0.000196609	5.07642E-05
Antricoccus_suffuscus	0.000768167	0.00019834	Iodobacter_uncultured_beta	0.000659377	0.00017025	Chloroflexaceae_FFCH7168uncultured_compost	0.000503007	0.000129876	Methylophilaceae_uncultured_beta	0.000619899	0.000160057	Absconditabacteriales_(SR1)_uncultured_candidate	0	0	Tessaracoccus_metagenome	0.000195147	5.03867E-05
Cryptobacterium_sp.	0.000768167	0.00019834	ASV_1208	0.000655799	0.000105973	Kryptoniales_MSB-3C8_sp.	0.000503007	0.000129876	ASV_15570	0.000601892	0.000155408	Absconditabacteriales_(SR1)_uncultured_Epsilonproteobacteria	0	0	Microgenomatia_uncultured_soil	0.000188108	4.85693E-05
Dysgonomonas_sp.	0.000768167	0.00019834	Actinomycetospora_sp.	0.000649013	0.000167574	Chloroplast_Melosira_varians	0.000502274	6.29698E-05	ASV_15576	0.000601892	0.000155408	Absconditabacteriales_(SR1)_uncultured_organism	0	0	Flexibacter_sp.	0.000184723	3.99662E-05
Alkanindiges_uncultured_bacterium	0.000755423	0.000195049	Thermoleophilia_uncultured_Gaiella_sp.	0.000649013	0.000167574	ASV_4353	0.000048753	0.000012588	LD29_uncultured_Verrucomicrobia	0.000601892	0.000155408	Absconditabacteriales_(SR1)_uncultured_prokaryote	0	0	Paludibaculum_fermentans	0.000178885	4.61879E-05
ASV_1901	0.000755423	0.000195049	ASV_2249	0.000641684	0.000165682	Parvimonas_sp.	0.000048099	7.48497E-05	Runella_metagenome	0.000601892	0.000155408	ABY1_sp.	0	0	ASV_3026	0.000178468	4.60802E-05
ASV_15004	0.000750399	0.000193752	Methylophilaceae_uncultured__sp.	0.000641684	0.000165682	Prevotella_denticola	0.000480143	0.000123972	Planctomicrobium_uncultured_bacterium	0.000059982	0.000154873	Acanthopleuribacter__uncultured_bacterium	0	0	Virgibacillus_sp.	0.000178468	4.60802E-05
Sphingobacteriaceae_uncultured__bacterium	0.000750399	0.000193752	FCPU426_metagenome	0.000640307	0.000165327	Lactobacillus_malefermentans	0.000474317	0.000122468	ASV_4770	0.000597327	0.000154229	Acetitomaculum__uncultured_bacterium	0	0	Clostridium_islandicum	0.000172032	4.44185E-05
Oligoflexia_uncultured_proteobacterium	0.000744166	0.000192143	Candidatus_Moranbacteria_uncultured_bacterium	0.000627878	0.000162117	Peptococcus_sp.	0.000047287	6.21633E-05	Alphaproteobacteria_uncultured_gut__metagenome	0.000584112	0.000150817	Acetitomaculum__uncultured_rumen	0	0	Nannocystis_unidentified	0.000172032	4.44185E-05
Robbsia_andropogonis	0.000738007	0.000190553	Comamonadaceae_uncultured__sp.	0.000627878	0.000162117	Bacillus_thermoamylovorans	0.000472188	0.000121918	ASV_17109	0.000584112	0.000150817	Acetitomaculum_sp.	0	0	Candidatus_Xiphinematobacter_sp.	0.000171927	4.43914E-05
Fusibacter_uncultured_bacterium	0.000720535	0.000186041	Kazania_uncultured_bacterium	0.000627878	0.000162117	ASV_15470	0.000467852	0.000120799	Bacteroidetes_BD2-2_uncultured_sediment	0.000584112	0.000150817	Acetivibrio_uncultured_bacterium	0	0	Micavibrionaceae_uncultured__bacterium	0.000171927	4.43914E-05
Ruminococcus_torques_group_sp.	0.000720535	0.000186041	Planococcaceae_sp.	0.000627878	0.000162117	bacterium_endosymbiont	0.000467852	0.000120799	Gammaproteobacteria_CHAB-XI-29_uncultured_bacterium	0.000584112	0.000150817	Acetoanaerobium_uncultured_bacterium	0	0	Qipengyuania_sp.	0.000171927	4.43914E-05
Spongiibacteraceae_BD1-7_cladeuncultured_Haliea	0.000720535	0.000186041	Actinomycetaceae_F0332unidentified	0.000625821	0.000134917	Sediminibacterium_ginsengisoli	0.000467852	0.000120799	Marvinbryantia_uncultured_bacterium	0.000584112	0.000150817	Acetobacter_sp.	0	0	Variovorax_sp.	0.000171927	4.43914E-05
Sporichthyaceae_hgcl_clade_uncultured_actinobacterium	0.000720535	0.000186041	Chthonobacter_uncultured_bacterium	0.000625434	0.000161486	Sutterella_sp.	0.000467852	0.000120799	Methylophilus_sp.	0.000584112	0.000150817	Acetobacteraceae_sp.	0	0	Lachnospiraceae_uncultured_Lachnospiraceae	0.000171007	4.41538E-05
ASV_7762	0.000071945	0.000185761	Collinsella_aerofaciens	0.000625434	0.000161486	Frankiales_uncultured_bacterium	0.000467814	0.000120789	Propionibacteriaceae_uncultured_Propionibacteriaceae	0.000584112	0.000150817	Acetobacteraceae_uncultured__bacterium	0	0	Rhodothermaceae_uncultured_Rhodothermaceae	0.000162623	4.19891E-05
Finegoldia_uncultured_bacterium	0.000713012	0.000184099	Woesearchaeales_AR15_metagenome	0.000625434	0.000161486	Nitrosarchaeum_sp.	0.000467814	0.000120789	Saccharimonadales_WWH38uncultured_Candidatus	0.000584112	0.000150817	Acetobacteraceae_uncultured__metagenome	0	0	Kocuria_marina	0.000016135	4.16604E-05
Candidatus_Endoecteinascidia_sp.	0.000693602	0.000179087	Papillibacteruncultured_Clostridiaceae	0.000604429	0.000156063	Oscillospiraceae_sp.	0.000467814	0.000120789	Acremonium_fuci	0.000562475	0.000014523	Acetobacteraceae_uncultured__sp.	0	0	Paenibacillus_phoenicis	0.000159744	4.12457E-05
Lachnospiraceae_NK3A20_group_uncultured_Firmicutes	0.000691012	0.000178419	ASV_18343	0.000599952	0.000154907	ASV_12478	0.000467071	0.000120597	ASV_2910	0.000562475	0.000014523	Acetobacteraceae_uncultured_alpha_proteobacterium	0	0	ASV_1161	0.000159731	3.55677E-05
ASV_15543	0.000685468	0.000176987	Blfdi19_uncultured_prokaryote	0.000599952	0.000154907	Roseburia_gut__metagenome	0.000467071	0.000120597	Mitochondria_Eukaryota_sp.	0.000562475	0.000014523	Acetobacterium_sp.	0	0	Flavobacterium_paronense	0.000156159	4.03201E-05
ASV_15546	0.000685468	0.000176987	Candidatus_Planktophila_sp.	0.000599952	0.000154907	Acinetobacter__metagenome	0.000463607	0.000119703	Gammaproteobacteria_211ds20__uncultured_bacterium	0.000559994	0.000014459	Acetothermiia__uncultured_bacterium	0	0	Xanthobacteraceae_Xanthobacter_sp.	0.000156159	4.03201E-05
ASV_5386	0.000685468	0.000176987	Clavibacter_sp.	0.000599952	0.000154907	Moraxella_atlantae	0.000463607	0.000119703	Methylobacterium_adhaesivum	0.000559994	0.000014459	Acholeplasma__uncultured_bacterium	0	0	Anaeromyxobacter_uncultured_bacterium	0.000154273	3.98331E-05
Chlamydiaceae_uncultured_Chlamydiales	0.000685468	0.000176987	Clostridium_perfringens	0.000599952	0.000154907	Phreatobacter_uncultured_Alphaproteobacteria	0.000463607	0.000119703	Prevotella_loescheii	0.000055871	0.000124736	Acholeplasma_axanthum	0	0	ASV_2433	0.000154273	3.98331E-05
Gastranaerophilales_uncultured_Merismopedia	0.000685468	0.000176987	Puia_uncultured_soil	0.000599952	0.000154907	Sericytochromatia_uncultured_soil	0.000463607	0.000119703	Eggerthella_uncultured_bacterium	0.000554807	0.000143251	Acholeplasma_brassicae	0	0	Berkelbacteria_candidate_division	0.000154273	3.98331E-05
Hymenobacter_uncultured_bacterium	0.000685468	0.000176987	Sandaracinus_metagenome	0.000598905	0.000154637	ASV_2390	0.000454595	0.000105475	Olsenella_umbonata	0.000541209	0.000013974	Acholeplasma_laidlawii	0	0	Candidatus_Woesebacteria	0.000154273	3.98331E-05
Thermoplasmata_Marine_Group_II_uncultured_archaeon	0.000685334	0.000176952	ASV_2386	0.000588704	0.000133979	ASV_2465	0.000434415	0.000112165	Paracaedibacteraceae_uncultured_Alphaproteobacteria	0.000541209	0.000013974	Acholeplasma_morum	0	0	Cytophaga_uncultured_Sphingobacteriia	0.000154273	3.98331E-05
ASV_8117	0.000683478	0.000176473	ASV_2180	0.000586354	0.000151396	ASV_8102	0.000434415	0.000112165	Shinella_sp.	0.000541209	0.000013974	Acholeplasma_sp.	0	0	Dadabacteriales_uncultured_bacterium	0.000154273	3.98331E-05

Rhodobacteraceae_Yoonia-LoktanellaLoktanella_fryxellensis	0.000683478	0.000176473	Leptotrichia_hofstadii	0.00058549	0.000151173	Prevotella_baroniae	0.000434415	0.000112165	ASV_4563	0.000523165	0.000135081	Acholeplasmataceae_EMP-G18_uncultured_Mollicutes	0	0	Mitochondriaunidentified_proteobacterium	0.000154273	3.98331E-05
Prevotella_pallens	0.000665753	0.000171897	Cutibacterium_sp.	0.000584112	0.000150817	ASV_5779	0.000433035	6.74115E-05	Micropruina_sp.	0.000523165	0.000135081	Acholeplasmauncultured_Acholeplasmataceae	0	0	Paludibacteraceae_uncultured__uncultured_soil	0.000154273	3.98331E-05
Leptotrichia_shahii	0.000665138	0.000118124	Eggerthellaceae_uncultured__metagenome	0.000584112	0.000150817	Anaerococcusuncultured_organism	0.000430715	8.19993E-05	Rhizobiales_Incertae_Sedis_uncultured_prokaryote	0.000523165	0.000135081	Achromatium_sp.	0	0	Saprospiraceae_uncultured_Saprospiraceae	0.000154273	3.98331E-05
Deefgea_uncultured_bacterium	0.000656599	0.000169533	RS25G_metagenome	0.000584112	0.000150817	Lachnospiraceae_uncultured_human_gut	0.00042461	0.000109634	Johnsonella_ignava	0.000517255	9.23451E-05	Acidaminobacter__uncultured_bacterium	0	0	Prevotellaceae_UCG-001_uncultured_rumen	0.000147456	3.8073E-05
Shinella_sp.	0.000653595	0.000168758	Christensenellaceae_R-7_group_uncultured_bacterium	0.000583345	0.000150619	Micrococcaceae_Yaniella_uncultured_bacterium	0.000421033	0.00010871	Candidatus_Magasanikbacteria_metagenome	0.000516582	0.000133381	Acidaminococcaceae_uncultured__bacterium	0	0	Phormidiaceae_uncultured__bacterium	0.000146484	3.7822E-05
Kocuria_marina	0.000644789	0.000133323	Opitutus_metagenome	0.000583345	0.000150619	Eubacterium_minutum	0.000418515	0.00010806	Methylobacillus_sp.	0.000516582	0.000133381	Acidibacter__metagenome	0	0	Planctomycetes_Pla4_lineageuncultured_prokaryote	0.000146361	3.77902E-05
Candidatus_Melainabacteria	0.000643432	0.000166133	Sporichthya_metagenome	0.000583345	0.000150619	Prolixibacteraceae_uncultured__sp.	0.000413462	0.000106755	Alistipes_uncultured_Alistipes	0.000515907	0.000133207	Acidibacter__uncultured_bacterium	0	0	ASV_1837	0.000144123	3.44724E-05
Gemmataceae_uncultured_bacterium_enrichment	0.000643432	0.000166133	ASV_2160	0.000579714	0.000108146	Carnobacterium_sp.	0.000412526	0.000106514	ASV_16736	0.000515907	0.000133207	Acidibacter_sp.	0	0	Pyrinomonadaceae_RB41_sp.	0.000140536	2.57431E-05
Candidatus_Nitrocosmicus_sp.	0.000634652	0.000163866	Sporichthyaceae_hgcI_clade_uncultured_Actinomycetales	0.000556126	0.000143591	Sphingobacterium_rhinocerotis	0.000412526	0.000106514	ASV_2862	0.000515907	0.000133207	Acidimicrobiia_IMCC26256_metagenome	0	0	Acholeplasma_brassicae	0.000138667	3.58037E-05
ASV_16643	0.000617602	0.000159464	ASV_15198	0.000555942	0.000143544	Z4MB62_Z4MB62_uncultured_bacterium	0.000412526	0.000106514	Berkelbacteria_uncultured_soil	0.000515907	0.000133207	Acidimicrobiia_IMCC26256_sp.	0	0	Paenibacillus_glucanolyticus	0.000138667	3.58037E-05
Chthoniobacter_uncultured_bacterium	0.000617602	0.000159464	Methylobacterium-Methylorubrum_uncultured_Alphaproteobacteria	0.000555942	0.000143544	ASV_1208	0.00040923	0.000105663	Patescibacteria_WWE3uncultured_anaerobic	0.000515907	0.000133207	Acidimicrobiia_IMCC26256_uncultured_bacterium	0	0	Latescibacterota_uncultured_Bacteroidetes	0.000137549	3.5515E-05
Deinococcus_xinjiangensis	0.000617602	0.000159464	Megasphaera_unidentified	0.000555494	0.000143428	Desulforhabdusunidentified_sulfate-reducing	0.00040923	0.000105663	Porticoccus_metagenome	0.000515907	0.000133207	Acidimicrobiia_IMCC26256_uncultured_soil	0	0	Obscuribacteraceaeuncultured_Gloeobacter	0.000136806	3.53232E-05
Lacunisphaera_uncultured_bacterium	0.000617602	0.000159464	ASV_5304	0.000554708	0.000143225	Johnsonella_Firmicutes_oral	0.000406308	9.79646E-05	Denitratisoma_sp.	0.000496192	0.000107322	Acidimicrobiia_IMCC26256uncultured_Aciditerrimonas	0	0	ASV_12573	0.000134277	3.46702E-05
Spirochaeta_sp.	0.000617602	0.000159464	ASV_7984	0.000554708	0.000143225	ASV_3021	0.000406036	8.69518E-05	Bdellovibrio_exovorus	0.00048676	0.000125681	Acidimicrobiia_IMCC26256uncultured_actinobacterium	0	0	Paracaedibacteraceae_uncultured_prokaryote	0.000134277	3.46702E-05
Hungateiclostridiaceae_uncultured__sp.	0.000614534	0.000158672	BSV13_sp.	0.000554708	0.000143225	Sphingomonas_panaciterrae	0.000402646	8.80517E-05	BIrii41_metagenome	0.00048676	0.000125681	Acidimicrobiia_IMCC26256uncultured_Actinomycetales	0	0	Nitrosomonadaceae_GOUTA6_metagenome	0.000133851	3.45602E-05
Bacteroidales_UCG-001_uncultured_prokaryote	0.000594177	0.000153416	Clostridia_uncultured__bacterium	0.000554708	0.000143225	Aerosphaera_sp.	0.000401016	0.000103542	Chlamydiaceae_uncultured_organism	0.00048676	0.000125681	Acidimicrobiia_sp.	0	0	Aliidongia_dinghuensis	0.000130098	3.35912E-05
Rhodoluna_uncultured_bacterium	0.000594177	0.000153416	Luteibacter_yeojuensis	0.000554708	0.000143225	Arachis_hypogaea	0.000401016	0.000103542	Phycisphaeraceae_SM1A02unidentified_marine	0.00048676	0.000125681	Acidimicrobiia_uncultured__bacterium	0	0	Candidatus_Peregrinibacteria_metagenome	0.000130098	3.35912E-05
Cellulomonadaceae_sp.	0.000592342	0.000152942	Sericytochromatia_sp.	0.000554708	0.000143225	ASV_9492	0.000401016	0.000103542	Pirellulaceae_uncultured_sludge	0.00048676	0.000125681	Acidimicrobiia_uncultured__metagenome	0	0	Defluviitaleaceae_UCG-011_sp.	0.000130098	3.35912E-05
ASV_16713	0.000587544	0.000151703	Candidatus_Moranbacteria__uncultured_Parcubacteria	0.000549481	0.000141875	Corynebacterium_matruchotii	0.000401016	0.000103542	Rubinisphaeraceae_uncultured__bacterium	0.00048676	0.000125681	Acidimicrobiia_uncultured__sp.	0	0	Gaiella_metagenome	0.000130098	3.35912E-05
Caedibacter_sp.	0.000587544	0.000151703	Chloroflexia_JG30-KF-CM67_metagenome	0.000549481	0.000141875	SphingorhabdusSphingomonas_sp.	0.000401016	0.000103542	uncultured_Thermovenabulum	0.00048676	0.000125681	Acidimicrobiia_uncultured_Acidimicrobiia	0	0	Rs-M47_sp.	0.000130098	3.35912E-05
Georgenia_sp.	0.000587544	0.000151703	Lawsonella_uncultured_bacterium	0.000523995	0.000103249	ASV_5832	0.000397377	0.000102602	ASV_16667	0.000482121	0.000124483	Acidimicrobiia_uncultured_actinobacterium	0	0	ASV_15451	0.000129421	3.34164E-05
Pyronema_omphalodes	0.000587544	0.000151703	ASV_19103	0.000523231	0.000135098	Chloroflexi_Gitt-GS-136_uncultured_bacterium	0.000390614	0.000100856	Bacteroides_plebeius	0.000482121	0.000124483	Acidimicrobiia_uncultured_Actinomycetales	0	0	Pirellulauncultured_Pasteuria	0.00012606	3.25486E-05
Latescibacterota_metagenome	0.000587429	0.000151674	Parcubacteria_UBA9983Candidatus_Nomurabacteria	0.000523231	0.000135098	Chloroplast_Mallomonas_splendens	0.000388687	0.000100359	Flavobacterium_lindanitolerans	0.000482121	0.000124483	Acidimicrobiia_uncultured_bacterium_YC-ZSS-LKJ199	0	0	ASV_3021	0.000122805	3.17081E-05
Breznakia_uncultured_bacterium	0.000584702	0.000150969	Sporichthya_sp.	0.000523231	0.000135098	Acetothermiia__uncultured_bacterium	0.000382149	9.86704E-05	Longivirga_uncultured_bacterium	0.000482121	0.000124483	Acidiphilium__uncultured_bacterium	0	0	Bacilli_RF39_human_gut	0.000122805	3.17081E-05
Megamonasuncultured_organism	0.000584702	0.000150969	ASV_15233	0.000519211	0.00013406	Lentimicrobiaceae_uncultured_Sphingobacteriales	0.000382149	9.86704E-05	Parvimonas_Peptostreptococcaceae_bacterium	0.000479364	0.000123771	Acidiphilium_sp.	0	0	Hydrogenophilus_sp.	0.000122805	3.17081E-05
Roseomonas_gilardii	0.000584702	0.000150969	Johnsonella_Firmicutes_oral	0.000519211	0.00013406	Verticiella_sp.	0.000382149	9.86704E-05	Klenkia_sp.	0.000465035	0.000120072	Acidisoma_sp.	0	0	Obscuribacteraceae_uncultured_bacterium	0.000122805	3.17081E-05
Mucilaginibacter_calamicampi	0.000580594	0.000149909	Methylophilaceae_uncultured__bacterium	0.000519211	0.00013406	Tsukamurella_paurometabola	0.000380683	7.12638E-05	Holophagaceae_metagenome	0.000463894	0.000119777	Acidithiobacillaceae_KCM-B-112_sp.	0	0	Pararhizobium_herbae	0.000122805	3.17081E-05
Caulobacter_metagenome	0.000579947	7.76645E-05	Patulibacter_sp.	0.000519211	0.00013406	Fimbriimonas_metagenome	0.000380385	9.8215E-05	Ruminococcus_gauvreauii_group_sp.	0.000447995	0.000115672	Acidithiobacillus_ferrooxidans	0	0	Parvimonas_sp.	0.000122805	3.17081E-05
Sphingobacteriales_S15A-MN91_uncultured_bacterium	0.000574006	0.000148208	Arcicella_uncultured_bacterium	0.000513347	0.000132546	ASV_6442	0.00037775	9.75346E-05	Haliangium_uncultured_prokaryote	0.000433711	0.000111984	Acidobacteria_bacterium	0	0	Singulisphaerauncultured_Planctomycetales	0.000122805	3.17081E-05
Anaerococcusuncultured_organism	0.000568057	0.000103626	Chloroflexia_JG30-KF-CM68_metagenome	0.000500612	0.000129257	uncultured_uncultured__metagenome	0.00037775	9.75346E-05	ASV_17967	0.000429923	0.000111006	Acidobacteriaceae_(Subgroup_1)_sp.	0	0	Paludibaculumuncultured_Acidobacteria	0.000121013	3.12454E-05

Chryseobacterium_uncultured_Bacteroidetes	0.000567848	0.000146618	Flavobacterium_arsenatis	0.000500612	0.000129257	Acidaminococcaceae_uncultured__bacterium	0.000375023	9.68305E-05	ASV_17973	0.000429923	0.000111006	Acidobacteriae_Elev-16S-1166_sp.	0	0	Pseudanabaena_PCC-7429_sp.	0.000121013	3.12454E-05
Afipia_sp.	0.000562799	0.000145314	Glutamicibacter_sp.	0.000500612	0.000129257	Actinoplanes_garbadinensis	0.000375023	9.68305E-05	ASV_17981	0.000429923	0.000111006	Acidobacteriae_Elev-16S-1166_uncultured_bacterium	0	0	Anaerococcusuncultured_organism	0.000119705	3.09077E-05
Cellulosimicrobium_sp.	0.000562799	0.000145314	Peptococcaceae_uncultured__sp.	0.000500612	0.000129257	ASV_2084	0.000375023	9.68305E-05	Caldilineaceae_uncultured__metagenome	0.000429923	0.000111006	Acidobacteriae_sp.	0	0	ASV_16127	0.000119705	3.09077E-05
Gemmataceae_uncultured__metagenome	0.000562799	0.000145314	Algoriphagus_uncultured_Bacteroidetes	0.000486449	0.000125601	ASV_14534	0.000374251	9.66312E-05	Deefgea_sp.	0.000429923	0.000111006	Acidocella__uncultured_bacterium	0	0	Giesbergeria_sp.	0.000119705	3.09077E-05
Hirschia_metagenome	0.000562799	0.000145314	ASV_16302	0.000486449	0.000125601	Defluviitaleaceae_UCG-011_sp.	0.000374251	9.66312E-05	Desulfobulbus_sp.	0.000429923	0.000111006	Acidothermus__uncultured_bacterium	0	0	Methylomonadaceae_pLW-20_sp.	0.000119705	3.09077E-05
Longimicrobium_uncultured_bacterium	0.000562799	0.000145314	Paeniglutamicibacter_sp.	0.000486449	0.000125601	Gemmata_metagenome	0.000374251	9.66312E-05	Kineosporiaceae_sp.	0.000429923	0.000111006	Acidothermus_sp.	0	0	Aliidiomarina_uncultured_bacterium	0.000113836	2.93923E-05
Sutterellaceae_uncultured__sp.	0.000562799	0.000145314	ASV_19178	0.000480538	0.000124074	Mycoplasmataceae_uncultured_Firmicutes	0.000374251	9.66312E-05	Opitutus_metagenome	0.000429923	0.000111006	Acidovorax_sp.	0	0	Rhodothermaceae_sp.	0.000113454	2.92937E-05
ASV_9885	0.000557849	0.000144036	Finegoldia_uncultured_bacterium	0.000480538	0.000124074	Rhodopirellula_metagenome	0.000374251	9.66312E-05	SB-5_uncultured_Cytophagales	0.000429923	0.000111006	Acinetobacter__metagenome	0	0	ASV_7974	0.000112945	2.91623E-05
ASV_9887	0.000557849	0.000144036	Methyloparacoccus_metagenome	0.000480538	0.000124074	Rickettsiales_SM2D12uncultured_Phyllobacteriaceae	0.000374251	9.66312E-05	Aerosphaera__uncultured_bacterium	0.000423363	0.000109312	Acinetobacter_baumannii	0	0	Bacteriovorax_uncultured_bacterium	0.000111542	2.88E-05
Butyrivibrio_Eubacterium_sp.	0.000550786	9.72477E-05	Microtrichales_metagenome	0.000480538	0.000124074	Afipia_sp.	0.000365823	9.44551E-05	Rickettsiaceae_uncultured__bacterium	0.000413266	0.000106705	Acinetobacter_kyonggiensis	0	0	Methylacidiphilaceae_uncultured_uncultured_Verrucomicrobiales	0.000111542	2.88E-05
ASV_7249	0.000539588	0.000139321	Pseudohongiella_metagenome	0.000480538	0.000124074	Hylemonella_sp.	0.000365823	9.44551E-05	Dysgonomonadaceae_sp.	0.000410662	0.000106032	Acinetobacter_sp.	0	0	oc32_sp.	0.000111542	2.88E-05
Aridibacter_sp.	0.000536193	0.000138444	Sericytochromatia_metagenome	0.000480538	0.000124074	ASV_11163	0.000362713	9.36521E-05	Anaerolineae_A4b__uncultured_soil	0.000406906	0.000105063	Acinetobacter_towneri	0	0	Sterolibacterium_uncultured_bacterium	0.000111542	2.88E-05
ASV_18963	0.000536193	0.000138444	Hymenobacter_ruber	0.000480231	0.000123995	ASV_11164	0.000362713	9.36521E-05	Moheibacteruncultured_compost	0.000406906	0.000105063	Acremonium_fuci	0	0	Mesorhizobium_albiziae	0.000110592	2.85547E-05
ASV_18967	0.000536193	0.000138444	ASV_19652	0.000479962	0.000123926	Bauldia_uncultured_bacterium	0.000362713	9.36521E-05	ASV_17893	0.000401768	0.000103736	Actinobacillus_sp.	0	0	Rhodospirillaceae_uncultured__metagenome	0.000110592	2.85547E-05
Peptococcus_sp.	0.000536193	0.000138444	ASV_2910	0.000479962	0.000123926	Bradymonadales_metagenome	0.00035089	9.05994E-05	Chloroplast_Synedra_hyperborea	0.000401768	0.000103736	Actinobacteria_0319-7L14_uncultured_actinobacterium	0	0	Verrucomicrobium_sp.	0.000109863	2.83665E-05
Rhodobacter_sp.	0.000536193	0.000138444	Burkholderiales_TRA3-20_sp.	0.000479962	0.000123926	WS2_uncultured_soil	0.000347308	8.96745E-05	Criblamydiaceae_sp.	0.000401768	0.000103736	Actinobacteria_PeM15_bacterium_rJ7	0	0	Hydrotalea_uncultured_bacterium	0.000104878	2.70794E-05
Prevotella_amnii	0.000531547	0.000137245	Caldilineaceae_sp.	0.000479962	0.000123926	Coriobacteriales_Incertae_Sedis_uncultured_organism	0.000342959	8.85516E-05	Kocuria_sp.	0.000401768	0.000103736	Actinobacteria_PeM15_metagenome	0	0	Rhizobiales_Incertae_Sedis_sp.	0.000103368	1.86846E-05
Propionibacterium_sp.	0.000531547	0.000137245	Uliginosibacterium_flavum	0.000479962	0.000123926	Prevotellaunidentified	0.000342959	8.85516E-05	Micromonosporaceae_sp.	0.000401768	0.000103736	Actinobacteria_PeM15_sp.	0	0	ASV_5084	0.000102849	2.65555E-05
ASV_2910	0.000527062	0.000105848	Actinobacteria_uncultured_Actinomycetales_bacterium	0.000479744	0.000123869	Chitinophagaceae_uncultured_Bacteroidetes	0.000339688	8.77071E-05	Sutterella_uncultured_bacterium	0.000401768	0.000103736	Actinobacteria_PeM15_uncultured_soil	0	0	Candidatus_Falkowbacteria_uncultured_bacterium	0.000102849	2.65555E-05
ASV_10460	0.000520659	0.000134434	Oxalobacter_uncultured_bacterium	0.000479744	0.000123869	Agitococcus_lubricus_group__uncultured_bacterium	0.000337521	8.71475E-05	Bacilli_RF39unidentified	0.00039988	0.000103249	Actinobacteria_PeM15uncultured_actinobacterium	0	0	Lactobacillus_delbrueckii	0.000102849	2.65555E-05
Pedosphaeraceae_ADurb.Bin063-1_uncultured_Verrucomicrobia	0.000520659	0.000134434	Fermentimonas_uncultured_prokaryote	0.000470568	0.0001215	Denitratisoma_uncultured_bacterium	0.000337521	8.71475E-05	Desulforhopalus_metagenome	0.00039988	0.000103249	Actinobacteria_sp.	0	0	Neochlamydia_endosymbiont_of	0.000102849	2.65555E-05
Chloroflexi_KD4-96_sp.	0.000520201	0.000134315	Clostridioides_difficile	0.000454309	0.000117302	Chryseomicrobium_sp.	0.000334812	8.64481E-05	ASV_15920	0.000389408	0.000100545	Actinobacteria_uncultured__bacterium	0	0	PelagibacteriumHyphomicrobiaceae_bacterium	0.000102849	2.65555E-05
Collimonas_sp.	0.000520201	0.000134315	dgA-11_gut_group_uncultured_bacterium	0.000454309	0.000117302	Adhaeribacter__metagenome	0.00033418	8.62849E-05	ASV_19624	0.000389408	0.000100545	Actinobacteria_uncultured__metagenome	0	0	Silvanigrellaceae_sp.	0.000102849	2.65555E-05
Clostridiaceae_sp.	0.000514668	0.000132887	Mycoplasma_sp.	0.000454309	0.000117302	Anaerovibrio_sp.	0.00033418	8.62849E-05	ASV_9544	0.000389408	0.000100545	Actinobacteria_uncultured__sp.	0	0	Aequorivita_sp.	0.000102604	2.64922E-05
Flavobacterium_anseonense	0.000514668	0.000132887	Patescibacteria_CPR2_uncultured_Firmicutes	0.000444988	0.000114895	ASV_17846	0.00033418	8.62849E-05	Chitinivorax_metagenome	0.000389408	0.000100545	Actinobacteria_uncultured_Actinomycetales_bacterium	0	0	Flavobacterium_kingsejongi	0.000102604	2.64922E-05
Paludibacteraceae_uncultured_Bacteroidetes	0.000514668	0.000132887	Cyanobacteriia_sp.	0.000444395	0.000114742	ASV_17854	0.00033418	8.62849E-05	Defluviicoccus_uncultured_bacterium	0.000389408	0.000100545	Actinobacteriota_MB-A2-108_metagenome	0	0	ASV_2386	0.000101441	2.30305E-05
Jatrophihabitans_metagenome	0.000507722	0.000131093	Ilumatobacter_metagenome	0.000444395	0.000114742	ASV_8228	0.00033418	8.62849E-05	Devosiaceae_sp.	0.000389408	0.000100545	Actinobacteriota_MB-A2-108_sp.	0	0	Ethanoligenens_uncultured_bacterium	9.83043E-05	2.53821E-05
Candidatus_Nomurabacteria_uncultured_soil	0.000492005	0.000127035	Actinobacteriota_MB-A2-108uncultured_actinobacterium	0.000439585	0.0001135	Babeliales_UBA12409uncultured_Candidatus	0.00033418	8.62849E-05	Lachnospiraceae_ND3007_group_metagenome	0.000389408	0.000100545	Actinobacteriota_MB-A2-108_uncultured_bacterium	0	0	Gaiella_actinobacterium_WWH12	9.83043E-05	2.53821E-05
Chitinivibrionaceae_possible_genus_03_sp.	0.000492005	0.000127035	Patescibacteria_CPR2_sp.	0.000439585	0.0001135	Competibacteraceae_sp.	0.00033418	8.62849E-05	Niveispirillum_sp.	0.000389408	0.000100545	Actinobacteriota_MB-A2-108uncultured_actinobacterium	0	0	ASV_3161	9.82439E-05	2.53665E-05
Oscillospiraceae_UCG-005_uncultured_bacterium	0.000492005	0.000127035	Armatimonadales_metagenome	0.000437509	0.000112964	Roseimaritima_sp.	0.00033418	8.62849E-05	Promicromonosporaceae_sp.	0.000389408	0.000100545	Actinomadura_sp.	0	0	Microscillaceae_uncultured__bacterium	9.82439E-05	2.53665E-05
Sphingomonas_glacialis	0.00049	0.00012	ASV_20515	0.00043	0.00011	Hydrocarboniphaga_uncultured_bacterium	0.00033	8.60693	Pseudomonas_japonica	0.00038	0.00010	Actinomyces__uncultured_bacterium	0	0	Pectobacterium_sp.	9.82439	2.53665

	2005	7035		7509	2964		3345	E-05		9408	0545					E-05	E-05
Alphaproteobacteria_uncultured_bacterium_enrichment	0.00048962	0.000126419	Flavobacteriales_NS9_marine_group_sp.	0.000437509	0.000112964	Deinococcus_reticulitermitis	0.000332836	8.59379E-05	Streptococcus_sobrinus	0.000389408	0.000100545	Actinomyces_graevenitzii	0	0	Planctomycetes_WD2101_soil_group_uncultured_bacterium	9.82439E-05	2.53665E-05
ASV_17928	0.00048962	0.000126419	Muribaculaceae_sp.	0.000437509	0.000112964	ASV_18301	0.000331148	8.5502E-05	Cloacimonadia_PBS-19_uncultured_bacterium	0.000386578	9.9814E-05	Actinomyces_sp.	0	0	RCP2-54uncultured_prokaryote	9.82439E-05	2.53665E-05
Candidatus_Saccharibacteria	0.00048962	0.000126419	Pseudoflavitalea_uncultured_bacterium	0.000437509	0.000112964	Pyrinomonadaceae_RB41_sp.	0.000331148	8.5502E-05	Lactobacillus_agilis	0.000386578	9.9814E-05	Actinomyces_unidentified	0	0	ASV_6298	9.76563E-05	2.52147E-05
Eubacterium_coprostanoligenes_group__uncultured_bacterium	0.00048962	0.000126419	Reyranellaceae_uncultured__bacterium	0.000437509	0.000112964	WS1_uncultured_bacterium	0.000331148	8.5502E-05	Vagococcus_sp.	0.000365523	9.4302E-05	Actinomycetaceae_uncultured__metagenome	0	0	BSV26_sp.	9.76563E-05	2.52147E-05
Inhella_sp.	0.00048962	0.000126419	ASV_6240	0.000430721	9.92667E-05	ASV_10613	0.00032747	8.45524E-05	Nannocystis_sp.	0.000348776	9.00536E-05	Actinomycetaceae_uncultured__rumen	0	0	Candidatus_Planktoluna_sp.	9.76563E-05	2.52147E-05
Lentimicrobiaceae_uncultured_Sphingobacteriales	0.00048962	0.000126419	ASV_13359	0.000427789	0.000110455	eub62A3_metagenome	0.00032747	8.45524E-05	ASV_2303	0.000343938	8.88044E-05	Actinomycetaceae_uncultured__sp.	0	0	ASV_7249	9.75737E-05	2.51934E-05
Simplicispira_sp.	0.00048962	0.000126419	Phreatobacter_uncultured_Brucella	0.000427789	0.000110455	Lactobacillus_senioris	0.00032747	8.45524E-05	ASV_5802	0.000343938	8.88044E-05	Actinomycetospora_sp.	0	0	Candidatus_Kaiserbacteriauncultured_organism	9.75737E-05	2.51934E-05
Triticum_aestivum	0.00048962	0.000126419	Psychrobacter_immobilis	0.000427789	0.000110455	Rs-K70_termite_group_uncultured_bacterium	0.00032747	8.45524E-05	Blastocatellaceae_sp.	0.000343938	8.88044E-05	Actinoplanes_garbadinensis	0	0	Z4MB62_Z4MB62uncultured_Bacteroidetes	9.75737E-05	2.51934E-05
Tropicimonas_sp.	0.00048962	0.000126419	Clostridium_sensu_stricto_9_uncultured_bacterium	0.000425811	0.000109944	Rhodocyclaceae_uncultured_Georgfuchsia	0.000320095	8.26482E-05	Candidatus_Omnitrophusuncultured_Firmicutes	0.000343938	8.88044E-05	Actinoplanes_sp.	0	0	ASV_11732	9.68101E-05	2.49963E-05
ASV_14864	0.000475342	0.000122733	Kazania_sp.	0.000418585	0.000108078	ASV_6176	0.000314892	6.92424E-05	Crocinitomicaceae_sp.	0.000343938	8.88044E-05	Actinotalea_sp.	0	0	ASV_513	9.68101E-05	2.49963E-05
Bacteroides_uncultured_Bacteroidaceae	0.000475342	0.000122733	Schlesneria_uncultured_bacterium	0.000418585	0.000108078	Bacteroidales_F082_uncultured_bacterium	0.000314231	8.11341E-05	Hyphomonas_sp.	0.000343938	8.88044E-05	Actinotignum_sp.	0	0	Chlamydiaceae_metagenome	0.000095226	2.45872E-05
ASV_6446	0.000469943	0.000121339	JG30-KF-CM45_sp.	0.000416956	0.000107658	ASV_7465	0.000301794	7.79229E-05	LD1-PB3_metagenome	0.000343938	8.88044E-05	Adhaeribacter__metagenome	0	0	Bacteroidia_uncultured__sp.	8.92339E-05	2.30401E-05
ASV_18506	0.000468999	0.000121095	Hymenobacter_sp.	0.000416031	0.000107419	ASV_4909	0.000299731	7.22949E-05	Moraxellaceae_uncultured_gamma	0.000343938	8.88044E-05	Adhaeribacter__uncultured_soil	0	0	Candidatus_Giovannonibacteria_uncultured_bacterium	8.92339E-05	2.30401E-05
ASV_2889	0.000468999	0.000121095	Hyphomicrobium_sp.	0.000416031	0.000107419	Ruminococcus_gauvreauii_group__uncultured_rumen	0.000297231	7.67447E-05	Neisseriaceae_uncultured_organism	0.000343938	8.88044E-05	Adhaeribacter_sp.	0	0	Flavobacterium_uncultured_bacterium	8.92339E-05	2.30401E-05
ASV_6298	0.000468999	0.000121095	Lachnospiraceae_sp.	0.000416031	0.000107419	Corynebacterium_amycolatum	0.000295873	6.88947E-05	Parvimonas_sp.	0.000343938	8.88044E-05	Advenella_sp.	0	0	Hypnocyclicus_uncultured_Fusobacteriales	8.92339E-05	2.30401E-05
Lactococcus_sp.	0.000468999	0.000121095	Rufibacter_uncultured_bacterium	0.000416031	0.000107419	Kapabacterialesuncultured_Bacteroidetes/Chlorobi	0.000291168	5.93752E-05	Planctomycetes_uncultured__bacterium	0.000343938	8.88044E-05	Aenigmarchaeales_sp.	0	0	Parabacteroides_distasonis	8.92339E-05	2.30401E-05
Luteolibacter_uncultured_Verrucomicrobiales	0.000468999	0.000121095	Sandaracinaceae_uncultured__metagenome	0.000416031	0.000107419	ASV_14494	0.00028823	5.50066E-05	Sterolibacterium_metagenome	0.000343938	8.88044E-05	Aenigmarcheota_sp.	0	0	Planctomycetes_Pla3_lineage_uncultured_bacterium	8.92339E-05	2.30401E-05
Roseburia_uncultured_rumen	0.000468999	0.000121095	Candidatus_Endonucleariobacter_sp.	0.000390327	0.000100782	ASV_5871	0.000286584	5.82279E-05	ASV_5322	0.000335996	8.67538E-05	Aequorivita_sp.	0	0	Plantibacter_sp.	8.92339E-05	2.30401E-05
Ammoniibacillus_agariperforans	0.000467643	0.000120745	ASV_17550	0.000389408	0.000100545	Pelagicoccusuncultured_Verrucomicrobia	0.000281154	7.25937E-05	Cyanobium_PCC-6307_sp.	0.000335996	8.67538E-05	Aerococcaceae_sp.	0	0	Solitalea_sp.	8.92339E-05	2.30401E-05
ASV_11030	0.000467643	0.000120745	ASV_17552	0.000389408	0.000100545	Hymenobacter_metagenome	0.000280689	7.24736E-05	Leeia_uncultured_bacterium	0.000335996	8.67538E-05	Aerococcaceae_uncultured__bacterium	0	0	ASV_2465	8.78433E-05	1.95584E-05
Blrii41_uncultured_Sorangiineae	0.000465411	0.000120169	Chloroplast_Chroomonas_coerulea	0.000389408	0.000100545	Pseudarcobacter_uncultured_bacterium	0.000280689	7.24736E-05	ASV_3560	0.00033284	6.4999E-05	Aerococcus_sp.	0	0	Capnocytophaga_ochracea	8.60162E-05	2.22093E-05
Paenalcaligenes_sp.	0.000465411	0.000120169	Prevotellaceae_UCG-001_uncultured_bacterium	0.000389365	0.000100534	Singulisphaera_sp.	0.000280689	7.24736E-05	Candidatus_Nitrosotenuis_sp.	0.000330403	8.53097E-05	Aeromicrobium_sp.	0	0	Candidatus_Accumulibacter_sp.	8.55037E-05	2.2077E-05
Quadrisphaera_sp.	0.000465411	0.000120169	Candidatus_Limnoluna_uncultured_bacterium	0.000384637	9.93128E-05	Stenotrophomonas_rhizophila	0.000280689	7.24736E-05	Conexibacter_sp.	0.000330403	8.53097E-05	Aeromonas__metagenome	0	0	Chloroplast_Tetraselmis_cordiformis	8.55037E-05	2.2077E-05
Anaerococcus_sp.	0.0004609	0.000119004	CL500-29_marine_group_bacterium_enrichment	0.000384637	9.93128E-05	Oscillospirales_UCG-010gut__metagenome	0.000274367	7.08413E-05	Antarctic_bacterium	0.000321414	8.29887E-05	Aeromonas_sp.	0	0	Leeia_sp.	8.55037E-05	2.2077E-05
ASV_21429	0.0004609	0.000119004	Rickettsiaceae_uncultured__sp.	0.000384637	9.93128E-05	Oxalobacteraceae_CM1G08_uncultured_bacterium	0.000274367	7.08413E-05	ASV_6422	0.000321414	8.29887E-05	Aeromonas_uncultured_gamma	0	0	Sutterellaceae_uncultured__bacterium	8.55037E-05	2.2077E-05
Candidatus_Staskawiczbacteria_uncultured_soil	0.0004609	0.000119004	Prevotella_nigrescens	0.000373134	9.63428E-05	Adhaeribacter_sp.	0.000267344	6.90279E-05	Candidatus_Uhrbacteria_uncultured_bacterium	0.000321414	8.29887E-05	Aerosphaera__uncultured_bacterium	0	0	ASV_16447	8.54492E-05	2.20629E-05
Lachnoclostridium_sp.	0.0004609	0.000119004	ASV_20445	0.000360404	9.30559E-05	Brevinema_uncultured_bacterium	0.000267344	6.90279E-05	Flavobacterium_unidentified	0.000321414	8.29887E-05	Aerosphaera_sp.	0	0	ASV_15253	8.46453E-05	2.18553E-05
Mitochondria_uncultured_bacterium	0.0004609	0.000119004	Blfdi19_uncultured_delta	0.000360404	9.30559E-05	Candidatus_Ryanbacteria	0.000267344	6.90279E-05	Lacunisphaera_metagenome	0.000321414	8.29887E-05	Aestuariicella__uncultured_bacterium	0	0	Cyanobacteria_sp.	8.46453E-05	2.18553E-05
Pirellulaceae_sp.	0.0004609	0.000119004	Candidatus_Curtissbacteria_uncultured_bacterium	0.000360404	9.30559E-05	Clostridia_UCG-014_uncultured_bacterium	0.000267344	6.90279E-05	Polymorphobacter_sp.	0.000321414	8.29887E-05	Afipia_genosp.	0	0	Hyphomicrobiaceae_sp.	8.13114E-05	2.09945E-05
Uruburuella_sp.	0.000450362	0.000116283	Clostridia_UCG-014_sp.	0.000360404	9.30559E-05	Flavobacterium_lindanitolerans	0.000267344	6.90279E-05	Spirochaeta_2_sp.	0.000321414	8.29887E-05	Afipia_sp.	0	0	ASV_13313	8.06751E-05	2.08302E-05

Elizabethkingia_sp.	0.000446279	0.000115229	Conexibacter_metagenome	0.000360404	9.30559E-05	Hungateiclostridiaceae_uncultured__bacterium	0.000267344	6.90279E-05	Terrimicrobium_sp.	0.000321414	8.29887E-05	Agathobacter_sp.	0	0	DEV007_uncultured_Verrucomicrobiales	8.06751E-05	2.08302E-05
Blfdi19_sp.	0.000435445	0.000112431	Oscillospiraceae_UCG-005_uncultured_Ruminococcaceae	0.000360404	9.30559E-05	Hyphomonadaceae_SWB02_metagenome	0.000267344	6.90279E-05	Lacunisphaerauncultured_Opitutus	0.000317033	8.18576E-05	Aggregatibacter_actinomycetemcomitans	0	0	Legionella_geestiana	7.56363E-05	1.95292E-05
Fastidiosipila_uncultured_bacterium	0.000435445	0.000112431	Paludibacteraceae_uncultured_Paludibacter	0.000360404	9.30559E-05	Kiritimatiellae_WCHB1-41_uncultured_bacterium	0.000267344	6.90279E-05	Rhizorhapis_sp.	0.000317033	8.18576E-05	Aggregatibacter_sp.	0	0	Rhodopirellula_planctomycete_str.	7.40647E-05	1.91234E-05
ASV_11696	0.00043167	0.000111457	Runellauncultured_Runella	0.000360404	9.30559E-05	Lachnospiraceae_uncultured_Roseburia	0.000267344	6.90279E-05	Prevotellaunidentified	0.000313773	7.52162E-05	Agitococcus_lubricus_group__uncultured_bacterium	0	0	Spirosoma_escalantus	7.40647E-05	1.91234E-05
ASV_6965	0.000430781	0.000111227	Chitinophagales_37-13_uncultured_Saprospiraceae	0.000359971	9.29441E-05	Meiothermus_uncultured_bacterium	0.000267344	6.90279E-05	Mucilaginibacter_galii	0.000312047	8.05702E-05	Agitococcus_lubricus_group_sp.	0	0	Acidibacter_sp.	7.37282E-05	1.90365E-05
ASV_20235	0.000428954	0.000110755	Elusimicrobiota_Lineage_IIb_uncultured_bacterium	0.000359971	9.29441E-05	Pseudarcobacter_uncultured_Arcobacter	0.000267344	6.90279E-05	ASV_21444	0.000309949	8.00285E-05	Agrococcus_sp.	0	0	Actinomycetaceae_uncultured__metagenome	7.37282E-05	1.90365E-05
Bacteroides_uncultured_Bacteroides	0.000428954	0.000110755	Eggerthellaceae_DMER64_bacterium_enrichment	0.000342231	8.83637E-05	ASV_19606	0.000264918	6.84015E-05	ASV_2160	0.000309949	8.00285E-05	Agromyces_sp.	0	0	ASV_10605	7.37282E-05	1.90365E-05
Raineya_orbicola	0.000428954	0.000110755	Pseudomonadaceae_sp.	0.000342231	8.83637E-05	Chitinophagales_37-13__uncultured_bacterium	0.000264918	6.84015E-05	ASV_7664	0.000309949	8.00285E-05	Ahniella_affigens	0	0	Candidatus_Alysiosphaera_uncultured_bacterium	7.37282E-05	1.90365E-05
Rhodocyclaceae_sp.	0.000428954	0.000110755	ASV_2285	0.000337101	8.70391E-05	Erysipelotrichaceae_sp.	0.000264918	6.84015E-05	Mycobacterium_sp.	0.000309949	8.00285E-05	Ahniella_metagenome	0	0	Legionella_metagenome	7.37282E-05	1.90365E-05
Solirubrobacterales_67-14_uncultured_Rubrobacteraceae	0.000428954	0.000110755	Bacillus_sp.	0.000333741	8.61716E-05	Isosphaeraceae_uncultured__metagenome	0.000264918	6.84015E-05	Rhodoluna_sp.	0.000309949	8.00285E-05	Ahniella_uncultured_bacterium	0	0	Treponema_denticola	7.37282E-05	1.90365E-05
Sporichthya_uncultured_bacterium	0.000428954	0.000110755	Sphaerochaeta_globosa	0.000333741	8.61716E-05	Methyloligellaceae_uncultured_bacterium	0.000264918	6.84015E-05	Selenomonas_lacticifex	0.000309949	8.00285E-05	Akkermansia_sp.	0	0	ASV_20495	7.36829E-05	1.90248E-05
Sporomusaceae_uncultured__sp.	0.000428954	0.000110755	Methanosaeta_sp.	0.000329688	8.51251E-05	Burkholderia-Caballeronia-Paraburkholderia_sp.	0.000262516	6.77813E-05	Chlamydiae_sp.	0.000309262	7.98511E-05	Akkermansia_uncultured_bacterium	0	0	ASV_16300	7.19048E-05	1.85657E-05
Dehalococcoidia_S085_sp.	0.000425238	0.000109796	Microscillaceae_OLB12_uncultured_bacterium	0.000329688	8.51251E-05	Methanoregula_sp.	0.000262516	6.77813E-05	Thermincolauncultured_Clostridiales	0.000309262	7.98511E-05	Alcaligenaceae_sp.	0	0	Acidimicrobiia_IMCC26256_uncultured_soil	0.000068403	1.76616E-05
Sulfurospirillum_uncultured_soil	0.000425238	0.000109796	Ruminococcaceae_uncultured__sp.	0.000329688	8.51251E-05	Eggerthellaceae_DNF00809_uncultured_bacterium	0.00025923	6.69329E-05	ASV_15330	0.000298663	7.71145E-05	Alcaligenes_sp.	0	0	ASV_19866	0.000068403	1.76616E-05
Flavobacterium_lindanitolerans	0.000424019	8.12347E-05	Turicella_uncultured_bacterium	0.000329688	8.51251E-05	Caulobacter_metagenome	0.000254897	3.87407E-05	Firmicutes_D8A-2_uncultured_bacterium	0.000296749	7.66203E-05	Algibacter_sp.	0	0	ASV_19872	0.000068403	1.76616E-05
Candidatus_Dichloromethanomonas_uncultured_bacterium	0.000423101	0.000109244	ASV_18786	0.000324507	8.37873E-05	ASV_17511	0.000254766	6.57803E-05	Actinomadura_sp.	0.000292056	7.54085E-05	Algoriphagus_sp.	0	0	Myxococcota_sp.	0.000068403	1.76616E-05
Actinobacteriota_MB-A2-108_sp.	0.000415924	0.000107391	Candidatus_Megaira_sp.	0.000324507	8.37873E-05	ASV_17516	0.000254766	6.57803E-05	ASV_8275	0.000292056	7.54085E-05	Algoriphagus_uncultured_Bacteroidetes	0	0	Roseomonas_sp.	0.000068403	1.76616E-05
Alishewanella_uncultured_bacterium	0.000415924	0.000107391	Chloroplast_Tetradesmus_obliquus	0.000324507	8.37873E-05	Chloroplast_Guillardia_theta	0.000254766	6.57803E-05	Candidatus_Planktophila_sp.	0.000292056	7.54085E-05	Algoriphagus_uncultured_Hongiella	0	0	ASV_2061	6.69254E-05	1.72801E-05
Ilumatobacteraceae_uncultured__sp.	0.000415924	0.000107391	Janthinobacterium_sp.	0.000324507	8.37873E-05	Halieaceae_OM60(NOR5)_clade_metagenome	0.000254766	6.57803E-05	Firmicutes_uncultured__bacterium	0.000292056	7.54085E-05	Aliidiomarina_uncultured_bacterium	0	0	ASV_20943	6.69254E-05	1.72801E-05
ASV_3349	0.00041581	7.5013E-05	Salinispira_metagenome	0.000324507	8.37873E-05	Ruminococcaceae_uncultured_anaerobic_digester	0.000254766	6.57803E-05	Gracilibacteria_JGI_0000069-P22Candidatus_Gracilibacteria	0.000292056	7.54085E-05	Aliidongia_dinghuensis	0	0	Polynucleobacter_sp.	6.69254E-05	1.72801E-05
ASV_2160	0.000412728	8.41585E-05	Candidatus_Vogelbacteria_uncultured_bacterium	0.000320154	8.26634E-05	ASV_4167	0.000252749	6.52595E-05	Myxococcaceae_P3OB-42_sp.	0.000292056	7.54085E-05	Aliivibrio_sp.	0	0	Acidiphilium_sp.	6.50491E-05	1.67956E-05
ASV_5218	0.000411734	0.000106309	Haliangium_metagenome	0.000320154	8.26634E-05	LaribacterLaribacter_hongkongensis	0.000251834	6.50233E-05	Prevotellaunidentified_eubacterium	0.000292056	7.54085E-05	Alishewanella_sp.	0	0	Anaerolineae_A4b__metagenome	6.50491E-05	1.67956E-05
CCM19a_uncultured_gamma	0.000411734	0.000106309	Micrarchaeales_CG1-02-32-21_uncultured_archaeon	0.000320154	8.26634E-05	ASV_12420	0.000251503	6.49378E-05	Syntrophobacteruncultured_Syntrophobacter	0.000292056	7.54085E-05	Alishewanella_uncultured_bacterium	0	0	Clostridia_UCG-014_uncultured_Firmicutes	6.50491E-05	1.67956E-05
Diplorickettsiaceae_uncultured_Coxiellaceae	0.000411734	0.000106309	Rubrobacter_sp.	0.000320154	8.26634E-05	Filifactor_sp.	0.000251503	6.49378E-05	Vicinamibacteraceae_sp.	0.000292056	7.54085E-05	Alistipes_gut__metagenome	0	0	Coprococcus_sp.	6.50491E-05	1.67956E-05
Isosphaeraceae_uncultured_Planctomycetaceae	0.000411734	0.000106309	Akkermansia_sp.	0.000313939	8.10587E-05	Johnsonella_ignava	0.000251503	6.49378E-05	Algoriphagus_sp.	0.000290647	7.50447E-05	Alistipes_sp.	0	0	Desulfobacterium_catecholicum_group_uncultured_bacterium	6.50491E-05	1.67956E-05
Rubinisphaeraceae_uncultured__metagenome	0.000411734	0.000106309	ASV_9544	0.000313939	8.10587E-05	Nevskia_uncultured_Nevskia	0.000251109	6.48361E-05	Desulfosporosinus_sp.	0.000290647	7.50447E-05	Alistipes_uncultured_Alistipes	0	0	Flavobacterium_lindanitolerans	6.50491E-05	1.67956E-05
Thermoactinomycetaceae_uncultured__bacterium	0.00041012	0.000106171	Candidatus_Collierbacteria_uncultured_soil	0.000313939	8.10587E-05	Xanthomonadaceae_Xanthomonas_sp.	0.000251109	6.48361E-05	Dyadobacter_sp.	0.000290647	7.50447E-05	Alistipes_uncultured_bacterium	0	0	Methanospirillum_uncultured_archaeon	6.50491E-05	1.67956E-05
Candidatus_Methylopumilus_sp.	0.000410004	0.000105863	Cytophaga_metagenome	0.000313939	8.10587E-05	ASV_2510	0.000244611	4.95203E-05	Eggerthia_catenaformis	0.000290647	7.50447E-05	Alistipes_uncultured_Bacteroidetes	0	0	Nitrospira_sp.	6.50491E-05	1.67956E-05
Chitinophaga_sp.	0.000410004	0.000105863	Fibrella_sp.	0.000313939	8.10587E-05	Saprospiraceae_uncultured_Cytophaga	0.000244309	6.30803E-05	Flavisolibacter_sp.	0.000290647	7.50447E-05	Aliterella_cyanobacterium_OU_20	0	0	Pedosphaeraceae_uncultured__bacterium	6.50491E-05	1.67956E-05
Cloacibacterium_sp.	0.000410004	0.000105863	Nitrosomonadaceae_GOUTA6_uncultured_beta	0.000313939	8.10587E-05	Campylobacter_curvus	0.000243301	6.282E-05	Clavibacter_sp.	0.000275335	7.10912E-05	Aliterella_sp.	0	0	Proteocatella_uncultured_bacterium	6.50491E-05	1.67956E-05

Dongia_sp.	0.000410004	0.000105863	Planctopirus_uncultured_bacterium	0.000313939	8.10587E-05	Candidatus_Saccharimonas_sp.	0.000233907	6.03945E-05	Prevotella_marshii	0.000273881	7.07158E-05	Aliterella_uncultured_bacterium	0	0	Elusimicrobiota_Lineage_IV_uncultured_Termite	6.47103E-05	1.67081E-05
Methylotenerauncultured_Methylotenera	0.000409089	0.000105626	ASV_6176	0.000305317	5.84896E-05	CL500-29_marine_group_sp.	0.000233907	6.03945E-05	Burkholderiales_sp.	0.000263911	5.03606E-05	Alkalibacter_uncultured_bacterium	0	0	Parachlamydiaceae_uncultured__metagenome	6.45401E-05	1.66642E-05
Sphingobacteriales_env.OPS_17_uncultured_Sphingobacteriia	0.000398666	0.000102935	ASV_16374	0.000299452	7.73182E-05	Parabacteroides_sp.	0.000233907	6.03945E-05	Lachnospiraceae_uncultured_Lachnospiraceae_bacterium	0.000262598	6.78025E-05	Alkalibacterium_uncultured_bacterium	0	0	Desulfatiglans_sp.	0.0000063484	1.63915E-05
Daphnia_magna	0.000395698	0.000102169	Aquicella_metagenome	0.000278118	7.18098E-05	Ruminiclostridium_metagenome	0.000233907	6.03945E-05	ASV_10385	0.000261331	6.74754E-05	Alkanibacter_metagenome	0	0	Microscillaceae_uncultured__metagenome	0.0000063484	1.63915E-05
Kineococcus_sp.	0.000395698	0.000102169	Facklamia_sp.	0.000278118	7.18098E-05	Tychonema_CCAP_1459-11B_sp.	0.000233907	6.03945E-05	Soortia_sp.	0.000261331	6.74754E-05	Alkanindiges_sp.	0	0	ASV_18695	6.30302E-05	1.62743E-05
Asticcacaulis_sp.	0.000391696	0.000101135	ASV_20042	0.000277971	7.17718E-05	Elstera_uncultured_bacterium	0.000229938	5.93697E-05	Altererythrobacter_alpha_proteobacterium	0.000257954	6.66034E-05	Alkanindiges_uncultured_bacterium	0	0	Solirubrobacteraceae_sp.	6.30302E-05	1.62743E-05
ASV_19207	0.000391696	0.000101135	Leeia_uncultured_bacterium	0.000277971	7.17718E-05	Bacteroides_pyogenes	0.000228639	5.90343E-05	Candidatus_Alysiosphaera_metagenome	0.000257954	6.66034E-05	Alkanindiges_uncultured_gamma	0	0	ASV_346	6.14402E-05	1.58638E-05
ASV_19219	0.000391696	0.000101135	T34_uncultured_Burkholderiales	0.000277971	7.17718E-05	Flavobacterium_weaverense	0.000228639	5.90343E-05	Endomicrobium_uncultured_bacterium	0.000257954	6.66034E-05	Allobranchiibius_sp.	0	0	Shimazuella_uncultured_bacterium	6.14402E-05	1.58638E-05
Candidatus_Roizmanbacteria_uncultured_bacterium	0.000391696	0.000101135	Comamonadaceae_sp.	0.000277354	7.16125E-05	Planktothrix_NIVA-CYA_15_sp.	0.000228639	5.90343E-05	Flavobacterium_Bacteroidetes_bacterium	0.000257954	6.66034E-05	Alloiococcus_uncultured_bacterium	0	0	Agrococcus_sp.	6.10352E-05	1.57592E-05
Chlamydiaceae_uncultured__bacterium	0.000391696	0.000101135	Solirubrobacterales_67-14_uncultured_actinobacterium	0.000277354	7.16125E-05	Oribacterium_uncultured_organism	0.00022823	5.89287E-05	Patescibacteria_WWE3bacterium_enrichment	0.000257954	6.66034E-05	Alloprevotella_sp.	0	0	ASV_18923	6.10352E-05	1.57592E-05
Clavibacter_sp.	0.000391696	0.000101135	Pedosphaeraceae_SH3-11_metagenome	0.00027474	7.09376E-05	Leeia_uncultured_bacterium	0.000225014	5.80984E-05	Sphingobacterium_sp.	0.000257954	6.66034E-05	Alloprevotella_tannerae	0	0	ASV_18928	6.10352E-05	1.57592E-05
Gaiellales_uncultured_prokaryote	0.000391696	0.000101135	Chlamydiales_sp.	0.000274267	7.08154E-05	Proteiniclasticum_sp.	0.000225014	5.80984E-05	Verrucomicrobiaceae_uncultured_uncultured_verrucomicrobium	0.000257954	6.66034E-05	Alloprevotella_uncultured_Bacteroidales	0	0	Fermentimonas_sp.	6.10352E-05	1.57592E-05
Haliangium_uncultured_Myxococcales	0.000391696	0.000101135	Oligoflexus_uncultured_delta	0.000271162	4.85848E-05	Acanthopleuribacter__uncultured_bacterium	0.000220354	5.68952E-05	Aneurinibacillus_uncultured_bacterium	0.00025698	6.6352E-05	Allorhizobium-Neorhizobium-Pararhizobium-Rhizobium_sp.	0	0	Metamycoplasma_salivarium	6.10352E-05	1.57592E-05
Ilumatobacteraceae_uncultured__metagenome	0.000391696	0.000101135	ASV_5961	0.000266525	6.88165E-05	ASV_16250	0.000220354	5.68952E-05	ASV_10098	0.00025698	6.6352E-05	allotenuales_AKIW781_uncultured_bacterium	0	0	Micrococcus_sp.	6.10352E-05	1.57592E-05
Isosphaeraceae_uncultured__uncultured_soil	0.000391696	0.000101135	Gammaproteobacteria_CHAB-XI-31_uncultured_bacterium	0.000259605	6.70297E-05	Bacteroides_coprophilus	0.000220354	5.68952E-05	Macromonas_sp.	0.00025698	6.6352E-05	alpha_proteobacterium	0	0	Planctomycetes_Pla4_lineage_sp.	6.10352E-05	1.57592E-05
Pseudonocardia_sp.	0.000391696	0.000101135	Gemmatimonadaceae_uncultured__uncultured_soil	0.000259605	6.70297E-05	Zoogloea_sp.	0.000220354	5.68952E-05	Candidatus_Campbellbacteria_sp.	0.000254018	6.55872E-05	Alphaproteobacteria_bacterium	0	0	ASV_305	5.80254E-05	1.06312E-05
Sphingobacteriales_AKYH767_uncultured_bacterium	0.000391696	0.000101135	Pedosphaeraceae_SH3-11uncultured_Verrucomicrobia	0.000259605	6.70297E-05	Treponema_genomosp.	0.000220346	5.68931E-05	Dehalococcoidia_RBG-13-46-9_uncultured_bacterium	0.000251096	6.48327E-05	Alphaproteobacteria_sp.	0	0	Pedosphaeraceae_SH3-11_sp.	5.64726E-05	1.45812E-05
Spirochaetota_MVP-15_uncultured_bacterium	0.000391696	0.000101135	Dechloromonas_sp.	0.000256674	6.62729E-05	ASV_2862	0.000217876	3.60111E-05	Elusimicrobia_MVP-88_uncultured_bacterium	0.000251096	6.48327E-05	alphaproteobacteria_uncultured__bacterium	0	0	ASV_1208	5.29033E-05	1.36596E-05
Sumerlaea_metagenome	0.000391696	0.000101135	Schlesneria_sp.	0.000256674	6.62729E-05	ASV_6194	0.000215	5.55128E-05	Streptomycetaceae_sp.	0.000251096	6.48327E-05	Alphaproteobacteria_uncultured__bacterium	0	0	ASV_2850	5.29033E-05	1.36596E-05
Arsenicicoccus_bolidensis	0.000375199	9.6876E-05	Campylobacter_concisus	0.00024839	6.4134E-05	Lautropia_sp.	0.000212305	5.48169E-05	ASV_20431	0.000241061	6.22417E-05	Alphaproteobacteria_uncultured__metagenome	0	0	Lactobacillales_P5D1-392_uncultured_bacterium	5.28044E-05	1.3634E-05
ASV_19823	0.000375199	9.6876E-05	Chryseobacterium_uncultured_Bacteroidetes	0.00024839	6.4134E-05	Xanthomonadaceae_uncultured__bacterium	0.000210534	5.43596E-05	Christensenellaceae_R-7_group_uncultured_compost	0.000241061	6.22417E-05	Alphaproteobacteria_uncultured__sp.	0	0	Candidatus_Colwellbacteria	5.13022E-05	1.32462E-05
Candidatus_Competibacter_sp.	0.000375199	9.6876E-05	Anaerovoracaceae_Family_XIII_AD3011_group_sp.	0.000240269	6.20372E-05	Clostridium_vincentii	0.000201196	5.19486E-05	Corynebacterium_propinquum	0.000241061	6.22417E-05	Alphaproteobacteria_uncultured_Alphaproteobacteria	0	0	Candidatus_Giovannonibacteria_sp.	5.13022E-05	1.32462E-05
Candidatus_Planktophila_uncultured_bacterium	0.000375199	9.6876E-05	Corynebacterium_amycolatum	0.000240269	6.20372E-05	Opitutus_uncultured_bacterium	0.000201196	5.19486E-05	Simkaniaceae_sp.	0.000241061	6.22417E-05	Alphaproteobacteria_uncultured_bacterium_enrichment	0	0	Gastranaerophilales_uncultured_bacterium	5.13022E-05	1.32462E-05
Chloroplast_Cinnamomum_camphora	0.000375199	9.6876E-05	Lachnospiraceae_uncultured_Clostridiaceae_bacterium	0.000240269	6.20372E-05	ASV_3630	0.000200668	3.99862E-05	Ralstonia_sp.	0.000237774	6.1393E-05	Alphaproteobacteria_uncultured_eubacterium	0	0	Gemmatimonas_sp.	5.13022E-05	1.32462E-05
Pajaroellobacter_sp.	0.000375199	9.6876E-05	Alphaproteobacteria_uncultured__sp.	0.000239981	6.19628E-05	Anaerolineaceae_uncultured_delta	0.000200508	5.17709E-05	ASV_10316	0.000237083	6.12146E-05	Alphaproteobacteria_uncultured_gut__metagenome	0	0	Porphyromonas_uncultured_bacterium	5.13022E-05	1.32462E-05
Aerococcus_sp.	0.000372083	9.60714E-05	ASV_5084	0.000239981	6.19628E-05	Bdellovibrionaceae_OM27_clade_sp.	0.000200508	5.17709E-05	Alphaproteobacteria_bacterium	0.000232518	6.00359E-05	Alphaproteobacteria_uncultured_Lacibacterium_aquatile	0	0	Sphingobacteriales_NS11-12_marine_groupuncultured_Cytophagales	5.13022E-05	1.32462E-05
Pseudomonas_graminis	0.000372083	9.60714E-05	Chloroplast_Oscillatoriales_cyanobacterium	0.000239981	6.19628E-05	Filimonas_lacunae	0.000200508	5.17709E-05	ASV_4527	0.000232518	6.00359E-05	Alphaproteobacteria_uncultured_Novosphingobium	0	0	Caulobacter_metagenome	5.04242E-05	1.30195E-05
Neisseriaceae_uncultured_organism	0.000372038	9.60598E-05	Margulisbacteria_sp.	0.000239981	6.19628E-05	Isosphaeraceae_sp.	0.000200508	5.17709E-05	Sandaracinaceae_uncultured_Oryza_longistaminata	0.000231947	5.98885E-05	Alphaproteobacteria_uncultured_Rhodospirillaceae_bacterium	0	0	Corynebacterium_aurimucosum	5.04242E-05	1.30195E-05
ASV_2433	0.000371899	9.60239E-05	Burkholderiales_SC-I-84_sp.	0.000219792	5.67501E-05	Jatrophihabitans_sp.	0.000200508	5.17709E-05	TaibaiellaTaibaiella_sp.	0.000231947	5.98885E-05	Altererythrobacter_alpha_proteobacterium	0	0	Corynebacterium_propinquum	5.04242E-05	1.30195E-05
Ignatzschineria_swine_effluent	0.000362871	9.36929E-05	Oscillospiraceae_UCG-005_sp.	0.000219792	5.67501E-05	Kocuria_marina	0.000200508	5.17709E-05	Clostridia_vadinBB60_group_Clostridiales_bacterium	0.000229881	4.5829E-05	Altererythrobacter_sp.	0	0	Phaselicystis_uncultured_Polyangiaceae	5.04242E-05	1.30195E-05

Diplorickettsiaceae_uncultured_bacterium_enrichment	0.000362424	9.35775E-05	Acholeplasma_laidlawii	0.000213895	5.52275E-05	Parcubacteria_wastewater__metagenome	0.000200508	5.17709E-05	Amb-16S-1323_uncultured_bacterium	0.000228269	5.89388E-05	Alysiella_sp.	0	0	ASV_19144	4.91521E-05	1.2691E-05
Woeseia_sp.	0.000362424	9.35775E-05	ASV_18835	0.000213895	5.52275E-05	Phascolarctobacterium uncultured_Veillonellaceae	0.000200508	5.17709E-05	Eubacterium_minutum	0.00022625	5.84175E-05	Amaricoccus_sp.	0	0	Chloroflexi_KD4-96_metagenome	4.91521E-05	1.2691E-05
Dysgonomonadaceae_uncultured_Petrimonas	0.000359725	9.28806E-05	Beggiatoa_metagenome	0.000213895	5.52275E-05	Sediminibacterium_sp.	0.000200508	5.17709E-05	Prevotella_disiens	0.00022625	5.84175E-05	Amaricoccus_tamworthensis	0	0	Luedemannella_sp.	4.91521E-05	1.2691E-05
Bacteriovorax_uncultured_delta	0.000356506	9.20495E-05	Lachnospirales_uncultured__bacterium	0.000213895	5.52275E-05	Silvanigrellaceae_uncultured__bacterium	0.000200508	5.17709E-05	SediminispirochaetaSpirochaeta_sp.	0.000222441	5.79424E-05	Amb-16S-1323_uncultured_bacterium	0	0	Mitochondria_Eukaryota_sp.	4.91521E-05	1.2691E-05
Nocardioides_ganghwensis	0.000356506	9.20495E-05	Kocuria_marina	0.000213322	5.50532E-05	Sphingobacterium_thermophilum	0.000200508	5.17709E-05	Pseudolabrys_sp.	0.000223998	5.7836E-05	Aminobacter_sp.	0	0	Nitrosomonadaceae_uncultured__metagenome	4.91521E-05	1.2691E-05
Neisseriaceae_uncultured_Neisseriaceae_bacterium	0.000346801	8.95436E-05	Novosphingobium_sp.	0.000213322	5.50532E-05	Turneriella_metagenome	0.000200508	5.17709E-05	Candidatus_Limnoluna_uncultured_bacterium	0.000220089	4.7685E-05	Ammoniibacillus_agariperforans	0	0	ASV_21506	4.91219E-05	1.26832E-05
Catenisphaera_uncultured_rumen	0.000338481	8.73954E-05	Thermoflexibacter_sp.	0.000213322	5.50532E-05	Vicinamibacteria_uncultured__bacterium	0.000200508	5.17709E-05	Legionella_sp.	0.000206633	5.33524E-05	Ammoniphilus_oxalaticus	0	0	ASV_6442	4.91219E-05	1.26832E-05
Anaeromyxobacter_dehalogenans	0.000334709	8.64215E-05	Berkelbacteriauncultured_organism	0.000212906	5.49721E-05	Bacteroidetes_BD2-2_sp.	0.000198689	5.13013E-05	Spirochaeta_aurantia	0.000206633	5.33524E-05	Amnibacterium_sp.	0	0	Burkholderiales_sp.	4.91219E-05	1.26832E-05
Croceicoccus_sp.	0.000334709	8.64215E-05	Candidatus_Microthrix_sp.	0.000209293	5.40392E-05	Blautia_sp.	0.000198689	5.13013E-05	Solirubrobacterales_67-14_uncultured_organism	0.000205442	5.30449E-05	Amnipila_sp.	0	0	Emticicia_sp.	4.91219E-05	1.26832E-05
Desulfovibrio_gut__metagenome	0.000334709	8.64215E-05	Anaerolineaceae_uncultured__metagenome	0.000208478	5.38288E-05	Candidatus_Jorgensenbacteria__Parcubacteria_group	0.000198689	5.13013E-05	Truepera_uncultured_bacterium	0.000205442	5.30449E-05	Amycolatopsis_sp.	0	0	Vicinamibacteria_uncultured__sp.	4.91219E-05	1.26832E-05
Flavobacterium_ardleyense	0.000334709	8.64215E-05	ASV_4909	0.000208478	5.38288E-05	Saccharimonadales_LWQ8_metagenome	0.000198689	5.13013E-05	Cryptobacterium_sp.	0.000203214	5.24696E-05	Anaerobacterium_uncultured_bacterium	0	0	Blfdi19_uncultured_bacterium	4.88281E-05	1.26074E-05
ASV_4527	0.000330014	7.57604E-05	ASV_7512	0.000208478	5.38288E-05	Cloacibacterium_sp.	0.000198462	5.12427E-05	C1-B045_gamma_proteobacterium	0.00019994	5.16243E-05	Anaerobiospirillum_succiniciproducens	0	0	Candidatus_Woesebacteria_uncultured_eubacterium	4.88281E-05	1.26074E-05
Candidatus_Finniella_uncultured_bacterium	0.000328003	8.469E-05	Chloroplast_Melosira_varians	0.000208478	5.38288E-05	Anaerovoracaceae_Family_XIII_UCG-004Eubacterium_sp.	0.000195307	5.04281E-05	Fluviicola_sp.	0.00019994	5.16243E-05	Anaerococcus_hydrogenalis	0	0	Capnocytophaga_granulosa	4.88281E-05	1.26074E-05
Chloroplast_Chloromonas_radiata	0.000328003	8.469E-05	Cytophaga_hutchinsonii	0.00020057	5.31115E-05	Propionibacteriaceae_sp.	0.000195307	5.04281E-05	Hungateiclostridiaceae_sp.	0.00019994	5.16243E-05	Anaerococcus_prevotii	0	0	Acetitomaculum_sp.	4.87868E-05	1.25967E-05
Erysipelotrichaceae_UCG-003_sp.	0.000328003	8.469E-05	ASV_12032	0.000195163	5.03909E-05	Alphaproteobacteria_uncultured_Novosphingobium	0.000188875	4.87673E-05	ASV_21656	0.000194704	5.02724E-05	Anaerococcus_sp.	0	0	Apibacter_sp.	4.85327E-05	1.25311E-05
Paracaedibacteraceae_uncultured__metagenome	0.000328003	8.469E-05	ASV_21156	0.000194704	5.02724E-05	ASV_17446	0.000188875	4.87673E-05	ASV_21657	0.000194704	5.02724E-05	Anaerococcus_uncultured_bacterium	0	0	Candidatus_Magasanikbacteria__Parcubacteria_group	4.84051E-05	1.24981E-05
Pseudokineococcus_sp.	0.000328003	8.469E-05	Aureimonas_sp.	0.000194704	5.02724E-05	Patescibacteria_CPR2_uncultured_Firmicutes	0.000188875	4.87673E-05	Pedosphaeraceae_SH3-11_uncultured_bacterium	0.000194704	5.02724E-05	Anaerocolumna_uncultured_bacterium	0	0	Aquicella_uncultured_bacterium	0.000044617	1.15201E-05
Coriobacteriia_OPB41uncultured_Coriobacteriia	0.000323753	8.35927E-05	Christensenellaceae_R-7_groupunidentified_rumen	0.000194704	5.02724E-05	Aeromonas_sp.	0.000187512	4.84154E-05	Anaerolineaceae_uncultured__sp.	0.000186665	4.81967E-05	Anaerofilumuncultured_Anaerofilum	0	0	Arenimonas_oryziterrae	0.000044617	1.15201E-05
GKS98_freshwater_group_sp.	0.000323753	8.35927E-05	Isosphaeraceae_uncultured__sp.	0.000194704	5.02724E-05	ASV_19081	0.000187512	4.84154E-05	Candidatus_Anammoximicrobium_sp.	0.000186665	4.81967E-05	Anaerofustis_uncultured_bacterium	0	0	Candidatus_Amesbacteria	0.000044617	1.15201E-05
ASV_21307	0.000321716	8.30667E-05	Oligoflexia_uncultured__metagenome	0.000194704	5.02724E-05	Beijerinckiaceae_1174-901-12_sp.	0.000187512	4.84154E-05	CL500-29_marine_group_metagenome	0.000186665	4.81967E-05	Anaeroglobus_uncultured_organism	0	0	MBNT15_uncultured_bacterium	0.000044617	1.15201E-05
Candidatus_Falkowbacteria_sp.	0.000321716	8.30667E-05	Paenibacillus_glucanolyticus	0.000182845	4.72104E-05	DEV114_metagenome	0.000187512	4.84154E-05	Mesorhizobium_sp.	0.000186665	4.81967E-05	Anaerolineaceae_sp.	0	0	Nitrosomonadaceae_Ellin6067_uncultured_beta	0.000044617	1.15201E-05
Gammaproteobacteria_uncultured_organism	0.000321716	8.30667E-05	Luteolibacter_uncultured_Verrucomicrobiaceae	0.000177421	4.58099E-05	Fimbriiglobusuncultured_Planctomycetales	0.000187512	4.84154E-05	Pseudarcobacter_sp.	0.000186665	4.81967E-05	Anaerolineaceae_UCG-001_uncultured_bacterium	0	0	Patescibacteria_sp.	0.000044617	1.15201E-05
Paludibacter_sp.	0.000321716	8.30667E-05	Aestuariicella__uncultured_bacterium	0.000171116	4.4182E-05	Babeliales_UBA12409_sp.	0.000187126	4.83157E-05	Thermoleophilia_uncultured__bacterium	0.000186665	4.81967E-05	Anaerolineaceae_uncultured__bacterium	0	0	Microscillaceae_uncultured_Flexibacter	4.43636E-05	1.14546E-05
Parcubacteriawastewater__metagenome	0.000321716	8.30667E-05	Halieaceae_sp.	0.000171116	4.4182E-05	Saprospiraceae_sp.	0.000187126	4.83157E-05	Alloiococcus_uncultured_bacterium	0.000183557	4.73942E-05	Anaerolineaceae_uncultured__metagenome	0	0	Deep_Sea_Euryarchaeotic_Group(DSEG)_uncultured_archaeon	4.23227E-05	1.09277E-05
Prosthecobacter_uncultured_bacterium	0.000321716	8.30667E-05	Sporomusaceae_uncultured_uncultured_Veillonellaceae	0.000171116	4.4182E-05	Shinella_sp.	0.000187126	4.83157E-05	Rs-M59_termite_group_uncultured_bacterium	0.000180976	4.67278E-05	Anaerolineaceae_uncultured__sp.	0	0	Microscillaceae_uncultured_Cytophagales_bacterium	4.22436E-05	1.09073E-05
Ruminococcus_sp.	0.000321716	8.30667E-05	TaibaiellaBacteroidetes_bacterium	0.000171116	4.4182E-05	ASV_3544	0.000186825	4.8238E-05	uncultured_Capnocytophaga	0.000179163	3.47825E-05	Anaerolineaceae_uncultured__uncultured_soil	0	0	Moraxellaceae_uncultured__metagenome	4.22436E-05	1.09073E-05
Thermoanaerobacterium_sp.	0.000321716	8.30667E-05	Altererythrobacter_sp.	0.000166871	4.30859E-05	Frankiales_sp.	0.000186825	4.8238E-05	Candidatus_Finniella_sp.	0.000174388	4.50268E-05	Anaerolineaceae_uncultured_Bellilinea	0	0	ASV_20029	4.10884E-05	1.0609E-05
ASV_17320	0.000318928	8.23469E-05	CCM19a_sp.	0.000166871	4.30859E-05	Pedosphaeraceae_Ellin517_uncultured_bacterium	0.000182911	4.72274E-05	Candidatus_Kerfeldbacteria_metagenome	0.000174388	4.50268E-05	Anaerolineaceae_uncultured_delta	0	0	Caldilineaceae_uncultured__sp.	4.10884E-05	1.0609E-05
Blautia_metagenome	0.000318928	8.23469E-05	Saccharimonadales_metagenome	0.000166871	4.30859E-05	Pyramidobacter_piscolens	0.000182911	4.72274E-05	Chloroplast_Pseudopediastrum_boryanum	0.000174388	4.50268E-05	Anaerolineaceae_uncultured_eubacterium	0	0	Edaphobaculum_uncultured_bacterium	4.10884E-05	1.0609E-05
Candidatus_Pacebacteria_sp.	0.000318928	8.23469E-05	ASV_4563	0.000164844	4.25625E-05	Oligoflexia_uncultured_marine	0.000181923	4.69723E-05	Gracilibacteria_uncultured_bacterium	0.000174388	4.50268E-05	Anaerolineaceae_uncultured_Longilinea	0	0	Sphingobacteriales_AKYH767uncultured_Bacteroidetes	4.10884E-05	1.0609E-05
Erysipelotrichaceae_UCG-	0.00031	8.23469	Saprospiraceae_uncultured__bacterium	0.00016	4.25625	ShimazuellaShimazuella_sp.	0.00018	4.69723	Turicella_uncultured_bacterium	0.00017	4.50268	Anaerolineaceae_uncultured_sludge	0	0	ASV_18768	4.03375	1.04151

009_uncultured_rumen	8928	E-05		4844	E-05		1923	E-05		4388	E-05					E-05	E-05
Leucobacter_sp.	0.000318928	8.23469E-05	Thermoleophilia_sp.	0.000164844	4.25625E-05	ASV_11832	0.000172453	4.45272E-05	Babeliales_uncultured_bacterium	0.000171969	4.44022E-05	Anaerolineaceae_uncultured_toluene-degrading_methanogenic	0	0	ASV_18771	4.03375E-05	1.04151E-05
Atopostipes_uncultured_bacterium	0.000308801	7.97321E-05	ASV_6710	0.000159915	4.12899E-05	Thermoplasmata_uncultured__sp.	0.000172453	4.45272E-05	Beijerinckiaceae_sp.	0.000171969	4.44022E-05	Anaerolineae_1-20_sp.	0	0	ASV_18778	4.03375E-05	1.04151E-05
Bacteroides_stercoris	0.000308801	7.97321E-05	Ferruginibacter_sp.	0.000159915	4.12899E-05	Candidatus_Saccharimonas	0.000168402	3.19398E-05	Candidatus_SaccharimonasTM7_phylum	0.000171969	4.44022E-05	Anaerolineae_1-20uncultured_Chloroflexia	0	0	ASV_3544	4.03375E-05	1.04151E-05
Cytophaga_uncultured_bacterium	0.000308801	7.97321E-05	Gemmatimonadaceae_uncultured__sp.	0.000159915	4.12899E-05	Bulleidia_extructa	0.000167615	3.18675E-05	Clostridia_vadinBB60_group_uncultured_bacterium	0.000171969	4.44022E-05	Anaerolineae_A4b__metagenome	0	0	Sandaracinaceae_sp.	4.03375E-05	1.04151E-05
Lactobacillus_sp.	0.000308801	7.97321E-05	Lachnospiraceae_uncultured__metagenome	0.000159915	4.12899E-05	Alloscardovia_omnicolens	0.000167406	4.3224E-05	Gemmata_sp.	0.000171969	4.44022E-05	Anaerolineae_A4b__uncultured_bacterium	0	0	Stenotrophomonas_sp.	4.03375E-05	1.04151E-05
Saccharimonadalesuncultured_Epsilonproteobacteria	0.000308801	7.97321E-05	Methanospirillum_sp.	0.000159679	4.12289E-05	Clostridium_sensu_stricto_9_uncultured_bacterium	0.000167406	4.3224E-05	Methylomonadaceae_pLW-20_uncultured_bacterium	0.000171969	4.44022E-05	Anaerolineae_A4b__uncultured_soil	0	0	Leeia_uncultured_bacterium	3.87414E-05	5.47024E-06
Turicella_uncultured_bacterium	0.000308801	7.97321E-05	Microtrichaceae_uncultured__metagenome	0.000159679	4.12289E-05	Gallionella_sp.	0.000165385	4.27022E-05	Microtrichales_uncultured_wastewater__metagenome	0.000171969	4.44022E-05	Anaerolineae_A4b_sp.	0	0	Actinomyces_sp.	3.78181E-05	9.76459E-06
ASV_10605	0.000307267	7.9336E-05	Prolixibacteraceae_WCHB1-32_uncultured_bacterium	0.000159679	4.12289E-05	MidBa8_uncultured_delta	0.000165385	4.27022E-05	Beijerinckiaceae_uncultured__bacterium	0.000165201	4.26547E-05	Anaerolineae_A4b_wastewater__metagenome	0	0	ASV_21095	3.78181E-05	9.76459E-06
Saccharimonadalesmetal-contaminated_soil	0.000307267	7.9336E-05	Fimbriimonadaceae_sp.	0.000145836	3.76547E-05	SAR324_clade(Marine_group_B)bacterium_enrichment	0.000165385	4.27022E-05	Sandaracinus_uncultured_bacterium	0.000165201	4.26547E-05	Anaerolineae_sp.	0	0	Desulfobacterium_catecholicum_group_sp.	3.78181E-05	9.76459E-06
Cytophagaceae_uncultured_bacterium	0.000297519	7.68191E-05	Facklamia_uncultured_bacterium	0.000141937	3.6648E-05	Aggregatibacter_sp.	0.000161663	4.17412E-05	ASV_2510	0.000161371	3.05544E-05	Anaerolineae_uncultured__bacterium	0	0	Pirellulauncultured_organism	3.78181E-05	9.76459E-06
Lentimicrobiaceae_uncultured_Cytophagales	0.000297519	7.68191E-05	Uruburuella_sp.	0.000141937	3.6648E-05	Beutenbergiaceae_sp.	0.000160048	4.13242E-05	Candidatus_Nomurabacteria	0.000160707	4.14944E-05	Anaeromusa-Anaeroarcus_sp.	0	0	Acholeplasmauncultured_Acholeplasmataceae	3.68641E-05	9.51827E-06
Ruminococcus_lactaris	0.000297519	7.68191E-05	Bacteroides_sp.	0.000138985	3.58858E-05	C2U_uncultured_bacterium	0.000160048	4.13242E-05	Pirellula_uncultured_bacterium	0.000160707	4.14944E-05	Anaeromyxobacter_dehalogenans	0	0	Candidatus_Woykebacteria_uncultured_bacterium	3.68641E-05	9.51827E-06
Ahniella_metagenome	0.000297089	7.6708E-05	Candidatus_Peribacteria_uncultured_bacterium	0.000138985	3.58858E-05	Parcubacteria_GWA2-38-13b_uncultured_bacterium	0.000160048	4.13242E-05	Spirochaetaceae_uncultured__sp.	0.000159788	4.12571E-05	Anaeromyxobacter_uncultured_bacterium	0	0	Iamia_sp.	3.68641E-05	9.51827E-06
Rhodospirillales_uncultured__uncultured_soil	0.000297089	7.6708E-05	Dyella_sp.	0.000138985	3.58858E-05	Taibaiellauncultured_Sphingobacteriia	0.000160048	4.13242E-05	Candidatus_Lloydbacteria	0.000154631	3.99256E-05	Anaerorhabdus_furcosa_group__uncultured_bacterium	0	0	Novosphingobium_uncultured_eubacterium	3.68641E-05	9.51827E-06
Saccharimonadalessoil_bacterium	0.000297089	7.6708E-05	Eggerthellaceae_uncultured__bacterium	0.000138985	3.58858E-05	ASV_14332	0.00015779	4.07696E-05	Desulfobacterota_uncultured_Desulfuromonadales	0.000154631	3.99256E-05	Anaerosinus_uncultured_bacterium	0	0	Steroidobacteraceae_uncultured__sp.	3.68641E-05	9.51827E-06
Vicinamibacteria_uncultured__metagenome	0.000297089	7.6708E-05	Ilumatobacteraceae_sp.	0.000138985	3.58858E-05	PHOS-HE36_metagenome	0.00015779	4.07696E-05	Mucinivorans_hirudinis	0.000154631	3.99256E-05	Anaerostipes_sp.	0	0	Thermicanus_uncultured_bacterium	3.68641E-05	9.51827E-06
ASV_16316	0.000296171	7.6471E-05	Pedosphaeraceae_ADurb.Bin063-1__uncultured_bacterium	0.000137133	3.54076E-05	Actinobacteriota_MB-A2-108_uncultured_bacterium	0.000157396	4.06395E-05	Oligoflexia_0319-6G20_sp.	0.000154631	3.99256E-05	Anaerotignum_lactatifermentans	0	0	ASV_2390	3.66211E-05	9.45553E-06
Lysinibacillus_sp.	0.000296171	7.6471E-05	Thiothrix_uncultured_bacterium	0.000137133	3.54076E-05	ASV_18676	0.000157396	4.06395E-05	Parcubacteria_uncultured_Parcubacteria	0.000154631	3.99256E-05	Anaerovibrio_sp.	0	0	Candidatus_Ovatusbacter_uncultured_bacterium	3.66211E-05	9.45553E-06
ASV_3639	0.000293772	7.58516E-05	ASV_21883	0.000129803	3.3515E-05	Candidatus_Brennerbacteria__Parcubacteria_group	0.000157396	4.06395E-05	Dehalococcoidia_sp.	0.000152411	3.93524E-05	Anaerovoracaceae_Family_XIII_AD3011_group_sp.	0	0	Leptotrichiaceae_uncultured__bacterium	3.66211E-05	9.45553E-06
Candidatus_Protochlamydia_sp.	0.000293772	7.58516E-05	Absconditabacteriales_(SR1)__metagenome	0.000128337	3.31365E-05	Gastranaerophilales_sp.	0.000157396	4.06395E-05	Saccharimonadalesuncultured_Chloroflexi	0.000152411	3.93524E-05	Anaerovoracaceae_Family_XIII_AD3011_group_uncultured_bacterium	0	0	Methylotenera_uncultured_bacterium	3.66211E-05	9.45553E-06
Devosia_sp.	0.000293772	7.58516E-05	Atopobium__uncultured_rumen	0.000128337	3.31365E-05	Gottschalkia_acidurici	0.000157396	4.06395E-05	Abditibacterium_sp.	0.000149332	3.85574E-05	Anaerovoracaceae_Family_XIII_AD3011_group_uncultured_rumen	0	0	Selenomonadaceae_sp.	3.66211E-05	9.45553E-06
Elioraea_sp.	0.000293772	7.58516E-05	Fibrobacter_sp.	0.000128337	3.31365E-05	Ilumatobacteraceae_uncultured__bacterium	0.000157396	4.06395E-05	Alcaligenes_sp.	0.000149332	3.85574E-05	Anaerovoracaceae_Family_XIII_AD3011_grouncultured_Clostridiales	0	0	Aquihabitans_daechungensis	3.42015E-05	8.83079E-06
Holosporaceae_uncultured__bacterium	0.000293772	7.58516E-05	Saccharimonadaceae_sp.	0.000128337	3.31365E-05	Rubinisphaeraceae_SH-PL14__uncultured_planctomycete	0.000157396	4.06395E-05	Aquaspirillum_putridiconchylium	0.000149332	3.85574E-05	Anaerovoracaceae_Family_XIII_UCG-002_sp.	0	0	Oligoflexia_uncultured__bacterium	3.42015E-05	8.83079E-06
Methylacidiphilaceae_uncultured__bacterium	0.000293772	7.58516E-05	Sphingomonadaceae_uncultured__sp.	0.000128337	3.31365E-05	ASV_2303	0.000152207	2.68938E-05	Bauldia_uncultured_bacterium	0.000149332	3.85574E-05	Anaerovoracaceae_Family_XIII_UCG-003_sp.	0	0	Methylophaga_sp.	3.39195E-05	8.75798E-06
Micropepsaceae_uncultured__sp.	0.000293772	7.58516E-05	Anaerostipes_sp.	0.000124195	3.2067E-05	ASV_20352	0.000150009	3.87322E-05	Weeksella_sp.	0.000138209	3.56854E-05	Anaerovorax_sp.	0	0	Porphyromonas_pasteri	3.39195E-05	8.75798E-06
Nocardioides_dilutus	0.000293772	7.58516E-05	Candidatus_Kaiserbacteriauncultured_prokaryote	0.000124195	3.2067E-05	Flavobacterium_hauense	0.000150009	3.87322E-05	ASV_17593	0.000136961	3.53632E-05	Anaerovorax_uncultured_bacterium	0	0	ASV_2910	3.25246E-05	8.39782E-06
Planctomycetota_BD7-11_uncultured_Planctomycetia	0.000293772	7.58516E-05	Sandarakinorhabdus_sp.	0.000124195	3.2067E-05	Hirschia_uncultured_bacterium	0.000150009	3.87322E-05	Thermoactinomyces_sp.	0.000136961	3.53632E-05	Anaerovoraxuncultured_prokaryote	0	0	ASV_5218	3.25246E-05	8.39782E-06
Pseudarcicella_hirudinis	0.000293772	7.58516E-05	Pedosphaeraceae_uncultured__bacterium	0.000120135	3.10187E-05	Lachnospiraceae_NK3A20_group_sp.	0.000150009	3.87322E-05	Micavibrionales_uncultured_sludge	0.000135476	3.49798E-05	Ancalomicrobium_sp.	0	0	Chitinophagaceae_uncultured__metagenome	3.23551E-05	8.35405E-06
Butyrivibrio_sp.	0.000293715	7.58369E-05	Singulisphaera_rosea	0.000114278	2.95065E-05	Paeniclostridium_sp.	0.000150009	3.87322E-05	ASV_14196	0.000124388	3.21168E-05	Aneurinibacillus_uncultured_bacterium	0	0	Crinalium_SAG_22.89_sp.	3.23551E-05	8.35405E-06
Blastocatellia_11-24_uncultured_Acidobacteriales	0.000290297	7.49544E-05	Candidatus_Jorgensenbacteria	0.000111247	2.87239E-05	Patescibacteria_WWE3candidate_division	0.000150009	3.87322E-05	S0134_terrestrial_group_sp.	0.000124388	3.21168E-05	Angustibacter_uncultured_bacterium	0	0	Noviherbaspirillum_sp.	3.23551E-05	8.35405E-06

Saprospiraceae_uncultured__uncultured_soil	0.000290297	7.49544E-05	Rheinheimera_sp.	0.000111247	2.87239E-05	Clostridia_vadinBB60_group_Clostridiales_bacterium	0.000149057	3.84864E-05	Prevotella_shahii	0.000123064	3.1775E-05	Anoxybacillus_sp.	0	0	Rothia_sp.	3.23551E-05	8.35405E-06
SB-5_metagenome	0.000290297	7.49544E-05	Facklamia_tabacinasalis	0.000109896	2.8375E-05	Aurantimicrobium_uncultured_bacterium	0.000148846	3.84319E-05	BIrii41_sp.	0.000118542	3.06074E-05	Antarctic_bacterium	0	0	Saccharimonadalesuncultured_prokaryote	3.23551E-05	8.35405E-06
Acholeplasma__uncultured_bacterium	0.000289939	7.48619E-05	Alloprevotella_sp.	0.000010661	2.75266E-05	Williamwhitmaniaceae_uncultured__sp.	0.000148846	3.84319E-05	Diplorickettsiaceae_uncultured_agricultural_soil	0.000118542	3.06074E-05	Antricoccus_suffuscus	0	0	ASV_21176	0.0000317420	8.19575E-06
Anaerolineaceae_uncultured__uncultured_soil	0.000028778	7.43045E-05	ASV_164	0.000010661	2.75266E-05	Corynebacterium_xerosis	0.000143711	3.7106E-05	Gemmatimonas_photrophica	0.000118542	3.06074E-05	Apibacter_sp.	0	0	Candidatus_Adlerbacteria	0.0000317420	8.19575E-06
Roseburia_sp.	0.000028778	7.43045E-05	Candidatus_Falkowbacteria__uncultured_Parcubacteria	0.000010661	2.75266E-05	Acidimicrobiia_uncultured__bacterium	0.000140344	3.62367E-05	ASV_1837	0.000116259	3.00179E-05	Aquabacterium_sp.	0	0	Friedmanniella_sp.	0.0000317420	8.19575E-06
ASV_20940	0.000281399	7.26569E-05	Parcubacteria_uncultured_soil	0.000010661	2.75266E-05	ASV_20460	0.000140344	3.62367E-05	Clostridium_sensu_stricto_8_iron-reducing_bacterium	0.000114134	2.94693E-05	Aquaspirillum_putridiconchylium	0	0	Parcubacteria_bacterium	0.0000317420	8.19575E-06
Gammaproteobacteria_211ds20_sp.	0.000274134	7.07811E-05	Alphaproteobacteria_uncultured_Alphaproteobacteria	0.000106453	2.7486E-05	Blfdi19_metagenome	0.000140344	3.62367E-05	Rhodospirillales_uncultured__bacterium	0.000114134	2.94693E-05	Aquaspirillum_sp.	0	0	Alloprevotella_uncultured_Bacteroidales	3.16827E-05	8.18044E-06
Chloroplast_Guillardia_theta	0.000271389	6.48518E-05	ASV_16822	0.000106453	2.7486E-05	Izemoplasmatales_uncultured_bacterium	0.000140344	3.62367E-05	SHA-41_uncultured_bacterium	0.000114134	2.94693E-05	Aquicella_metagenome	0	0	Hypsibius_dujardini	3.16827E-05	8.18044E-06
Desulfocapsaceae_uncultured__sp.	0.000265774	6.86226E-05	BergeyellaBergeyella_sp.	0.000106453	2.7486E-05	Rickettsiella_uncultured_bacterium	0.000140344	3.62367E-05	ASV_2820	0.000111999	2.8918E-05	Aquicella_sp.	0	0	Planctomycetes_uncultured__sp.	3.16827E-05	8.18044E-06
Lachnospiraceae_NK3A20_group_uncultured_rumen	0.000265774	6.86226E-05	Gammaproteobacteria_R7C24_uncultured_soil	0.000106453	2.7486E-05	Saccharimonadales_WWH38_uncultured_bacterium	0.000140344	3.62367E-05	ASV_3443	0.000111999	2.8918E-05	Aquicella_uncultured_bacterium	0	0	ASV_21123	3.08163E-05	7.95673E-06
Microvirga_sp.	0.000265774	6.86226E-05	Mitochondria_Eukaryota_sp.	0.000106453	2.7486E-05	Sutterellaceae_AAP99__uncultured_bacterium	0.000140344	3.62367E-05	BIrii41_uncultured_delta	0.000111999	2.8918E-05	Aquicella_unidentified	0	0	Eggerthellaceae_sp.	3.08163E-05	7.95673E-06
Sphingobacteriaceae_sp.	0.000026033	6.72169E-05	NB1-j_uncultured_bacterium	0.000106453	2.7486E-05	Anaerovoracaceae_Family_XIII_UCG-005Eubacterium_sp.	0.000139505	3.602E-05	Margulisbacteria_microbial_mat	0.000111999	2.8918E-05	Aquihabitans_daechungensis	0	0	Simkaniaceae_uncultured__metagenome	3.08163E-05	7.95673E-06
ASV_8275	0.000254553	6.57253E-05	Lacihabitans_sp.	9.14223E-05	2.36051E-05	Clostridium_sensu_stricto_10_uncultured_bacterium	0.000139505	3.602E-05	Margulisbacteria_uncultured_bacterium	0.000111999	2.8918E-05	Aquisphaera_sp.	0	0	Flavobacterium_	2.71356E-05	7.00638E-06
Deinococcus_antarcticus	0.000254553	6.57253E-05	Catenulispora_sp.	8.87107E-05	2.2905E-05	Gastranaerophilales_uncultured_prokaryote	0.000135092	3.48806E-05	Synechocystis_PCC-6803_uncultured_bacterium	0.000111999	2.8918E-05	Aquisphaera_uncultured_bacterium	0	0	Williamwhitmania_sp.	2.71356E-05	7.00638E-06
Pedomicrobium_uncultured_Hyphomicrobium	0.000254553	6.57253E-05	Methylocystis_sp.	8.87107E-05	2.2905E-05	ASV_21460	0.000133672	3.4514E-05	ASV_15165	0.000110567	2.85483E-05	Arachis_hypogaea	0	0	ASV_21838	2.52121E-05	6.50974E-06
Rhizorhapis_uncultured_Alphaproteobacteria	0.000254553	6.57253E-05	Rivicola_sp.	8.87107E-05	2.2905E-05	ASV_21461	0.000133672	3.4514E-05	Cellvibrio_uncultured_Cellvibrio	0.000110134	2.84365E-05	Arboricoccus_pini	0	0	Bacilli_RF39_sp.	2.52121E-05	6.50974E-06
Amaricoccus_sp.	0.000253861	6.55466E-05	Alphaproteobacteria_uncultured__bacterium	8.55578E-05	2.20909E-05	ASV_21463	0.000133672	3.4514E-05	Gammaproteobacteria_R7C24_metagenome	0.000110134	2.84365E-05	Arcicella_rigui	0	0	Bdellovibrio_uncultured_proteobacterium	2.52121E-05	6.50974E-06
Clostridia_UCG-014_uncultured_organism	0.000253697	6.55043E-05	Microtrichaceae_IMCC26207_metagenome	8.55578E-05	2.20909E-05	Corynebacterium_glutamicum	0.000133672	3.4514E-05	Magnetospirillum_sp.	0.000110134	2.84365E-05	Arcicella_sp.	0	0	Candidatus_Falkowbacteria_metagenome	2.52121E-05	6.50974E-06
Rhodoferax_sp.	0.000253697	6.55043E-05	ASV_19339	7.09685E-05	1.8324E-05	Elusimicrobiota_Lineage_IV_uncultured_bacterium	0.000133672	3.4514E-05	Porphyrobacter_sp.	0.000110134	2.84365E-05	Arcicella_uncultured_bacterium	0	0	Diplorickettsiaceae_uncultured__metagenome	2.52121E-05	6.50974E-06
ASV_7514	0.000251808	6.50165E-05	Fluviicola_metagenome	7.09685E-05	1.8324E-05	Hydrogenophilus_sp.	0.000133672	3.4514E-05	Rhodocyclaceae_uncultured__sp.	0.000110134	2.84365E-05	Arcobacter_cryaerophilus	0	0	Emticicia_paludis	2.52121E-05	6.50974E-06
Exiguobacterium_undae	0.000251808	6.50165E-05	Novispirillum_sp.	7.09685E-05	1.8324E-05	Ilumatobacteraceae_uncultured_Actinomycetales	0.000133672	3.4514E-05	Serratia_sp.	0.000107171	2.76714E-05	Arcobacter_sp.	0	0	Microgenomatia_sp.	2.52121E-05	6.50974E-06
Peptostreptococcaceae_sp.	0.000251808	6.50165E-05	Aquicella_sp.	6.85667E-05	1.77038E-05	Tundrisphaera_uncultured_bacterium	0.000133672	3.4514E-05	Patescibacteria_WWE3_uncultured_bacterium	0.000101607	2.62348E-05	Arcobacteraceae_sp.	0	0	Patescibacteria_WWE3uncultured_candidate	2.52121E-05	6.50974E-06
Blastocatellaceae_JGI_0001001-H03uncultured_Acidobacterium	0.000246002	6.35174E-05	Cryptosporangium_sp.	6.85667E-05	1.77038E-05	ASV_1071	0.000132459	3.42008E-05	Leptolinea_uncultured_soil	9.67466E-05	2.49799E-05	Arcobacteraceae_uncultured__sp.	0	0	Planctomycetes_uncultured__planctomycete	2.52121E-05	6.50974E-06
Candidatus_Bealeia_uncultured_bacterium	0.000246002	6.35174E-05	Cyclobacteriaceae_uncultured_Aquiflexum_sp.	6.85667E-05	1.77038E-05	Bacteroides_ovatus	0.000132459	3.42008E-05	Malikia_sp.	9.67466E-05	2.49799E-05	Arcticibacter_sp.	0	0	SAR324_clade(Marine_group_B)_metagenome	2.52121E-05	6.50974E-06
Candidatus_Zambryskibacteria	0.000246002	6.35174E-05	Kapabacterialesuncultured_Bacteroidetes/Chlorobi	6.85667E-05	1.77038E-05	Candidatus_Curtissbacteria_sp.	0.000132459	3.42008E-05	Alloscardovia_omnicolens	0.000095263	2.45968E-05	Arcticibacter_uncultured_bacterium	0	0	ASV_4527	2.45761E-05	6.34552E-06
Thermodesulfovibrionia_uncultured_Nitrospiraceae	0.000246002	6.35174E-05	Prolixibacteraceae_WCHB1-32_sp.	6.85667E-05	1.77038E-05	Candidatus_Magasanikbacteria_uncultured_bacterium	0.000132459	3.42008E-05	ASV_1161	9.17785E-05	2.36971E-05	Arenicellaceae_uncultured_prokaryote	0	0	Eubacterium_siraeum	2.45761E-05	6.34552E-06
ASV_19610	0.000237671	6.13664E-05	ASV_7664	5.33049E-05	1.37633E-05	Clostridium_magnum	0.000132459	3.42008E-05	ASV_13680	9.17785E-05	2.36971E-05	Arenimonas_oryziterrae	0	0	Thymallus_thymallus	2.45761E-05	6.34552E-06
Butyricicoccus_sp.	0.000237671	6.13664E-05	Antricoccus_suffuscus	5.32264E-05	1.3743E-05	Weeksellaceae_sp.	0.000132459	3.42008E-05	ASV_3580	9.17785E-05	2.36971E-05	Arenimonas_sp.	0	0	ASV_8590	0.0000024561	6.34162E-06
Haliangium_uncultured_proteobacterium	0.000237671	6.13664E-05	ASV_20551	5.32264E-05	1.3743E-05	Acidiphilium__uncultured_bacterium	0.000132308	3.41618E-05	Aurantisolimonas_uncultured_bacterium	9.17785E-05	2.36971E-05	Aridibacter_sp.	0	0	Absconditabacteriales_(SR1)_uncultured_candidate	2.44141E-05	6.30369E-06
Kapabacterialesuncultured_Chlorobi	0.000237671	6.13664E-05	ASV_20552	5.32264E-05	1.3743E-05	ASV_14583	0.000132308	3.41618E-05	Cellulomonas_sp.	9.17785E-05	2.36971E-05	Armatimonadales_metagenome	0	0	ASV_21925	2.44141E-05	6.30369E-06

Leptotrichiaceae_uncultured__metagenome	0.000237671	6.13664E-05	Brevibacterium_sp.	5.32264E-05	1.3743E-05	Dinghuibacter_sp.	0.000132308	3.41618E-05	Herminiimonas_sp.	9.17785E-05	2.36971E-05	Armatimonadales_uncultured_bacterium	0	0	ASV_3639	2.44141E-05	6.30369E-06
Lysinibacillus_uncultured_prokaryote	0.000237671	6.13664E-05	Candidatus_Liptonbacteria__Parcubacteria_group	5.32264E-05	1.3743E-05	Endobacter_uncultured_bacterium	0.000132308	3.41618E-05	Elusimicrobiota_Lineage_IIa_sp.	9.13075E-05	2.35755E-05	Armatimonadales_uncultured_Chloroflexi	0	0	ASV_5871	2.42025E-05	6.24906E-06
Spirosoma_lituiforme	0.000237671	6.13664E-05	Christensenellaceae_R-7_group_bacterium_AC2043	5.32264E-05	1.3743E-05	Prevotella_loescheii	0.000132308	3.41618E-05	Flavobacterium_noncentrifugens	9.13075E-05	2.35755E-05	Armatimonadales_uncultured_eubacterium	0	0	Blastocatellia_11-24__uncultured_bacterium	2.42025E-05	6.24906E-06
ASV_11306	0.000234972	6.06695E-05	Fibrobacterales_sp.	5.32264E-05	1.3743E-05	Candidatus_Magasanikbacteria__uncultured_soil	0.00012934	3.33954E-05	Rubellimicrobium_uncultured_bacterium	9.13075E-05	2.35755E-05	Armatimonadalesuncultured_Armatimonadetes	0	0	Candidatus_Staskawiczbacteria_uncultured_bacterium	2.42025E-05	6.24906E-06
Nitrospinota_P9X2b3D02_sp.	0.000234972	6.06695E-05	Lentimicrobiaceae_uncultured_Sphingobacteriales	5.32264E-05	1.3743E-05	Granulicella_tundricola	0.00012934	3.33954E-05	Anticoccus_suffuscus	8.59845E-05	2.22011E-05	Armatimonadia_sp.	0	0	Hyphomonadaceae_uncultured__bacterium	2.42025E-05	6.24906E-06
Rhizorhapis_uncultured_bacterium	0.000234972	6.06695E-05	Segetibacter_uncultured_Bacteroidetes	5.32264E-05	1.3743E-05	Rhizobiales_A0839_sp.	0.000127383	3.28901E-05	Candidatus_Berkiella_uncultured_bacterium	8.46726E-05	2.18624E-05	Armatimonadota_uncultured__bacterium	0	0	Rickettsiales_SM2D12uncultured_Hyphomicrobiaceae	2.42025E-05	6.24906E-06
ASV_17573	0.00022314	5.76145E-05	Sphingobacteriales_env.OPS_17_uncultured_Bacteroidetes	5.32264E-05	1.3743E-05	Blastocatella_sp.	0.000125917	3.25116E-05	Ruminococcus_bicirculans	8.46726E-05	2.18624E-05	Armatimonadota_uncultured__metagenome	0	0	Aurantisolimonas_metagenome	2.11613E-05	5.46382E-06
Actinobacteria_PeM15_uncultured_soil	0.000217723	5.62158E-05	Candidatus_Metachlamydia	4.57112E-05	1.18026E-05	Fusibacter_sp.	0.000125917	3.25116E-05	Prevotella_micans	8.33552E-05	2.15222E-05	Armatimonadota_uncultured__sp.	0	0	Candidatus_Limnoluna_uncultured_bacterium	2.11613E-05	5.46382E-06
Woesearchaeales_GW2011_GWC1_47_15_Candidatus_Pacearchaeota	0.000217723	5.62158E-05	Clostridioides_sp.	4.57112E-05	1.18026E-05	Porphyrobacter_mercurialis	0.000125917	3.25116E-05	ASV_17471	8.29256E-05	2.14113E-05	Armatimonadota_uncultured__uncultured_soil	0	0	Pseudomonas_sp.	2.11613E-05	5.46382E-06
Methylophilaceae_MM1_uncultured_bacterium	0.000217454	5.61464E-05	Candidatus_Abawacabacteria	4.27789E-05	1.10455E-05	ASV_3026	0.000125664	2.25821E-05	Burkholderiales_TRA3-20uncultured_beta	8.29256E-05	2.14113E-05	Armatimonas_sp.	0	0	Rubinisphaeraceae_SH-PL14__uncultured_planctomycete	2.11613E-05	5.46382E-06
Rickettsiaceae_uncultured__metagenome	0.000215835	5.57284E-05	alphaproteobacteria_uncultured__bacterium	3.54843E-05	9.16201E-06	Deinococcus_aquatilis	0.000123625	3.19198E-05	Curvibacter_sp.	8.29256E-05	2.14113E-05	Armatimonas_uncultured_bacterium	0	0	Candidatus_Woesebacteria_uncultured_bacterium	2.11218E-05	5.45363E-06
Spirochaetota_MVP-15_sp.	0.000215835	5.57284E-05	Anoxybacillus_sp.	3.54843E-05	9.16201E-06	Propionivibrio_sp.	0.000122811	3.17097E-05	Pedomicrobium_metagenome	8.29256E-05	2.14113E-05	Arsenicicoccus_bolidensis	0	0	Chitinophagales_uncultured_uncultured_Sphingobacteriia	2.05442E-05	5.30449E-06
Leptolinea_sp.	0.000215391	5.56137E-05	Candidatus_Kaiserbacteria_uncultured_bacterium	3.54843E-05	9.16201E-06	DesulfovibrioDesulfovibrio_putealis	0.000115769	2.98914E-05	Propionibacter_sp.	7.96294E-05	2.05602E-05	Arthrobacter_sp.	0	0	Clostridium_sp.	2.05442E-05	5.30449E-06
Candidatus_Pacearchaeota	0.000214477	5.53777E-05	Coprococcus_metagenome	3.54843E-05	9.16201E-06	Marvinbryantia_sp.	0.000115769	2.98914E-05	Chitinophagales_uncultured__bacterium	7.73156E-05	1.99628E-05	Aspergillus_nidulans	0	0	Fibrobacteraceae_possible_genus_04_uncultured_bacterium	2.05442E-05	5.30449E-06
Chitinophagaceae_uncultured__sp.	0.000214477	5.53777E-05	Peredibacter_sp.	3.54843E-05	9.16201E-06	Mitochondria_Eukaryota_sp.	0.000114969	2.96849E-05	ASV_5832	7.46659E-05	1.92787E-05	Asticcacaulis_sp.	0	0	Formivibrio_uncultured_bacterium	2.05442E-05	5.30449E-06
Chthoniobacter_sp.	0.000214477	5.53777E-05	Polyangia_mle1-27uncultured_Polyangiaceae	3.54843E-05	9.16201E-06	Sphingorhabdus_uncultured_bacterium	0.000114969	2.96849E-05	Candidatus_Azambacteria_sp.	7.46659E-05	1.92787E-05	ASV_10003	0	0	Gracilibacteria_sp.	2.05442E-05	5.30449E-06
Fimbriimonadaceae_uncultured_Armatimonadetes	0.000214477	5.53777E-05	009E01-B-SD-P15__uncultured_bacterium	0	0	Armatimonadota_uncultured__uncultured_soil	0.00011432	2.95173E-05	Fluviicola_uncultured_bacterium	7.46659E-05	1.92787E-05	ASV_10004	0	0	Mitochondria_metagenome	2.05442E-05	5.30449E-06
Lacihabitans_uncultured_bacterium	0.000214477	5.53777E-05	1013-28-CG33__uncultured_soil	0	0	Clostridium_sensu_stricto_8_sp.	0.00011432	2.95173E-05	Lewinella_uncultured_bacterium	7.46659E-05	1.92787E-05	ASV_10035	0	0	Oligoflexia_uncultured__sp.	2.05442E-05	5.30449E-06
SAR324_clade(Marine_group_B)_uncultured_bacterium	0.000214477	5.53777E-05	Abditibacterium_sp.	0	0	Rhizobiaceae_sp.	0.00011432	2.95173E-05	Rubinisphaeraceae_SH-PL14_uncultured_bacterium	7.46659E-05	1.92787E-05	ASV_10043	0	0	Pirellula_sp.	2.05442E-05	5.30449E-06
Terrimicrobium_uncultured_bacterium	0.000214477	5.53777E-05	Absconditabacteriales_(SR1)__uncultured_bacterium	0	0	Thermobacillus_uncultured_bacterium	0.00011432	2.95173E-05	Caulobacteraceae_PMMR1uncultured_endolithic	7.34228E-05	1.89577E-05	ASV_10098	0	0	Spirochaetaceae_uncultured_organism	2.05442E-05	5.30449E-06
Chloroflexi_OLB14_sp.	0.000212619	5.4898E-05	Absconditabacteriales_(SR1)_candidate_division	0	0	Clostridia_uncultured_Clostridiaceae	0.000112507	2.90492E-05	Gemmataceae_sp.	7.34228E-05	1.89577E-05	ASV_10170	0	0	ASV_3580	1.71007E-05	4.41538E-06
Duganella_sp.	0.000212619	5.4898E-05	Absconditabacteriales_(SR1)_sp.	0	0	Diplorickettsia_uncultured_bacterium	0.000112507	2.90492E-05	Rickettsiales_SM2D12uncultured_Alphaproteobacteria	7.34228E-05	1.89577E-05	ASV_10179	0	0	Clostridium_sensu_stricto_1_metagenome	1.61776E-05	4.17704E-06
ASV_18750	0.000211551	5.46222E-05	Absconditabacteriales_(SR1)_SR1_bacterium	0	0	Oligoflexales_uncultured__uncultured_soil	0.000112507	2.90492E-05	Pseudopropionibacterium_uncultured_bacterium	7.14473E-05	1.84476E-05	ASV_1021	0	0	Capnocytophaga_sp.	0.000016135	4.16604E-06
C39_sp.	0.000211551	5.46222E-05	Absconditabacteriales_(SR1)_uncultured_candidate	0	0	Microbacteriaceae_sp.	0.000111604	2.8816E-05	Agromyces_sp.	6.91047E-05	1.78428E-05	ASV_10253	0	0	SBR1031_uncultured_Gemmatimonadetes	0.000016135	4.16604E-06
Dermacoccus_sp.	0.000211551	5.46222E-05	Absconditabacteriales_(SR1)_uncultured_Epsilonproteobacteria	0	0	ASV_17662	0.000105267	2.71798E-05	ASV_18698	6.91047E-05	1.78428E-05	ASV_10292	0	0	Subdoligranulum_sp.	0.000016135	4.16604E-06
Oligella_urethralis	0.000211551	5.46222E-05	Absconditabacteriales_(SR1)_uncultured_organism	0	0	SediminispirochaetaSpirochaeta_sp.	0.000103692	2.67732E-05	Fimbriiglobus_sp.	6.91047E-05	1.78428E-05	ASV_10316	0	0	Acinetobacter_sp.	1.35678E-05	3.50319E-06
Oscillospiraceae_UCG-005uncultured_prokaryote	0.000211551	5.46222E-05	Absconditabacteriales_(SR1)_uncultured_prokaryote	0	0	Pedosphaeraceae_metagenome	0.000100598	2.59743E-05	Paludibaculum_metagenome	6.91047E-05	1.78428E-05	ASV_10346	0	0	Proteobacteria_sp.	1.35678E-05	3.50319E-06
Polyangia_mle1-27_metagenome	0.000211551	5.46222E-05	ABY1_sp.	0	0	Phreatobacter_uncultured_Rhizobiales	0.000100598	2.59743E-05	Absconditabacteriales_(SR1)_sp.	6.84806E-05	1.76816E-05	ASV_10385	0	0	Prevotella_baroniae	6.78389E-06	1.75159E-06
Salinicoccus_albus	0.000211551	5.46222E-05	Acanthopleuribacter__uncultured_bacterium	0	0	Ralstonia_sp.	0.000100031	1.93484E-05	Actinobacteria_sp.	6.84806E-05	1.76816E-05	ASV_10460	0	0	009E01-B-SD-P15__uncultured_bacterium	0	0
Sphingobacteriales_AKYH767_metagenome	0.000211551	5.46222E-05	Acetitomaculum__uncultured_bacterium	0	0	Anaerolineae_A4b_sp.	0.000099231	2.56213E-05	Flavobacteriales_NS9_marine_groupuncultured_Bacteroidetes	6.84806E-05	1.76816E-05	ASV_10537	0	0	1013-28-CG33__uncultured_soil	0	0

Sporichthyaceae_hgcl_clade_Candidatus_Planktophila	0.000211551	5.46222E-05	Acetitomaculum__uncultured_rumen	0	0	ASV_9544	0.000099231	2.56213E-05	Kiritimatiellae_WCHB1-41_uncultured_rumen	6.84806E-05	1.76816E-05	ASV_10583	0	0	Abditibacterium_sp.	0	0
ASV_2249	0.000207503	4.02756E-05	Acetitomaculum_sp.	0	0	Cereibacter_sp.	0.000099231	2.56213E-05	Luteolibacter_sp.	6.84806E-05	1.76816E-05	ASV_10605	0	0	Absconditabacteriales_(SR1)__metagenome	0	0
Azospirillum_uncultured_bacterium	0.000205867	5.31546E-05	Acetivibrio_uncultured_bacterium	0	0	Chloroplast_groundwater__metagenome	0.000099231	2.56213E-05	Oligoflexia_0319-6G20_uncultured_Syntrophobacteraceae	6.84806E-05	1.76816E-05	ASV_10606	0	0	Absconditabacteriales_(SR1)__uncultured_bacterium	0	0
Frankiales_uncultured_actinobacterium	0.000205867	5.31546E-05	Acetoanaerobium_uncultured_bacterium	0	0	Chloroplast_Hafniomonas_laevis	0.000099231	2.56213E-05	Parcubacteriauncultured_organism	6.84806E-05	1.76816E-05	ASV_10613	0	0	Absconditabacteriales_(SR1)_candidate_division	0	0
Polyangia_mle1-27uncultured_proteobacterium	0.000205867	5.31546E-05	Acetobacter_sp.	0	0	Dehalococcoidia_uncultured_Chloroflexi	0.000099231	2.56213E-05	Sporichthyaceae_hgcl_clade_sp.	6.84806E-05	1.76816E-05	ASV_10814	0	0	Absconditabacteriales_(SR1)_sp.	0	0
Rhizobiales_Incertae_Sedis_uncultured__sp.	0.000205867	5.31546E-05	Acetobacteraceae_sp.	0	0	Edaphobaculum_metagenome	0.000099231	2.56213E-05	ASV_19840	6.77381E-05	1.74899E-05	ASV_10821	0	0	Absconditabacteriales_(SR1)_SR1_bacterium	0	0
Tyzzerella_uncultured_Clostridium	0.000205867	5.31546E-05	Acetobacteraceae_uncultured__bacterium	0	0	Nitrospira_defluvii	0.000099231	2.56213E-05	FCPU426_uncultured_bacterium	6.77381E-05	1.74899E-05	ASV_10869	0	0	Absconditabacteriales_(SR1)_uncultured_Epsilonproteobacteria	0	0
ASV_4909	0.000200839	3.53469E-05	Acetobacteraceae_uncultured__metagenome	0	0	oc32_metagenome	0.000099231	2.56213E-05	Thermomicrobiales_JG30-KF-CM45_uncultured_bacterium	6.77381E-05	1.74899E-05	ASV_10995	0	0	Absconditabacteriales_(SR1)_uncultured_organism	0	0
Caulobacter_sp.	0.000195848	5.05677E-05	Acetobacteraceae_uncultured__sp.	0	0	Verrucomicrobiae_sp.	0.000099231	2.56213E-05	Cutibacterium_granulosum	5.81294E-05	1.50089E-05	ASV_11002	0	0	Absconditabacteriales_(SR1)_uncultured_prokaryote	0	0
Opitutaceae_sp.	0.000195848	5.05677E-05	Acetobacterium_sp.	0	0	Empedobacter_brevis	9.44376E-05	2.43837E-05	ASV_20010	5.52837E-05	1.42742E-05	ASV_11030	0	0	ABY1_sp.	0	0
Rickettsiales_SM2D12_metagenome	0.000195848	5.05677E-05	Acetothermiia__uncultured_bacterium	0	0	SepB-3_uncultured_bacterium	9.44376E-05	2.43837E-05	Peptoclostridium_uncultured_organism	5.52837E-05	1.42742E-05	ASV_11102	0	0	Acanthopleuribacter__uncultured_bacterium	0	0
Schlesneria_uncultured_planctomycete	0.000195848	5.05677E-05	Acholeplasma__uncultured_bacterium	0	0	Aeromonas_uncultured_gamma	9.35629E-05	2.41578E-05	Ruminococcus_metagenome	5.52837E-05	1.42742E-05	ASV_11103	0	0	Acetitomaculum__uncultured_bacterium	0	0
Enterovibrio_uncultured_bacterium	0.00019581	5.05579E-05	Acholeplasma_axanthum	0	0	ASV_21484	9.35629E-05	2.41578E-05	Coxiella_uncultured_Coxiellaceae	5.50671E-05	1.42183E-05	ASV_11163	0	0	Acetitomaculum__uncultured_rumen	0	0
Gemmataceae_uncultured_Crater	0.00019581	5.05579E-05	Acholeplasma_brassicae	0	0	ASV_21486	9.35629E-05	2.41578E-05	Defluviitaleaceae_UCG-011_uncultured_bacterium	5.50671E-05	1.42183E-05	ASV_11164	0	0	Acetivibrio_uncultured_bacterium	0	0
Luteolibacter_uncultured_bacterium	0.00019581	5.05579E-05	Acholeplasma_morum	0	0	Haliangium_sp.	9.35629E-05	2.41578E-05	Gammaproteobacteria_EV818SWSAP88_uncultured_bacterium	5.50671E-05	1.42183E-05	ASV_11306	0	0	Acetoanaerobium_uncultured_bacterium	0	0
Bdellovibrio_uncultured_Bdellovibrio	0.0001876	4.84381E-05	Acholeplasma_sp.	0	0	Haliangium_uncultured_Myxococcales	9.35629E-05	2.41578E-05	Neochlamydia_uncultured_bacterium	5.50671E-05	1.42183E-05	ASV_11362	0	0	Acetobacter_sp.	0	0
Patescibacteria_CPR2_uncultured_Firmicutes	0.0001876	4.84381E-05	Acholeplasmataceae_EMP-G18_uncultured_Mollicutes	0	0	Nanoarchaeia_sp.	9.35629E-05	2.41578E-05	Phycisphaeraceae_SM1A02_sp.	5.50671E-05	1.42183E-05	ASV_11393	0	0	Acetobacteraceae_sp.	0	0
Planctopirus_metagenome	0.0001876	4.84381E-05	Acholeplasmauncultured_Acholeplasmataceae	0	0	Opitutus_metagenome	9.35629E-05	2.41578E-05	Afipia_sp.	5.08035E-05	1.31174E-05	ASV_11399	0	0	Acetobacteraceae_uncultured__bacterium	0	0
Aerococcaceae_sp.	0.00018595	4.80121E-05	Achromatium_sp.	0	0	Afipia_genosp.	9.14557E-05	2.36138E-05	Geobacteraceae_uncultured_delta	5.08035E-05	1.31174E-05	ASV_11414	0	0	Acetobacteraceae_uncultured__metagenome	0	0
Bacteroidetes_VC2.1_Bac22_metagenome	0.00018595	4.80121E-05	Acidaminobacter__uncultured_bacterium	0	0	ASV_2692	9.14557E-05	2.36138E-05	Rickettsiales_SM2D12_sp.	5.08035E-05	1.31174E-05	ASV_11446	0	0	Acetobacteraceae_uncultured__sp.	0	0
Rubinisphaeraceae_uncultured__sp.	0.00018595	4.80121E-05	Acidaminococcaceae_uncultured__bacterium	0	0	ASV_3803	9.14557E-05	2.36138E-05	Methylobacterium-Methylorubrum_sp.	4.76315E-05	1.22984E-05	ASV_11492	0	0	Acetobacterium_sp.	0	0
Leptospiraceae_RBG-16-49-21_uncultured_bacterium	0.000181212	4.67887E-05	Acidibacter__metagenome	0	0	Caulobacteraceae_sp.	9.14557E-05	2.36138E-05	ASV_3436	4.56538E-05	1.17878E-05	ASV_11530	0	0	Acetothermiia__uncultured_bacterium	0	0
eub62A3_uncultured_bacterium	0.000179863	4.64404E-05	Acidibacter__uncultured_bacterium	0	0	Paenibacillus_sp.	9.14557E-05	2.36138E-05	ASV_5779	4.56538E-05	1.17878E-05	ASV_11581	0	0	Acholeplasma__uncultured_bacterium	0	0
Magnetospirillum_uncultured_bacterium	0.000179863	4.64404E-05	Acidibacter_sp.	0	0	Pajaroellobacteruncultured_prokaryote	9.14557E-05	2.36138E-05	Caulobacter_metagenome	4.56538E-05	1.17878E-05	ASV_1161	0	0	Acholeplasma_axanthum	0	0
SR-FBR-L83_Ignavibacteria_bacterium	0.000179863	4.64404E-05	Acidimicrobiia_IMCC26256_sp.	0	0	Rhizobiales_A0839__metagenome	9.14557E-05	2.36138E-05	Faecalibacterium_sp.	4.56538E-05	1.17878E-05	ASV_11616	0	0	Acholeplasma_laidlawii	0	0
ASV_2390	0.000178253	4.60247E-05	Acidimicrobiia_IMCC26256_uncultured_bacterium	0	0	Acholeplasma_laidlawii	8.77224E-05	2.26498E-05	Fluviicola_uncultured_Fluviicola	4.56538E-05	1.17878E-05	ASV_11696	0	0	Acholeplasma_morum	0	0
ASV_5304	0.000178253	4.60247E-05	Acidimicrobiia_IMCC26256_uncultured_soil	0	0	Anaerovoraxuncultured_prokaryote	8.77224E-05	2.26498E-05	Spirochaeta_2_metagenome	4.56538E-05	1.17878E-05	ASV_11732	0	0	Acholeplasma_sp.	0	0
Gammaproteobacteria_uncultured__metagenome	0.000178253	4.60247E-05	Acidimicrobiia_IMCC26256uncultured_Aciditerrimonas	0	0	ASV_18896	8.77224E-05	2.26498E-05	Candidatus_Magasanikbacteria	4.14628E-05	1.07056E-05	ASV_11789	0	0	Acholeplasmataceae_EMP-G18_uncultured_Mollicutes	0	0
Polyangia_mle1-27_sp.	0.000178253	4.60247E-05	Acidimicrobiia_IMCC26256uncultured_actinobacterium	0	0	Azospirillum_sp.	8.77224E-05	2.26498E-05	Mucilaginibacter_sp.	4.14628E-05	1.07056E-05	ASV_11832	0	0	Achromatium_sp.	0	0
SB-5_sp.	0.000178253	4.60247E-05	Acidimicrobiia_IMCC26256uncultured_Actinomycetales	0	0	Diplorickettsiaceae_uncultured_proteobacterium	8.77224E-05	2.26498E-05	Nitrospira_japonica	4.14628E-05	1.07056E-05	ASV_12032	0	0	Acidaminobacter__uncultured_bacterium	0	0

vadinHA49_sp.	0.000178253	4.60247E-05	Acidimicrobiia_sp.	0	0	Gammaproteobacteria_JG36-TzT-191uncultured_proteobacterium	8.77224E-05	2.26498E-05	Saccharimonadales_LWQ8_uncultured_bacterium	4.14628E-05	1.07056E-05	ASV_1208	0	0	Acidaminococcaceae_uncultured__bacterium	0	0
Bifidobacterium_bifidum	0.000176229	4.55021E-05	Acidimicrobiia_uncultured__bacterium	0	0	Marinospirillum_minutulum	8.77224E-05	2.26498E-05	Verrucomicrobiaceae_uncultured__uncultured_soil	4.14628E-05	1.07056E-05	ASV_12134	0	0	Acidibacter__metagenome	0	0
ASV_20058	0.000169241	4.36978E-05	Acidimicrobiia_uncultured__metagenome	0	0	Opitutus_sp.	8.77224E-05	2.26498E-05	ASV_2061	3.67114E-05	9.47884E-06	ASV_12280	0	0	Acidibacter__uncultured_bacterium	0	0
ASV_2080	0.000169241	4.36978E-05	Acidimicrobiia_uncultured__sp.	0	0	Prosthecobacter_metagenome	8.77224E-05	2.26498E-05	ASV_21534	3.67114E-05	9.47884E-06	ASV_12284	0	0	Acidimicrobiia_IMCC26256_metagenome	0	0
Candidatus_Nucleicultrix	0.000169241	4.36978E-05	Acidimicrobiia_uncultured_Acidimicrobiia	0	0	Terrimicrobiumuncultured_Verrucomicrobiales	8.77224E-05	2.26498E-05	Cohnella_sp.	3.67114E-05	9.47884E-06	ASV_12313	0	0	Acidimicrobiia_IMCC26256_sp.	0	0
Chryseobacterium_chaponense	0.000169241	4.36978E-05	Acidimicrobiia_uncultured_actinobacterium	0	0	Lactobacillus_rhamnosus	8.68931E-05	2.24357E-05	Johnsonella_Firmicutes_oral	3.61952E-05	9.34556E-06	ASV_12382	0	0	Acidimicrobiia_IMCC26256_uncultured_bacterium	0	0
Fibrobacterales_sp.	0.000169241	4.36978E-05	Acidimicrobiia_uncultured_Actinomycetales	0	0	ASV_17700	8.62267E-05	2.22636E-05	Butyrivibrio_Eubacterium_sp.	3.57236E-05	9.22379E-06	ASV_12391	0	0	Acidimicrobiia_IMCC26256uncultured_actinobacterium	0	0
Gaiellales_uncultured_uncultured_Solirubrobacter	0.000169241	4.36978E-05	Acidimicrobiia_uncultured_bacterium_YC-ZSS-LKJ199	0	0	Rubritaleaceae_uncultured_bacterium	8.62267E-05	2.22636E-05	Candidatus_Azambacteria	3.57236E-05	9.22379E-06	ASV_12397	0	0	Acidimicrobiia_IMCC26256uncultured_Actinomycetales	0	0
Rudanella_lutea	0.000164002	4.23451E-05	Acidiphilium__uncultured_bacterium	0	0	Fibrobacterales_B122_uncultured_bacterium	8.55863E-05	2.20983E-05	ASV_4673	0.000033869	8.74494E-06	ASV_12420	0	0	Acidimicrobiia_sp.	0	0
ASV_20977	0.000159464	4.11734E-05	Acidiphilium_sp.	0	0	Roseomonas_pecuniae	8.55863E-05	2.20983E-05	Bacilli_sp.	0.000033869	8.74494E-06	ASV_12452	0	0	Acidimicrobiia_uncultured__bacterium	0	0
Eubacterium_hallii_group__uncultured_rumen	0.000159464	4.11734E-05	Acidisoma_sp.	0	0	ASV_3531	8.49221E-05	2.19268E-05	CAG-352_uncultured_bacterium	0.000033869	8.74494E-06	ASV_12478	0	0	Acidimicrobiia_uncultured__metagenome	0	0
Bacteriovoracaceae_uncultured__sp.	0.000156648	4.04463E-05	Acidithiobacillaceae_KCM-B-112_sp.	0	0	Terrimonas_sp.	8.44324E-05	2.18004E-05	Candidatus_Yonathbacteria_uncultured_bacterium	0.000033869	8.74494E-06	ASV_12525	0	0	Acidimicrobiia_uncultured__sp.	0	0
Luteimonas_sp.	0.000156648	4.04463E-05	Acidithiobacillus_ferrooxidans	0	0	Prevotella_micans	0.000083703	2.1612E-05	Clostridium_sensu_stricto_1_sp.	0.000033869	8.74494E-06	ASV_12573	0	0	Acidimicrobiia_uncultured_Acidimicrobiia	0	0
Micavibrionales_uncultured_eubacterium	0.000156648	4.04463E-05	Acidobacteria_bacterium	0	0	Saccharimonadales_marine__metagenome	0.000083703	2.1612E-05	Jonquetella_anthropi	0.000033869	8.74494E-06	ASV_12623	0	0	Acidimicrobiia_uncultured_actinobacterium	0	0
Micromonospora_sp.	0.000156648	4.04463E-05	Acidobacteriaceae_(Subgroup_1)_sp.	0	0	Woesearchaeales_uncultured_archaeon	0.000083703	2.1612E-05	Megamonas_sp.	0.000033869	8.74494E-06	ASV_12634	0	0	Acidimicrobiia_uncultured_Actinomycetales	0	0
Pseudolabrys_metagenome	0.000156648	4.04463E-05	Acidobacteriae_Elev-16S-1166_sp.	0	0	Cellulosimicrobium_sp.	8.26925E-05	2.13511E-05	Blastocatellaceae_uncultured__sp.	2.89561E-05	7.47643E-06	ASV_12731	0	0	Acidimicrobiia_uncultured_bacterium_YC-ZSS-LKJ199	0	0
Rs-M47uncultured_organism	0.000156648	4.04463E-05	Acidobacteriae_sp.	0	0	Chitinophaga_sp.	8.26925E-05	2.13511E-05	Leptotrichia_hongkongensis	2.89561E-05	7.47643E-06	ASV_12760	0	0	Acidiphilium__uncultured_bacterium	0	0
ASV_20091	0.00014876	3.84097E-05	Acidocella__uncultured_bacterium	0	0	Microscillaceae_uncultured_Flexibacteraceae	8.26925E-05	2.13511E-05	Woesearchaeales_uncultured_bacterium	2.89561E-05	7.47643E-06	ASV_12772	0	0	Acidisoma_sp.	0	0
Bradymonadales_uncultured_bacterium	0.00014876	3.84097E-05	Acidothermus__uncultured_bacterium	0	0	Yersiniaceae_sp.	8.26925E-05	2.13511E-05	Armatimonas_sp.	2.76419E-05	7.13711E-06	ASV_12862	0	0	Acidithiobacillaceae_KCM-B-112_sp.	0	0
Conexibacter_sp.	0.00014876	3.84097E-05	Acidothermus_sp.	0	0	ASV_6240	7.60767E-05	1.96429E-05	Babeliales_UBA12409_uncultured_bacterium	2.76419E-05	7.13711E-06	ASV_12880	0	0	Acidithiobacillus_ferrooxidans	0	0
Nocardioidaceae_sp.	0.00014876	3.84097E-05	Acidovorax_sp.	0	0	Spirosoma_rigui	7.60767E-05	1.96429E-05	Bdellovibrio_sp.	2.76419E-05	7.13711E-06	ASV_12965	0	0	Acidobacteria_bacterium	0	0
ASV_21853	0.000145148	3.74771E-05	Acinetobacter__metagenome	0	0	Antricoccus_suffuscus	7.50047E-05	1.93661E-05	Mycoplasma_uncultured_bacterium	2.76419E-05	7.13711E-06	ASV_12970	0	0	Acidobacteriaceae_(Subgroup_1)_sp.	0	0
ABY1_sp.	0.000144497	3.74311E-05	Acinetobacter_baumannii	0	0	Candidatus_Jidaibacter_metagenome	7.50047E-05	1.93661E-05	Salinirepens_sp.	2.76419E-05	7.13711E-06	ASV_13011	0	0	Acidobacteriae_Elev-16S-1166_sp.	0	0
Aerococcaceae_uncultured__bacterium	0.000144497	3.74311E-05	Acinetobacter_sp.	0	0	Kiritimatiellae_WCHB1-41_metagenome	7.50047E-05	1.93661E-05	Simkaniaceae_uncultured_Candidatus	2.76419E-05	7.13711E-06	ASV_13022	0	0	Acidobacteriae_Elev-16S-1166_uncultured_bacterium	0	0
ASV_19954	0.000144497	3.74311E-05	Acinetobacter_towneri	0	0	Saprospiraceae_uncultured_Haliscomenobacter_sp.	7.50047E-05	1.93661E-05	Sphingobacteriales_env.OPS_17_sp.	2.76419E-05	7.13711E-06	ASV_13091	0	0	Acidobacteriae_sp.	0	0
Spirochaeta_2_Spirochaeta_sp.	0.000144497	3.74311E-05	Acremonium_fuci	0	0	Butyricimonas_sp.	7.18556E-05	1.8553E-05	Candidatus_Magasanikbacteria_sp.	2.38158E-05	6.14921E-06	ASV_13184	0	0	Acidocella__uncultured_bacterium	0	0
Aenigmarcheota_sp.	0.000144389	3.71522E-05	Actinobacillus_sp.	0	0	Corynebacterium_glucuronolyticum	7.18556E-05	1.8553E-05	Bulleidia_extructa	2.17171E-05	5.60733E-06	ASV_13191	0	0	Acidothermus__uncultured_bacterium	0	0
Dokdonella_sp.	0.000144389	3.71522E-05	Actinobacteria_0319-7L14_uncultured_actinobacterium	0	0	ASV_12452	7.12883E-05	1.84066E-05	Slackia_exigua	2.17171E-05	5.60733E-06	ASV_13193	0	0	Acidothermus_sp.	0	0
ASV_21136	0.000126693	3.27732E-05	Actinobacteria_PeM15_bacterium_rJ7	0	0	Absconditabacteriales_(SR1)_candidate_division	7.01779E-05	1.81199E-05	ASV_4143	1.69345E-05	4.37247E-06	ASV_13313	0	0	Acinetobacter__metagenome	0	0
ASV_21137	0.000126693	3.27732E-05	Actinobacteria_PeM15_metagenome	0	0	Mogibacterium_uncultured_rumen	7.01779E-05	1.81199E-05	Actinomyces_graevenitzii	1.44781E-05	3.73823E-06	ASV_13359	0	0	Acinetobacter_kyonggiensis	0	0

Hydrogenophilaceae_uncultured__sp.	0.00012693	3.27732E-05	Actinobacteria_PeM15_sp.	0	0	Pir4_lineage_uncultured_Planctomycetaceae	7.01779E-05	1.81199E-05	Anaerococcusuncultured_organism	1.44781E-05	3.73823E-06	ASV_13391	0	0	Acremonium_fuci	0	0
Leptotrichia_wadei	0.00012693	3.27732E-05	Actinobacteria_PeM15_uncultured_soil	0	0	Saccharimonadales_LWQ8uncultured_Candidatus	7.01779E-05	1.81199E-05	ASV_1642	1.44781E-05	3.73823E-06	ASV_13403	0	0	Actinobacillus_sp.	0	0
Omnitrophales_uncultured_bacterium	0.00012693	3.27732E-05	Actinobacteria_sp.	0	0	Leptotrichia_uncultured_bacterium	6.95144E-05	1.79485E-05	MND1_sp.	1.44781E-05	3.73823E-06	ASV_13405	0	0	Actinobacteria_0319-7L14_uncultured_actinobacterium	0	0
Prevotella_uncultured_bacterium	0.00012693	3.27732E-05	Actinobacteria_uncultured__bacterium	0	0	Microscillaceae_OLB12uncultured_prokaryote	6.95144E-05	1.79485E-05	Pirellulaceae_uncultured_bacterium_enrichment	1.44781E-05	3.73823E-06	ASV_13432	0	0	Actinobacteria_PeM15_bacterium_rJ7	0	0
Sphingomonadaceae_sp.	0.00012693	3.27732E-05	Actinobacteria_uncultured__metagenome	0	0	Berkeleya_fennica	6.85918E-05	1.77103E-05	Sphingobacteriales_env.OPS_17_uncultured_bacterium	1.44781E-05	3.73823E-06	ASV_13441	0	0	Actinobacteria_PeM15_metagenome	0	0
Tsukamurella_paurometabola	0.00012693	3.27732E-05	Actinobacteria_uncultured__sp.	0	0	Candidatus_Omnitrophus_metagenome	6.85918E-05	1.77103E-05	Sphingomonas_sp.	1.44781E-05	3.73823E-06	ASV_13503	0	0	Actinobacteria_PeM15_sp.	0	0
ASV_3256	0.000118835	3.06831E-05	Actinobacteriota_MB-A2-108_metagenome	0	0	Marinimicrobia_(SAR406_clade)_metagenome	6.85918E-05	1.77103E-05	Hydrogenophilus_sp.	1.38209E-05	3.56854E-06	ASV_13525	0	0	Actinobacteria_PeM15_uncultured_soil	0	0
ASV_9544	0.000118835	3.06831E-05	Actinobacteriota_MB-A2-108_sp.	0	0	Oscillospirales_UCG-010_uncultured_bacterium	6.85918E-05	1.77103E-05	009E01-B-SD-P15__uncultured_bacterium	0	0	ASV_13612	0	0	Actinobacteria_PeM15uncultured_actinobacterium	0	0
Candidatus_Yanofskybacteria_sp.	0.000118835	3.06831E-05	Actinobacteriota_MB-A2-108_uncultured_bacterium	0	0	Telmatocola_sp.	6.85918E-05	1.77103E-05	1013-28-CG33__uncultured_soil	0	0	ASV_13680	0	0	Actinobacteria_sp.	0	0
Elusimicrobiota_Lineage_IIa_uncultured_bacterium	0.000118835	3.06831E-05	Actinomadura_sp.	0	0	Cyclobacteriaceae_uncultured_Flammeovirgaceae	6.75459E-05	1.74403E-05	Absconditabacteriales_(SR1)__metagenome	0	0	ASV_13728	0	0	Actinobacteria_uncultured__bacterium	0	0
Methanocorpusculum_uncultured_archaeon	0.000118835	3.06831E-05	Actinomyces__uncultured_bacterium	0	0	Fimbriiglobus_uncultured_planctomycete	6.75459E-05	1.74403E-05	Absconditabacteriales_(SR1)__uncultured_bacterium	0	0	ASV_13765	0	0	Actinobacteria_uncultured__metagenome	0	0
SAR324_clade(Marine_group_B)_sp.	0.000118835	3.06831E-05	Actinomyces_dentalis	0	0	Hyphomicrobium_metagenome	6.75459E-05	1.74403E-05	Absconditabacteriales_(SR1)_candidate_division	0	0	ASV_13773	0	0	Actinobacteria_uncultured__sp.	0	0
Actinobacteria_PeM15_sp.	0.000117486	3.03348E-05	Actinomyces_graevenitzii	0	0	Planctomycetota_OM190_sp.	6.65671E-05	1.71876E-05	Absconditabacteriales_(SR1)_SR1_bacterium	0	0	ASV_13775	0	0	Actinobacteria_uncultured_Actinomycetales_bacterium	0	0
Planctomycetes_uncultured_Planctomycetaceae	0.000117486	3.03348E-05	Actinomyces_israelii	0	0	Actinomyces_unidentified	0.000066154	1.70809E-05	Absconditabacteriales_(SR1)_uncultured_candidate	0	0	ASV_13785	0	0	Actinobacteriota_MB-A2-108_metagenome	0	0
Thermovirga_uncultured_bacterium	0.000117486	3.03348E-05	Actinomyces_sp.	0	0	Defluviitaleaceae_UCG-011_Lachnospiraceae_bacterium	0.000066154	1.70809E-05	Absconditabacteriales_(SR1)_uncultured_Epsilonproteobacteria	0	0	ASV_13794	0	0	Actinobacteriota_MB-A2-108_sp.	0	0
Beggiatoa_uncultured_prokaryote	0.000111157	2.88073E-05	Actinomyces_unidentified	0	0	Fibrobacterales_sp.	0.000066154	1.70809E-05	Absconditabacteriales_(SR1)_uncultured_prokaryote	0	0	ASV_13859	0	0	Actinobacteriota_MB-A2-108_uncultured_bacterium	0	0
KF-JG30-B3_metagenome	0.000111157	2.88073E-05	Actinomycetaceae_F0332_uncultured_bacterium	0	0	Fimbriimonadaceae_uncultured_bacterium	0.000066154	1.70809E-05	ABY1_sp.	0	0	ASV_13883	0	0	Actinobacteriota_MB-A2-108uncultured_actinobacterium	0	0
Bacilli_RF39_gut__metagenome	0.000108727	2.80732E-05	Actinomycetaceae_uncultured__bacterium	0	0	Phocaeicola_abscessus	6.48076E-05	1.67333E-05	Acanthopleuribacter__uncultured_bacterium	0	0	ASV_13900	0	0	Actinomadura_sp.	0	0
Chlamydiales_cvE6_uncultured_Chlamydia	0.000108727	2.80732E-05	Actinomycetaceae_uncultured__metagenome	0	0	ASV_4673	6.34994E-05	1.12293E-05	Acetitomaculum__uncultured_rumen	0	0	ASV_13923	0	0	Actinomyces__uncultured_bacterium	0	0
Pirellulaceae_uncultured__sp.	0.000108727	2.80732E-05	Actinomycetaceae_uncultured__rumen	0	0	Candidatus_Omnitrophus_sp.	6.29584E-05	1.62558E-05	Acetitomaculum_sp.	0	0	ASV_13925	0	0	Actinomyces_dentalis	0	0
ASV_21086	0.000107918	2.78643E-05	Actinomycetaceae_uncultured__sp.	0	0	Spirochaetaceae_uncultured_prokaryote	6.29584E-05	1.62558E-05	Acetivibrio_uncultured_bacterium	0	0	ASV_13930	0	0	Actinomyces_graevenitzii	0	0
Candidatus_Giovannonibacteria	0.000107918	2.78643E-05	Actinoplanes_garbadinensis	0	0	uncultured_Capnocytophaga	6.19038E-05	1.21774E-05	Acetoanaerobium_uncultured_bacterium	0	0	ASV_13977	0	0	Actinomyces_israelii	0	0
Silvanigrella_metagenome	0.000106309	2.74489E-05	Actinotalea_sp.	0	0	Alkalibacter_uncultured_bacterium	5.83268E-05	1.50599E-05	Acetobacter_sp.	0	0	ASV_14011	0	0	Actinomyces_unidentified	0	0
ASV_6176	9.79048E-05	2.52789E-05	Actinotignum_sp.	0	0	Schlegelella_sp.	5.83268E-05	1.50599E-05	Acetobacteraceae_sp.	0	0	ASV_14013	0	0	Actinomycetaceae_F0332_uncultured_bacterium	0	0
Emticicia_sediminis	9.79048E-05	2.52789E-05	Adhaeribacter__metagenome	0	0	Aquaspirillum_sp.	5.74845E-05	1.48424E-05	Acetobacteraceae_uncultured__bacterium	0	0	ASV_14099	0	0	Actinomycetaceae_F0332unidentified	0	0
Methylophilaceae_uncultured__metagenome	9.79048E-05	2.52789E-05	Adhaeribacter__uncultured_soil	0	0	Pseudarcicella_sp.	5.74845E-05	1.48424E-05	Acetobacteraceae_uncultured__metagenome	0	0	ASV_14118	0	0	Actinomycetaceae_uncultured__bacterium	0	0
Opitutus_metagenome	9.79048E-05	2.52789E-05	Adhaeribacter_sp.	0	0	Solirubrobacteraceae_uncultured_Colobanthus_quitensis	5.74845E-05	1.48424E-05	Acetobacteraceae_uncultured__sp.	0	0	ASV_14135	0	0	Actinomycetaceae_uncultured__rumen	0	0
Phycisphaera_metagenome	9.79048E-05	2.52789E-05	Advenella_sp.	0	0	Gaiellales_sp.	5.60749E-05	1.13928E-05	Acetobacterium_sp.	0	0	ASV_14140	0	0	Actinomycetaceae_uncultured__sp.	0	0
Cellvibrio_sp.	8.46203E-05	2.18489E-05	Aenigmarchaeales_sp.	0	0	Mitochondria_sp.	0.000055802	1.4408E-05	Acetothermiia__uncultured_bacterium	0	0	ASV_14196	0	0	Actinomycetospora_sp.	0	0
Psychrobacter_sanguinis	8.46203E-05	2.18489E-05	Aenigmarcheota_sp.	0	0	alpha_proteobacterium	5.26334E-05	1.35899E-05	Acholeplasma__uncultured_bacterium	0	0	ASV_14325	0	0	Actinoplanes_garbadinensis	0	0

Acidimicrobiia_IMCC26256uncultured_actinobacterium	7.83239E-05	2.02231E-05	Aequorivita_sp.	0	0	Anaerovoraceae_Family_XIII_AD3011_group_uncultured_rumen	5.26334E-05	1.35899E-05	Acholeplasma_axanthum	0	0	ASV_14332	0	0	Actinoplanes_sp.	0	0
Arcicella_rigui	7.83239E-05	2.02231E-05	Aerococcaceae_sp.	0	0	ASV_21256	5.26334E-05	1.35899E-05	Acholeplasma_brassicae	0	0	ASV_14376	0	0	Actinotalea_sp.	0	0
ASV_19296	7.83239E-05	2.02231E-05	Aerococcaceae_uncultured__bacterium	0	0	ASV_21257	5.26334E-05	1.35899E-05	Acholeplasma_laidlawii	0	0	ASV_14382	0	0	Actinotignum_sp.	0	0
Caulobacteraceae_uncultured_eubacterium	7.83239E-05	2.02231E-05	Aerococcus_sp.	0	0	ASV_21259	5.26334E-05	1.35899E-05	Acholeplasma_morum	0	0	ASV_144	0	0	Adhaeribacter__metagenome	0	0
Cyanobium_PCC-6307_Synechococcus-like_str.	7.83239E-05	2.02231E-05	Aeromicrobium_sp.	0	0	Saprospiraceae_uncultured__metagenome	5.26334E-05	1.35899E-05	Acholeplasma_sp.	0	0	ASV_14404	0	0	Adhaeribacter__uncultured_soil	0	0
Desulfatirhabdium_uncultured_bacterium	7.83239E-05	2.02231E-05	Aeromonas__metagenome	0	0	Gammaproteobacteria_CHAB-XI-27_uncultured_bacterium	5.21358E-05	1.34614E-05	Acholeplasmataceae_EMP-G18_uncultured_Mollicutes	0	0	ASV_14447	0	0	Adhaeribacter_sp.	0	0
Gemmataceae_uncultured__planctomycete	7.83239E-05	2.02231E-05	Aeromonas_sp.	0	0	Micropepsaceae_uncultured__metagenome	0.000051846	1.33866E-05	Acholeplasmauncultured_Acholeplasmataceae	0	0	ASV_14482	0	0	Advenella_sp.	0	0
Pectobacteriaceae_sp.	7.83239E-05	2.02231E-05	Aeromonas_uncultured_gamma	0	0	Peptostreptococcales-Tissierellales_uncultured_Peptostreptococcus	0.000051846	1.33866E-05	Achromatium_sp.	0	0	ASV_14494	0	0	Aenigmarchaeales_sp.	0	0
Acidimicrobiia_sp.	7.43799E-05	1.92048E-05	Aerosphaera__uncultured_bacterium	0	0	Planctomycetes_Pla4_lineage_metagenome	0.000051846	1.33866E-05	Acidaminobacter__uncultured_bacterium	0	0	ASV_14534	0	0	Aenigmarcheota_sp.	0	0
ASV_21892	7.43799E-05	1.92048E-05	Aerosphaera_sp.	0	0	ASV_20969	5.06594E-05	1.30802E-05	Acidaminococcaceae_uncultured__bacterium	0	0	ASV_14583	0	0	Aerococcaceae_sp.	0	0
Longimicrobiaceae_uncultured_soil	7.43799E-05	1.92048E-05	Afipia_genosp.	0	0	ASV_20970	5.06594E-05	1.30802E-05	Acidibacter__metagenome	0	0	ASV_14663	0	0	Aerococcaceae_uncultured__bacterium	0	0
Solirubrobacteraceae_uncultured_Phytoplasma_sp.	7.43799E-05	1.92048E-05	Afipia_sp.	0	0	Blastococcus_sp.	5.06594E-05	1.30802E-05	Acidibacter__uncultured_bacterium	0	0	ASV_14678	0	0	Aerococcus_sp.	0	0
Plantibacter_sp.	7.25742E-05	1.87386E-05	Agathobacter_sp.	0	0	Burkholderiales_sp.	5.06594E-05	1.30802E-05	Acidibacter_sp.	0	0	ASV_14706	0	0	Aeromicrobium_sp.	0	0
ZOR0006_sp.	7.24848E-05	1.87155E-05	Aggregatibacter_actinomycetemcomitans	0	0	Ekhidna_sp.	5.06594E-05	1.30802E-05	Acidimicrobiia_IMCC26256_metagenome	0	0	ASV_14729	0	0	Aeromonas__metagenome	0	0
Candidatus_Lloydbacteria_uncultured_bacterium	0.000071945	1.85761E-05	Aggregatibacter_sp.	0	0	Opitutaceae_uncultured__metagenome	5.06594E-05	1.30802E-05	Acidimicrobiia_IMCC26256_sp.	0	0	ASV_14736	0	0	Aeromonas_sp.	0	0
Eggerthellaceae_DMER64_Rikenellaceae_bacterium	0.000071945	1.85761E-05	Agitococcus_lubricus_group__uncultured_bacterium	0	0	ASV_2249	4.96155E-05	1.28107E-05	Acidimicrobiia_IMCC26256_uncultured_bacterium	0	0	ASV_14771	0	0	Aeromonas_uncultured_gamma	0	0
Fibrobacteraceae_uncultured__metagenome	0.000071945	1.85761E-05	Agitococcus_lubricus_group_sp.	0	0	ASV_2910	4.96155E-05	1.28107E-05	Acidimicrobiia_IMCC26256_uncultured_soil	0	0	ASV_14819	0	0	Aerosphaera__uncultured_bacterium	0	0
Neochlamydia_metagenome	0.000071945	1.85761E-05	Agrococcus_sp.	0	0	ASV_5084	4.96155E-05	1.28107E-05	Acidimicrobiia_IMCC26256uncultured_Aciditerrimonas	0	0	ASV_14825	0	0	Aerosphaera_sp.	0	0
Saprospiraceae_uncultured__sp.	0.000071945	1.85761E-05	Agromyces_sp.	0	0	Bdellovibrionaceae_OM27_clade_metagenome	4.96155E-05	1.28107E-05	Acidimicrobiia_IMCC26256uncultured_actinobacterium	0	0	ASV_14847	0	0	Aestuariicella__uncultured_bacterium	0	0
Lachnospiraceae_UCG-002_uncultured_rumen	5.87429E-05	1.51674E-05	Ahniella_affigens	0	0	Holdemanella_sp.	4.96155E-05	1.28107E-05	Acidimicrobiia_IMCC26256uncultured_Actinomycetales	0	0	ASV_14864	0	0	Afipia_genosp.	0	0
Phaselicystis_Myxococcales_bacterium	5.87429E-05	1.51674E-05	Ahniella_metagenome	0	0	Oligoflexia_0319-6G20__metagenome	4.96155E-05	1.28107E-05	Acidimicrobiia_sp.	0	0	ASV_14881	0	0	Afipia_sp.	0	0
Treponema_sp.	5.87429E-05	1.51674E-05	Ahniella_uncultured_bacterium	0	0	Porphyromonas_catoniae	4.96155E-05	1.28107E-05	Acidimicrobiia_uncultured__bacterium	0	0	ASV_14909	0	0	Agathobacter_sp.	0	0
ASV_21525	3.91619E-05	1.01116E-05	Akkermansia_uncultured_bacterium	0	0	uncultured_uncultured__sp.	4.96155E-05	1.28107E-05	Acidimicrobiia_uncultured__metagenome	0	0	ASV_14953	0	0	Aggregatibacter_actinomycetemcomitans	0	0
Candidatus_Nomurabacteria_uncultured_Parcubacteria	3.91619E-05	1.01116E-05	Alcaligenaceae_sp.	0	0	Gemmataceae_Zavarzinellauncultured_Zavarzinella	0.000047548	1.22768E-05	Acidimicrobiia_uncultured__sp.	0	0	ASV_14967	0	0	Aggregatibacter_sp.	0	0
Dojkabacteria_uncultured_bacterium	3.91619E-05	1.01116E-05	Alcaligenes_sp.	0	0	ASV_1837	4.57279E-05	1.18069E-05	Acidimicrobiia_uncultured_Acidimicrobiia	0	0	ASV_15004	0	0	Agitococcus_lubricus_group__uncultured_bacterium	0	0
Microbacterium_sp.	3.91619E-05	1.01116E-05	Algibacter_sp.	0	0	Saccharimonadales_uncultured_soil	4.57279E-05	1.18069E-05	Acidimicrobiia_uncultured_actinobacterium	0	0	ASV_15081	0	0	Agitococcus_lubricus_group_sp.	0	0
009E01-B-SD-P15__uncultured_bacterium	0	0	Algoriphagus_sp.	0	0	WOR-1_uncultured_bacterium	4.57279E-05	1.18069E-05	Acidimicrobiia_uncultured_Actinomycetales	0	0	ASV_15083	0	0	Agromyces_sp.	0	0
1013-28-CG33__uncultured_soil	0	0	Algoriphagus_uncultured_Hongiella	0	0	Acidimicrobiia_IMCC26256_sp.	4.53653E-05	1.17133E-05	Acidimicrobiia_uncultured_bacterium_YC-ZSS-LKJ199	0	0	ASV_15102	0	0	Ahniella_affigens	0	0
Abditibacterium_sp.	0	0	Aliidiomarina_uncultured_bacterium	0	0	Alishewanella_sp.	4.53653E-05	1.17133E-05	Acidiphilium__uncultured_bacterium	0	0	ASV_15165	0	0	Ahniella_metagenome	0	0
Absconditabacteriales_(SR1)__metagenome	0	0	Aliidongia_dinghuensis	0	0	Bacteroidales_RF16_group_uncultured_bacterium	4.53653E-05	1.17133E-05	Acidiphilium_sp.	0	0	ASV_15198	0	0	Ahniella_uncultured_bacterium	0	0

Absconditabacteriales_(SR1)__uncultured_bacterium	0	0	Aliivibrio_sp.	0	0	Pseudomonas_peli	4.53653E-05	1.17133E-05	Acidisoma_sp.	0	0	ASV_15233	0	0	Akkermansia_sp.	0	0
Absconditabacteriales_(SR1)_candidate_division	0	0	Alishewanella_sp.	0	0	ASV_3161	4.31134E-05	1.11318E-05	Acidithiobacillaceae_KCM-B-112_sp.	0	0	ASV_15253	0	0	Akkermansia_uncultured_bacterium	0	0
Absconditabacteriales_(SR1)_sp.	0	0	Alishewanella_uncultured_bacterium	0	0	Candidatus_Portnoybacteria_uncultured_deep-sea	4.31134E-05	1.11318E-05	Acidithiobacillus_ferrooxidans	0	0	ASV_15298	0	0	Alcaligenes_sp.	0	0
Absconditabacteriales_(SR1)_SR1_bacterium	0	0	Alistipes_gut__metagenome	0	0	Cloacimonadia_sp.	4.31134E-05	1.11318E-05	Acidobacteria_bacterium	0	0	ASV_15306	0	0	Algibacter_sp.	0	0
Absconditabacteriales_(SR1)_uncultured_candidate	0	0	Alistipes_sp.	0	0	Brevifollis_sp.	3.88845E-05	1.00399E-05	Acidobacteriaceae_(Subgroup_1)_sp.	0	0	ASV_15330	0	0	Algoriphagus_sp.	0	0
Absconditabacteriales_(SR1)_uncultured_Epsilonproteobacteria	0	0	Alistipes_uncultured_Alistipes	0	0	ASV_20078	3.80384E-05	9.82147E-06	Acidobacteriae_Elev-16S-1166_sp.	0	0	ASV_15347	0	0	Algoriphagus_uncultured_Bacteroidetes	0	0
Absconditabacteriales_(SR1)_uncultured_organism	0	0	Alistipes_uncultured_bacterium	0	0	Planctomycetota_BD7-11_uncultured_marine	3.80384E-05	9.82147E-06	Acidobacteriae_Elev-16S-1166_uncultured_bacterium	0	0	ASV_15366	0	0	Algoriphagus_uncultured_Hongiella	0	0
Absconditabacteriales_(SR1)_uncultured_prokaryote	0	0	Alistipes_uncultured_Bacteroidetes	0	0	SJA-15_uncultured_soil	3.80384E-05	9.82147E-06	Acidobacteriae_sp.	0	0	ASV_15373	0	0	Aliivibrio_sp.	0	0
Acanthopleuribacter__uncultured_bacterium	0	0	Aliterella_cyanobacterium_OU_20	0	0	ASV_21920	0.000035089	9.05994E-06	Acidocella__uncultured_bacterium	0	0	ASV_15451	0	0	Alishewanella_sp.	0	0
Acetitomaculum__uncultured_bacterium	0	0	Aliterella_sp.	0	0	Candidatus_Kuenenbacteria_uncultured_bacterium	0.000035089	9.05994E-06	Acidothermus__uncultured_bacterium	0	0	ASV_15470	0	0	Alishewanella_uncultured_bacterium	0	0
Acetitomaculum__uncultured_rumen	0	0	Aliterella_uncultured_bacterium	0	0	Alphaproteobacteria_sp.	3.47572E-05	8.97427E-06	Acidothermus_sp.	0	0	ASV_15543	0	0	Alistipes_gut__metagenome	0	0
Acetitomaculum_sp.	0	0	Alkalibacter_uncultured_bacterium	0	0	Neisseria_sp.	3.47572E-05	8.97427E-06	Acidovorax_sp.	0	0	ASV_15546	0	0	Alistipes_sp.	0	0
Acetivibrio_uncultured_bacterium	0	0	Alkalibacterium_uncultured_bacterium	0	0	Oxalobacteraceae_sp.	3.47572E-05	8.97427E-06	Acinetobacter__metagenome	0	0	ASV_15570	0	0	Alistipes_uncultured_Alistipes	0	0
Acetoanaerobium_uncultured_bacterium	0	0	Alkanibacter_metagenome	0	0	Pseudorhodobacter_sp.	3.47572E-05	8.97427E-06	Acinetobacter_baumannii	0	0	ASV_15576	0	0	Alistipes_uncultured_bacterium	0	0
Acetobacter_sp.	0	0	Alkanindiges_sp.	0	0	Ruminiclostridium_sp.	3.47572E-05	8.97427E-06	Acinetobacter_kyongiensis	0	0	ASV_15672	0	0	Alistipes_uncultured_Bacteroidetes	0	0
Acetobacteraceae_sp.	0	0	Alkanindiges_uncultured_bacterium	0	0	Turicella_uncultured_bacterium	3.47572E-05	8.97427E-06	Acinetobacter_sp.	0	0	ASV_15707	0	0	Aliterella_cyanobacterium_OU_20	0	0
Acetobacteraceae_uncultured__bacterium	0	0	Alkanindiges_uncultured_gamma	0	0	Actinobacteria_PeM15_bacterium_rJ7	3.37729E-05	8.72013E-06	Acinetobacter_towneri	0	0	ASV_1571	0	0	Aliterella_sp.	0	0
Acetobacteraceae_uncultured__metagenome	0	0	Allobranchiibius_sp.	0	0	Agathobacter_sp.	3.37729E-05	8.72013E-06	Actinobacillus_sp.	0	0	ASV_15723	0	0	Aliterella_uncultured_bacterium	0	0
Acetobacteraceae_uncultured__sp.	0	0	Alloiococcus_uncultured_bacterium	0	0	Bacilli_RF39_uncultured_Firmicutes	3.37729E-05	8.72013E-06	Actinobacteria_0319-7L14_uncultured_actinobacterium	0	0	ASV_15737	0	0	Alkalibacter_uncultured_bacterium	0	0
Acetobacterium_sp.	0	0	Alloprevotella_rava	0	0	Bacteroides_graminisolvens	3.37729E-05	8.72013E-06	Actinobacteria_PeM15_bacterium_rJ7	0	0	ASV_15770	0	0	Alkalibacterium_uncultured_bacterium	0	0
Acetothermiia__uncultured_bacterium	0	0	Alloprevotella_tanneriae	0	0	Chloroplast_Cinnamomum_camphora	3.37729E-05	8.72013E-06	Actinobacteria_PeM15_metagenome	0	0	ASV_15776	0	0	Alkanibacter_metagenome	0	0
Acholeplasma_axanthum	0	0	Alloprevotella_uncultured_Bacteroidales	0	0	Pirellula_metagenome	3.37729E-05	8.72013E-06	Actinobacteria_PeM15_sp.	0	0	ASV_15821	0	0	Alkanindiges_sp.	0	0
Acholeplasma_brassicae	0	0	Allorhizobium-Neorhizobium-Pararhizobium-Rhizobium_sp.	0	0	Roseimarinus_uncultured_bacterium	3.37729E-05	8.72013E-06	Actinobacteria_PeM15_uncultured_soil	0	0	ASV_15881	0	0	Alkanindiges_uncultured_bacterium	0	0
Acholeplasma_morum	0	0	Alloscardovia_omnicolens	0	0	Woesearchaeales_sp.	3.37729E-05	8.72013E-06	Actinobacteria_PeM15uncultured_actinobacterium	0	0	ASV_15912	0	0	Alkanindiges_uncultured_gamma	0	0
Acholeplasma_sp.	0	0	allotenuales_AKIW781_uncultured_bacterium	0	0	Xanthobacteraceae_sp.	3.37729E-05	8.72013E-06	Actinobacteria_uncultured__bacterium	0	0	ASV_1592	0	0	Allobranchiibius_sp.	0	0
Acholeplasmataceae_EMP-G18_uncultured_Mollicutes	0	0	alpha_proteobacterium	0	0	ASV_21519	0.000033077	8.54044E-06	Actinobacteria_uncultured__metagenome	0	0	ASV_15920	0	0	Alloiococcus_uncultured_bacterium	0	0
Acholeplasmauncultured_Acholeplasmataceae	0	0	Alphaproteobacteria_bacterium	0	0	Candidatus_Diapherotrites	0.000033077	8.54044E-06	Actinobacteria_uncultured__sp.	0	0	ASV_15928	0	0	Alloprevotella_rava	0	0
Achromatium_sp.	0	0	Alphaproteobacteria_sp.	0	0	Ferruginibacter_uncultured_soil	0.000033077	8.54044E-06	Actinobacteria_uncultured_Actinomycetales_bacterium	0	0	ASV_15996	0	0	Alloprevotella_sp.	0	0
Acidaminobacter__uncultured_bacterium	0	0	Alphaproteobacteria_uncultured__metagenome	0	0	Finegoldia_uncultured_bacterium	0.000033077	8.54044E-06	Actinobacteriota_MB-A2-108_metagenome	0	0	ASV_16023	0	0	Alloprevotella_tanneriae	0	0
Acidaminococcaceae_uncultured__bacterium	0	0	Alphaproteobacteria_uncultured_bacterium_enrichment	0	0	Gemmatimonadaceae_uncultured__metagenome	0.000033077	8.54044E-06	Actinobacteriota_MB-A2-108_sp.	0	0	ASV_16127	0	0	Allorhizobium-Neorhizobium-Pararhizobium-Rhizobium_sp.	0	0

Acidibacter__metagenome	0	0	Alphaproteobacteria_uncultured_eubacterium	0	0	Anaerosinus_uncultured_bacterium	3.24038E-05	8.36663E-06	Actinobacteriota_MB-A2-108_uncultured_bacterium	0	0	ASV_16215	0	0	Alloscardovia_omnicolens	0	0
Acidibacter__uncultured_bacterium	0	0	Alphaproteobacteria_uncultured_gut__metagenome	0	0	Burkholderiales_B1-7BS_metagenome	3.24038E-05	8.36663E-06	Actinobacteriota_MB-A2-108uncultured_actinobacterium	0	0	ASV_16250	0	0	allotenuales_AKIW781_uncultured_bacterium	0	0
Acidibacter_sp.	0	0	Alphaproteobacteria_uncultured_Novosphingobium	0	0	Massilia_sp.	3.24038E-05	8.36663E-06	Actinomyces__uncultured_bacterium	0	0	ASV_16300	0	0	alpha_proteobacterium	0	0
Acidimicrobiia_IMCC26256_metagenome	0	0	Alphaproteobacteria_uncultured_Rhodospirillaceae_bacterium	0	0	Sporichthyaceae_hgcl_clade_metagenome	3.24038E-05	8.36663E-06	Actinomyces_sp.	0	0	ASV_16302	0	0	Alphaproteobacteria_bacterium	0	0
Acidimicrobiia_IMCC26256_sp.	0	0	Altererythrobacter_alpha_proteobacterium	0	0	Bdellovibrio_uncultured_bacterium	2.87422E-05	7.4212E-06	Actinomyces_unidentified	0	0	ASV_16316	0	0	Alphaproteobacteria_sp.	0	0
Acidimicrobiia_IMCC26256_uncultured_bacterium	0	0	Alysiella_sp.	0	0	Tepidimonas_sp.	2.87422E-05	7.4212E-06	Actinomycetaceae_F0332_uncultured_bacterium	0	0	ASV_16374	0	0	alphaproteobacteria_uncultured__bacterium	0	0
Acidimicrobiia_IMCC26256_uncultured_soil	0	0	Alysiella_uncultured_bacterium	0	0	Alkanindiges_sp.	2.85288E-05	7.3661E-06	Actinomycetaceae_uncultured__metagenome	0	0	ASV_16382	0	0	Alphaproteobacteria_uncultured__bacterium	0	0
Acidimicrobiia_IMCC26256uncultured_Aciditerrimonas	0	0	Amaricoccus_sp.	0	0	Arenimonas_sp.	2.85288E-05	7.3661E-06	Actinomycetaceae_uncultured__rumen	0	0	ASV_16398	0	0	Alphaproteobacteria_uncultured__metagenome	0	0
Acidimicrobiia_IMCC26256uncultured_Actinomycetales	0	0	Amaricoccus_tamworthensis	0	0	ASV_21166	2.85288E-05	7.3661E-06	Actinomycetaceae_uncultured__sp.	0	0	ASV_164	0	0	Alphaproteobacteria_uncultured__sp.	0	0
Acidimicrobiia_uncultured__bacterium	0	0	Amb-16S-1323_uncultured_bacterium	0	0	Desulfobacterota_uncultured_Desulfuromonadales	2.85288E-05	7.3661E-06	Actinomycetospora_sp.	0	0	ASV_1642	0	0	Alphaproteobacteria_uncultured_Alphaproteobacteria	0	0
Acidimicrobiia_uncultured__metagenome	0	0	Aminobacter_sp.	0	0	Oligoflexia_0319-6G20_uncultured_prokaryote	2.85288E-05	7.3661E-06	Actinoplanes_garbadinensis	0	0	ASV_16447	0	0	Alphaproteobacteria_uncultured_bacterium_enrichment	0	0
Acidimicrobiia_uncultured__sp.	0	0	Ammoniibacillus_agariperforans	0	0	ASV_20020	0.000025923	6.69329E-06	Actinoplanes_sp.	0	0	ASV_16467	0	0	Alphaproteobacteria_uncultured_eubacterium	0	0
Acidimicrobiia_uncultured_Acidimicrobiia	0	0	Amnibacterium_sp.	0	0	ASV_20022	0.000025923	6.69329E-06	Actinotalea_sp.	0	0	ASV_16643	0	0	Alphaproteobacteria_uncultured_gut__metagenome	0	0
Acidimicrobiia_uncultured_actinobacterium	0	0	Amnipila_sp.	0	0	Acidimicrobiia_uncultured__sp.	1.94423E-05	5.01998E-06	Actinotignum_sp.	0	0	ASV_16667	0	0	Alphaproteobacteria_uncultured_Lacibacterium_aquatile	0	0
Acidimicrobiia_uncultured_Actinomycetales	0	0	Amycolatopsis_sp.	0	0	Arcicella_sp.	1.94423E-05	5.01998E-06	Adhaeribacter__metagenome	0	0	ASV_16713	0	0	Alphaproteobacteria_uncultured_Novosphingobium	0	0
Acidimicrobiia_uncultured_bacterium_YC-ZSS-LKJ199	0	0	Anaerobacterium_uncultured_bacterium	0	0	ASV_21115	1.94423E-05	5.01998E-06	Adhaeribacter__uncultured_soil	0	0	ASV_16736	0	0	Alphaproteobacteria_uncultured_Rhodospirillaceae_bacterium	0	0
Acidiphilium__uncultured_bacterium	0	0	Anaerococcus_octavius	0	0	Chlamydiales_sp.	1.94423E-05	5.01998E-06	Adhaeribacter_sp.	0	0	ASV_16822	0	0	Altererythrobacter_alpha_proteobacterium	0	0
Acidiphilium_sp.	0	0	Anaerococcus_prevotii	0	0	Sulfuricurvum_sp.	1.94423E-05	5.01998E-06	Aenigmarchaeales_sp.	0	0	ASV_16866	0	0	Altererythrobacter_sp.	0	0
Acidisoma_sp.	0	0	Anaerococcus_sp.	0	0	Woesearchaeales_GW2011_GWC1_47_15_uncultured_archaeon	1.90192E-05	4.91074E-06	Aenigmarcheota_sp.	0	0	ASV_16876	0	0	Alysiella_sp.	0	0
Acidithiobacillaceae_KCM-B-112_sp.	0	0	Anaerococcus_uncultured_bacterium	0	0	ASV_21851	1.29615E-05	3.34664E-06	Aequorivita_sp.	0	0	ASV_16882	0	0	Alysiella_uncultured_bacterium	0	0
Acidithiobacillus_ferrooxidans	0	0	Anaerococcusuncultured_organism	0	0	Gammaproteobacteria_uncultured__bacterium	1.29615E-05	3.34664E-06	Aerococcaceae_sp.	0	0	ASV_16940	0	0	Amaricoccus_sp.	0	0
Acidobacteria_bacterium	0	0	Anaerocolumna_uncultured_bacterium	0	0	ASV_2180	6.48076E-06	1.67333E-06	Aerococcaceae_uncultured__bacterium	0	0	ASV_16946	0	0	Amaricoccus_tamworthensis	0	0

Implant																	
Assistant			Environment			Operator			Patient			Saliva			Waterline		
Taxon ID	Mean relative abundance	Std dev	Taxon ID	Mean relative abundance	Std dev	Taxon ID	Mean relative abundance	Std dev	Taxon ID	Mean relative abundance	Std dev	Taxon ID	Mean relative abundance	Std dev	Taxon ID	Mean relative abundance	Std dev
Uncultured_Vulcaniibacterium	0.49812895	0.03228121	Uncultured_Vulcanibacterium	0.512054355	0.031452708	Uncultured_Vulcanibacterium	0.378010085	0.033080948	Uncultured_Vulcanibacterium	0.467936176	0.033208517	Rothia_aeria	0.025421663	0.003100451	Uncultured_Vulcanibacterium	0.327631228	0.030468353
Uncultured_Vulcanibacterium	0.41615963	0.02573199	Uncultured_Vulcanibacterium	0.467976932	0.026920999	Uncultured_Vulcanibacterium	0.346678323	0.030424246	Uncultured_Vulcanibacterium	0.4012571	0.023581677	Prevotella_nigrescens	0.020610782	0.005789665	Uncultured_Vulcanibacterium	0.301208887	0.029697561
Schlegelella_uncultured_bacterium	0.08521363	0.00000000	Schlegelella_uncultured_bacterium	0.0495545	0	Schlegelella_uncultured_bacterium	0.046210715	0.009581533	Schlegelella_uncultured_bacterium	0.01692113	0.002175298	Solobacterium_moorei	0.009923831	0.001193183	ASV_363	0.030733877	0.006135132
Cellvibrio_uncultured_Cellvibrio	0.00629149	0.00076412	Rhodococcus_erythropolis	0.007159086	0	Rhodococcus_erythropolis	0.010407384	0.001162855	Rhodococcus_erythropolis	0.016248868	0.001674334	Butyrivibrio_Eubacterium_sp.	0.00771154	0.002004141	ASV_366	0.030232865	0.005669934

Rhodococcus_erythropolis	0.00538521	0.00000000	ASV_363	0.005384657	0.000860717	Methylobacterium_organophilum	0.008599509	0.002719404	Cellvibrio_uncultured_Cellvibrio	0.005035573	0.000574775	ASV_292	0.004822044	0.000831838	Schlegelella_uncultured_bacterium	0.02641167	0.004956564
ASV_720	0.00516711	0.00082012	Brevundimonas_diminuta	0.005098239	0.000953258	Solobacterium_moorei	0.008100413	0.001922327	Saccharimonadalesuncultured_organism	0.004516243	0.001133779	ASV_256	0.004636821	0.000811774	Brevundimonas_diminuta	0.005919731	0.000571455
ASV_709	0.00464152	0.00071324	Acetobacteraceae_uncultured_alpha_proteobacterium	0.004833691	0.001158501	ASV_709	0.006464128	0.001262197	ASV_1161	0.003931948	0.000770518	Leptotrichia_wadei	0.004109339	0.001113082	Obscuribacteraceae_sp.	0.005605344	0.001772565
ASV_2465	0.00371040	0.00117333	ASV_1161	0.004351085	0.000583716	ASV_720	0.005493932	0.000683843	Rubellimicrobiumuncultured_Alphaproteobacteria	0.003881262	0.001227363	ASV_2163	0.003217305	0.001017401	Rhodococcus_erythropolis	0.004846375	0.000545104
ASV_2084	0.00370294	0.00117097	ASV_366	0.004324562	0.000862239	ASV_20325	0.004914005	0.001553945	Brevundimonas_diminuta	0.003563123	0.000375844	ASV_1976	0.003215985	0.000862262	ASV_211	0.00456333	0.001408543
Brevundimonas_diminuta	0.00353284	0.00055688	Blattabacterium_uncultured_bacterium	0.004145507	0.001310924	ASV_292	0.004722487	0.000607167	Rothia_aeria	0.003556673	0.000912963	Fusobacterium_necrophorum	0.003096403	0.000979169	Denitratisoma_sp.	0.004397006	0.001390455
Acidimicrobiia_uncultured_Acidimicrobiia	0.00313957	0.00099282	ASV_720	0.004108376	0.00077794	ASV_256	0.004320128	0.000539402	ASV_1208	0.003484187	0.000798907	ASV_2250	0.002442749	0.00071024	ASV_720	0.003720732	0.000509649
uncultured_Anaeromyxobacter	0.00292180	0.00000000	ASV_709	0.003801775	0.000714603	ASV_366	0.004207108	0.000604302	Acetobacteraceae_uncultured_alpha_proteobacterium	0.003449648	0.000815572	ASV_2802	0.002190506	0.000692699	Paeniclostridium_sp.	0.00036056	0.000794241
Lactobacillus_delbrueckii	0.00267435	0.00084570	Pseudoxanthomonas_taiwanensis	0.003747118	0	ASV_363	0.004058643	0.000631299	ASV_4615	0.003197223	0.001011051	Prevotella_intermedia	0.002011527	0.000636101	ASV_709	0.003451548	0.000463753
Polyangium_sp.	0.00263012	0.00083172	ASV_4077	0.003660657	0.001157601	Diplorickettsiaceae_uncultured__sp.	0.003685504	0.001165459	Sphingomonas_crusticola	0.002882096	0.000911399	Streptococcus_anginosus	0.002007842	0.000589155	Solobacterium_moorei	0.003392111	0.000570692
Rhodanobacteraceae_uncultured__sp.	0.00245538	0.00000000	Brevundimonas_alba	0.003530796	0.001116536	Patescibacteria_CPR2_uncultured_bacterium	0.003685504	0.001165459	ASV_720	0.002668125	0.000475709	ASV_3085	0.001916692	0.000606111	ASV_621	0.002668412	0.000843826
Corynebacterium_aurimucosum	0.00245532	0.00057799	Clostridia_UCG-014_unidentified	0.003530796	0.001116536	Rickettsia_sp.	0.003685504	0.001165459	ASV_2692	0.002466429	0.000779953	Treponema_lecithinolyticum	0.001812133	0.000333698	Desulfomonile_uncultured_delta	0.002598608	0.000821752
Psychrobacter_sanguinis	0.00238745	0.00000000	ASV_1642	0.003323421	0.000867129	Thermobacillus_sp.	0.003685504	0.001165459	ASV_2103	0.002206027	0.000449207	Peptococcus_sp.	0.001781917	0.00022153	ASV_1161	0.00256164	0.000342181
Oligoflexales_uncultured__soil	0.00235211	0.00031804	ASV_292	0.002492026	0.000788048	Brevundimonas_diminuta	0.003521397	0.000523738	ASV_6343	0.002183406	0.000690454	ASV_305	0.001741727	0.000550782	ASV_4322	0.002509001	0.000769397
Rarobacter_faecitabidus	0.00230218	0.00000000	ASV_8009	0.002265679	0.000679382	Parcubacteria_group	0.003250271	0.001027826	Desulfovermiculus_sp.	0.002183406	0.000690454	Anaerovoracaceae_Family_XIII_UCG-001_sp.	0.001726315	0.000321514	Chloroplast_Melosira_varians	0.002314061	0.000704924
ASV_5051	0.00221484	0.00070039	Scytonema_UTEX_2349_sp.	0.002228478	0	Actinobacteria_uncultured__metagenome	0.003033586	0.000959304	ASV_709	0.002147063	0.000224165	Alloscardovia_omnicolens	0.001496409	0.000291357	Cloacibacterium_sp.	0.00226535	0.000676098
Chryseobacterium_gregarium	0.00221435	0.00070024	ASV_5961	0.002192982	0.000693482	Negativibacillus_uncultured_bacterium	0.00256998	0.000812699	Lawsonella_uncultured_bacterium	0.002106836	0.000444251	Anaerovoracaceae_Family_XIII_UCG-002_sp.	0.001495767	0.000234925	Dysgonomonadaceae_uncultured__bacterium	0.002148074	0.000679281
ASV_6823	0.00218818	0.00069196	ASV_6381	0.002163378	0.00068412	ASV_21995	0.002457002	0.000776972	Megamonasuncultured_organism	0.00209607	0.000662836	Lactobacillus_kalixensis	0.001471746	0.000465407	Rothia_aeria	0.00203784	0.000338722
ASV_6826	0.00218818	0.00069196	Lawsonella_uncultured_bacterium	0.002037239	0.00044067	Deinococcus_antarcticus	0.002457002	0.000776972	ASV_366	0.002086301	0.000414487	Prevotella_baroniae	0.001341713	0.000336989	ASV_7836	0.00203314	0.000642935
Blastopirellula_sp.	0.00218818	0.00069196	Chthoniobacter_metagenome	0.00199362	0.000630438	Parcubacteria_sp.	0.002457002	0.000776972	Chloroplast_Cinnamomum_camphora	0.002009683	0.000635518	Mycoplasmataceae_genomosp.	0.001299383	0.000410901	ASV_10253	0.002004869	0.000633995
Alphaproteobacteria_uncultured_eubacterium	0.00212578	0.00067223	Cellvibrio_uncultured_Cellvibrio	0.001959465	0.000373649	ASV_1161	0.002258912	0.000573072	Lactobacillus_senioris	0.002009683	0.000635518	Actinomycetaceae_F0332unidentified	0.001286818	0.00028276	Desulfocapsaceae_sp.	0.002004869	0.000633995
Fibrobacteraceae_uncultured_Juncus_effusus	0.00209937	0.00064233	ASV_8569	0.001865478	0.000589916	ASV_5337	0.002166847	0.000685217	uncultured_Chloroflexi	0.001940631	0.000613681	Leptotrichia_buccalis	0.001240346	0.000392232	Kaistia_uncultured_bacterium	0.002004869	0.000633995
NKB15_uncultured_bacterium	0.00209305	0.00061868	Paeniclostridium_sp.	0.001865478	0.000589916	Bacteroidales_sp.	0.002094619	0.000662377	ASV_363	0.001927297	0.000421795	ASV_346	0.001230267	0.000389045	Pedosphaeraceae_SH3-11uncultured_verrucomicrobium	0.001861664	0.00058871
Hypnocyclicus_uncultured_bacterium	0.00203720	0.00064422	alphaproteobacteria_uncultured__bacterium	0.001730703	0.000547296	SBR1031_uncultured_Gemmatimonadetes	0.002094619	0.000662377	ASV_3228	0.001826985	0.000577743	Corynebacterium_durum	0.001065907	0.000139555	Pseudoxanthomonas_Xanthomonas_sp.	0.001829826	0.000578642
Diplorickettsia_uncultured_bacterium	0.00200802	0.00053921	ASV_1760	0.001701542	0.000412134	Prevotellaceae_UCG-004uncultured_Bacteroidales	0.002022391	0.000639536	ASV_7992	0.001786554	0.000564958	Treponema_denticola	0.001006487	0.000191451	ASV_292	0.001819896	0.000362403
Oligella_sp.	0.00190277	0.00060171	Coprococcus_metagenome	0.001647296	0.000520921	Fodinicurvataceae_uncultured_Rhodospirillaceae	0.002018897	0.000638431	ASV_8075	0.001735635	0.000548856	Selenomonas_Veillonellaceae_bacterium	0.000992465	0.000313845	Simkaniaceae_uncultured_Candidatus	0.001812506	0.000573165
Brevibacillus_sp.	0.00188875	0.00059728	Clostridium_sensu_stricto_1_human_gut	0.001644168	0.000519932	Oxalobacteraceae_CM1G08_sp.	0.001997302	0.000615218	bacterium	0.001581255	0.000500037	Ottowia_sp.	0.000836253	0.000264446	Oligoflexales_uncultured__soil	0.001767519	0.000309804
Halomonas_uncultured_soil	0.00186328	0.00048147	Chelatococcus_sp.	0.001633359	0.000516513	ASV_3419	0.001978519	0.000625663	Anaerolineaceae_uncultured_Bellilinea	0.001574338	0.000497849	Enterococcus_faecium	0.00082144	0.000259762	Salinicola_sp.	0.001763341	0.000557617
Lawsonella_sp.	0.00185147	0.00058549	Gemmatimonas_phototrophica	0.001495215	0.000472828	Exiguobacterium_sp.	0.001972252	0.000623681	Candidatus_Nitrotoga_sp.	0.001572052	0.000497126	Streptococcus_mutans	0.000749906	0.000207987	Edaphobaculum_uncultured_Chitinophagaceae	0.001762159	0.000557244
Paenibacillaceae_uncultured__bacterium	0.00180763	0.00057162	MND1uncultured_Burkholderiales	0.001471097	0.000465202	Cloacibacterium_sp.	0.001949855	0.000616598	Methylomonadaceae_sp.	0.001475942	0.000466734	Lachnoanaerobaculum_uncultured_bacterium	0.000735159	0.000232478	ASV_144	0.001678247	0.000530708

Rhodobacter_uncultured_bacterium	0.00170532	0.00000000	Sphingomonadaceae_Ellin6055_sp.	0.001395534	0	NB1-j_sp.	0.001805932	0.000571086	Globicatella_uncultured_bacterium	0.001437504	0.000454579	Porphyromonas_catoniae	0.00056537	0.000119306	ASV_9468	0.001626512	0.000514348
ASV_8087	0.00168291	0.00053218	Candidatus_Wolfebacteria_uncultured_Microgenomates	0.001373087	0.000434208	ASV_6329	0.001805706	0.000571014	TG3_uncultured_Chitinivibrionia	0.001410437	0.000446019	Neisseria_oralis	0.000551369	0.000174358	Butyrivibrio_Eubacterium_sp.	0.001609665	0.000509021
Blastocatellaceae_uncultured__metagenome	0.00162006	0.00051231	Hydrogenispora_uncultured_bacterium	0.001373087	0.000434208	ASV_2390	0.00174009	0.000499446	Colidextribacter_uncultured_rumen	0.001377546	0.000435618	Anaerovoracaceae_Family_XIII_UCG-004Eubacterium_sp.	0.000530249	8.71093E-05	Acetobacteraceae_uncultured_alpha_proteobacterium	0.001513459	0.000239637
Oceanobacter_uncultured_bacterium	0.00162006	0.00051231	ASV_3161	0.001372746	0.00043441	Staphylococcus_pettenkoferi	0.001737291	0.00054938	Ohtaekwangia_uncultured_Bacteroidetes	0.001377546	0.000435618	Anaeroglobus_uncultured_organism	0.000477853	0.00015111	Singulisphaera_Isosphaera_sp.	0.001510422	0.000477637
Rufibacter_sp.	0.00161735	0.00000000	ASV_2285	0.001287204	0.00040705	Marinagarivorans_gamma_proteobacterium	0.001736473	0.000549121	Pedosphaeraceae_ADurb.Bin063-1_sp.	0.001377546	0.000435618	Bulleidia_extructa	0.000461903	0.000101315	Mucilaginibacter_myungsuensis	0.001486989	0.000470227
Paenibacillus_rigui	0.00159192	0.00050341	ASV_3021	0.00128123	0.000405161	Gottschalkia_uncultured_bacterium	0.001733478	0.000548174	VHS-B3-70_sp.	0.00127915	0.000404503	Prevotella_micans	0.000438967	9.25437E-05	Hydrogenispora_uncultured_Firmicutes	0.001432049	0.000452854
Cyclobacteriaceae_uncultured__bacterium	0.00157786	0.00039399	ASV_10821	0.001249039	0.000394981	ASV_1208	0.00171256	0.000350598	Caulobacter_metagenome	0.001222707	0.000386654	Anaerovoracaceae_Family_XIII_UCG-003_sp.	0.000429987	0.000135974	Methylomirabilaceae_MIZ17_uncultured_bacterium	0.001423198	0.000450055
Megamonasuncultured_organism	0.00153852	0.00026919	Diplorickettsiaceae_uncultured_Legionellales	0.001211492	0.000383107	Candidatus_Ovatusbacter_uncultured_gamma	0.001686056	0.000533178	Rhodoluna_uncultured_actinobacterium	0.001222707	0.000386654	Clostridia_vadinBB60_group_Clostridiales_bacterium	0.000400562	9.4506E-05	Alkalibacterium_uncultured_bacterium	0.001392111	0.000440224
ASV_8378	0.00153479	0.00048534	Clostridium_sensu_stricto_12_sp.	0.001176932	0.000372179	Hydrocarboniphaga_sp.	0.00166125	0.000525333	ASV_10814	0.001222379	0.00038655	Paludibacteraceae_F0058_uncultured_bacterium	0.00039224	0.000124037	Shinella_sp.	0.001332876	0.000322561
Cyclobacteriaceae_uncultured_uncultured_Sphingobacteriales	0.00152222	0.00048137	Comamonas_sp.	0.001176932	0.000372179	Isosphaeraceae_uncultured_Isosphaera_sp.	0.001655495	0.000523513	Gemmatimonas_uncultured_bacterium	0.001222379	0.00038655	Cryptobacterium_sp.	0.000389466	5.65254E-05	Oscillospiraceae_uncultured__rumen	0.001288844	0.000407568
Acetobacteraceae_uncultured_alpha_proteobacterium	0.00151501	0.00032088	Finegoldia_uncultured_bacterium	0.001152959	0.000364598	Rothia_aeria	0.001592058	0.000343264	ASV_3531	0.00118754	0.000375533	Metamycoplasma_salivarium	0.000362067	0.000114496	ASV_4353	0.001265995	0.000350115
ASV_1208	0.00145642	0.00030310	Gammaproteobacteria_JTB23_metagenome	0.001152959	0.000364598	ASV_4250	0.001534362	0.000485208	Bacteroidales_p-251-o5_uncultured_bacterium	0.001180754	0.000373387	Bacilli_RF39_Firmicutes_oral	0.000359404	0.000113654	ASV_256	0.001222409	0.000291462
Rubinisphaeraceae_SH-PL14__uncultured_planctomycete	0.00143308	0.00000000	Clostridioides_sp.	0.001096491	0.000346741	Aquisphaera_uncultured_bacterium	0.001517451	0.00047986	ASV_2390	0.001150185	0.000245625	Actinomycetaceae_F0332_uncultured_bacterium	0.000358186	8.10131E-05	Oscillospiraceae_UCG-003_sp.	0.001206497	0.000381528
Agromyces_sp.	0.00142708	0.00045128	Sphaerotilus_natans	0.001096491	0	Hungateiclostridiaceae_HN-HF0106_uncultured_bacterium	0.001517451	0.00047986	ASV_11002	0.001135371	0.000359036	Porphyromonas_gingivalis	0.000356817	0.000112835	Actinobacillus_sp.	0.001150562	0.00036384
ASV_9779	0.00142708	0.00045128	Streptococcus_mutans	0.001096491	0	ASV_3544	0.001516793	0.000479652	Rhizobiales_Incertae_Sedis_uncultured__metagenome	0.001135371	0.000359036	Eggerthia_catenaformis	0.000345581	0.000109282	ASV_164	0.001141208	0.000360882
ASV_9780	0.00142708	0.00045128	Vicinamibacteria_uncultured_Acidobacteriales	0.001096491	0	Uliginosibacterium_uncultured_bacterium	0.001453606	0.000459671	ASV_3716	0.001097613	0.000347096	Lachnoanaerobaculum_sp.	0.000330822	0.000104615	ASV_3349	0.001113689	0.000352179
Roseococcus_sp.	0.00141656	0.00002176	Beijerinckiaceae_FukuN57_uncultured_bacterium	0.001056879	0.000334214	ASV_2510	0.001444565	0.000456812	Anaerolineaceae_sp.	0.001048035	0.000331418	Campylobacter_concisus	0.000303238	7.17122E-05	ASV_5066	0.001103111	0.000319586
ASV_5386	0.00136765	0.00043249	FCPU426uncultured_microorganism	0.001056879	0.000334214	Coriobacteriia_OPB41_uncultured_bacterium	0.001444565	0.000456812	ASV_3568	0.001048035	0.000331418	Absconditabacteriales_(SR1)_SR1_bacterium	0.000293819	9.29137E-05	Anaerolineaceae_uncultured_eubacterium	0.001073936	0.000339608
Aurantisolimonas_Terrimonas_sp.	0.00136765	0.00043249	Solobacterium_moorei	0.00100479	0	Frankiales_sp.	0.001444565	0.000456812	Limnobacter_sp.	0.001034321	0.000327081	ASV_366	0.000271328	6.10319E-05	Babeliales_UBA12409_metagenome	0.001066139	0.000337143
Pseudomonas_bioreactor__metagenome	0.00133717	0.00000000	ASV_8590	0.000980777	0.000310149	Lawsonella_uncultured_bacterium	0.001434649	0.000314488	Saprospiraceae_OLB8_sp.	0.001034321	0.000327081	Prevotella_maculosa	0.000271217	8.57663E-05	Amb-16S-1323_uncultured_bacterium	0.001042729	0.000221161
Desemzia_sp.	0.00133194	0.00042120	Kribbella_sp.	0.000980777	0.000310149	Rhodospirillales_uncultured_Rhodospirillales	0.001428183	0.000451631	Sporichthyaceae_hgcl_clade_marine__metagenome	0.001034321	0.000327081	Amnipila_sp.	0.000251885	7.9653E-05	Kocuria_marina	0.001036323	0.000174507
Klenkia_sp.	0.00132861	0.00042014	Sphingomonas_groundwater__metagenome	0.000980777	0	Staphylococcus_vitulinus	0.00137285	0.000434133	Bacteroidetes_BD2-2_uncultured_organism	0.001006253	0.000318205	Aggregatibacter_actinomycetemcomitans	0.000248818	7.86832E-05	Propionibacteriaceae_uncultured_Actinomycetales	0.001020882	0.000322831
Corynebacterium_amycolatum	0.00130839	0.00024317	Candidatus_Kerfeldbacteria_Parcubacteria_group	0.000951886	0.000301013	Gemmataceae_uncultured_Planctomycetaceae	0.001348845	0.000426542	ASV_1837	0.000970991	0.000266132	ASV_6	0.000234371	6.93938E-05	ASV_13503	0.00101657	0.000321468
Actinotignum_sp.	0.00127899	0.00040445	ASV_1208	0.00094154	0.000192577	Oligoflexia_0319-6G20_uncultured_delta	0.001348845	0.000426542	SphingobiumSphingobium_sufflavum	0.000960699	0.00030038	Tannerella_forsythia	0.000229722	5.07486E-05	Acidothermus__uncultured_bacterium	0.001009265	0.000319158
Clostridium_sensu_stricto_1_uncultured_Clostridia	0.00127899	0.00040445	ASV_14447	0.000932739	0.000294958	Megamonasuncultured_organism	0.00129651	0.000386688	Blastocatellaceae_uncultured_Acidobacteria	0.000947938	0.000299764	Cardiobacterium_uncultured_bacterium	0.000220658	6.97782E-05	Leeia_uncultured_bacterium	0.001002434	0.000316997
Sphingobacterium_uncultured_bacterium	0.00124004	0.00000000	Dongia_rigui	0.000915164	0.00028894	ASV_3560	0.001292094	0.000408596	Anaerolineaceae_uncultured_sludge	0.000940291	0.000297346	Anaerovoracaceae_Family_XIII_UCG-005Eubacterium_sp.	0.00020977	4.43888E-05	Nitrosomonadaceae_IS-44_sp.	0.001002434	0.000316997
ASV_2303	0.00123233	0.00027345	Tepidimonas_sp.	0.000912117	0	ASV_5010	0.001292094	0.000408596	Pseudorhodobacter_metagenome	0.000940291	0.000297346	Prevotella_pleuritidis	0.00020536	6.49405E-05	Treponema_uncultured_bacterium	0.001002434	0.000316997
Spirosoma_arcticum	0.00119372	0.00000000	Pir4_lineage_sp.	0.000865351	0.000273648	ASV_1592	0.001285646	0.000371003	Neisseria_uncultured_bacterium	0.000919319	0.000290714	Murdochiella_uncultured_bacterium	0.000193525	6.1198E-05	Thermomicrobiales_JG30-KF-CM45bacterium_QTYC46b	0.000991326	0.000313485
Aliivibrio_sp.	0.00118115	0.00037351	Trichococcus_sp.	0.000864719	0	ASV_8346	0.001224157	0.000387112	Pedobacter_composti	0.000885565	0.000280004	Treponema_socranskii	0.000193525	6.1198E-05	ASV_3639	0.000980388	0.000270152
Lawsonella_uncultured_bacterium	0.00115249	0.00020407	ASV_2061	0.000852419	0.000148808	bacterium_YT0326	0.001211338	0.000383059	Rokubacteriales_sp.	0.000885565	0.000280004	Kingella_sp.	0.00018379	5.81195E-05	Monoglobus_sp.	0.000928074	0.000293483

Candidatus_Planktophila_uncultured_bacterium	0.00113325	0.00035837	Cutibacterium_granulosum	0.000847448	0.000241569	Bacillus_thermolactis	0.001180802	0.000373402	Syntrophus_sp.	0.000885565	0.00028004	ASV_2084	0.000180811	5.71775E-05	ASV_12634	0.000908715	0.000287361
Sediminibacterium_uncultured_Sediminibacterium	0.00113325	0.00000000	Jeotgalibaca_sp.	0.000823648	0.00026046	Roseomonas_genomospecies	0.00117096	0.00037029	Acidimicrobiia_IMCC26256uncultured_Actinomycetales	0.000862503	0.000272747	ASV_363	0.000173345	3.65867E-05	ASV_2692	0.000875757	0.000276939
Rokubacteriales_WX65_uncultured_bacterium	0.00111899	0.00000000	Clostridium_sensu_stricto_1_metagenome	0.000797448	0.000252175	Sphingomonas_oligoaromativorans	0.001156148	0.000365606	ASV_13859	0.000846262	0.000267612	Filifactor_alocis	0.000171133	5.4117E-05	ASV_6828	0.000875757	0.000276939
Megamonas_uncultured_bacterium	0.00110846	0.00035053	Fimbriiglobus_uncultured_bacterium	0.000797448	0.000252175	Clostridium_sensu_stricto_8_uncultured_bacterium	0.001155652	0.000365449	Burkholderiales_A21b_uncultured_bacterium	0.000846262	0.000267612	Mogibacterium_timidum	0.000169744	3.77355E-05	Leptotrichia_wadei	0.000865386	0.000193763
Cryobacterium_sp.	0.00110742	0.00035020	Solirubrobacteraceae_uncultured__sp.	0.000784621	0	Lactobacillus_iners	0.001129482	0.000317931	Chloroplast_Vischeria_sp.	0.000846262	0.000267612	Pseudopropionibacterium_sp.	0.000169633	4.62501E-05	ASV_7664	0.000865181	0.000255871
ASV_1592	0.00110544	0.00020451	ASV_14325	0.000778816	0.000246283	Oligoflexales_uncultured__soil	0.001122288	0.000214413	Planctopirus_sp.	0.000846262	0.000267612	Tannerella_sp.	0.000158585	5.0149E-05	Actinotalea_sp.	0.00085923	0.000271712
ASV_363	0.00110179	0.00023455	Candidatus_Roizmanbacteria	0.000778816	0.000246283	Acidobacteriae_Elev-16S-1166_sp.	0.001111343	0.000351438	Spirochaetaceae_uncultured_Spironema_culicis	0.000846262	0.000267612	Oribacterium_parvum	0.00015821	5.00304E-05	Candidatus_Staskawiczbacteria	0.00085923	0.000271712
Arcticibacter_sp.	0.00107466	0.00033984	Fermentimonas_sp.	0.000771118	0.000243849	Beggiatoaceae_sp.	0.001090204	0.000344753	Prevotellaceae_bacterium	0.00082321	0.000260322	Leptotrichia_hofstadii	0.000148336	3.81951E-05	Eubacterium_nodatum_group_sp.	0.00085923	0.000271712
ASV_1901	0.00106289	0.00033611	ASV_3986	0.000737795	0.000233311	Paeniclostridium_sp.	0.001090204	0.000344753	ASV_256	0.000808329	0.000160455	Fretibacterium_sp.	0.000147032	4.64956E-05	Flectobacillus_sp.	0.00085923	0.000271712
Ulvibacter_uncultured_bacterium	0.00106289	0.00000000	Clostridium_sensu_stricto_9uncultured_Clostridium	0.000725464	0.000229412	Desulfobacterota_uncultured_delta_proteobacterium	0.001088139	0.00034441	Oligoflexales_uncultured__sp.	0.000794586	0.000234265	Olsenella_sp.	0.000147032	4.64956E-05	Geobacter_sp.	0.00085923	0.000271712
Aquicella_unidentified	0.00104652	0.00033094	Ferruginibacter_metagenome	0.000725464	0.000229412	Actinomyces_israelii	0.00108518	0.000330928	Aeromonas_uncultured_gamma	0.000787169	0.000248925	Alloprevotella_tannerae	0.000143934	4.55159E-05	Ruminococcaceae_sp.	0.00085923	0.000271712
Chlamydiales_sp.	0.00104652	0.00033094	Marinifilaceae_uncultured__sp.	0.00070373	0.000222539	MidBa8_sp.	0.001083424	0.000342609	Gracilibacteriauncultured_marine	0.000787169	0.000248925	Prevotella_melaninogenica	0.000136907	4.32938E-05	ASV_5713	0.00083768	0.000264898
Thermobacillus_uncultured_compost	0.00104652	0.00000000	Gammaproteobacteria_JG36-TzT-191_metagenome	0.000697767	0.000220653	ASV_2249	0.001057888	0.000214629	Modestobacter_sp.	0.000787169	0.000248925	Kingella_uncultured_bacterium	0.000132304	4.18382E-05	Halieaceae_OM60(NOR5)_cladeuncultured_Oceanospirillales	0.00083768	0.000264898
ASV_9969	0.00103821	0.00032831	Salinispira_sp.	0.000697767	1.1381E-05	Acetobacteraceae_uncultured_alpha_proteobacterium	0.001041884	0.000329473	Sphingomonas_fonticola	0.000787169	0.000248925	ASV_2285	0.000125942	3.98264E-05	Anaerobacterium_uncultured_bacterium	0.000835267	0.000264135
Dietzia_sp.	0.00102860	0.00032527	ASV_2386	0.000693767	0.000126196	ASV_9967	0.001041884	0.000329473	Leeia_uncultured_bacterium	0.00078505	0.00020755	Desulfomicrobium_orale	0.000124409	3.93416E-05	Pseudoclavibacter_sp.	0.000835267	0.000264135
oc32_sp.	0.00102319	0.00032356	ASV_15306	0.000692281	0.000218918	Propionicimonas_metagenome	0.001020131	0.000322594	Planctomycetales_uncultured__sp.	0.000766099	0.000242262	ASV_476	0.000118939	3.76118E-05	ASV_2820	0.000813487	0.000257247
Cutibacterium_granulosum	0.00099730	0.00024262	ASV_4353	0.000692281	0.000218918	Babeliaceaeuncultured_organism	0.001011634	0.000319907	ASV_14847	0.000752233	0.000237877	Fretibacterium_Synergistales_bacterium	0.000118939	3.76118E-05	Caldicellulosiruptor_sp.	0.000813256	0.000257174
Hydrogenophilus_sp.	0.00098191	0.00021006	Fibrobacteraceae_uncultured_bacterium	0.000692281	0.000218918	Berkelbacteriauncultured_Epsilonproteobacteria	0.001011634	0.000319907	Saccharimonadalesuncultured_Sphingobium	0.000752233	0.000237877	Leptotrichia_unidentified	0.000110274	3.48717E-05	Candidatus_Collierbacteriauncultured_Anaerolineales	0.000813256	0.000257174
ASV_12391	0.00097431	0.00030811	Ignatzschineria_sp.	0.000692281	0.000218918	Desulfobacterota_uncultured__sp.	0.001011634	0.000319907	Anaerococcus_uncultured_bacterium	0.000744694	0.000235493	Pasteurellaceae_sp.	0.00010268	3.24703E-05	Clostridioides_sp.	0.000811159	0.000239592
ASV_12397	0.00097431	0.00030811	Spirosoma_litoris	0.000692281	0	Stenotrophobacter_uncultured_bacterium	0.001011634	0.000319907	ASV_292	0.000744694	0.000235493	WolinellaWolinella_sp.	9.67626E-05	3.0599E-05	ASV_2390	0.000803919	0.000156653
Megamonas_hypermegale	0.00097431	0.00030811	ASV_15881	0.00067256	0.000212682	Luteitalea_uncultured_bacterium	0.001009448	0.000319215	Terrisporobacter_sp.	0.00074421	0.00023534	Cardiobacterium_sp.	8.82632E-05	2.79113E-05	Afipia_sp.	0.000793552	0.000250943
Vitreoscilla_sp.	0.00097431	0.00000000	Sphingomonas_soli	0.00067256	0	Candidatus_Omnitrophusuncultured_organism	0.000972425	0.000307508	Pelotomaculum_sp.	0.000703322	0.00022241	Butyrivibrio_Firmicutes_oral	8.29394E-05	2.62277E-05	Rhodanobacter_uncultured_Rudaea	0.000787227	0.000248943
Deinococcus_aquatilis	0.00096720	0.00030585	ASV_2850	0.000665219	0.000162216	Paenibacillus_hordei	0.000938967	0.000296927	Pseudoglutamicibacter_sp.	0.000703322	0.00022241	Butyrivibrio_unidentified	8.29394E-05	2.62277E-05	ASV_2622	0.000755211	0.000238819
Kocuria_marina	0.00096544	0.00022419	ASV_2888	0.000656737	0.000207678	Shinella_sp.	0.000921395	0.000180213	Anaerotignum_lactatifermentans	0.00069869	0.000220945	Actinomyces_israelii	7.80068E-05	2.46679E-05	Acholeplasma_axanthum	0.000742459	0.000234786
ASV_13191	0.00095138	0.00030085	ASV_2910	0.000656022	0.000159935	Butyrivibrio_Eubacterium_sp.	0.00090743	0.000286955	Blastomonas_sp.	0.00069869	0.000220945	Neisseriaceae_sp.	7.35159E-05	2.32478E-05	Chitinophagales_37-13_uncultured_Bacteroidetes	0.000742459	0.000234786
ASV_13193	0.00095138	0.00030085	Methylobacterium_komagatae	0.000654282	0.000206902	Prevotella_colorans	0.000906413	0.000286633	Lactobacillus_helveticus	0.000698481	0.000220879	Paludibacteraceae_F0058_sp.	0.000071967	2.2758E-05	Lawsonella_uncultured_bacterium	0.000742459	0.000234786
Limnohabitans_sp.	0.00095138	0.00030085	ASV_2303	0.000649915	0.000137378	ASV_3526	0.000893839	0.000191991	ASV_15996	0.000688773	0.000217809	Micrococcaceae_sp.	6.84533E-05	2.16468E-05	Parablastomonas_uncultured_bacterium	0.000742459	0.000234786
Paenisporosarcina_sp.	0.00095138	0.00030085	Arcobacter_sp.	0.000640615	0.00020258	Antricoccus_suffuscus	0.000866739	0.000274087	Erwiniaceae_sp.	0.000688773	0.000217809	Leptotrichia_shahii	6.61518E-05	2.0919E-05	ASV_18385	0.000716025	0.000226427
Alistipes_uncultured_Bacteroidetes	0.00094438	0.00029864	ASV_16215	0.000640615	0.00020258	Plantibacter_sp.	0.000856203	0.000139901	Verrucomicrobiaceae_uncultured_uncultured_Verrucomicrobia	0.000688773	0.000217809	Veillonella_sp.	6.24054E-05	1.97343E-05	Frankiales_sp.	0.000709637	0.000224407
Mogibacterium_sp.	0.00094438	0.00029864	Calothrix_PCC-6303_uncultured_cyanobacterium	0.000630467	0.000199371	Cellvibrionaceae_uncultured__metagenome	0.000847937	0.000268141	Anoxybacillus_sp.	0.000685486	0.000149355	Oribacterium_asaccharolyticum	5.39753E-05	1.70685E-05	Streptococcus_mutans	0.000707243	0.000115282
ASV_9910	0.0009	0.0002	Kocuria_marina	0.00062	0.00013	bacterium_YT0325	0.00084	0.00026	Nitrosotaleaceae_uncultured_archaeon	0.00065	0.00020	Filifactor_sp.	4.52028	1.42944	Crocinitomix_sp.	0.00068	0.00021

	3249	9488		6888	7289		3028	6589		8204	8142		E-05	E-05		5375	6735
Isosphaeraceae_uncultured__uncultured_soil	0.00093249	0.00029488	ASV_2160	0.000626184	0.000132177	Hyphomonas_uncultured_bacterium	0.000843028	0.000266589	Nocardia_pseudovaccinii	0.000658204	0.000208142	Anaerovoracaceae_Family_XIII_UCG-006Peptostreptococcaceae_bacterium	4.14697E-05	1.31139E-05	Solitaleauncultured_Solitalea	0.000685375	0.000216735
Lentimicrobiaceae_uncultured_bacterium	0.00093249	0.00029488	Myxococcaceae_P3OB-42uncultured_delta	0.000605746	0.000191554	Amaricoccus_tamworthensis	0.000833507	0.000263578	ASV_1592	0.000641816	0.000139577	ASV_1071	4.14697E-05	1.31139E-05	Patescibacteria_CPR2_uncultured_Firmicutes	0.000666251	0.000144669
Prevotella_nigrescens	0.00089655	0.00025867	Terrimicrobium_metagenome	0.000605746	0	ASV_7706	0.000807559	0.000255373	Rickettsiales_SM2D12unidentified_marine	0.000639445	0.00020221	Odoribacter_Bacteroidetes_sp.	4.14697E-05	1.31139E-05	Rhodoblastus_uncultured_bacterium	0.000651901	0.000206149
ASV_4838	0.00088660	0.00028037	ASV_17791	0.000598086	0.000189131	Tundrisphaera_lichenicola	0.000794511	0.000251246	Pedobacter_cryoconitis	0.000623644	0.000197214	Rhodococcus_erythropolis	3.67579E-05	1.16239E-05	Cutibacterium_granulosum	0.000648007	0.000142916
Fusicatenibacter_uncultured_organism	0.00088574	0.00028010	Pseudomonas_anguilliseptica	0.000598086	0	ASV_1760	0.00078782	0.000249131	Opitutaceae_uncultured_soil__metagenome	0.000620578	0.000196244	ASV_678	3.30759E-05	1.04595E-05	Hungateiclostridiaceae_uncultured_Firmicutes	0.000647298	0.000204694
LiUU-11-161uncultured_Bacteroidetes	0.00088574	0.00028010	Bacteroidetes_vadinHA17_sp.	0.000588466	0.000186089	Actinomycetaceae_uncultured__sp.	0.000767181	0.000242604	Flavobacteriales_NS9_marine_group_uncultured_soil	0.000611354	0.000193327	Neisseriaceae_uncultured_organism	3.30759E-05	1.04595E-05	ASV_3580	0.000638058	0.000151595
Micrococcales_sp.	0.00088407	0.00027957	Chloroplast_Melosira_varians	0.000588466	0.000186089	CAG-56_uncultured_bacterium	0.000767181	0.000242604	Marinifilaceae_uncultured__sp.	0.000611354	0.000193327	Slackia_exigua	2.26014E-05	7.14719E-06	Bdellovibrionales_bacterium	0.000609942	0.000192881
ASV_2160	0.00087274	0.00018401	Halieaceae_OM60(NOR5)_clade_sp.	0.000588466	0.000186089	Candidatus_Protochlamydia_metagenome	0.000764048	0.000241613	Micrarchaeales_CG1-02-32-21_sp.	0.000611354	0.000193327	ASV_9	1.65379E-05	5.22974E-06	Candidatus_Shapirobacteria_uncultured_bacterium	0.000609942	0.000192881
Hyphomicrobiaceae_uncultured__metagenome	0.00085625	0.00027077	Holosporaceae_uncultured_Holosporaceae	0.000588466	0.000186089	Bergeyella_sp.	0.00075542	0.000238885	Tardiphaga_sp.	0.000611354	0.000193327	Xanthomonadaceae_Xanthomonas_sp.	1.56014E-05	4.9336E-06	Chloroflexi_KD4-96_uncultured_bacterium	0.000609942	0.000192881
ASV_6442	0.00085266	0.00026963	Leuconostoc_sp.	0.000588466	0.000186089	ASV_8430	0.000726803	0.000229835	WPS-2_uncultured_bacterium	0.000611354	0.000193327	009E01-B-SD-P15__uncultured_bacterium	0	0	Ruminiclostridium_metagenome	0.000609239	0.000133363
Dehalococcoidia_S085_metagenome	0.00085266	0.00026963	Thermomicrobiales_JG30-KF-CM45_metagenome	0.000588466	0	Lachnospiraceae_uncultured_Eubacterium	0.000726803	0.000229835	C39_uncultured_bacterium	0.000598698	0.000189325	1013-28-CG33__uncultured_soil	0	0	Acidithiobacillaceae_KCM-B-112_sp.	0.000578273	0.000182866
Saccharimonadalesuncultured_organism	0.00084994	0.00000000	ASV_12970	0.000586441	0.000185449	Ferruginibacter_uncultured_Ferruginibacter	0.000689343	0.000217989	Actinomyces__uncultured_bacterium	0.000590991	0.000186888	Abditibacterium_sp.	0	0	Hydrogenophilus_sp.	0.000578273	0.000182866
ASV_366	0.00084734	0.00015791	Bdellovibrio_uncultured_delta	0.00057648	0.000182299	Rubritepida_uncultured_bacterium	0.000686425	0.000217067	ASV_5066	0.000590377	0.000186694	Absconditabacteriales_(SR1)__metagenome	0	0	Intrasporangiaceae_sp.	0.000578273	0.000182866
Anaerovoracaceae_Family_XIII_AD3011_groupuncultured_Clostridiales	0.00083057	0.00026265	Methylacidiphilaceae_uncultured__sp.	0.00057648	0.000182299	Catellicoccus_sp.	0.000680087	0.000215062	Cloacimonadaceae_uncultured_bacterium	0.000590377	0.000186694	Absconditabacteriales_(SR1)__uncultured_bacterium	0	0	Arcobacteraceae_sp.	0.00057282	0.000181142
Rothia_aeria	0.00082761	0.00000000	Patescibacteria_CPR2_uncultured_Firmicutes	0.00057648	0.000182299	Lentimicrobiaceae_wastewater__metagenome	0.00067981	0.000214975	Dehalococcoidia_uncultured__sp.	0.000590377	0.000186694	Absconditabacteriales_(SR1)_candidate_division	0	0	Berkelbacteria_sp.	0.00057282	0.000181142
Rhodospirillales_uncultured_Rhodospirillaceae	0.00082288	0.00000000	Oligoflexales_uncultured__soil	0.000571916	0.000151456	Tepidiphilus_uncultured_bacterium	0.00067981	0.000214975	Lachnospiraceae_NK4A136_group_sp.	0.000590377	0.000186694	Absconditabacteriales_(SR1)_sp.	0	0	Candidatus_Blackburnbacteria	0.00057282	0.000181142
Solobacterium_moorei	0.00082288	0.00000000	ASV_7308	0.000551659	0.00017445	Atopostipes_sp.	0.000674423	0.000213271	MarmoricolaMarmoricola_sp.	0.000579206	0.000183161	Absconditabacteriales_(SR1)_uncultured_candidate	0	0	Candidatus_Saccharimonas_uncultured_rumen	0.00057282	0.000181142
Slackia_isoflavoniconvertens	0.00080816	0.00000000	Candidatus_Finniella	0.000549099	0.00017364	Dorea_sp.	0.000674423	0.000213271	Rhodocytophaga_sp.	0.000579206	0.000183161	Absconditabacteriales_(SR1)_uncultured_Epsilonproteobacteria	0	0	Dorea_uncultured_Lachnospiraceae	0.00057282	0.000181142
ASV_4573	0.00080296	0.00025392	Saccharina_japonica	0.000527797	0	Peptoclostridium_uncultured_organism	0.000674423	0.000213271	Sporomusaceae_uncultured__bacterium	0.000579206	0.000183161	Absconditabacteriales_(SR1)_uncultured_organism	0	0	Erysipelatoclostridium_sp.	0.00057282	0.000181142
ASV_14135	0.00079717	0.00025209	Pseudomonas_agarici	0.000525389	0.000189131	ASV_2888	0.000669647	0.000211761	ASV_3544	0.000575002	0.000181832	Absconditabacteriales_(SR1)_uncultured_prokaryote	0	0	Jeotgalicoccus_sp.	0.00057282	0.000181142
Planctomycetota_OM190_metagenome	0.00079717	0.00025209	Sporomusaceae_uncultured__bacterium	0.000519211	0	Smithella_sp.	0.000650054	0.000205565	Finegoldia_uncultured_bacterium	0.00056462	0.000131154	ABY1_sp.	0	0	Leptospiraceae_RBG-16-49-21_sp.	0.00057282	0.000181142
Noviherbaspirillum_sp.	0.00079129	0.00021514	uncultured_Thermoflavimicrobium	0.000519211	0	Uliginosibacterium_metagenome	0.000650054	0.000205565	Anaerolineaceae_uncultured_Longilinea	0.000564175	0.000178408	Acanthopleuribacter__uncultured_bacterium	0	0	Spirosomaceae_uncultured__bacterium	0.00057282	0.000181142
ASV_13923	0.00076739	0.00024267	ASV_256	0.000498405	0.000157609	ASV_5871	0.000646047	0.000204298	BSV26_uncultured_bacterium	0.000564175	0.000178408	Acetitomaculum__uncultured_bacterium	0	0	Thermoleophilia_uncultured__sp.	0.00057282	0.000181142
ASV_13925	0.00076739	0.00024267	Chloroplast_Cymbella_subturgidula	0.000498405	0.000157609	Bacillaceae_uncultured__bacterium	0.000646047	0.000204298	Rs-M47uncultured_delta	0.000564175	0.000178408	Acetitomaculum__uncultured_rumen	0	0	Candidatus_Colwellbacteria_uncultured_bacterium	0.000571146	0.000180612
ASV_13930	0.00076739	0.00024267	Streptomyces_sp.	0.000498405	0	Propionivibrio_metagenome	0.000646047	0.000204298	Cytophaga_xylanolytica_groupuncultured_prokaryote	0.000548806	0.000173548	Acetitomaculum_sp.	0	0	Christensenellaceae_R-7_group_iron-reducing_bacterium	0.000571146	0.000180612
Candidatus_Methanoplasma_uncultured_archaeon	0.00076135	0.00024076	ASV_1592	0.000483271	9.00052E-05	Marinifilaceae_uncultured__bacterium	0.00062513	0.000197683	ASV_17382	0.000548095	0.000173323	Acetivibrio_uncultured_bacterium	0	0	Clostridium_acidisoli	0.000571146	0.000180612
Pirellulauncultured_organism	0.00076111	0.00024068	Sporichthyaceae_metagenome	0.000469153	0	ASV_13794	0.000612078	0.000193556	Thermomicrobiales_JG30-KF-CM45uncultured_Thermomicrobium	0.000547214	0.000173044	Acetoanaerobium_uncultured_bacterium	0	0	Prolixibacteraceae_sp.	0.000571146	0.000180612
ASV_14819	0.00075550	0.00023891	Syntrophia_sp.	0.000469153	0	SBR1031_wastewater__metagenome	0.000612078	0.000193556	Epulopiscium_uncultured_Clostridium	0.000537835	0.000170078	Acetobacter_sp.	0	0	Gracilibacteria_Candidatus_Peregrinibacteria	0.000570366	0.000180366
ASV_14825	0.00075550	0.00023891	ASV_2249	0.00046787	0.000147953	Burkholderiales_TRA3-20_metagenome	0.000604275	0.000191089	Nakamurella_uncultured_bacterium	0.000507382	0.000160448	Acetobacteraceae_sp.	0	0	Defluviitaleaceae_UCG-011_sp.	0.000559026	0.000152443
Thermomicrobiales_AKYG1722_sp.	0.00075550	0.00000000	Sandaracinus_sp.	0.00046787	0	Cavicella_uncultured_bacterium	0.000604275	0.000191089	ASV_11446	0.000496463	0.000156995	Acetobacteraceae_uncultured__bacterium	0	0	ASV_7512	0.000556845	0.00017609

Lentimicrobium_saccharophilum	0.00074599	0.00023590	Izemoplasmataceae_uncultured_bacterium	0.000467344	0.000147787	Corynebacterium_propinquum	0.000604275	0.000191089	Acetobacter_sp.	0.000491981	0.000155578	Acetobacteraceae_uncultured__metagenome	0	0	Novosphingobium_uncultured_prokaryote	0.000556845	0.00017609
Mitochondria_Eukaryota_sp.	0.00073045	0.00017741	Lachnoanaerobaculum_umeaense	0.000467344	0.000147787	Moraxella_sp.	0.000604275	0.000191089	Anaerofilumuncultured_Anaerofilum	0.000491981	0.000155578	Acetobacteraceae_uncultured__sp.	0	0	Oscillospiraceae_NK4A214_group_sp.	0.000556845	0.00017609
ASV_7625	0.00072002	0.00022769	Caldicoprobacter_uncultured_bacterium	0.000457582	0.0001447	Rokubacteriales_WX65bacterium_WX65	0.000579741	0.00018333	Armatimonadota_uncultured__metagenome	0.000491981	0.000155578	Acetobacteraceae_uncultured_alpha_proteobacterium	0	0	Sulfurimonas_Sulfurimonas_sp.	0.000556845	0.00017609
Galbitalea_sp.	0.00071216	0.00022521	Vampirovibrio_sp.	0.000443977	0	ASV_15081	0.000577826	0.000182725	ASV_18394	0.000491981	0.000155578	Acetobacterium_sp.	0	0	Phreatobacter_metagenome	0.000545003	0.000172345
Clostridium_sensu_stricto_1_metagenome	0.00070859	0.00022408	Flavobacteriales_NS9_marine_group_uncultured_bacterium	0.000432676	0.000136824	ASV_15083	0.000577826	0.000182725	Candidatus_Renichlamydia_uncultured_Candidatus	0.000491981	0.000155578	Acetothermiia__uncultured_bacterium	0	0	Fusobacterium_necrophorum	0.000533069	0.000168571
Myxococcaceae_uncultured__sp.	0.00069853	0.00022089	Moraxella_catarrhalis	0.000432676	0.000136824	Hyphomonadaceae_SWB02_uncultured_soil	0.000577826	0.000182725	Cytophaga_uncultured_Bacteroidetes	0.000491981	0.000155578	Acholeplasma__uncultured_bacterium	0	0	Pirellulauncultured_Pirellula	0.000533069	0.000168571
LActobacillales_sp.	0.00068761	0.00021744	Prevotella_corporis	0.000420312	0.000132914	Rothia_dentocariosa	0.000577826	0.000182725	Dysgonomonadaceae_uncultured__uncultured_soil	0.000491981	0.000155578	Acholeplasma_axanthum	0	0	Deinococcus_radioresistens	0.000524818	0.000165962
Gaiellales_sp.	0.00068382	0.00021624	Anoxybacillus_sp.	0.000414551	0.000131093	ASV_10346	0.000565291	0.000178761	Microscillaceae_sp.	0.000491981	0.000155578	Acholeplasma_brassicae	0	0	Candidatus_Abawacabacteria	0.000521365	0.00011058
Sphingobacteriaceae_uncultured__sp.	0.00068382	0.00000000	Prevotella_nigrescens	0.000414551	0.000131093	ASV_3443	0.000565291	0.000178761	Rhizobiales_Incertae_Sedis_uncultured__bacterium	0.000491981	0.000155578	Acholeplasma_laidlawii	0	0	Fretibacterium_uncultured_bacterium	0.000519247	0.00016442
Sphingobacteriales_KD3-93_metagenome	0.00068382	0.00000000	Cloacimonadia_PBS-18_uncultured_bacterium	0.000413885	0.000130882	Salinicoccus_sp.	0.000565291	0.000178761	Rhizobiales_sp.	0.000491981	0.000155578	Acholeplasma_morum	0	0	ASV_19068	0.000508285	0.000160734
Acidocella__uncultured_bacterium	0.00068213	0.00021571	Phycisphaerales_AKAU3564_sediment_group_uncultured_bacterium	0.000410509	0.000129814	Eubacterium_coprostanoligenes_group__uncultured_rumen	0.000555671	0.000175719	Paeniclostridium_sp.	0.000482013	0.000152426	Acholeplasma_sp.	0	0	ASV_2910	0.000508285	0.000160734
ASV_14909	0.00068213	0.00021571	SR-FBR-L83_uncultured_bacterium	0.000410509	0	Acidithiobacillus_ferrooxidans	0.00054407	0.00017205	ASV_5832	0.000473969	0.000149882	Acholeplasmataceae_EMP-G18_uncultured_Mollicutes	0	0	Chloroflexia_JG30-KF-CM69_uncultured_soil	0.000508285	0.000160734
Butyricicoccaceae_UCG-009_uncultured_Ruminococcaceae	0.00068213	0.00021571	Afipia_sp.	0.000394042	0.000124607	ASV_14771	0.00054407	0.00017205	Bacteroidetes_vadinHA17__uncultured_soil	0.000470146	0.000148673	Acholeplasmauncultured_Acholeplasmataceae	0	0	Parcubacteriawastewater__metagenome	0.000508285	0.000160734
Fibrobacteria_sp.	0.00068213	0.00021571	ASV_2889	0.000392311	0.00012406	Gammaproteobacteria_CHAB-XI-28_uncultured_bacterium	0.00054407	0.00017205	Rikenellaceae_uncultured_bacterium	0.000470146	0.000148673	Achromatium_sp.	0	0	ASV_1642	0.000504633	0.000159579
Dialister_sp.	0.00066597	0.00021060	ASV_9591	0.000392311	0.00012406	Leptospiraceae_RBG-16-49-21_microbial_mat	0.00054407	0.00017205	ASV_3349	0.000455091	0.000143912	Acidaminobacter__uncultured_bacterium	0	0	ASV_5529	0.000501939	0.000158727
Tissierella_sp.	0.00066531	0.00003106	Candidatus_Colwellbacteria_sp.	0.000392311	0.00012406	Leeia_uncultured_bacterium	0.000537701	0.000114048	Leptospira_biflexa	0.000455091	0.000143912	Acidaminococcaceae_uncultured__bacterium	0	0	Clostridia_UCG-014_Clostridiales_bacterium	0.000501939	0.000158727
Candidatus_Saccharimonas	0.00066305	0.00020967	Acetobacterium_sp.	0.00038432	0.000121533	Ktedonobacteraceae_uncultured_Ktedobacteria	0.000528741	0.000167203	Atopobiaceae_sp.	0.000449023	0.000141994	Acidibacter__metagenome	0	0	ASV_2084	0.000496686	0.000157066
Candidatus_Collierbacteriauncultured_Chloroflexi	0.00066106	0.00020905	Saprospiraceae_uncultured_Bacteroidetes	0.000377895	0	Pediococcus_sp.	0.000527037	0.000166664	Simkaniaceae_uncultured_marine_sediment	0.000437771	0.000138435	Acidibacter__uncultured_bacterium	0	0	Blvii28_wastewater-sludge_group_uncultured_Bacteroidetes	0.000495663	0.000156742
Coriobacteriia_OPB41_sp.	0.00066106	0.00020905	Corynebacterium_amycolatum	0.000373875	0.00011823	Cellvibrio_metagenome	0.000524913	0.000165992	Solitaleauncultured_Sphingobacteriales	0.000437771	0.000138435	Acidibacter_sp.	0	0	Monoglobus_uncultured_Firmicutes	0.000495663	0.000156742
Luteolibacter_luojiensis	0.00066106	0.00020905	Myroides_indicus	0.000373875	0.00011823	Chitinophagaceae_uncultured_uncultured_Sphingobacterium	0.000505817	0.000159953	WS4_uncultured_bacterium	0.000437771	0.000138435	Acidimicrobiia_IMCC26256_metagenome	0	0	Ruminococcus_bicirculans	0.00048463	0.000153253
Cloacibacterium_sp.	0.00065696	0.00014848	Tundrisphaera_sp.	0.000373875	0	Methylotenera_sp.	0.000505817	0.000159953	Anaerorhabdus_furcosa_group__uncultured_bacterium	0.000436681	0.000138091	Acidimicrobiia_IMCC26256_sp.	0	0	ASV_6484	0.000484447	0.000153196
Flexibacter_flexilis	0.00064480	0.00020390	Clostridium_gasigenes	0.000367773	0.0001163	Runella_sp.	0.000505817	0.000159953	ASV_10605	0.000436681	0.000138091	Acidimicrobiia_IMCC26256_uncultured_bacterium	0	0	Meiothermus_uncultured_Deinococci	0.000469909	0.000148598
Fibrobacterales_sp.	0.00062332	0.00013505	Flavobacterium_unidentified	0.000366066	0.00011576	Oscillospirales_UCG-010uncultured_Ruminococcaceae	0.000505598	0.000159884	Woesearchaeales_SCGC_AAA286-E23_uncultured_archaeon	0.000424078	0.000134105	Acidimicrobiia_IMCC26256_uncultured_soil	0	0	Gemmataceae_uncultured_planctomycete_WY69	0.000467322	0.00014778
Anaerococcusuncultured_organism	0.00062166	0.00019659	Flexibacter_sp.	0.000366066	0.00011576	ASV_11616	0.000484535	0.000153223	ASV_12965	0.000413719	0.000130829	Acidimicrobiia_IMCC26256uncultured_Aciditerrimonas	0	0	Chujaibacter_uncultured_Xanthomonadaceae	0.000465643	0.000147249
Ochrobactrum_sp.	0.00062166	0.00019659	Babelialesuncultured_prokaryote	0.0003599	0.00011381	Rikenellaceae_uncultured_anaerobic	0.000484535	0.000153223	ASV_2250	0.000393994	0.000124592	Acidimicrobiia_IMCC26256uncultured_actinobacterium	0	0	Siphonobacter_uncultured_bacterium	0.000464262	0.000146813
Photobacterium_sp.	0.00062166	0.00019659	Cloacibacterium_sp.	0.000358965	7.5695E-05	Bacteriovoracaceae_uncultured_deep-sea	0.000476061	0.000150544	ASV_19717	0.000393585	0.000124463	Acidimicrobiia_IMCC26256uncultured_Actinomycetales	0	0	Candidatus_Collierbacteria_uncultured_bacterium	0.000464037	0.000146741
ASV_3161	0.00061793	0.00019541	Armatimonadales_uncultured_Chloroflexi	0.000351865	0.000111269	Corynebacterium_mycetoides	0.000476061	0.000150544	Brevundimonas_terrae	0.000393585	0.000124463	Acidimicrobiia_sp.	0	0	Eubacterium_limosum	0.000464037	0.000146741
Oxalobacteraceae_CM1G08_sp.	0.00061716	0.00019516	ASV_3560	0.000351865	0.000111269	Ralstonia_sp.	0.000476061	0.000150544	Flectobacillus_fontis	0.000393585	0.000124463	Acidimicrobiia_uncultured__bacterium	0	0	Holophagaceae_uncultured__bacterium	0.000464037	0.000146741
Actinobacteria_PeM15_metagenome	0.00059686	0.00018874	Lacibacter_sp.	0.000351865	0.000111269	Candidatus_Limnoluna_uncultured_bacterium	0.000473925	0.000101271	LiUU-11-161_metagenome	0.000393585	0.000124463	Acidimicrobiia_uncultured__metagenome	0	0	Hungateiclostridiaceae_uncultured_Saccharofermentans	0.000464037	0.000146741
Chloroflexi_TK10uncultured_Chloroflexus	0.00059686	0.00018874	ASV_20168	0.000346141	0.000109459	Lachnospiraceae_uncultured_Cellulosilyticum	0.000472692	0.000149478	Macellibacteroides_uncultured_bacterium	0.000393585	0.000124463	Acidimicrobiia_uncultured__sp.	0	0	Sporichthyaceae_sp.	0.000464037	0.000146741
Demequinaceae_uncultured__bacterium	0.0005	0.0001	ASV_3349	0.00034	0.00010	Methanobrevibacter_millerae	0.00045	0.00014	Planctomycetota_OM190_planctomycete_A-2	0.00039	0.00012	Acidimicrobiia_uncultured_Acidimicrobiia	0	0	Sulfurospirillum_sp.	0.00046	0.00014

	9686	8874		6141	9459		6765	4442		3585	4463					4037	6741
Lactobacillus_acidipiscis	0.00059686	0.00018874	Beijerinckiaceae_1174-901-12__uncultured_bacterium	0.00346141	0.00109459	Nocardia_sp.	0.00453206	0.00143316	ASV_9917	0.00380537	0.00120336	Acidimicrobiia_uncultured_actinobacterium	0	0	Woesearchaeales_SCGC_AAA011-D5Nanoarchaeota_archaeon	0.00464037	0.00146741
Flavobacteriales_sp.	0.00059648	0.00018862	Candidatus_Ryanbacteria_sp.	0.00346141	0.00109459	Noviherbaspirillum_sp.	0.00453206	0.00143316	Candidatus_Vogelbacteria_uncultured_Parcubacteria	0.00376117	0.00118939	Acidimicrobiia_uncultured_Actinomycetales	0	0	Cloacimonadales_uncultured_soil	0.00450122	0.00142341
Microtrichales_sp.	0.00059648	0.00018862	Hyaloperonospora_arabidopsidis	0.00346141	0.00109459	Paludisphaera_sp.	0.00453206	0.00143316	Dehalococcoidia_RBG-13-46-9_metagenome	0.00376117	0.00118939	Acidimicrobiia_uncultured_bacterium_YC-ZSS-LKJ199	0	0	Saccharimonadalesuncultured_organism	0.00450122	0.00142341
ASV_3228	0.00059106	0.00018691	ASV_7625	0.00341503	0.00107993	Peptoniphilus_sp.	0.00453206	0.00143316	Flavobacterium_subsaxonicum	0.00376117	0.00118939	Acidiphilium__uncultured_bacterium	0	0	Methanofastidiosales_uncultured_archaeon	0.00450014	0.00142307
Rhizorhapis_uncultured_Sphingomonadaceae	0.00057083	0.00000000	ASV_5084	0.00338413	8.31018E-05	Rhizobiales_Incertae_Sedis_uncultured_Alphaproteobacteria	0.00453206	0.00143316	Luteolibacter_uncultured_Verrucomicrobia	0.00376117	0.00118939	Acidiphilium_sp.	0	0	Chloroplast_Planoglabratella_opercularis	0.00436344	0.00137984
Sumerlaeauncultured_Desulfocaldus	0.00057083	0.00000000	Chloroplast_Cymatopleura_solea	0.00327141	0.00103451	Sphingobacteriales_NS11-12_marine_groupHaematococcus_lacustris	0.00453206	0.00143316	Pedosphaeraceae_ADurb.Bin118__uncultured_bacterium	0.00376117	0.00118939	Acidisoma_sp.	0	0	ASV_6340	0.00432706	0.00136834
Enterobacteriaceae_sp.	0.00056663	0.00017918	ASV_11414	0.00315234	9.96857E-05	ASV_6702	0.00452997	0.00014325	Rubinisphaeraceae_SH-PL14_sp.	0.00376117	0.00118939	Acidithiobacillaceae_KCM-B-112_sp.	0	0	allotenuales_AKIW781_uncultured_bacterium	0.00429615	0.00135856
Oligoflexus_uncultured_delta	0.00056663	0.00017918	Pyrinomonadaceae_RB41_sp.	0.00313273	0	Devosia_uncultured_Rhizobiales	0.00452997	0.00014325	S-BQ2-57_soil_group_uncultured_bacterium	0.00376117	0.00118939	Acidithiobacillus_ferrooxidans	0	0	Candidatus_Moranbacteria__uncultured_soil	0.00429615	0.00135856
Paludibacteraceae_uncultured__bacterium	0.00056663	0.00017918	Acidothermus_sp.	0.00303774	9.60618E-05	Eisenbergiella_sp.	0.00444157	0.00140455	Sericytochromatia_uncultured_bacterium	0.00376117	0.00118939	Acidobacteria_bacterium	0	0	Hyphomonadaceae_SWB02_sp.	0.00429615	0.00135856
Enhydrobacter_sp.	0.00056482	0.00017861	Oscillospiraceae_UCG-002gut__metagenome	0.00303774	9.60618E-05	Geobacteraceae_uncultured_bacterium	0.00444157	0.00140455	ASV_9602	0.00374186	0.00118328	Acidobacteriaceae_(Subgroup_1)_sp.	0	0	Kapabacteriales_metagenome	0.00429615	0.00135856
Sphingomonas_koreensis	0.00056420	0.00000000	ASV_21390	0.00299043	9.45657E-05	Prevotella_nigrescens	0.00442422	0.00010155	Planktothrix_NIVA-CYA_15_sp.	0.00372347	0.00117746	Acidobacteriae_Elev-16S-1166_sp.	0	0	NannocystaceaeEnhygromyxa_sp.	0.00429615	0.00135856
ASV_2433	0.00055949	0.00017693	Solirubrobacterales_67-14_sp.	0.00299043	0	Candidatus_Ovatusbacter	0.00433369	0.00137043	Methanobacteriumuncultured_Methanobacteriaceae	0.00372105	0.00011767	Acidobacteriae_Elev-16S-1166_uncultured_bacterium	0	0	Oligoflexales_uncultured__sp.	0.00429615	0.00135856
Johnsonella_ignava	0.00055371	0.00017510	Clostridium_sensu_stricto_13_Clostridium_sp.	0.00293221	9.27246E-05	Microscillaceae_uncultured_Cytophagaceae	0.00042163	0.00133331	ASV_19908	0.00365397	0.00115549	Acidobacteriae_sp.	0	0	Sphingobacteriales_NS11-12_marine_group_metagenome	0.00429615	0.00135856
ASV_6989	0.00054026	0.00017085	ASV_12134	0.00288964	9.13784E-05	Trueperauncultured_Deinococcales	0.00416753	0.00131789	Candidatus_Saccharimonas_uncultured_bacterium	0.00365397	0.00115549	Acidocella__uncultured_bacterium	0	0	Methylobacterium_jeotgali	0.00423891	0.00134046
Clostridia_UCG-014_uncultured_organism	0.00053144	0.00016806	Gardnerella_sp.	0.00028824	9.11495E-05	ASV_16969	0.00408052	0.00129037	Sphingobacteriales_KD3-93_uncultured_Bacteroidetes	0.00365397	0.00115549	Acidothermus__uncultured_bacterium	0	0	Chromobacteriaceae_sp.	0.00421575	0.00133314
Dehalococcoidia_RBG-13-46-9hot_springs	0.00053144	0.00016806	Gemmata_uncultured_Gemmata	0.00028824	9.11495E-05	Fervidobacterium_uncultured_bacterium	0.00408052	0.00129037	ASV_9492	0.00349345	0.00110473	Acidothermus_sp.	0	0	Polynucleobacter_cosmopolitanus	0.00415397	0.00013136
Sporichthyaceae_hgcl_clade_uncultured_Frankineae	0.00053144	0.00000000	Simplicispira_metagenome	0.00028824	0	Geobacter_uncultured_Banisveld	0.00408052	0.00129037	Cellvibrionaceae_sp.	0.00349345	0.00110473	Acidovorax_sp.	0	0	Anoxybacillus_sp.	0.00413052	0.00130619
Chryseoglobus_sp.	0.00051160	0.00016178	Candidatus_Paracaedibacter_sp.	0.00028792	9.10483E-05	Agromyces_sp.	0.00403779	0.00127686	Candidatus_Finniella_sp.	0.00034924	0.00110439	Acinetobacter__metagenome	0	0	SJA-28uncultured_Ignavibacterium	0.00413052	0.00130619
Corynebacterium_mycetoides	0.00051160	0.00016178	Desulfitobacteriales_TC1_uncultured_bacterium	0.00274549	8.682E-05	ASV_13184	0.00403779	0.00127686	Mycobacterium_abscessus	0.00034924	0.00110439	Acinetobacter_baumannii	0	0	ASV_3568	0.00407568	9.43391E-05
Gemmataceae_uncultured_uncultured_Zavarzinella	0.00051160	0.00016178	Oscillospirales_UCG-010_sp.	0.00274549	8.682E-05	ASV_3442	0.00403779	0.00127686	Centipeda_uncultured_bacterium	0.00329799	0.00104292	Acinetobacter_kyonggiensis	0	0	ASV_20342	0.00406628	0.00128587
Spongiibacteraceae_BD1-7_clade_sp.	0.00051160	0.00000000	Spirosoma_sp.	0.00274549	0	Paenarthrobacter_sp.	0.00403779	0.00127686	Staphylococcus_vitulinus	0.00329799	0.00104292	Acinetobacter_sp.	0	0	ASV_2433	0.00406628	0.00128587
Sporobacter_sp.	0.00051160	0.00000000	Candidatus_Amoebophilus_microbial_mat	0.00269925	8.53578E-05	Chamaesiphon_PCC-7430_uncultured_bacterium	0.00377672	0.00011943	ASV_1976	0.00328328	0.00103826	Acinetobacter_towneri	0	0	Fibrobacteraceae_possible_genus_04_metagenome	0.00406628	0.00128587
Rhizorhapis_metagenome	0.00051047	0.00000000	Absconditabacteriales_(SR1)__uncultured_bacterium	0.00259605	8.20943E-05	Chitinimonas_taiwanensis	0.00377672	0.00011943	ASV_9639	0.00328328	0.00103826	Acremonium_fuci	0	0	Haliangium_uncultured_bacterium	0.00406628	0.00128587
Kaistia_uncultured_bacterium	0.00050471	0.00015960	Candidatus_Kerfeldbacteria_uncultured_bacterium	0.00259605	8.20943E-05	Rhodomicrobium_sp.	0.00377672	0.00011943	Chloroflexia_JG30-KF-CM69_uncultured_soil	0.00324295	0.00102551	Actinobacillus_sp.	0	0	Woeseia_uncultured_bacterium	0.00403706	0.00127663
Oligoflexales_uncultured__bacterium	0.00049115	0.00015531	Gracilibacteriauncultured_beta	0.00259605	8.20943E-05	Verrucomicrobiaceae_sp.	0.00377672	0.00011943	Oligoflexales_uncultured__soil	0.00324295	0.00102551	Actinobacteria_0319-7L14_uncultured_actinobacterium	0	0	ASV_6623	0.00402779	0.00012737
1013-28-CG33__uncultured_soil	0.00048360	0.00015293	Gracilibacteriawastewater__metagenome	0.00259605	8.20943E-05	ASV_7913	0.00374215	0.00118337	ASV_2910	0.00312914	7.22346E-05	Actinobacteria_PeM15_bacterium_rJ7	0	0	Bacteroidales_FTLpost3_uncultured_bacterium	0.00388036	0.00122708
ASV_3021	0.00048360	0.00015293	Oligoflexia_0319-6G20_uncultured_Roseobacter	0.00259605	8.20943E-05	Balneolaceae_sp.	0.00374215	0.00118337	Gaiellales_sp.	0.00030644	9.69048E-05	Actinobacteria_PeM15_metagenome	0	0	Gulosibacter_sp.	0.00385997	0.00122063
Erysipelothrix_uncultured_bacterium	0.00048360	0.00015293	Spirochaetaceae_uncultured__bacterium	0.00259605	0	Pararhodospirillum_photometricum	0.00374215	0.00118337	ASV_11789	0.00304429	9.62689E-05	Actinobacteria_PeM15_sp.	0	0	ASV_1760	0.00383521	0.00012128
Lentimicrobiaceae_wastewater__metagenome	0.00048360	0.00015293	ASV_13785	0.00236425	7.47641E-05	Aquicella_unidentified	0.00363401	0.00114917	Oligoflexus_uncultured_Desulfocurvus	0.00304429	9.62689E-05	Actinobacteria_PeM15_uncultured_soil	0	0	Chloroflexi_OLB14_metagenome	0.00383521	0.00012128
Mucilaginibacter_jinjuensis	0.0004	0.0001	Prolixibacteraceae_WCHB1-	0.00023	0	SS1-B-02-17uncultured_Lentisphaerae	0.00036	0.00011	Paludibaculum_uncultured_bacterium	0.00030	9.62689	Actinobacteria_PeM15uncultured_actinobacterium	0	0	ASV_7105	0.00038	0.00012

	8360	5293	32uncultured_Bacteroidetes	6425			3401	4917		4429	E-05					0781	0414
Eubacterium_eligens_group_sp.	0.00048177	0.00015235	ASV_19533	0.000234577	7.41798E-05	ASV_18612	0.000361141	0.000114203	Anaerolineaceae_uncultured__bacterium	0.000299349	9.46625E-05	Actinobacteria_sp.	0	0	Victivallales_GWF2-44-16_Lentisphaerae_bacterium	0.000380781	0.000120414
Candidatus_Omnitrophusuncultured_Banisveld	0.00047569	0.00015043	ASV_5218	0.000234577	7.41798E-05	ASV_18619	0.000361141	0.000114203	ASV_11362	0.000299349	9.46625E-05	Actinobacteria_uncultured__bacterium	0	0	MoheibacterMoheibacter_sediminis	0.000372515	0.00011178
Cephaloticoccus_sp.	0.00047569	0.00015043	Prevotellaceae_uncultured__metagenome	0.000234577	7.41798E-05	Erysipelothrix_sp.	0.000361141	0.000114203	Bacteroides_massiliensis	0.000299349	9.46625E-05	Actinobacteria_uncultured__metagenome	0	0	ASV_7984	0.000371978	0.000117763
Opitutaceae_IMCC26134_metagenome	0.00047569	0.00015043	ASV_2433	0.000233935	7.39767E-05	Silvanigrella_uncultured_bacterium	0.000361141	0.000114203	ASV_20846	0.000295188	9.33466E-05	Actinobacteria_uncultured__sp.	0	0	Gastranaerophilales_uncultured_cyanobacterium	0.000371978	0.000117763
ASV_18243	0.00047219	0.00014932	Clavibacter_sp.	0.000233935	7.39767E-05	ASV_1642	0.000354519	0.000112109	ASV_20847	0.000295188	9.33466E-05	Actinobacteria_uncultured_Actinomycetales_bacterium	0	0	Aquicella_unidentified	0.00037123	0.000117393
ASV_3442	0.00047219	0.00014932	ASV_1901	0.00021594	6.82862E-05	ASV_8293	0.000354519	0.000112109	Beggiatoa_uncultured_gamma	0.000295188	9.33466E-05	Actinobacteriota_MB-A2-108_metagenome	0	0	ASV_19569	0.00037123	0.000117393
ASV_5084	0.00047219	0.00014932	Limnobacter_metagenome	0.00021594	6.82862E-05	Qipengyuania_uncultured_bacterium	0.000351358	0.000111109	Lacunisphaera_sp.	0.000295188	9.33466E-05	Actinobacteriota_MB-A2-108_sp.	0	0	Bdellovibrio_uncultured_gamma	0.00037123	0.000117393
bacterium_RS30A	0.00047219	0.00014932	Solirubrobacteraceae_uncultured_Phytoplasma_sp.	0.000210305	2.21681E-05	ASV_19037	0.000347295	0.000109824	Spirochaetaceae_GWE2-31-10_sp.	0.000295188	9.33466E-05	Actinobacteriota_MB-A2-108_uncultured_bacterium	0	0	Caulobacteraceae_uncultured__sp.	0.00037123	0.000117393
Pleomorphomonas_uncultured_bacterium	0.00047219	0.00014932	Dermabacter_sp.	0.000210156	6.64572E-05	ASV_7974	0.000340044	0.000107531	WPS-2_metagenome	0.000295188	9.33466E-05	Actinobacteriota_MB-A2-108uncultured_actinobacterium	0	0	Kiritimatiellae_WCHB1-41unidentified_rumen	0.00037123	0.000117393
ASV_3531	0.00045673	0.00014443	SB-5_uncultured_Bacteroidetes	0.000210156	0	Dorea_uncultured_Dorea	0.000340044	0.000107531	ASV_15821	0.000289603	9.15805E-05	Actinomadura_sp.	0	0	Panacagrimonas_uncultured_bacterium	0.00037123	0.000117393
ASV_8725	0.00045673	0.00014443	Christensenellaceae_R-7_group_sp.	0.000207275	6.55461E-05	Gammaproteobacteria_CHAB-XI-27_uncultured_bacterium	0.000340044	0.000107531	Chroococcidiopsaceae_uncultured_cyanobacterium	0.000289603	9.15805E-05	Actinomyces__uncultured_bacterium	0	0	Phytophthora_agathidicida	0.00037123	0.000117393
Cytophaga_xylanolytica_group_sp.	0.00045673	0.00014443	Peptococcus_sp.	0.000199362	6.30438E-05	Haliangium_uncultured_sludge	0.000340044	0.000107531	Confluentibacter_sp.	0.000289603	9.15805E-05	Actinomyces_dentalis	0	0	Rhizobiaceae_metagenome	0.00037123	0.000117393
Gemmatimonas_phototrophica	0.00042877	0.00014005	ASV_12382	0.000197945	6.25957E-05	Bacteroidia_uncultured__metagenome	0.000337211	0.000106635	Corynebacterium_glutamicum	0.000289603	9.15805E-05	Actinomyces_graevenitzii	0	0	Tyzzerella_uncultured_Eubacteriaceae	0.00037123	0.000117393
Planctomycetes_uncultured_Planctomyces	0.00042877	0.00014005	Oligoflexia_0319-6G20_uncultured_organism	0.000197945	6.25957E-05	Candidatus_Uhrbacteria	0.000337211	0.000106635	Quadrisphaera_uncultured_bacterium	0.000289603	9.15805E-05	Actinomyces_sp.	0	0	Williamsia_sp.	0.00037123	0.000117393
Plantibacter_sp.	0.00042877	0.00014005	Mitochondria_Eukaryota_sp.	0.00019216	6.07663E-05	Nitrosomonadaceae_966-1__metagenome	0.000334824	0.000105881	ASV_20321	0.000287501	9.09158E-05	Actinomyces_unidentified	0	0	Candidatus_Bealeia_uncultured_Alphaproteobacteria	0.000369214	0.000116756
Lactobacillales_P5D1-392Granulicatella_sp.	0.00042003	0.00013978	ASV_2862	0.000186938	5.9115E-05	Rudanella_uncultured_bacterium	0.000334824	0.000105881	Hypnocyclicus_sp.	0.000287501	9.09158E-05	Actinomycetaceae_uncultured__bacterium	0	0	Oceanobacter_sp.	0.000369214	0.000116756
ASV_2862	0.00038894	0.00009355	Megasphaera_sp.	0.000186938	5.9115E-05	Rummeliibacillus_sp.	0.000333379	0.000105554	Schaalia_odontolytica	0.000284551	8.99829E-05	Actinomycetaceae_uncultured__metagenome	0	0	ASV_14099	0.000342687	0.000108367
Candidatus_Limnoluna_uncultured_bacterium	0.00037844	0.00011006	Chitinophagaceae_uncultured__bacterium	0.000183886	5.81499E-05	ASV_3026	0.000323023	0.000102149	Candidatus_Campbellbacteria	0.000282087	8.92037E-05	Actinomycetaceae_uncultured__rumen	0	0	MBNT15_sp.	0.000342687	0.000108367
ASV_16382	0.00035166	0.00013761	DEV114_uncultured_bacterium	0.000183886	5.81499E-05	Candidatus_Komeiliibacteria_uncultured_bacterium	0.000323023	0.000102149	Fibrobacterales_sp.	0.000282087	8.92037E-05	Actinomycetaceae_uncultured__sp.	0	0	ASV_2303	0.000335649	0.000106142
Proteiniphilumuncultured_prokaryote	0.00035166	0.00000000	Escherichia-Shigella_sp.	0.000183886	5.81499E-05	Edaphobaculum_Chitinophagaceae_bacterium	0.000323023	0.000102149	Mitsuokella_sp.	0.000282087	8.92037E-05	Actinomycetospora_sp.	0	0	ASV_5218	0.000335649	0.000106142
Tolypothrix_sp.	0.00035166	0.00000000	ASV_21806	0.000183033	5.78801E-05	ASV_9126	0.000315128	9.96522E-05	Pleurocapsa_PCC-7319_sp.	0.000282087	8.92037E-05	Actinoplanes_garbadinensis	0	0	Parapedobacter_sp.	0.000330442	0.000104495
Gammaproteobacteria_EV818SWSAP88_sp.	0.00042987	0.00013594	Bacteroidetes_BD2-2_Bacteroidetes_bacterium	0.000183033	5.78801E-05	Xanthomonadaceae_uncultured_Luteimonas_sp.	0.000315128	9.96522E-05	Saccharibacillus_deserti	0.000282087	8.92037E-05	Actinoplanes_sp.	0	0	Reichenbachiella_uncultured_bacterium	0.000330442	0.000104495
Cnuella_uncultured_organism	0.00042633	0.00013482	Candidatus_Megaira_uncultured_bacterium	0.000183033	5.78801E-05	Cellvibrio_uncultured_Cellvibrio	0.000313299	6.60095E-05	Sanguibacter_sp.	0.000282087	8.92037E-05	Actinotalea_sp.	0	0	Saprospiraceae_uncultured_Sargassum_vachellianum	0.000330442	0.000104495
KI89A_clade_sp.	0.00042633	0.00013482	Leeia_uncultured_bacterium	0.000183033	5.78801E-05	Bacteroidia_uncultured_Bacteroidales	0.000302138	9.55444E-05	Tolumonas_sp.	0.000282087	8.92037E-05	Actinotignum_sp.	0	0	ASV_7299	0.00032595	0.000103074
Microscillaceae_uncultured_prokaryote	0.00042633	0.00013482	Peredibacter_uncultured_delta	0.000183033	5.78801E-05	Saccharimonadales_TM7a_sp.	0.000302138	9.55444E-05	Dendrosporobacter_unidentified_eubacterium	0.00027906	8.82465E-05	Adhaeribacter__metagenome	0	0	Legionella_drancourtii	0.000322965	0.000102131
Rhodanobacteraceae_uncultured__metagenome	0.00042633	0.00077646	Verrucomicrobiaceae_uncultured__bacterium	0.000183033	0	SJA-28uncultured_Chlorobi	0.000302138	9.55444E-05	Rhizorhapis_uncultured_Sphingomonas	0.00027906	8.82465E-05	Adhaeribacter__uncultured_soil	0	0	Candidatus_Captivus	0.000318867	0.000100835
ASV_6710	0.00041748	0.00013202	Algibacter_sp.	0.00017995	5.69052E-05	Undibacterium_sp.	0.000302138	9.55444E-05	Candidatus_Jidaibacter_uncultured_bacterium	0.000274403	8.67738E-05	Adhaeribacter_sp.	0	0	Acidimicrobiia_uncultured_actinobacterium	0.000311548	9.85201E-05
Microscillaceae_OLB12_sp.	0.00041748	0.00013202	Ferruginibacter_uncultured_Sphingobacteriales	0.00017995	5.69052E-05	Catonella_uncultured_bacterium	0.000295433	9.34241E-05	Clostridium_sensu_stricto_9_sp.	0.000274403	8.67738E-05	Advenella_sp.	0	0	ASV_15920	0.000304971	9.64403E-05
Edaphobaculum_sp.	0.00041528	0.00013132	Babeliae_sp.	0.000175932	5.56346E-05	Flavobacterium_uncultured_Bacteroidetes	0.000295433	9.34241E-05	Rothia_mucilaginosa	0.000274403	8.67738E-05	Aenigmarchaeales_sp.	0	0	ASV_5084	0.000304971	9.64403E-05
ASV_8328	0.00041295	0.00013059	Caenarcaniphilales_uncultured_bacterium	0.000175932	5.56346E-05	ASV_13680	0.000288913	9.13623E-05	Entomoplasmatales_bacterium	0.000274048	8.66616E-05	Aenigmarcheota_sp.	0	0	Bdellovibrio_bacteriovorus	0.000304971	9.64403E-05

Candidatus_Amesbacteria__uncultured_soil	0.00041144	0.00013011	ASV_15723	0.000163571	5.17257E-05	Fimbriiglobus_metagenome	0.000288913	9.13623E-05	Simkaniaceae_uncultured_Rhabdochlamydiaceae_bacterium	0.000274048	8.66616E-05	Aequorivita_sp.	0	0	Brachybacterium_sp.	0.000304971	9.64403E-05
Trichuris_trichiura	0.00041144	0.00000000	Blastocatellia_sp.	0.000163571	5.17257E-05	Fluviicola_uncultured_Flexibacter	0.000288913	9.13623E-05	ASV_21042	0.000262009	8.28545E-05	Aerococcaceae_sp.	0	0	Myxococcaceae_P3OB-42_uncultured_bacterium	0.000304971	9.64403E-05
ASV_9657	0.00040300	0.00012744	Corynebacterium_glucuronolyticum	0.000163571	5.17257E-05	Iamia_metagenome	0.000288913	9.13623E-05	ASV_2386	0.000262009	8.28545E-05	Aerococcaceae_uncultured__bacterium	0	0	Saccharimonadales_LWQ8_sp.	0.000304971	9.64403E-05
Saccharimonadaceae_metagenome	0.00039292	0.00000000	Fokiniaceae_uncultured__bacterium	0.000163571	5.17257E-05	Salinirepens_sp.	0.000288913	9.13623E-05	ASV_3639	0.000262009	8.28545E-05	Aerococcus_sp.	0	0	oc32_uncultured_bacterium	0.000302084	9.55273E-05
Caulobacter_metagenome	0.00039018	0.00010724	Ilumatobacteraceae_uncultured_marine	0.000163571	5.17257E-05	Dysgonomonadaceae_uncultured_bacterium	0.000281086	8.88872E-05	Bacillaceae_sp.	0.000262009	8.28545E-05	Aeromicrobium_sp.	0	0	009E01-B-SD-P15__uncultured_bacterium	0.00028641	9.05708E-05
ASV_8696	0.00039001	0.00012333	ASV_14118	0.000161955	5.12147E-05	Subgroup_7_sp.	0.000281086	8.88872E-05	Nitrosomonadaceae_Ellin6067_uncultured_bacterium	0.000262009	8.28545E-05	Aeromonas__metagenome	0	0	ASV_21679	0.00028641	9.05708E-05
Blastocatellaceae_uncultured__bacterium	0.00039001	0.00012333	Acholeplasmataceae_EMP-G18_uncultured_Mollicutes	0.000157617	4.98429E-05	ASV_20307	0.000277836	8.78595E-05	Arcobacter_cryaerophilus	0.000253691	8.02241E-05	Aeromonas_sp.	0	0	Candidatus_Anammoximicrobium__uncultured_soil	0.00028641	9.05708E-05
ASV_2910	0.00038055	0.00012034	Babelialesuncultured_Candidatus	0.000140203	4.43361E-05	Solirubrobacter_sp.	0.000277836	8.78595E-05	ASV_13391	0.000253691	8.02241E-05	Aeromonas_uncultured_gamma	0	0	Clostridium_sensu_stricto_13_sp.	0.00028641	9.05708E-05
ASV_2249	0.00038013	0.00008592	Shinella_sp.	0.000140203	0	Weissella_sp.	0.000277836	8.78595E-05	Solobacterium_moorei	0.000249517	7.89042E-05	Aerosphaera__uncultured_bacterium	0	0	Plantibacter_sp.	0.00028641	9.05708E-05
ASV_3803	0.00037775	0.00011946	Chloroplast_Guillardia_theta	0.000131347	4.15356E-05	Acidimicrobiia_uncultured_Actinomycetales	0.000275737	8.71957E-05	Streptococcus_mutans	0.000249517	7.89042E-05	Aerosphaera_sp.	0	0	Singulisphaera_metagenome	0.00028641	9.05708E-05
Turicibacter_sp.	0.00037775	0.00000000	Anaerolineae_1-20uncultured_Chloroflexia	0.000125965	3.98336E-05	ASV_2889	0.000275737	8.71957E-05	Alysiella_sp.	0.000249457	7.88852E-05	Aestuariicella__uncultured_bacterium	0	0	Woesearchaeales_uncultured_euryarchaeote	0.00028641	9.05708E-05
Algoriphagus_uncultured_Hongiella	0.00037300	0.00011795	Geobacteraceae_uncultured__sp.	0.000117288	3.70897E-05	Rhodospirillaceae_uncultured_Magnetospirillum	0.000275737	8.71957E-05	ASV_12880	0.000249457	7.88852E-05	Afipia_genosp.	0	0	ASV_413	0.000285302	9.02204E-05
ASV_5713	0.00037300	0.00011795	Hydrogenophilus_sp.	0.000117288	3.70897E-05	Turicella_uncultured_bacterium	0.000275737	8.71957E-05	Dongia_metagenome	0.000249457	7.88852E-05	Afipia_sp.	0	0	CL500-29_marine_group_uncultured_Acidimicrobiaceae	0.000285302	9.02204E-05
Candidatus_Bacilloplasma_sp.	0.00037300	0.00011795	ASV_18112	0.000116836	3.69468E-05	Dolosigranulum_sp.	0.000272035	8.6025E-05	Fluviicoccus_metagenome	0.000249457	7.88852E-05	Agathobacter_sp.	0	0	Chloroflexi_OLB13_uncultured_bacterium	0.000279386	8.83496E-05
Steroidobacterbacterium_MI-37	0.00037300	0.00000000	ASV_17387	0.00010797	3.41431E-05	Gaiellales_uncultured_organism	0.000272035	8.6025E-05	Pedomicrobium)uncultured_Pedomicrobium	0.000249457	7.88852E-05	Aggregatibacter_sp.	0	0	Diplorickettsia_uncultured_bacterium	0.000278422	8.80448E-05
ASV_9586	0.00036836	0.00011649	Cryomorphaceae_sp.	0.00010797	3.41431E-05	Kiritimatiellaceae_MSBL3_uncultured_bacterium	0.000272035	8.6025E-05	ASV_2862	0.000248231	7.84975E-05	Agitococcus_lubricus_group__uncultured_bacterium	0	0	Lachnospiraceae_uncultured__rumen	0.000278422	8.80448E-05
Flavobacterium_cheniae	0.00036836	0.00011649	Anaerolineaceae_UCG-001_uncultured_bacterium	0.000105078	3.32286E-05	ASV_9616	0.000263518	8.33317E-05	Bordetella_sp.	0.000248231	7.84975E-05	Agitococcus_lubricus_group_sp.	0	0	Neisseriaceae_uncultured__metagenome	0.000278422	8.80448E-05
Anoxybacillus_sp.	0.00036707	0.00011608	ASV_19465	0.000105078	3.32286E-05	Nostoc_PCC-73102_sp.	0.000263518	8.33317E-05	Dermabacter_sp.	0.000240774	7.61394E-05	Agrococcus_sp.	0	0	Peredibacter_metagenome	0.000278422	8.80448E-05
Nevskia_uncultured_bacterium	0.00036707	0.00011608	Chloroplast_Prasiolopsis_sp.	0.000105078	3.32286E-05	Oligoflexia_0319-6G20_uncultured_marine	0.000263518	8.33317E-05	Holosporaceae_uncultured__metagenome	0.000224512	7.09969E-05	Agromyces_sp.	0	0	Syntrophomonas_sp.	0.000278422	8.80448E-05
Candidatus_Kaiserbacteria_sp.	0.00035430	0.00011204	Lactobacillus_iners	0.000105078	3.32286E-05	Chloroplast_Castanea_mollissima	0.000256042	8.09676E-05	Hyphomicrobium_uncultured_Hyphomicrobiaceae	0.000224512	7.09969E-05	Ahniella_affigens	0	0	ASV_2103	0.000276932	8.75736E-05
Corynebacterium_glutamicum	0.00035430	0.00011204	Dyadobacter_metagenome	9.34689E-05	2.95575E-05	ASV_1837	0.000245951	7.77765E-05	Gemmataceae_uncultured_Planctomycetales	0.000218885	6.92175E-05	Ahniella_metagenome	0	0	ASV_9331	0.000276932	8.75736E-05
Nordella_oligomobilis	0.00034607	0.00010944	FCPU426_sp.	9.34689E-05	2.95575E-05	Coriobacteriia_OPB41uncultured_Coriobacteriaceae	0.000245951	7.77765E-05	Opitutus_uncultured_Verrucomicrobia	0.000218885	6.92175E-05	Ahniella_uncultured_bacterium	0	0	Mitochondria_marine__metagenome	0.000276932	8.75736E-05
Bradyrhizobium_sp.	0.00034380	0.00010872	KI89A_clade_uncultured_bacterium	0.0000899975	2.84526E-05	Elusimicrobia_MVP-88uncultured_delta	0.000245951	7.77765E-05	Candidatus_Magasanikbacteria__uncultured_planctomycete	0.000215626	6.81869E-05	Akkermansia_sp.	0	0	Ancalomicrobium_sp.	0.000272784	8.62619E-05
Clostridioides_sp.	0.00034380	0.00010872	Uruburuella_sp.	0.0000899975	0	Fimbriiglobus_Gemmata_sp.	0.000245951	7.77765E-05	Acholeplasma__uncultured_bacterium	0.000206859	6.54146E-05	Akkermansia_uncultured_bacterium	0	0	ASV_6422	0.000272784	8.62619E-05
Gracilibacteria_JGI_0000069-P22_uncultured_Epsilonproteobacteria	0.00034380	0.00010872	Actinobacteriota_MB-A2-108_metagenome	7.88084E-05	2.49214E-05	Rhodocyclaceae_Z-35_uncultured_bacterium	0.000245951	7.77765E-05	Chloroplast_Guillardia_theta	0.000206859	6.54146E-05	Alcaligenaceae_sp.	0	0	Fusobacterium_gut__metagenome	0.000272784	8.62619E-05
Silvanigrella_sp.	0.00034380	0.00000000	ASV_20641	7.88084E-05	2.49214E-05	ASV_17351	0.000242268	7.66119E-05	Demequina_uncultured_bacterium	0.000206859	6.54146E-05	Alcaligenes_sp.	0	0	Fimbriimonadaceae_sp.	0.00026387	6.50136E-05
Actinobacteria_0319-7L14_uncultured_actinobacterium	0.00034106	0.00010785	ASV_5322	0.00007198	2.27621E-05	ASV_17352	0.000242268	7.66119E-05	LD29_uncultured_Xiphinematobacteriaceae	0.000206859	6.54146E-05	Algibacter_sp.	0	0	Saccharimonadales_WWH38_uncultured_soil	0.000263865	8.34414E-05
Ahniella_uncultured_bacterium	0.00034106	0.00010785	Caproiciproducens_sp.	0.00007198	2.27621E-05	ASV_3630	0.000242268	7.66119E-05	ASV_2850	0.000203545	3.46359E-05	Algoriphagus_sp.	0	0	Corynebacterium_amycolatum	0.000262409	8.2981E-05
ASV_19671	0.00034106	0.00010785	Neochlamydia_sp.	0.00007198	2.27621E-05	Christensenellaceae_uncultured__bacterium	0.000236346	7.47392E-05	ASV_15298	0.000202953	6.41794E-05	Algoriphagus_uncultured_Bacteroidetes	0	0	Diplorickettsiaceae_sp.	0.000262409	8.2981E-05
Candidatus_Planktophila_sp.	0.00034106	0.00010785	Candidatus_Omnitrophus_bioreactor__metagenome	7.01016E-05	2.21681E-05	Clostridia_uncultured__sp.	0.000236346	7.47392E-05	Chloroplast_Sarcinofilum_mucosum	0.000202953	6.41794E-05	Algoriphagus_uncultured_Hongiella	0	0	Gammaproteobacteria_Ga0077536_sp.	0.000262409	8.2981E-05

DEV008_metagenome	0.00034106	0.00010785	Chitinophagales_37-13_Bacteroidetes_bacterium	7.01016E-05	2.21681E-05	Kapabacteriales_sp.	0.000236346	7.47392E-05	Clostridiales_uncultured_Bacillus	0.000202953	6.41794E-05	Aliidiomarina_uncultured_bacterium	0	0	ASV_4563	0.000259623	8.21E-05
Margulisbacteria_uncultured_Gram-positive	0.00034106	0.00010785	Naumannella_sp.	7.01016E-05	2.21681E-05	Schaalia_radingae	0.000228383	7.221E-05	Terriglobus_sp.	0.000202953	6.41794E-05	Aliidongia_dinghuensis	0	0	Pseudoramibacter_uncultured_bacterium	0.000259623	8.21E-05
Paraprevotella_clara	0.00034106	0.00010785	Planctomycetota_OM190_uncultured_bacterium	7.01016E-05	2.21681E-05	alphaproteobacteria_uncultured__bacterium	0.000226603	7.16582E-05	Treponema_zuelzerae	0.000202953	6.41794E-05	Aliivibrio_sp.	0	0	Anaerofustis_uncultured_bacterium	0.000247985	7.84197E-05
Taibaiella_sp.	0.00034106	0.00000000	Plantibacter_sp.	7.01016E-05	2.21681E-05	Oscillospiraceae_UCG-005_uncultured_rumen	0.000226603	7.16582E-05	Acetivibrio_uncultured_bacterium	0.000199566	6.31083E-05	Alishewanella_sp.	0	0	Chloroflexia_JG30-KF-CM66_metagenome	0.000247985	7.84197E-05
Thermoleophilia_uncultured_actinobacterium	0.00034106	0.00000000	Solirubrobacterales_67-14__metagenome	7.01016E-05	0	Paludibacteraceae_sp.	0.000226603	7.16582E-05	ASV_14706	0.000199566	6.31083E-05	Alishewanella_uncultured_bacterium	0	0	Clostridium_sensu_stricto_12_uncultured_Clostridium	0.000247985	7.84197E-05
Unknown_Family_sp.	0.00034106	0.00000000	ASV_21025	0.000053985	1.70716E-05	Phaselicystis_metagenome	0.000226603	7.16582E-05	Chitinophagaceae_uncultured__bacterium	0.000199566	6.31083E-05	Alistipes_gut__metagenome	0	0	Gastranaerophilales_uncultured_rumen	0.000247985	7.84197E-05
Candidatus_HepatincolaCandidatus_Hepatincola	0.00032240	0.00010195	Bdellovibrionaceae_OM27_clade_uncultured_bacterium	0.000053985	1.70716E-05	Rickettsiales_SM2D12_uncultured_bacterium	0.000226603	7.16582E-05	Flavobacteriaceae_sp.	0.000199566	6.31083E-05	Alistipes_sp.	0	0	Bifidobacterium_sp.	0.000247831	7.8371E-05
ASV_10170	0.00032118	0.00010157	Lentimicrobiaceae_wastewater__metagenome	0.000053985	1.70716E-05	Sandaracinaceae_uncultured__bacterium	0.000226603	7.16582E-05	Sphingobacteriales_FFCH9454_metagenome	0.000199566	6.31083E-05	Alistipes_uncultured_Alistipes	0	0	Deinococcus_sp.	0.000247831	7.8371E-05
Desulfomicrobium_sp.	0.00032118	0.00010157	Anaerovorax_uncultured_bacterium	5.25389E-05	1.66143E-05	Simkaniaceae_uncultured__bacterium	0.000226603	7.16582E-05	ASV_13773	0.000196997	6.22959E-05	Alistipes_uncultured_bacterium	0	0	ASV_4527	0.000242315	7.66267E-05
LiUU-11-161_sp.	0.00032118	0.00010157	Gaiellales_sp.	5.25389E-05	1.66143E-05	Solirubrobacterales_bacterium	0.000226603	7.16582E-05	ASV_13775	0.000196997	6.22959E-05	Alistipes_uncultured_Bacteroidetes	0	0	Pirellula_uncultured_soil	0.000242224	7.6598E-05
ASV_9591	0.00031925	0.00010095	Peptoniphilus_sp.	5.25389E-05	1.66143E-05	Akkermansia_uncultured_bacterium	0.000216685	6.85218E-05	Fusibacteruncultured_prokaryote	0.000196997	6.22959E-05	Aliterella_cyanobacterium_OU_20	0	0	ASV_2862	0.000234955	7.42993E-05
Chroococcidiopsaceae_uncultured__bacterium	0.00031925	0.00010095	Thermoleophilia_uncultured__metagenome	5.25389E-05	0	Chloroflexi_TK10_metagenome	0.000216685	6.85218E-05	Hirschia_sp.	0.000196997	6.22959E-05	Aliterella_sp.	0	0	Chitinophagales_uncultured__bacterium	0.000234955	7.42993E-05
ASV_2180	0.00031083	0.00009829	ASV_6240	4.67344E-05	1.47787E-05	Chloroplast_Melosira_varians	0.000216685	6.85218E-05	Oligoflexia_0319-6G20_uncultured_Myxococcales	0.000196997	6.22959E-05	Aliterella_uncultured_bacterium	0	0	Cellvibrio_uncultured_Cellvibrio	0.000230715	5.32778E-05
bacterium_enrichment	0.00031083	0.00009829	Tessaracoccus_sp.	4.67344E-05	0	Planctomycetes_Pla4_lineage_uncultured_bacterium	0.000216685	6.85218E-05	ASV_3352	0.000196792	6.22311E-05	Alkalibacter_uncultured_bacterium	0	0	Sulfuritalea_sp.	0.000228458	7.22448E-05
Exiguobacterium_undae	0.00031083	0.00009829	Bacilli_RF39_uncultured_bacterium	0.00003599	1.1381E-05	Prevotella_gut__metagenome	0.000216685	6.85218E-05	ASV_5779	0.000196792	6.22311E-05	Alkalibacterium_uncultured_bacterium	0	0	Anaerolineae_sp.	0.000225007	7.11535E-05
Halomonas_sp.	0.00031083	0.00009829	Cellulosimicrobium_sp.	0.00003599	1.1381E-05	Butyricicoccaceae_UCG-008_uncultured_bacterium	0.000216651	6.85111E-05	ASV_7664	0.000196792	6.22311E-05	Alkanibacter_metagenome	0	0	ASV_10995	0.000225007	7.11535E-05
Rhodanobacteraceae_uncultured__bacterium	0.00031083	0.00013482	Desulforegula_uncultured_bacterium	0.00003599	1.1381E-05	Novosphingobium_rosa	0.000216651	6.85111E-05	Gemmata_uncultured_bacterium	0.000196792	6.22311E-05	Alkanindiges_sp.	0	0	Paludibacteraceae_uncultured__sp.	0.000225007	7.11535E-05
Weeksellaceae_uncultured_Chishuiella_sp.	0.00031083	0.00000000	Oligoflexia_0319-6G20_uncultured_gamma	0.00003599	1.1381E-05	Gaiellales_sp.	0.000210815	6.66656E-05	Gemmataceae_Zavarzinella_uncultured_bacterium	0.000196792	6.22311E-05	Alkanindiges_uncultured_bacterium	0	0	Ferribacterium_sp.	0.000223187	7.05779E-05
DEV008_uncultured_bacterium	0.00030858	0.00009758	Salinispira_uncultured_bacterium	0.00003599	0	Botryosphaeria_dothidea	0.000208377	6.58946E-05	Haliangium_uncultured_soil	0.000196792	6.22311E-05	Alkanindiges_uncultured_gamma	0	0	ASV_11102	0.000218172	6.8992E-05
Fokiniaceae_uncultured__bacterium	0.00030858	0.00009758	009E01-B-SD-P15__uncultured_bacterium	0	0	Candidatus_Nomurabacteria_marine__metagenome	0.000208377	6.58946E-05	Muribaculaceae_uncultured_bacterium	0.000196792	6.22311E-05	Allobranchiibius_sp.	0	0	ASV_11103	0.000218172	6.8992E-05
Kapabacteriales_uncultured_bacterium	0.00030858	0.00009758	1013-28-CG33__uncultured_soil	0	0	Sphingopyxis_sp.	0.000208377	6.58946E-05	Patescibacteria_CPR2_uncultured_microorganism	0.000196792	6.22311E-05	Alloiococcus_uncultured_bacterium	0	0	ASV_3026	0.000218172	6.8992E-05
Oligoflexia_0319-6G20__uncultured_bacterium	0.00030858	0.00009758	Abditibacterium_sp.	0	0	Acetobacteraceae_uncultured__bacterium	0.000204026	6.45187E-05	Chloroplast_Pyramimonas_tetrarhynchus	0.000188058	5.94692E-05	Alloprevotella_rava	0	0	Beijerinckiaceae_1174-901-12_Rhizobiales_bacterium	0.000218172	6.8992E-05
Sphingomonadaceae_uncultured_Sphingopyxis_sp.	0.00030858	0.00000000	Absconditabacteriales_(SR1)__metagenome	0	0	Rhodococcus_sp.	0.000204026	6.45187E-05	Micropepsaceae_uncultured__bacterium	0.000188058	5.94692E-05	Alloprevotella_sp.	0	0	Psychrobacter_sanguinis	0.000218172	6.8992E-05
Acidibacter__uncultured_bacterium	0.00029824	0.00009431	Absconditabacteriales_(SR1)_candidate_division	0	0	Sphingobium_metagenome	0.000204026	6.45187E-05	Phreatobacter_sp.	0.000188058	5.94692E-05	Alloprevotella_uncultured_Bacteroidales	0	0	Verrucomicrobia_bacterium	0.000218172	6.8992E-05
Oligoflexia_sp.	0.00029824	0.00009431	Absconditabacteriales_(SR1)_sp.	0	0	ASV_18573	0.00020189	6.38432E-05	Vermiphilaceae_sp.	0.000188058	5.94692E-05	Allorhizobium-Neorhizobium-Pararhizobium-Rhizobium_sp.	0	0	ASV_10179	0.00021173	6.87163E-05
WPS-2_sp.	0.00029824	0.00000000	Absconditabacteriales_(SR1)_SR1_bacterium	0	0	Leptotrichia_shahii	0.000200123	5.2122E-05	Cutibacterium_granulosum	0.000186495	4.19231E-05	allotenuales_AKIW781_uncultured_bacterium	0	0	ASV_1592	0.000214619	4.53321E-05
Pajaroellobacteruncultured_Polyangiaceae	0.00029553	0.00009346	Absconditabacteriales_(SR1)_uncultured_candidate	0	0	Chloroflexia_JG30-KF-CM70_uncultured_soil	0.000196955	6.22826E-05	Isosphaeraceae_uncultured__bacterium	0.000182698	5.77742E-05	alpha_proteobacterium	0	0	Actinomycetaceae_F0332unidentified	0.000207699	6.56802E-05
ASV_21009	0.00028542	0.00009026	Absconditabacteriales_(SR1)_uncultured_Epsilonproteobacteria	0	0	Lactobacillus_mucosae	0.000193247	6.11101E-05	Armatimonadia_sp.	0.000177584	5.6157E-05	Alphaproteobacteria_bacterium	0	0	Campylobacter_concisus	0.000207699	6.56802E-05
Caldilineaceae_uncultured__bacterium	0.00028542	0.00009026	Absconditabacteriales_(SR1)_uncultured_organism	0	0	Chloroflexia_JG30-KF-CM69_uncultured_soil	0.00017726	5.60545E-05	Mucispirillum_sp.	0.000177584	5.6157E-05	Alphaproteobacteria_sp.	0	0	ASV_2249	0.000203314	6.42935E-05
Staphylococcus_sp.	0.00028542	0.00000000	Absconditabacteriales_(SR1)_uncultured_prokaryote	0	0	Haliscomenobacter_uncultured_Haliscomenobacter	0.00017726	5.60545E-05	Paludisphaera_uncultured_bacterium	0.000175108	5.5374E-05	alphaproteobacteria_uncultured__bacterium	0	0	Candidatus_Ovatusbacter_sp.	0.000203314	6.42935E-05

Chitinophaga_sp.	0.00028524	0.00006156	ABY1_sp.	0	0	Ruminiclostridium_metagenome	0.00017726	5.60545E-05	Rhodanobacter_sp.	0.000175108	5.5374E-05	Alphaproteobacteria_uncultured__bacterium	0	0	ASV_12862	0.000201853	6.38315E-05
ASV_3443	0.00028331	0.00008959	Acanthopleuribacter__uncultured_bacterium	0	0	Leptotrichia_hofstadii	0.000164987	5.21735E-05	ASV_15707	0.00017462	5.52197E-05	Alphaproteobacteria_uncultured__metagenome	0	0	Bradymonadalesuncultured_organism	0.000201853	6.38315E-05
Fibrobacterales_B18_uncultured_bacterium	0.00028331	0.00008959	Acetitomaculum__uncultured_bacterium	0	0	Saccharimonadaceae_uncultured_bacterium	0.000164987	5.21735E-05	Pararheinheimera_soli	0.00017462	5.52197E-05	Alphaproteobacteria_uncultured__sp.	0	0	Devosiaceae_uncultured__sp.	0.000201853	6.38315E-05
Hungateiclostridiaceae_uncultured__metagenome	0.00028331	0.00008959	Acetitomaculum__uncultured_rumen	0	0	Candidatus_Omnitrophus_Omnitrophica_bacterium	0.000161512	5.10746E-05	Achromatium_sp.	0.000165488	5.23319E-05	Alphaproteobacteria_uncultured_Alphaproteobacteria	0	0	Solibacillus_sp.	0.00020139	6.36851E-05
Microtrichaceae_IMCC26207_uncultured_bacterium	0.00028331	0.00008959	Acetitomaculum_sp.	0	0	Rhodovastum_metagenome	0.000161512	5.10746E-05	ASV_19509	0.000165488	5.23319E-05	Alphaproteobacteria_uncultured_bacterium_enrichment	0	0	Aeromonas_uncultured_gamma	0.000198388	6.27358E-05
Rhodanobacteraceae_sp.	0.00028331	0.00009829	Acetivibrio_uncultured_bacterium	0	0	Candidatus_Ovatusbacter_metagenome	0.000158111	4.99991E-05	ASV_19510	0.000165488	5.23319E-05	Alphaproteobacteria_uncultured_eubacterium	0	0	Clostridium_sensu_stricto_1_metagenome	0.000198388	6.27358E-05
Rs-M47_uncultured_bacterium	0.00028331	0.00000000	Acetoanaerobium_uncultured_bacterium	0	0	Methanosphaera_sp.	0.000158111	4.99991E-05	Bdellovibrio_unidentified	0.000165488	5.23319E-05	Alphaproteobacteria_uncultured_gut__metagenome	0	0	Lentimicrobiaceae_wastewater__metagenome	0.000198388	6.27358E-05
Solirubrobacterales_67-14__uncultured_soil	0.00028331	0.00004377	Acetobacter_sp.	0	0	Opitutaceae_IMCC26134Verrucomicrobia_bacterium	0.000158111	4.99991E-05	Beggiatoa_uncultured_bacterium	0.000165488	5.23319E-05	Alphaproteobacteria_uncultured_Lacibacterium_aquatile	0	0	Candidatus_Iainarchaeumuncultured_archaeon	0.00019039	6.02066E-05
Thauera_sp.	0.00028331	0.00000000	Acetobacteraceae_sp.	0	0	ASV_14663	0.000157564	4.98261E-05	Oligoflexus_sp.	0.000165488	5.23319E-05	Alphaproteobacteria_uncultured_Novosphingobium	0	0	Gracilibacteria_JGI_0000069-P22_metagenome	0.00019039	6.02066E-05
ASV_1161	0.00027686	0.00008755	Acetobacteraceae_uncultured__bacterium	0	0	ASV_4322	0.000157564	4.98261E-05	Pirellulaceae_uncultured_Planctomycetaceae	0.000165488	5.23319E-05	Alphaproteobacteria_uncultured_Rhodospirillaceae_bacterium	0	0	Geothrix_sp.	0.000190382	6.02041E-05
Gemmataceae_uncultured_sludge	0.00027686	0.00008755	Acetobacteraceae_uncultured__metagenome	0	0	Prevotella_nanceiensis	0.000157564	4.98261E-05	Sedimentibacter_sp.	0.000165488	5.23319E-05	Altererythrobacter_alpha_proteobacterium	0	0	Psychrobacillus_sp.	0.000190382	6.02041E-05
Saccharimonadales_TM7a_Candidatus_Saccharibacteria	0.00027686	0.00000000	Acetobacteraceae_uncultured__sp.	0	0	Allorhizobium-Neorhizobium-Pararhizobium-Rhizobium_sp.	0.000151069	4.77722E-05	Sericytochromatiauncultured_prokaryote	0.000165488	5.23319E-05	Altererythrobacter_sp.	0	0	ASV_11399	0.000186257	5.88996E-05
ASV_11393	0.00027530	0.00008706	Acetothermiia__uncultured_bacterium	0	0	Gemmataceae_uncultured__bacterium	0.000151069	4.77722E-05	Luteitalea_sp.	0.000155948	4.93151E-05	Alysiella_sp.	0	0	Bacteroidales_genomosp.	0.000185615	5.86966E-05
Olsenella_uncultured_bacterium	0.00027530	0.00008706	Acholeplasma__uncultured_bacterium	0	0	Kocuria_marina	0.000151069	4.77722E-05	Nitrososphaeraceae_sp.	0.000155948	4.93151E-05	Alysiella_uncultured_bacterium	0	0	Diplorickettsiaceae_uncultured__bacterium	0.000185615	5.86966E-05
Perlucidibaca_sp.	0.00027530	0.00008706	Acholeplasma_axanthum	0	0	Micavibrionaceae_uncultured_Alphaproteobacteria	0.000151069	4.77722E-05	ASV_15770	0.00015322	4.84524E-05	Amaricoccus_sp.	0	0	Flavobacterium_sp.	0.000185615	5.86966E-05
Prevotellauncultured_Bacteroidetes	0.00027530	0.00008706	Acholeplasma_brassicae	0	0	SaccharimonadalesTM7_phylum	0.000151069	4.77722E-05	ASV_15776	0.00015322	4.84524E-05	Amaricoccus_tamworthensis	0	0	Gastranaerophilales_uncultured_organism	0.000185615	5.86966E-05
Methylopilaceae_sp.	0.00027013	0.00008542	Acholeplasma_laidlawii	0	0	Saccharimonadia_sp.	0.000151069	4.77722E-05	Caulobacteraceae_uncultured__metagenome	0.00015322	4.84524E-05	Amb-16S-1323_uncultured_bacterium	0	0	Haloplasma_uncultured_bacterium	0.000185615	5.86966E-05
Dialister_Veillonellaceae_bacterium	0.00026867	0.00008496	Acholeplasma_morum	0	0	Solirubrobacterales_67-14_uncultured_Conexibacter	0.000151069	4.77722E-05	Gaiella_uncultured_Conexibacteraceae	0.00015322	4.84524E-05	Aminobacter_sp.	0	0	Paracaedibacteraceae_uncultured__sp.	0.000185615	5.86966E-05
Taibaiella_metagenome	0.00026867	0.00010785	Acholeplasma_sp.	0	0	Tepidimonas_sp.	0.000151069	4.77722E-05	RsaHf231_uncultured_bacterium	0.00015322	4.84524E-05	Ammoniibacillus_agariperforans	0	0	ASV_4143	0.000184607	5.83779E-05
Candidatus_Komeilibacteria_sp.	0.00026572	0.00008403	Acholeplasmauncultured_Acholeplasmataceae	0	0	Woesearchaeales_metagenome	0.000151069	4.77722E-05	Labrys_sp.	0.000152215	4.81346E-05	Ammoniphilus_oxalaticus	0	0	Azovibrio_uncultured_bacterium	0.000184607	5.83779E-05
Chloroplast_Carteria_sp.	0.00026572	0.00008403	Achromatium_sp.	0	0	ASV_7664	0.000144456	4.5681E-05	Malikia_uncultured_bacterium	0.000152215	4.81346E-05	Amnibacterium_sp.	0	0	ASV_1901	0.000182724	3.50397E-05
Desulfovibrio_sp.	0.00026572	0.00008403	Acidaminobacter__uncultured_bacterium	0	0	Prevotella_paludivivens	0.000144456	4.5681E-05	ASV_2433	0.000149674	4.73311E-05	Amycolatopsis_sp.	0	0	ASV_13728	0.000181668	5.74485E-05
Marinimicrobia_(SAR406_clade)_metagenome	0.00026572	0.00008403	Acidaminococcaceae_uncultured__bacterium	0	0	Acetobacteraceae_uncultured__metagenome	0.000140543	4.44436E-05	Beijerinckiaceae_1174-901-12_uncultured_Alphaproteobacteria	0.000149674	4.73311E-05	Anaerobacterium_uncultured_bacterium	0	0	Berkelbacteria_Candidatus_Berkelbacteria	0.00017359	5.4894E-05
ASV_1837	0.00026528	0.00006350	Acidibacter__metagenome	0	0	ASV_14729	0.000140543	4.44436E-05	Holosporaceae_uncultured__sp.	0.000149674	4.73311E-05	Anaerobiospirillum_succiniciproducens	0	0	Dyadobacter_fermentans	0.00017359	5.4894E-05
Arthrobacter_sp.	0.00025580	0.00008089	Acidibacter__uncultured_bacterium	0	0	Bauldia_metagenome	0.000140543	4.44436E-05	Synergistaceae_uncultured_bacterium_enrichment	0.000149674	4.73311E-05	Anaerococcus_hydrogenalis	0	0	Lachnospiraceae_NK3A20_groupunidentified_rumen	0.00017359	5.4894E-05
Candidatus_Lloydbacteria_sp.	0.00025580	0.00008089	Acidibacter_sp.	0	0	Lentimicrobiaceae_sp.	0.000140543	4.44436E-05	Vicinamibacteria_uncultured_Acidobacteria	0.000149674	4.73311E-05	Anaerococcus_octavius	0	0	Spirochaetaceae_uncultured_uncultured_Treponema	0.000170736	5.39915E-05
Candidatus_Rhabdochlamydia	0.00025580	0.00008089	Acidimicrobiia_IMCC26256_metagenome	0	0	Pseudomonas_caeni	0.000140543	4.44436E-05	ASV_3580	0.00014375	4.54577E-05	Anaerococcus_prevotii	0	0	Subgroup_10_sp.	0.000167825	5.30709E-05
Chlamydiales_cvE6_uncultured_bacterium	0.00025580	0.00008089	Acidimicrobiia_IMCC26256_sp.	0	0	Woesearchaeales_SCGC_AAA011-D5_sp.	0.000138918	4.39297E-05	Bdellovibrio_metagenome	0.00014375	4.54577E-05	Anaerococcus_sp.	0	0	Thioclava_uncultured_bacterium	0.000167825	5.30709E-05
Christensenellaceae_R-7_groupuncultured_prokaryote	0.00025580	0.00008089	Acidimicrobiia_IMCC26256_uncultured_bacterium	0	0	ASV_15672	0.000137869	4.3598E-05	Chloroplast_metagenome	0.00014375	4.54577E-05	Anaerococcus_uncultured_bacterium	0	0	Armatimonadota_uncultured__sp.	0.000165221	5.22475E-05
Fimbriiglobusuncultured_Gemmata	0.00025580	0.00008089	Acidimicrobiia_IMCC26256_uncultured_soil	0	0	Calothrix_KVSF5_uncultured_bacterium	0.000137869	4.3598E-05	Parcubacteria_UBA9983_sp.	0.00014375	4.54577E-05	Anaerococcusuncultured_organism	0	0	Chlamydiales_cvE6_metagenome	0.000165221	5.22475E-05

Flavobacterium_unidentified	0.00025580	0.00008089	Acidimicrobiia_IMCC26256uncultured_Aciditerrimonas	0	0	Candidatus_Microthrix_sp.	0.000137869	4.3598E-05	Ruminococcaceae_uncultured_Ruminococcaceae	0.00014375	4.54577E-05	Anaerocolumna_uncultured_bacterium	0	0	Oligoflexia_0319-6G20_uncultured_proteobacterium	0.000165221	5.22475E-05
Lachnospiraceae_UCG-010_uncultured_bacterium	0.00025580	0.00008089	Acidimicrobiia_IMCC26256uncultured_actinobacterium	0	0	Candidatus_Planktophila_sp.	0.000137869	4.3598E-05	ASV_2285	0.000131331	4.15305E-05	Anaerofilumuncultured_Anaerofilum	0	0	Pelosinus_sp.	0.000165221	5.22475E-05
LD29_uncultured_bacterium	0.00025580	0.00008089	Acidimicrobiia_IMCC26256uncultured_Actinomycetales	0	0	Gemmataceae_uncultured_Planctomyceta	0.000137869	4.3598E-05	Anaerovoracaceae_Family_XIII_AD3011_group_uncultured_bacterium	0.000126846	4.01122E-05	Anaerofustis_uncultured_bacterium	0	0	Intestinimonas_Clostridium_sp.	0.000161482	5.10651E-05
Microtrichaceae_uncultured__sp.	0.00025580	0.00008089	Acidimicrobiia_sp.	0	0	ASV_21599	0.000136017	4.30124E-05	ASV_18878	0.000126846	4.01122E-05	Anaeroglobus_geminatus	0	0	Lentimicrobiaceae_uncultured_Sphingobacteriales	0.000161482	5.10651E-05
Rickettsiaceae_sp.	0.00025580	0.00000000	Acidimicrobiia_uncultured__bacterium	0	0	Candidatus_Udaeobacter_sp.	0.000136017	4.30124E-05	Methanobacterium_sp.	0.000126846	4.01122E-05	Anaerolineaceae_bacterium	0	0	Saccharibacillus_sp.	0.000161482	5.10651E-05
Sphingobacteriales_env.OPS_17_metagenome	0.00025580	0.00000000	Acidimicrobiia_uncultured__metagenome	0	0	Corynebacterium_aurimucosum	0.000136017	4.30124E-05	Rurimicrobium_uncultured_bacterium	0.000126846	4.01122E-05	Anaerolineaceae_sp.	0	0	ASV_14140	0.000155774	4.92601E-05
Verrucomicrobiaceae_uncultured__sp.	0.00025580	0.00000000	Acidimicrobiia_uncultured__sp.	0	0	Lachnospira_sp.	0.000136017	4.30124E-05	Cellulosimicrobium_sp.	0.000124758	3.94519E-05	Anaerolineaceae_UCG-001_uncultured_bacterium	0	0	Actinobacteria_PeM15uncultured_actinobacterium	0.000155214	4.9083E-05
Armatimonadota_uncultured__bacterium	0.00025236	0.00007980	Acidimicrobiia_uncultured_Acidimicrobiia	0	0	Telmatocola_metagenome	0.000136017	4.30124E-05	Latescibacterota_sp.	0.000124758	3.94519E-05	Anaerolineaceae_uncultured__bacterium	0	0	Azospira_uncultured_Rhodocyclaceae	0.000152306	4.81634E-05
Formivibrio_sp.	0.00025236	0.00007980	Acidimicrobiia_uncultured_actinobacterium	0	0	Woesearchaeales_SCGC_AAA011-D5_uncultured_euryarchaeote	0.000136017	4.30124E-05	Legionella_pneumophila	0.000124758	3.94519E-05	Anaerolineaceae_uncultured__metagenome	0	0	Cavicella_sp.	0.000152306	4.81634E-05
NannocystaceaeEnhygromyxa_sp.	0.00025236	0.00007980	Acidimicrobiia_uncultured_Actinomycetales	0	0	ASV_5084	0.000134621	2.86441E-05	Burkholderiales_B1-7BS_uncultured_bacterium	0.000124729	3.94428E-05	Anaerolineaceae_uncultured__sp.	0	0	Fokiniaceae_uncultured__bacterium	0.000152306	4.81634E-05
Aurantimicrobium_sp.	0.00024866	0.00007863	Acidimicrobiia_uncultured_bacterium_YC-ZSS-LKJ199	0	0	ASV_12525	0.000129633	4.09936E-05	Hungateiclostridiaceae_uncultured_bacterium	0.000124729	3.94428E-05	Anaerolineaceae_uncultured__uncultured_soil	0	0	Ilumatobacter_sp.	0.000152306	4.81634E-05
Eubacterium_coprostanoligenes_group_sp.	0.00024866	0.00007863	Acidiphilium__uncultured_bacterium	0	0	ASV_15737	0.000122975	3.88881E-05	Acidobacteria_bacterium	0.000124116	3.92489E-05	Anaerolineaceae_uncultured_Bellilinea	0	0	Rhodocyclaceae_uncultured__metagenome	0.000152306	4.81634E-05
Ferruginibacter_uncultured_bacterium	0.00024866	0.00007863	Acidiphilium_sp.	0	0	bacterium_UW	0.000122975	3.88881E-05	Acidobacteriae_sp.	0.000124116	3.92489E-05	Anaerolineaceae_uncultured_delta	0	0	Treponema_denticola	0.000151042	4.77637E-05
Ga0077536_sp.	0.00024866	0.00007863	Acidisoma_sp.	0	0	Candidatus_Hepatincola_uncultured_bacterium	0.000122975	3.88881E-05	ASV_20677	0.000124116	3.92489E-05	Anaerolineaceae_uncultured_eubacterium	0	0	Micavibrionaceae_uncultured__sp.	0.000148791	4.70518E-05
Nocardioides_sp.	0.00024866	0.00007863	Acidithiobacillaceae_KCM-B-112_sp.	0	0	Neisseriaceae_uncultured__sp.	0.000122975	3.88881E-05	ASV_4527	0.000124116	3.92489E-05	Anaerolineaceae_uncultured_Longilinea	0	0	Parasutterella_sp.	0.000148791	4.70518E-05
Psychrobacter_pulmonis	0.00024866	0.00075498	Acidithiobacillus_ferrooxidans	0	0	ASV_21002	0.000121134	3.83059E-05	Methylomonadaceae_pLW-20_uncultured_soil	0.000124116	3.92489E-05	Anaerolineaceae_uncultured_sludge	0	0	Roseomonas_aquatica	0.000148791	4.70518E-05
Kineosporia_sp.	0.00024557	0.00007766	Acidobacteria_bacterium	0	0	ASV_2433	0.000121134	3.83059E-05	Microtrichaceae_IMCC26207_sp.	0.000124116	3.92489E-05	Anaerolineaceae_uncultured_toluene-degrading_methanogenic	0	0	Candidatus_Limnoluna_uncultured_bacterium	0.00014422	3.38862E-05
Pusillimonasuncultured_Pusillimonas	0.00024557	0.00000000	Acidobacteriaceae_(Subgroup_1)_sp.	0	0	Nitzschia_sp.	0.000121134	3.83059E-05	Pedosphaeraceae_sp.	0.000124116	3.92489E-05	Anaerolineae_1-20_sp.	0	0	Spirochaetaceae_M2PT2-76_termite_group_uncultured_bacterium	0.000141297	4.4682E-05
Shuttleworthia_uncultured_bacterium	0.00024557	0.00000000	Acidobacteriae_Elev-16S-1166_sp.	0	0	Nostocoida_limicola	0.000121134	3.83059E-05	Peredibacter_uncultured_bacterium	0.000124116	3.92489E-05	Anaerolineae_1-20uncultured_Chloroflexia	0	0	Tepidisphaeraceae_uncultured_bacterium	0.000141297	4.4682E-05
Acidobacteriaceae_(Subgroup_1)_sp.	0.00024180	0.00007646	Acidobacteriae_Elev-16S-1166_uncultured_bacterium	0	0	Reyranella_sp.	0.000121134	3.83059E-05	Polyangia_mle1-27_uncultured_bacterium	0.000124116	3.92489E-05	Anaerolineae_A4b__metagenome	0	0	ASV_15102	0.000138466	4.37868E-05
Pseudarcobacter_uncultured_Arcobacter	0.00024180	0.00000000	Acidobacteriae_sp.	0	0	Afipia_sp.	0.000118173	3.73696E-05	Ruminococcaceae_uncultured__bacterium	0.000124116	3.92489E-05	Anaerolineae_A4b__uncultured_bacterium	0	0	Cephaloticoccusuncultured_Verrucomicrobia	0.000138466	4.37868E-05
Tsukamurella_paurometabola	0.00024180	0.00000000	Acidocella__uncultured_bacterium	0	0	ASV_4527	0.000118173	3.73696E-05	Saccharimonadales_WWH38_sp.	0.000124116	3.92489E-05	Anaerolineae_A4b__uncultured_soil	0	0	Edaphobaculum_uncultured_Bacteroidetes	0.000138466	4.37868E-05
Brevundimonas_aveniformis	0.00022942	0.00007255	Acidothermus__uncultured_bacterium	0	0	Hydrogenophilaceae_sp.	0.000118173	3.73696E-05	Thermus_sp.	0.000124116	3.92489E-05	Anaerolineae_A4b_sp.	0	0	Desulfobacterota_sp.	0.00013426	4.24567E-05
Chloroplast_Guillardia_theta	0.00022942	0.00007255	Acidovorax_sp.	0	0	MidBa8_uncultured_bacterium	0.000118173	3.73696E-05	Actinobacteria_uncultured__sp.	0.000109443	3.46089E-05	Anaerolineae_A4b_wastewater__metagenome	0	0	TelmatocolaTelmatocola_sphagniphila	0.00013426	4.24567E-05
Blrii41_uncultured_bacterium	0.00022102	0.00006989	Acinetobacter__metagenome	0	0	Capnocytophaga_haemolytica	0.000117848	3.72668E-05	Anaerococcusuncultured_organism	0.000109443	3.46089E-05	Anaerolineae_sp.	0	0	ASV_3442	0.000130237	3.18101E-05
Flavobacterium_kingsejongi	0.00022102	0.00006989	Acinetobacter_baumannii	0	0	PaludisphaeraIsosphaera_sp.	0.000117848	3.72668E-05	ASV_2249	0.000109443	3.46089E-05	Anaerolineae_uncultured__bacterium	0	0	Flavobacterium_tegetincola	0.000124172	3.92666E-05
Sulfurimonas_sp.	0.00022102	0.00000000	Acinetobacter_kyonggiensis	0	0	Candidatus_Endoecteinascidia_uncultured_gamma	0.000105407	3.33326E-05	ASV_3026	0.000109443	3.46089E-05	Anaeromusa-Anaeroarcus_sp.	0	0	Gaiellales_sp.	0.000124172	3.92666E-05
Anaerocolumna_uncultured_bacterium	0.00020764	0.00006566	Acinetobacter_sp.	0	0	Paludibacteraceae_uncultured_marine__metagenome	0.000105407	3.33326E-05	Chlamydiales_sp.	0.000109443	3.46089E-05	Anaeromyxobacter_dehalogenans	0	0	ASV_18208	0.000123993	3.921E-05
Berkelbacteria_metagenome	0.00020764	0.00006566	Acinetobacter_towneri	0	0	Caulobacter_metagenome	9.84775E-05	3.11413E-05	Clostridia_UCG-014_gut__metagenome	0.000109443	3.46089E-05	Anaeromyxobacter_uncultured_bacterium	0	0	ASV_10385	0.000121158	3.83135E-05
Lactobacillus_iners	0.00020764	0.00006566	Acremonium_fuci	0	0	Conexibacter_sp.	9.84775E-05	3.11413E-05	Kocuria_marina	0.000109443	3.46089E-05	Anaerorhabdus_furcosa_group__uncultured_bacterium	0	0	Candidatus_Ovatusbacter_uncultured_proteobacterium	0.000121158	3.83135E-05
Leptotrichia_wadei	0.0002	0.0000	Actinobacillus_sp.	0	0	Eubacterium_siraeum_group_sp.	9.84775	3.11413	Myxococcaceae_sp.	0.00010	3.46089	Anaerosinus_uncultured_bacterium	0	0	Gammaproteobacteria_CHAB-XI-	0.00012	3.83135

	0764	6566					E-05	E-05		9443	E-05				27_uncultured_bacterium	1158	E-05
ASV_13765	0.00020647	0.00006529	Actinobacteria_0319-7L14_uncultured_actinobacterium	0	0	Neisseriaceae_uncultured_marine	9.84775E-05	3.11413E-05	SandarakinorhabdusSandarakinorhabdus_sp.	0.000109443	3.46089E-05	Anaerostipes_sp.	0	0	Mycoplasmataceae_genomosp.	0.000121158	3.83135E-05
SalinispirillumSalinispirillum_sp.	0.00020647	0.00000000	Actinobacteria_PeM15_bacterium_rJ7	0	0	Prevotella_marshii	9.42785E-05	2.98135E-05	Berkelbacteria_uncultured_bacterium	0.000101476	3.20895E-05	Anaerotignum_lactatifermentans	0	0	Patescibacteria_WWE3uncultured_Alphaproteobacteria	0.000121158	3.83135E-05
Candidatus_Nomurabacteria_uncultured_bacterium	0.00020572	0.00006505	Actinobacteria_PeM15_metagenome	0	0	Aerococcus_sp.	8.78395E-05	2.77773E-05	Candidatus_Adlerbacteria_metagenome	0.000101476	3.20895E-05	Anaerovibrio_sp.	0	0	Treponema_metagenome	0.000121158	3.83135E-05
Methanobrevibacter_sp.	0.00020572	0.00006505	Actinobacteria_PeM15_sp.	0	0	Ahniella_affigens	8.78395E-05	2.77773E-05	Moraxellaceae_uncultured__sp.	0.000101476	3.20895E-05	Anaerovoracaceae_Family_XIII_AD3011_group_sp.	0	0	ASV_16882	0.000121112	3.8299E-05
ASV_14678	0.00019646	0.00006213	Actinobacteria_PeM15_uncultured_soil	0	0	ASV_9492	8.78395E-05	2.77773E-05	Phaeodactylibacter_uncultured_bacterium	0.000101476	3.20895E-05	Anaerovoracaceae_Family_XIII_AD3011_group_uncultured_bacterium	0	0	Candidatus_Aquiluna_sp.	0.000121112	3.8299E-05
Hydrogenophaga_sp.	0.00019646	0.00006213	Actinobacteria_PeM15uncultured_actinobacterium	0	0	Babeliaceae_uncultured_bacterium	8.78395E-05	2.77773E-05	Sphingobium_sp.	0.000101476	3.20895E-05	Anaerovoracaceae_Family_XIII_AD3011_group_uncultured_rumen	0	0	Rickettsiales_AB1_sp.	0.000121112	3.8299E-05
Marinilabiliaceae_uncultured__bacterium	0.00019646	0.00006213	Actinobacteria_sp.	0	0	Nitrosomonadaceae_uncultured__bacterium	8.24937E-05	2.60868E-05	Bradymonadales_uncultured_rumen	0.000099783	3.15542E-05	Anaerovoracaceae_Family_XIII_AD3011_groupuncultured_Clostridiales	0	0	Spirosoma_agri	0.000121112	3.8299E-05
Candidatus_Zambryskibacteria_Parcubacteria_group	0.00019028	0.00006017	Actinobacteria_uncultured__bacterium	0	0	Peptococcus_sp.	8.24937E-05	2.60868E-05	Coxiella_sp.	0.000099783	3.15542E-05	Anaerovoracaceae_Family_XIII_UCG-007Peptostreptococcaceae_bacterium	0	0	ASV_3443	0.00012096	2.63308E-05
Candidatus_Peregrinibacteria_uncultured_bacterium	0.00018888	0.00005973	Actinobacteria_uncultured__metagenome	0	0	Candidatus_Peribacteria_metagenome	8.07559E-05	2.55373E-05	RCP2-54hydrothermal_vent	0.000099783	3.15542E-05	Anaerovoracaceae_sp.	0	0	Amnibacterium_sp.	0.000117477	3.71495E-05
Leeia_uncultured_bacterium	0.00018888	0.00005973	Actinobacteria_uncultured__sp.	0	0	Kiritimatiellae_WCHB1-41uncultured_verrucomicrobium	8.07559E-05	2.55373E-05	Rickettsiaceae_uncultured_Alphaproteobacteria	0.000099783	3.15542E-05	Anaerovoracaceae_uncultured__bacterium	0	0	ASV_16398	0.000117477	3.71495E-05
Pajaroellobacter_metagenome	0.00018888	0.00005973	Actinobacteria_uncultured_Actinomycetales_bacterium	0	0	Alistipes_uncultured_bacterium	0.000078782	2.49131E-05	ASV_4322	9.40291E-05	2.97346E-05	Anaerovorax_sp.	0	0	ASV_3630	0.000117477	3.71495E-05
Plasticicumulans_lactativorans	0.00018807	0.00005947	Actinobacteriota_MB-A2-108_sp.	0	0	ASV_19357	0.000078782	2.49131E-05	Bdellovibrio_uncultured_deep-sea	9.35687E-05	2.9589E-05	Anaerovorax_uncultured_bacterium	0	0	Blastocatellia_11-24_sp.	0.000117477	3.71495E-05
Absconditabacteriales_(SR1)_uncultured_Epsilonproteobacteria	0.00018650	0.00005898	Actinobacteriota_MB-A2-108_uncultured_bacterium	0	0	Negativicutes_sp.	0.000078782	2.49131E-05	Candidatus_Omnitrophus_uncultured_bacterium	9.35687E-05	2.9589E-05	Anaerovoraxuncultured_prokaryote	0	0	Flavobacteriaceae_bacterium	0.000117477	3.71495E-05
ASV_3568	0.00018650	0.00005898	Actinobacteriota_MB-A2-108uncultured_actinobacterium	0	0	Rathayibacter_sp.	0.000078782	2.49131E-05	Chryseobacterium_sp.	9.35687E-05	2.9589E-05	Ancalomicrobium_sp.	0	0	Pseudarcobacter_uncultured_Arcobacter	0.000117477	3.71495E-05
ASV_3639	0.00018650	0.00005898	Actinomadura_sp.	0	0	Thaumarchaeota_archaeon	0.000078782	2.49131E-05	Eubacterium_brachy	9.35687E-05	2.9589E-05	Aneurinibacillus_uncultured_bacterium	0	0	ASV_2061	0.000114229	3.61224E-05
Rikenellaceae_sp.	0.00018650	0.00000000	Actinomyces__uncultured_bacterium	0	0	Woesearchaeales_AR15uncultured_euryarchaeote	0.000078782	2.49131E-05	Flexibacter_sp.	9.35687E-05	2.9589E-05	Angustibacter_uncultured_bacterium	0	0	ASV_21015	0.000114229	3.61224E-05
Senegalimassilia_uncultured_bacterium	0.00018650	0.00000000	Actinomyces_dentalis	0	0	Blyi10_metagenome	7.07089E-05	2.23601E-05	Afipia_sp.	8.75542E-05	2.76871E-05	Anoxybacillus_sp.	0	0	Leptotrichia_sp.	0.000114229	3.61224E-05
ASV_14736	0.00018353	0.00005804	Actinomyces_graevenitzii	0	0	Brevundimonas_sp.	7.02716E-05	2.22218E-05	ASV_19457	8.75542E-05	2.76871E-05	Antarctic_bacterium	0	0	ASV_17401	0.000103849	3.28399E-05
Blastocatella_uncultured_bacterium	0.00018353	0.00005804	Actinomyces_israelii	0	0	Clostridia_UCG-014_uncultured_rumen	7.02716E-05	2.22218E-05	ASV_2061	8.75542E-05	2.76871E-05	Antricoccus_suffuscus	0	0	Gammaproteobacteria_CHAB-XI-28_uncultured_bacterium	0.000103849	3.28399E-05
Paenibacillus_barengoltzii	0.00018353	0.00005804	Actinomyces_sp.	0	0	Leptolinea_uncultured_bacterium	7.02716E-05	2.22218E-05	Eubacteriaceae_uncultured__bacterium	8.75542E-05	2.76871E-05	Apibacter_sp.	0	0	Parvibaculum_sp.	0.000103849	3.28399E-05
Chlamydiales_cvE6_sp.	0.00017715	0.00005602	Actinomyces_unidentified	0	0	Rickettsiella_sp.	7.02716E-05	2.22218E-05	Longimicrobiaceae_uncultured_bacterium	8.75542E-05	2.76871E-05	Aquabacterium_sp.	0	0	Rubinisphaeraceae_SH-PL14uncultured_Planctomycetaceae	0.000103849	3.28399E-05
Simkaniaceae_uncultured_Chlamydiales	0.00017715	0.00000000	Actinomycetaceae_F0332_uncultured_bacterium	0	0	Solobacterium_uncultured_rumen	7.02716E-05	2.22218E-05	Verrucomicrobiaceae_uncultured__metagenome	8.75542E-05	2.76871E-05	Aquaspirillum_putridiconchylium	0	0	ASV_5779	0.000101657	3.21468E-05
Calothrix_PCC-6303_Calothrix_sp.	0.00017190	0.00005436	Actinomycetaceae_F0332unidentified	0	0	Chitinophagaceae_uncultured__bacterium	6.80087E-05	2.15062E-05	ASV_2303	8.27438E-05	2.61659E-05	Aquaspirillum_sp.	0	0	ASV_4909	0.000100927	3.19159E-05
Candidatus_Pacebacteria_uncultured_bacterium	0.00017190	0.00005436	Actinomycetaceae_uncultured__bacterium	0	0	ASV_20561	5.90865E-05	1.86848E-05	Hylemonella_sp.	8.27438E-05	2.61659E-05	Aquicella_metagenome	0	0	CR-115_sp.	0.000100695	3.18426E-05
Corynebacterium_maris	0.00017190	0.00005436	Actinomycetaceae_uncultured__metagenome	0	0	ASV_2080	5.90865E-05	1.86848E-05	Mitochondria_Eukaryota_sp.	8.27438E-05	2.61659E-05	Aquicella_sp.	0	0	Lysinibacillus_sp.	0.000100695	3.18426E-05
Oligoflexia_0319-6G20_uncultured_microorganism	0.00017190	0.00005436	Actinomycetaceae_uncultured__rumen	0	0	Nannocystis_metagenome	5.90865E-05	1.86848E-05	Sandaracinaceae_uncultured_delta	8.27438E-05	2.61659E-05	Aquicella_uncultured_bacterium	0	0	Anaerolineae_A4b__uncultured_bacterium	0.000099194	3.13679E-05
Selenomonas_sp.	0.00017190	0.00000000	Actinomycetaceae_uncultured__sp.	0	0	Parachlamydiaceae_sp.	5.90865E-05	1.86848E-05	Solitalea_uncultured_bacterium	8.27438E-05	2.61659E-05	Aquicella_unidentified	0	0	ASV_19506	0.000099194	3.13679E-05
ASV_21678	0.00017053	0.00005393	Actinomycetospora_sp.	0	0	bacterium_PS7	0.000058924	1.86334E-05	Sphingobacteriales_AKYH767_sp.	8.27438E-05	2.61659E-05	Aquihabitans_daechungensis	0	0	Candidatus_Captivus_uncultured_bacterium	0.000099194	3.13679E-05
Bacteroides_vulgatus	0.00017053	0.00005393	Actinoplanes_garbadinensis	0	0	Chlorella_pyrenoidosa	0.000058924	1.86334E-05	ASV_21242	7.61074E-05	2.40673E-05	Aquisphaera_sp.	0	0	Clostridia_UCG-014_uncultured_Acetivibrio	0.000099194	3.13679E-05
Candidatus_Zambryskibacteria_uncultured_bacterium	0.00017053	0.00005393	Actinoplanes_sp.	0	0	Fusobacterium_necrophorum	0.000058924	1.86334E-05	ASV_4563	7.61074E-05	2.40673E-05	Aquisphaera_uncultured_bacterium	0	0	Gammaproteobacteria_R7C24_sp.	0.000099194	3.13679E-05

Cellvibrionaceae_uncultured__bacterium	0.00016120	0.00005098	Actinotalea_sp.	0	0	Johnsonella_ignava	0.000058924	1.86334E-05	Blvii28_wastewater-sludge_group_uncultured_bacterium	7.61074E-05	2.40673E-05	Arachis_hypogaea	0	0	Paludibacteraceae_uncultured__metagenome	0.000099194	3.13679E-05
Oscillibacter_sp.	0.00016120	0.00005098	Actinotignum_sp.	0	0	Spongospora_subterranea	0.000058924	1.86334E-05	Microscillaceae_uncultured__sp.	7.61074E-05	2.40673E-05	Arboricoccus_pini	0	0	Polyangiaceae_sp.	0.000099194	3.13679E-05
Sphingobacteriales_KD3-93_uncultured_bacterium	0.00016120	0.00000000	Adhaeribacter__metagenome	0	0	Johnsonella_uncultured_Lachnospiraceae	5.27037E-05	1.66664E-05	Saccharimonadalesuncultured_gamma	7.61074E-05	2.40673E-05	Arcicella_rigui	0	0	ASV_16940	9.31287E-05	2.94499E-05
Sutterellaceae_AAP99_sp.	0.00016120	0.00000000	Adhaeribacter__uncultured_soil	0	0	Rhodothermaceae_uncultured_bacterium	5.27037E-05	1.66664E-05	Sphingobacteriales_NS11-12_marine_group_sp.	7.61074E-05	2.40673E-05	Arcicella_sp.	0	0	ASV_16946	9.31287E-05	2.94499E-05
Flexibacter_sp.	0.00016059	0.00005078	Adhaeribacter_sp.	0	0	ASV_20126	4.71392E-05	1.49067E-05	ASV_6710	7.48372E-05	2.36656E-05	Arcicella_uncultured_bacterium	0	0	Candidatus_Kaiserbacteria_uncultured_Parcubacteria	9.31287E-05	2.94499E-05
Frigoribacterium_sp.	0.00016059	0.00005078	Advenella_sp.	0	0	Chloroplast_Cryptomonas_curvata	4.71392E-05	1.49067E-05	C1-B045_uncultured_gamma	7.48372E-05	2.36656E-05	Arcobacter_cryaerophilus	0	0	Micropepsis_pineolensis	9.31287E-05	2.94499E-05
MN_122.2a_uncultured_bacterium	0.00016059	0.00005078	Aenigmarchaeales_sp.	0	0	Paracoccus_sp.	4.71392E-05	1.49067E-05	Erysipelotrichaceae_ZOR0006_uncultured_bacterium	7.48372E-05	2.36656E-05	Arcobacter_sp.	0	0	Singulisphaera_uncultured_bacterium	9.31287E-05	2.94499E-05
Ohtaekwangia_metagenome	0.00016059	0.00005078	Aenigmarcheota_sp.	0	0	ASV_4563	0.000039391	1.24565E-05	Pedosphaeraceaeuncultured_Verrucomicrobia	7.48372E-05	2.36656E-05	Arcobacteraceae_sp.	0	0	ASV_2080	8.65411E-05	2.73667E-05
QuadrisphaeraQuadrisphaera_sp.	0.00016059	0.00000000	Aequorivita_sp.	0	0	Bacteroidales_oral	0.000039391	1.24565E-05	Plantibacter_sp.	7.48372E-05	2.36656E-05	Arcobacteraceae_uncultured__sp.	0	0	Candidatus_Harrisonbacteria	8.65411E-05	2.73667E-05
Acidibacter__metagenome	0.00014734	0.00004659	Aerococcaceae_sp.	0	0	Leptospira_metagenome	0.000039391	1.24565E-05	Sphingobacteriia_bacterium	7.48372E-05	2.36656E-05	Arcticibacter_sp.	0	0	Paracaedibacteraceae_uncultured__bacterium	8.65411E-05	2.73667E-05
ASV_16866	0.00014734	0.00004659	Aerococcaceae_uncultured__bacterium	0	0	Rhodopirellula_sp.	0.000039391	1.24565E-05	ASV_3630	6.56656E-05	2.07653E-05	Arcticibacter_uncultured_bacterium	0	0	Erysipelatoclostridiaceae_uncultured__bacterium	8.39123E-05	2.65354E-05
ASV_16876	0.00014734	0.00004659	Aerococcus_sp.	0	0	Actinomyces_dentalis	3.53544E-05	1.118E-05	Slackia_sp.	6.56656E-05	2.07653E-05	Arenicellaceae_uncultured_prokaryote	0	0	Rhodopseudomonas_sp.	8.39123E-05	2.65354E-05
ASV_2103	0.00014734	0.00004659	Aeromicrobium_sp.	0	0	Candidatus_Falkowbacteria	3.53544E-05	1.118E-05	ASV_6176	6.23791E-05	1.9726E-05	Arenimonas_oryziterrae	0	0	Sporomusaceae_uncultured__bacterium	8.07412E-05	2.55326E-05
Clostridia_vadinBB60_groupunidentified	0.00014734	0.00004659	Aeromonas__metagenome	0	0	Dyadobacter_uncultured_bacterium	3.53544E-05	1.118E-05	ASV_6240	6.23791E-05	1.9726E-05	Arenimonas_sp.	0	0	uncultured_Asticcacaulis	8.07412E-05	2.55326E-05
Methylophilaceae_sp.	0.00014734	0.00004659	Aeromonas_sp.	0	0	Nitrosomonadaceae_Ellin6067_sp.	3.53544E-05	1.118E-05	Candidatus_Methylopumilus_uncultured_beta	6.23791E-05	1.9726E-05	Aridibacter_sp.	0	0	ASV_18156	7.76072E-05	2.45416E-05
Nordella_uncultured_bacterium	0.00014734	0.00004659	Aeromonas_uncultured_gamma	0	0	Saccharimonadales_LWQ8_uncultured_soil	3.53544E-05	1.118E-05	Candidatus_Vogelbacteria_sp.	5.07382E-05	1.60448E-05	Armatimonadales_metagenome	0	0	Erysipelatoclostridiaceae_UCG-004_sp.	7.76072E-05	2.45416E-05
Picea_glauca	0.00014734	0.00004659	Aerosphaera__uncultured_bacterium	0	0	Bacteroidia_uncultured__bacterium	3.51358E-05	1.11109E-05	Pseudanabaena_PCC-6802_uncultured_cyanobacterium	5.07382E-05	1.60448E-05	Armatimonadales_uncultured_bacterium	0	0	Sphingobacteriales_NS11-12_marine_group_uncultured_bacterium	7.76072E-05	2.45416E-05
Rhodobacteraceae_Yangia_sp.	0.00014734	0.00000000	Aerosphaera_sp.	0	0	Candidatus_Uhrbacteria_metagenome	3.51358E-05	1.11109E-05	Aliterella_sp.	4.98915E-05	1.57771E-05	Armatimonadales_uncultured_Chloroflexi	0	0	Yersiniaceae_Yersinia_sp.	7.76072E-05	2.45416E-05
Gemmataceae_uncultured__sp.	0.00013843	0.00004377	Aestuariicella__uncultured_bacterium	0	0	Gemmata_uncultured_Planctomycetales	3.51358E-05	1.11109E-05	ASV_21560	4.98915E-05	1.57771E-05	Armatimonadales_uncultured_eubacterium	0	0	Acidimicrobiia_uncultured__metagenome	7.61528E-05	2.40816E-05
Gemmataceae_uncultured_prokaryote	0.00013843	0.00004377	Afipia_genosp.	0	0	Nakamurella_sp.	3.51358E-05	1.11109E-05	Candidatus_Campbellbacteria_uncultured_bacterium	4.98915E-05	1.57771E-05	Armatimonadalesuncultured_Armatimonadetes	0	0	ASV_305	7.61528E-05	2.40816E-05
Saccharimonadales_uncultured_bacterium	0.00013843	0.00000000	Agathobacter_sp.	0	0	ASV_21907	2.35696E-05	7.45336E-06	Chloroflexi_Gitt-GS-136_sp.	4.98915E-05	1.57771E-05	Armatimonadia_sp.	0	0	ASV_346	7.61528E-05	2.40816E-05
Solirubrobacterales_67-14_Solirubrobacterales_bacterium	0.00013843	0.00000000	Aggregatibacter_actinomycetemcomitans	0	0	Pedobacter_sp.	2.35696E-05	7.45336E-06	Cytophaga_sp.	4.98915E-05	1.57771E-05	Armatimonadota_uncultured__bacterium	0	0	Bulleidia_extructa	7.61528E-05	2.40816E-05
Caldalkalibacillus_uncultured_compost	0.00013765	0.00004353	Aggregatibacter_sp.	0	0	Rubinisphaeraceae_SH-PL14_uncultured_planctomycete	2.35696E-05	7.45336E-06	Ruminococcus_torques_group_uncultured_Clostridiales	4.98915E-05	1.57771E-05	Armatimonadota_uncultured__metagenome	0	0	Armatimonadales_uncultured_bacterium	7.43955E-05	2.35259E-05
Candidatus_Woykebacteria_uncultured_Microgenomates	0.00013765	0.00004353	Agitococcus_lubricus_group__uncultured_bacterium	0	0	Faecalibacterium_metagenome	1.96955E-05	6.22826E-06	Sandaracinaceae_uncultured__sp.	4.98915E-05	1.57771E-05	Armatimonadota_uncultured__sp.	0	0	ASV_1837	7.43955E-05	2.35259E-05
Phreatobacter_uncultured_bacterium	0.00013765	0.00004353	Agitococcus_lubricus_group_sp.	0	0	009E01-B-SD-P15__uncultured_bacterium	0	0	Candidatus_Jorgensenbacteria_sp.	4.37771E-05	1.38435E-05	Armatimonadota_uncultured__uncultured_soil	0	0	Candidatus_Kerfeldbacteria_sp.	7.43955E-05	2.35259E-05
Rhizobiaceae_uncultured__metagenome	0.00013433	0.00000000	Agrococcus_sp.	0	0	1013-28-CG33__uncultured_soil	0	0	Methanobrevibacter_archaeon_enrichment	4.37771E-05	1.38435E-05	Armatimonas_sp.	0	0	Candidatus_Protochlamydia	7.43955E-05	2.35259E-05
ASV_256	0.00012433	0.00003932	Agromyces_sp.	0	0	Abditibacterium_sp.	0	0	Propionispira_paucivorans	4.37771E-05	1.38435E-05	Armatimonas_uncultured_bacterium	0	0	Prevotellaceae_UCG-004_uncultured_bacterium	7.43955E-05	2.35259E-05
Candidatus_Yanofskybacteria_uncultured_bacterium	0.00012433	0.00003932	Ahniella_affigens	0	0	Absconditabacteriales_(SR1)__metagenome	0	0	009E01-B-SD-P15__uncultured_bacterium	0	0	Arsenicicoccus_bolidensis	0	0	Skermanella_sp.	7.43955E-05	2.35259E-05
ASV_18078	0.00012279	0.00003883	Ahniella_metagenome	0	0	Absconditabacteriales_(SR1)__uncultured_bacterium	0	0	1013-28-CG33__uncultured_soil	0	0	Arthrobacter_sp.	0	0	ASV_19928	6.92329E-05	2.18934E-05
ASV_2889	0.00012279	0.00003883	Ahniella_uncultured_bacterium	0	0	Absconditabacteriales_(SR1)_candidate_division	0	0	Abditibacterium_sp.	0	0	Aspergillus_nidulans	0	0	Candidatus_Falkowbacteria_uncultured_prokaryote	6.92329E-05	2.18934E-05

Bacteroidales_RF16_groupuncultured_Parabacteroides	0.00012279	0.00003883	Akkermansia_sp.	0	0	Absconditabacteriales_(SR1)_sp.	0	0	Absconditabacteriales_(SR1)__metagenome	0	0	Asticcacaulis_sp.	0	0	Bryobacter_sp.	6.71299E-05	2.12283E-05
Campylobacteria_uncultured__sp.	0.00012279	0.00003883	Akkermansia_uncultured_bacterium	0	0	Absconditabacteriales_(SR1)_SR1_bacterium	0	0	Absconditabacteriales_(SR1)__uncultured_bacterium	0	0	ASV_10003	0	0	Sericytochromatiaunidentified	6.71299E-05	2.12283E-05
Candidatus_Obscuribacter_sp.	0.00012279	0.00003883	Alcaligenaceae_sp.	0	0	Absconditabacteriales_(SR1)_uncultured_candidate	0	0	Absconditabacteriales_(SR1)_candidate_division	0	0	ASV_10004	0	0	ASV_19447	6.20858E-05	1.96333E-05
Chloroplast_Monoraphidium_neglectum	0.00012279	0.00003883	Alcaligenes_sp.	0	0	Absconditabacteriales_(SR1)_uncultured_Epsilonproteobacteria	0	0	Absconditabacteriales_(SR1)_sp.	0	0	ASV_10035	0	0	DEV007_uncultured_verrucomicrobium	6.20858E-05	1.96333E-05
Coriobacteriia_OPB41_uncultured_soil	0.00012279	0.00003883	Algoriphagus_sp.	0	0	Absconditabacteriales_(SR1)_uncultured_organism	0	0	Absconditabacteriales_(SR1)_SR1_bacterium	0	0	ASV_10043	0	0	Faecalibacterium_metagenome	6.20858E-05	1.96333E-05
Desulfuromonadia_PB19_sp.	0.00012279	0.00003883	Algoriphagus_uncultured_Bacteroidetes	0	0	Absconditabacteriales_(SR1)_uncultured_prokaryote	0	0	Absconditabacteriales_(SR1)_uncultured_candidate	0	0	ASV_10098	0	0	Pajaroellobacteruncultured_delta	6.20858E-05	1.96333E-05
Holophagaceae_sp.	0.00012279	0.00003883	Algoriphagus_uncultured_Hongiella	0	0	ABY1_sp.	0	0	Absconditabacteriales_(SR1)_uncultured_Epsilonproteobacteria	0	0	ASV_10170	0	0	Simkaniaceae_Ga0074140_sp.	6.05559E-05	1.91495E-05
Lactobacillus_senioris	0.00012279	0.00003883	Aliidiomarina_uncultured_bacterium	0	0	Acanthopleuribacter__uncultured_bacterium	0	0	Absconditabacteriales_(SR1)_uncultured_organism	0	0	ASV_10179	0	0	Spongiibacteraceae_BD1-7_clade_metagenome	6.05559E-05	1.91495E-05
Limnochordia_MBA03_uncultured_bacterium	0.00012279	0.00003883	Aliidongia_dinghuensis	0	0	Acetitomaculum__uncultured_bacterium	0	0	Absconditabacteriales_(SR1)_uncultured_prokaryote	0	0	ASV_1021	0	0	Sporosarcina_sp.	6.05559E-05	1.91495E-05
ASV_18142	0.00011471	0.00003627	Aliivibrio_sp.	0	0	Acetitomaculum__uncultured_rumen	0	0	ABY1_sp.	0	0	ASV_10253	0	0	Phaselicystis_uncultured_bacterium	5.19247E-05	1.642E-05
Babeliales_metagenome	0.00011471	0.00003627	Alishewanella_sp.	0	0	Acetitomaculum_sp.	0	0	Acanthopleuribacter__uncultured_bacterium	0	0	ASV_10292	0	0	Aenigmarchaeales_sp.	5.03474E-05	1.59212E-05
Patescibacteria_CPR2_uncultured_eubacterium	0.00011471	0.00003627	Alishewanella_uncultured_bacterium	0	0	Acetivibrio_uncultured_bacterium	0	0	Acetitomaculum__uncultured_bacterium	0	0	ASV_10316	0	0	ASV_21236	5.03474E-05	1.59212E-05
Patescibacteria_CPR2_uncultured_Firmicutes	0.00011471	0.00003627	Alistipes_gut__metagenome	0	0	Acetoanaerobium_uncultured_bacterium	0	0	Acetitomaculum__uncultured_rumen	0	0	ASV_10346	0	0	Bacilli_RF39_uncultured_Erysipelotrichaceae	5.03474E-05	1.59212E-05
Propioniciclava_sp.	0.00011471	0.00000000	Alistipes_sp.	0	0	Acetobacter_sp.	0	0	Acetitomaculum_sp.	0	0	ASV_10385	0	0	Bacteriovoracaceae_sp.	5.03474E-05	1.59212E-05
Saccharimonadales_TM7_sp.	0.00011471	0.00000000	Alistipes_uncultured_Alistipes	0	0	Acetobacteraceae_sp.	0	0	Acetoanaerobium_uncultured_bacterium	0	0	ASV_10460	0	0	Brachymonas_sp.	5.03474E-05	1.59212E-05
Tahibacter_sp.	0.00011471	0.00008496	Alistipes_uncultured_bacterium	0	0	Acetobacteraceae_uncultured__sp.	0	0	Acetobacteraceae_sp.	0	0	ASV_10537	0	0	Chitinophagales_37-13_sp.	5.03474E-05	1.59212E-05
ASV_19488	0.00010747	0.00003398	Alistipes_uncultured_Bacteroidetes	0	0	Acetobacterium_sp.	0	0	Acetobacteraceae_uncultured__bacterium	0	0	ASV_10583	0	0	Desulfobacterota_uncultured_Desulfuromonadales	5.03474E-05	1.59212E-05
ASV_19490	0.00010747	0.00003398	Aliterella_cyanobacterium_OU_20	0	0	Acetothermiia__uncultured_bacterium	0	0	Acetobacteraceae_uncultured__metagenome	0	0	ASV_10605	0	0	Methylacidiphilaceae_uncultured__metagenome	5.03474E-05	1.59212E-05
Antricoccus_suffuscus	0.00009823	0.00003106	Aliterella_sp.	0	0	Acholeplasma__uncultured_bacterium	0	0	Acetobacteraceae_uncultured__sp.	0	0	ASV_10606	0	0	Obscuribacteraceae_metagenome	5.03474E-05	1.59212E-05
ASV_19368	0.00009823	0.00003106	Aliterella_uncultured_bacterium	0	0	Acholeplasma_axanthum	0	0	Acetobacterium_sp.	0	0	ASV_10613	0	0	Pelosinus_uncultured_bacterium	5.03474E-05	1.59212E-05
Babelialescandidate_division	0.00009823	0.00003106	Alkalibacter_uncultured_bacterium	0	0	Acholeplasma_brassicae	0	0	Acetothermiia__uncultured_bacterium	0	0	ASV_10814	0	0	SB-5_uncultured_soil	5.03474E-05	1.59212E-05
Cryptobacterium_sp.	0.00009823	0.00003106	Alkalibacterium_uncultured_bacterium	0	0	Acholeplasma_laidlawii	0	0	Acholeplasma_axanthum	0	0	ASV_10821	0	0	Sphingobacteriales_KD3-93_sp.	5.03474E-05	1.59212E-05
Gemmata__uncultured_planctomycete	0.00009823	0.00003106	Alkanibacter_metagenome	0	0	Acholeplasma_morum	0	0	Acholeplasma_brassicae	0	0	ASV_10869	0	0	ASV_21606	0.000049597	1.56839E-05
Geodermatophilaceae_sp.	0.00009823	0.00003106	Alkanindiges_sp.	0	0	Acholeplasma_sp.	0	0	Acholeplasma_laidlawii	0	0	ASV_10995	0	0	Bacteroides_plebeius	0.000049597	1.56839E-05
Gracilibacteria_JGI_0000069-P22_sp.	0.00009823	0.00003106	Alkanindiges_uncultured_bacterium	0	0	Acholeplasmataceae_EMP-G18_uncultured_Mollicutes	0	0	Acholeplasma_morum	0	0	ASV_11002	0	0	Clavibacter_sp.	0.000049597	1.56839E-05
Sphingobacteriales_NS11-12_marine_groupuncultured_Flexibacteraceae	0.00009823	0.00000000	Alkanindiges_uncultured_gamma	0	0	Acholeplasmauncultured_Acholeplasmataceae	0	0	Acholeplasma_sp.	0	0	ASV_11030	0	0	Coxiella_uncultured_bacterium	0.000049597	1.56839E-05
Tissierella_uncultured_bacterium	0.00009823	0.00000000	Allobranchiibius_sp.	0	0	Achromatium_sp.	0	0	Acholeplasmataceae_EMP-G18_uncultured_Mollicutes	0	0	ASV_11102	0	0	Granulicella_sp.	0.000049597	1.56839E-05
ASV_2061	0.00009444	0.00002986	Alloiococcus_uncultured_bacterium	0	0	Acidaminobacter__uncultured_bacterium	0	0	Acholeplasmauncultured_Acholeplasmataceae	0	0	ASV_11103	0	0	Phycisphaeraceae_SM1A02_uncultured_bacterium	4.65643E-05	1.47249E-05
Candidatus_Aquirestis_sp.	0.00009444	0.00002986	Alloprevotella_rava	0	0	Acidaminococcaceae_uncultured__bacterium	0	0	Acidaminobacter__uncultured_bacterium	0	0	ASV_11163	0	0	Silvanigrella_Geobacter_sp.	4.65643E-05	1.47249E-05
Candidatus_Magasanikbacteria__uncultured_Parcubacteria	0.00009177	0.00002902	Alloprevotella_sp.	0	0	Acidibacter__metagenome	0	0	Acidaminococcaceae_uncultured__bacterium	0	0	ASV_11164	0	0	Corynebacterium_mycetoides	4.03706E-05	1.27663E-05
Candidatus_Peribacteria_sp.	0.0000	0.0000	Alloprevotella_tannerae	0	0	Acidibacter__uncultured_bacterium	0	0	Acidibacter__metagenome	0	0	ASV_11306	0	0	Flavobacterium_unidentified	4.03706	1.27663

	9177	2902														E-05	E-05
Chitinophaga_niastensis	0.00009177	0.00002902	Alloprevotella_uncultured_Bacteroidales	0	0	Acidibacter_sp.	0	0	Acidibacter__uncultured_bacterium	0	0	ASV_11362	0	0	Acidaminobacter__uncultured_bacterium	3.46164E-05	1.09467E-05
Conexibacter_uncultured_bacterium	0.00009177	0.00002902	Allorhizobium-Neorhizobium-Pararhizobium-Rhizobium_sp.	0	0	Acidimicrobiia_IMCC26256_metagenome	0	0	Acidibacter_sp.	0	0	ASV_11393	0	0	ASV_2510	3.46164E-05	1.09467E-05
Eubacterium_coprostanoligenes_group_uncultured_Clostridia	0.00009177	0.00002902	Alloscardovia_omnicolens	0	0	Acidimicrobiia_IMCC26256_sp.	0	0	Acidimicrobiia_IMCC26256_metagenome	0	0	ASV_11399	0	0	Chloroplast_sp.	3.46164E-05	1.09467E-05
MBNT15uncultured_Anaeromyxobacter	0.00009177	0.00002902	allotenuales_AKIW781_uncultured_bacterium	0	0	Acidimicrobiia_IMCC26256_uncultured_bacterium	0	0	Acidimicrobiia_IMCC26256_sp.	0	0	ASV_11414	0	0	Bacteriovorax_sp.	3.35649E-05	1.06142E-05
Pirellulaceae_uncultured__bacterium	0.00009177	0.00002902	alpha_proteobacterium	0	0	Acidimicrobiia_IMCC26256_uncultured_soil	0	0	Acidimicrobiia_IMCC26256_uncultured_bacterium	0	0	ASV_11446	0	0	LCP-89_uncultured_bacterium	3.35649E-05	1.06142E-05
ASV_7664	0.00008857	0.00002801	Alphaproteobacteria_bacterium	0	0	Acidimicrobiia_IMCC26256uncultured_Aciditerrimonas	0	0	Acidimicrobiia_IMCC26256_uncultured_soil	0	0	ASV_11492	0	0	ASV_2889	3.10429E-05	9.81663E-06
Chitinophagales_uncultured_delta	0.00008060	0.00002549	Alphaproteobacteria_sp.	0	0	Acidimicrobiia_IMCC26256uncultured_actinobacterium	0	0	Acidimicrobiia_IMCC26256uncultured_Aciditerrimonas	0	0	ASV_11530	0	0	Candidatus_Vogelbacteria	3.10429E-05	9.81663E-06
Sphingobacteriales_NS11-12_marine_groupuncultured_Bacteroidetes	0.00008060	0.00000000	Alphaproteobacteria_uncultured__bacterium	0	0	Acidimicrobiia_IMCC26256uncultured_Actinomycetales	0	0	Acidimicrobiia_IMCC26256uncultured_actinobacterium	0	0	ASV_11581	0	0	Lentimicrobium_metagenome	3.10429E-05	9.81663E-06
Sphingopyxis_uncultured_bacterium	0.00008060	0.00000000	Alphaproteobacteria_uncultured__metagenome	0	0	Acidimicrobiia_sp.	0	0	Acidimicrobiia_sp.	0	0	ASV_1161	0	0	ASV_2160	2.47985E-05	7.84197E-06
ASV_2386	0.00007367	0.00002330	Alphaproteobacteria_uncultured__sp.	0	0	Acidimicrobiia_uncultured__bacterium	0	0	Acidimicrobiia_uncultured__bacterium	0	0	ASV_11616	0	0	1013-28-CG33__uncultured_soil	0	0
Bryobacter_metagenome	0.00007367	0.00002330	Alphaproteobacteria_uncultured_Alphaproteobacteria	0	0	Acidimicrobiia_uncultured__metagenome	0	0	Acidimicrobiia_uncultured__metagenome	0	0	ASV_11696	0	0	Abditibacterium_sp.	0	0
Caedibacteraceae_uncultured__bacterium	0.00007367	0.00002330	Alphaproteobacteria_uncultured_bacterium_enrichment	0	0	Acidimicrobiia_uncultured__sp.	0	0	Acidimicrobiia_uncultured__sp.	0	0	ASV_11732	0	0	Absconditabacteriales_(SR1)__metagenome	0	0
Desulfobacteraceae_uncultured_delta	0.00007367	0.00002330	Alphaproteobacteria_uncultured_eubacterium	0	0	Acidimicrobiia_uncultured_Acidimicrobiia	0	0	Acidimicrobiia_uncultured_Acidimicrobiia	0	0	ASV_11789	0	0	Absconditabacteriales_(SR1)__uncultured_bacterium	0	0
Lentimicrobium_sp.	0.00007367	0.00002330	Alphaproteobacteria_uncultured_gut__metagenome	0	0	Acidimicrobiia_uncultured_actinobacterium	0	0	Acidimicrobiia_uncultured_actinobacterium	0	0	ASV_11832	0	0	Absconditabacteriales_(SR1)_candidate_division	0	0
Prolixibacteraceae_uncultured_prokaryote	0.00007367	0.00000000	Alphaproteobacteria_uncultured_Lacibacterium_aquatile	0	0	Acidimicrobiia_uncultured_bacterium_YC-ZSS-LKJ199	0	0	Acidimicrobiia_uncultured_Actinomycetales	0	0	ASV_12032	0	0	Absconditabacteriales_(SR1)_sp.	0	0
Silene_dioica	0.00007367	0.00000000	Alphaproteobacteria_uncultured_Novosphingobium	0	0	Acidiphilium__uncultured_bacterium	0	0	Acidimicrobiia_uncultured_bacterium_YC-ZSS-LKJ199	0	0	ASV_1208	0	0	Absconditabacteriales_(SR1)_SR1_bacterium	0	0
Verrucomicrobiae_uncultured__metagenome	0.00007367	0.00000000	Alphaproteobacteria_uncultured_Rhodospirillaceae_bacterium	0	0	Acidiphilium_sp.	0	0	Acidiphilium__uncultured_bacterium	0	0	ASV_12134	0	0	Absconditabacteriales_(SR1)_uncultured_candidate	0	0
Aerococcus_sp.	0.00006882	0.00002176	Altererythrobacter_alpha_proteobacterium	0	0	Acidisoma_sp.	0	0	Acidiphilium_sp.	0	0	ASV_12280	0	0	Absconditabacteriales_(SR1)_uncultured_Epsilonproteobacteria	0	0
ASV_20627	0.00006882	0.00002176	Altererythrobacter_sp.	0	0	Acidithiobacillaceae_KCM-B-112_sp.	0	0	Acidisoma_sp.	0	0	ASV_12284	0	0	Absconditabacteriales_(SR1)_uncultured_organism	0	0
ASV_3526	0.00006882	0.00002176	Alysiella_sp.	0	0	Acidobacteria_bacterium	0	0	Acidithiobacillaceae_KCM-B-112_sp.	0	0	ASV_12313	0	0	Absconditabacteriales_(SR1)_uncultured_prokaryote	0	0
Campylobacter_ureolyticus	0.00006882	0.00002176	Alysiella_uncultured_bacterium	0	0	Acidobacteriaceae_(Subgroup_1)_sp.	0	0	Acidithiobacillus_ferrooxidans	0	0	ASV_12382	0	0	ABY1_sp.	0	0
Gallionellaceae_sp.	0.00006882	0.00002176	Amaricoccus_sp.	0	0	Acidobacteriae_Elev-16S-1166_uncultured_bacterium	0	0	Acidobacteriaceae_(Subgroup_1)_sp.	0	0	ASV_12391	0	0	Acanthopleuribacter__uncultured_bacterium	0	0
Hungateiclostridiaceae_HN-HF0106_sp.	0.00006882	0.00002176	Amaricoccus_tamworthensis	0	0	Acidobacteriae_sp.	0	0	Acidobacteriae_Elev-16S-1166_sp.	0	0	ASV_12397	0	0	Acetitomaculum__uncultured_bacterium	0	0
Mariprofundus_sp.	0.00006882	0.00002176	Amb-16S-1323_uncultured_bacterium	0	0	Acidocella__uncultured_bacterium	0	0	Acidobacteriae_Elev-16S-1166_uncultured_bacterium	0	0	ASV_12420	0	0	Acetitomaculum__uncultured_rumen	0	0
Oscillospiraceae_NK4A214_group_uncultured_rumen	0.00006882	0.00002176	Aminobacter_sp.	0	0	Acidothermus__uncultured_bacterium	0	0	Acidocella__uncultured_bacterium	0	0	ASV_12452	0	0	Acetitomaculum_sp.	0	0
Planctomycetota_OM190_uncultured_soil	0.00006882	0.00002176	Ammoniibacillus_agariperforans	0	0	Acidothermus_sp.	0	0	Acidothermus__uncultured_bacterium	0	0	ASV_12478	0	0	Acetivibrio_uncultured_bacterium	0	0
Prosthecobacter_sp.	0.00006882	0.00000000	Ammoniphilus_oxalaticus	0	0	Acidovorax_sp.	0	0	Acidothermus_sp.	0	0	ASV_12525	0	0	Acetoanaerobium_uncultured_bacterium	0	0
Rikenellaceae_RC9_gut_group_sp.	0.00006882	0.00005898	Amnibacterium_sp.	0	0	Acinetobacter__metagenome	0	0	Acidovorax_sp.	0	0	ASV_12573	0	0	Acetobacter_sp.	0	0
Roseococcus_uncultured_bacterium	0.00006882	0.00000000	Amnipila_sp.	0	0	Acinetobacter_baumannii	0	0	Acinetobacter__metagenome	0	0	ASV_12623	0	0	Acetobacteraceae_sp.	0	0
ASV_6240	0.00006217	0.00001966	Amycolatopsis_sp.	0	0	Acinetobacter_kyongensis	0	0	Acinetobacter_baumannii	0	0	ASV_12634	0	0	Acetobacteraceae_uncultured__bacterium	0	0

Armatimonas_uncultured_bacterium	0.00005373	0.00001699	Anaerobacterium_uncultured_bacterium	0	0	Acinetobacter_sp.	0	0	Acinetobacter_kyonggiensis	0	0	ASV_12731	0	0	Acetobacteraceae_uncultured__metagenome	0	0
Bacteroidia_sp.	0.00005373	0.00001699	Anaerobiospirillum_succiniciproducens	0	0	Acinetobacter_towneri	0	0	Acinetobacter_sp.	0	0	ASV_12760	0	0	Acetobacteraceae_uncultured__sp.	0	0
TaibaiellaTaibaiella_sp.	0.00005373	0.00000000	Anaerococcus_hydrogenalis	0	0	Acremonium_fuci	0	0	Acinetobacter_towneri	0	0	ASV_12772	0	0	Acetobacterium_sp.	0	0
Woesearchaeales_SCGC_AAA011-D5_uncultured_archaeon	0.00005373	0.00000000	Anaerococcus_octavius	0	0	Actinobacillus_sp.	0	0	Acremonium_fuci	0	0	ASV_12862	0	0	Acetothermiia__uncultured_bacterium	0	0
Pirellulaceae_uncultured__metagenome	0.00004911	0.00001553	Anaerococcus_prevotii	0	0	Actinobacteria_0319-7L14_uncultured_actinobacterium	0	0	Actinobacillus_sp.	0	0	ASV_12880	0	0	Acholeplasma__uncultured_bacterium	0	0
Acholeplasma_sp.	0.00004588	0.00001451	Anaerococcus_sp.	0	0	Actinobacteria_PeM15_bacterium_rJ7	0	0	Actinobacteria_0319-7L14_uncultured_actinobacterium	0	0	ASV_12965	0	0	Acholeplasma_brassicae	0	0
ASV_21578	0.00004588	0.00001451	Anaerococcus_uncultured_bacterium	0	0	Actinobacteria_PeM15_metagenome	0	0	Actinobacteria_PeM15_bacterium_rJ7	0	0	ASV_12970	0	0	Acholeplasma_laidlawii	0	0
Candidatus_Magasanikbacteria_Parcubacteria_group	0.00004588	0.00001451	Anaerococcusuncultured_organism	0	0	Actinobacteria_PeM15_sp.	0	0	Actinobacteria_PeM15_metagenome	0	0	ASV_13011	0	0	Acholeplasma_morum	0	0
Gracilibacteria_JGI_0000069-P22_uncultured_bacterium	0.00004588	0.00001451	Anaerocolumna_uncultured_bacterium	0	0	Actinobacteria_PeM15_uncultured_soil	0	0	Actinobacteria_PeM15_sp.	0	0	ASV_13022	0	0	Acholeplasma_sp.	0	0
Neisseriaceae_uncultured_beta	0.00004588	0.00001451	Anaerofilumuncultured_Anaerofilum	0	0	Actinobacteria_PeM15uncultured_actinobacterium	0	0	Actinobacteria_PeM15_uncultured_soil	0	0	ASV_13091	0	0	Acholeplasmataceae_EMP-G18_uncultured_Mollicutes	0	0
Saccharimonadales_wastewater__metagenome	0.00004588	0.00000000	Anaerofustis_uncultured_bacterium	0	0	Actinobacteria_sp.	0	0	Actinobacteria_PeM15uncultured_actinobacterium	0	0	ASV_13184	0	0	Acholeplasmauncultured_Acholeplasmataceae	0	0
Simkaniaceae_uncultured__sp.	0.00004588	0.00000000	Anaeroglobus_geminatus	0	0	Actinobacteria_uncultured__bacterium	0	0	Actinobacteria_sp.	0	0	ASV_13191	0	0	Achromatium_sp.	0	0

Restorative																	
Assistant			Environment			Operator			Patient			Saliva			Waterline		
Taxon ID	Mean relative abundance	Std dev	Taxon ID	Mean relative abundance	Std dev	Taxon ID	Mean relative abundance	Std dev	Taxon ID	Mean relative abundance	Std dev	Taxon ID	Mean relative abundance	Std dev	Taxon ID	Mean relative abundance	Std dev
Uncultured_Vulcanibacterium	0.10941917	0.03535827	Uncultured_Vulcanibacterium	0.10390528	0.02611684	Uncultured_Vulcanibacterium	0.05259294	0.01374113	Streptococcus_mutans	0.1168548	0.02614685	Streptococcus_mutans	0.00676376	0.00340499	ASV_211	0.04176426	0.01102878
Uncultured_Vulcanibacterium	0.10124024	0.03319062	Uncultured_Vulcanibacterium	0.09899517	0.0203019	Uncultured_Vulcanibacterium	0.04861326	0.01163287	Uncultured_Vulcanibacterium	0.06898096	0.00435307	Prevotella_nigrescens	0.00309012	0.00161126	ASV_413	0.02660377	0.01535969
Schlegelella_uncultured_bacterium	0.023426	0.01299206	Schlegelella_uncultured_bacterium	0.01255624	0.00638408	Streptococcus_mutans	0.04106286	0.02263534	Uncultured_Vulcanibacterium	0.06673392	0.00557853	Leptotrichia_wadei	0.00126016	0.00052516	ASV_164	0.01571232	0.00858011
Actinomyces_israelii	0.00894752	0.00516585	Streptococcus_mutans	0.00624378	0.00346947	Lactobacillus_amylovorus	0.0277399	0.01601564	Lactobacillus_amylovorus	0.00827626	0.0047783	Atopobium_uncultured_bacterium	0.00098728	0.00057001	Amb-16S-1323_uncultured_bacterium	0.01441913	0.00832489
Aquisphaera_sp.	0.00858034	0.00495386	ASV_1901	0.0042605	0.0024598	Prevotella_nigrescens	0.00813242	0.00237072	Rhodococcus_erythropolis	0.00812199	0.00457615	Solobacterium_moorei	0.00056396	0.00022674	ASV_144	0.01246186	0.00675321
Adhaeribacter__uncultured_soil	0.00639626	0.00369288	ASV_8402	0.00273889	0.0015813	Solobacterium_moorei	0.00573824	0.00277949	Cryptobacterium_sp.	0.00443755	0.00138108	Streptococcus_anginosus	0.00047663	0.00014711	Uncultured_Vulcanibacterium	0.0091771	0.00463423
Aurantisolimonas_haloimpatiens	0.00514821	0.00297232	ASV_8010	0.00249017	0.0014377	Actinomyces_israelii	0.00494384	0.00272988	Denitratisoma_sp.	0.00428082	0.00247153	ASV_292	0.00037697	0.00021764	Denitratisoma_sp.	0.00853988	0.00461982
Rubinisphaeraceae_SH-PL14_metagenome	0.00514821	0.00297232	ASV_2160	0.00228241	0.00131775	Lactobacillus_salivarius	0.00441958	0.00255164	Prevotella_nigrescens	0.00276775	0.00081334	Rothia_aeria	0.00037697	0.00021764	Azospira_sp.	0.00807684	0.00466316
Solobacterium_moorei	0.00446983	0.00194464	ASV_4487	0.00228241	0.00131775	Rhodococcus_erythropolis	0.00390012	0.0022131	Actinomyces_israelii	0.0027467	0.00092389	Pseudoramibacter_sp.	0.00035698	0.0002061	Uncultured_Vulcanibacterium	0.00759534	0.00361373
Prevotella_nigrescens	0.00344569	0.00198937	ASV_9735	0.00228241	0.00131775	ASV_2108	0.00283828	0.00163868	Schlegelella_uncultured_bacterium	0.00264883	0.00125756	Cryptobacterium_sp.	0.00029489	0.00017026	C1-B045_uncultured_gamma	0.00680307	0.00342783
Defluviicoccusuncultured_Defluviicoccus	0.00327613	0.00189148	ASV_9740	0.00228241	0.00131775	Howardella_sp.	0.00200239	0.00115608	ASV_14967	0.00228311	0.00131815	Selenomonadaceae_uncultured_Veillonellaceae_bacterium	0.00029489	0.00017026	Edaphobaculum_uncultured_Chitinophagaceae	0.00481568	0.00142304
Opitutaceae_uncultured__sp.	0.00327613	0.00189148	Methylomonadaceae_uncultured__sp.	0.00228241	0.00131775	Jonquetella_anthropi	0.00136121	0.0007859	Lautropia_uncultured_bacterium	0.00170265	0.00098303	ASV_1071	0.00023281	0.00013441	Obscuribacteraceae_sp.	0.00431912	0.00183837
Gemmatimonadaceae_uncultured_proteobacterium	0.00312013	0.00180141	ASV_10292	0.00213025	0.0012299	Cryptobacterium_sp.	0.00123204	0.00036434	Actinomyces_dentalis	0.00159397	0.00092028	Eubacterium_infirmum	0.00017073	9.857E-05	Streptococcus_mutans	0.00366685	0.00211706
Chloroplast_Synura_petersenii	0.00296412	0.00171133	Microtrichaceae_uncultured__bacterium	0.00213025	0.0012299	Lawsonella_uncultured_bacterium	0.00120889	0.00047507	Lawsonella_uncultured_bacterium	0.00142694	0.00082384	Stomatobaculum_longum	0.00017073	9.857E-05	ASV_1777	0.00219629	0.00126803

Saprospiraceae_uncultured_Haliscomenobacter	0.00296412	0.00171133	Brevundimonas_diminuta	0.00207131	0.00059807	Flavobacterium_metagenome	0.00115848	0.00066885	Actinomycetaceae_F0332unidentified	0.00141284	0.0008157	Campylobacter_concisus	0.00015521	8.9609E-05	ASV_1862	0.00150876	0.00087108
Sulfurifustis_sp.	0.00280811	0.00162126	ASV_256	0.00197809	0.00114205	Brevundimonas_diminuta	0.00108139	0.00062434	Flavobacterium_terrigena	0.00141284	0.0008157	Howardella_sp.	0.00012417	7.1687E-05	Thioclava_uncultured_bacterium	0.00118827	0.00036329
Howardella_sp.	0.00239414	0.00138226	Prevotella_nigrescens	0.00188828	0.0010902	Schlegelella_uncultured_bacterium	0.00093473	0.00021947	ASV_3436	0.00130416	0.00075296	Anaerococcusuncultured_organism	0.00011615	6.706E-05	Schlegelella_uncultured_bacterium	0.00091671	0.00052926
Nitrososphaeraceae_uncultured_bacterium	0.00234009	0.00135105	ASV_2820	0.00183486	0.00105936	Arboricoccus_pini	0.00089782	0.00051836	ASV_3803	0.00126793	0.00073204	Lactobacillus_amylovorus	8.7113E-05	5.0295E-05	Prevotella_nigrescens	0.00085942	0.00049619
Amycolatopsis_sp.	0.00218409	0.00126098	Chryseobacterium_uncultured_Bacteroidetes	0.00183486	0.00105936	Intestinibacter_sp.	0.00089782	0.00051836	Prevotella_oralis	0.00115925	0.00066929	Bulleidia_extructa	5.8075E-05	3.353E-05	Singulisphaera_Isosphaera_sp.	0.00082122	0.00047413
Chitinivorax_sp.	0.00218409	0.00126098	Cryptobacterium_sp.	0.00173092	0.00099935	ASV_5309	0.00086886	0.00050164	Coprococcus_metagenome	0.00114155	0.00065908	Treponema_lecithinolyticum	5.8075E-05	3.353E-05	Fibrisoma_sp.	0.00076393	0.00044105
Rhodococcus_hoagii	0.00202808	0.00117091	Peptostreptococcales-Tissierellales_sp.	0.00173092	0.00099935	Methylomonas_sp.	0.00086886	0.00050164	Babeliales_sp.	0.00112303	0.00064838	Dialister_micraerophilus	4.6562E-05	2.6883E-05	Lactobacillus_rhamnosus	0.00074483	0.00043003
Sumerlaea_uncultured_bacterium	0.00202808	0.00117091	ASV_10869	0.0017038	0.00098369	Fimbriimonadaceae_sp.	0.00086473	0.00026364	Acetobacteraceae_uncultured_alpha_proteobacterium	0.00110908	0.00034185	ASV_2084	3.1041E-05	1.7922E-05	ASV_1071	0.00068754	0.00039695
Woesearchaeales_GW2011_GWC1_47_15_sp.	0.00202808	0.00117091	Fusarium_circinatum	0.00167377	0.00096635	Chitinibacter_metagenome	0.0008399	0.00048492	Holophagaceae_uncultured_Acidobacteria	0.00083321	0.00048106	Campylobacter_sp.	3.1041E-05	1.7922E-05	ASV_4516	0.00068754	0.00039695
ASV_1071	0.00202798	0.00117085	ASV_292	0.00152161	0.0008785	Leptotrichia_trevisanii	0.00079929	0.00046147	Listeria_sp.	0.00073056	0.00042179	Prevotella_saccharolytica	3.1041E-05	1.7922E-05	Lactobacillus_fermentum	0.00055385	0.00031976
Streptococcus_mutans	0.00193409	0.00111665	Lachnospiraceae_UCG-010uncultured_organism	0.00144168	0.00083235	Phycisphaerae_mle1-8_uncultured_bacterium	0.00078197	0.00045147	ASV_1208	0.00061585	0.00035556	ASV_9	1.5521E-05	8.9609E-06	Actinomyces_israelii	0.00053475	0.00030874
Spirochaetaceae_uncultured_Ruditapes_philippinarum	0.00187208	0.00108084	ASV_14013	0.00136945	0.00079065	SphingorhabdusSphingopyxis_sp.	0.00072405	0.00041803	Parasediminibacterium_uncultured_bacterium	0.00057963	0.00033465	009E01-B-SD-P15__uncultured_bacterium	0	0	ASV_800	0.00048214	0.00019805
ASV_1837	0.00186514	0.00056154	Novosphingobium_alpha_proteobacterium	0.00136945	0.00079065	Bacillus_thermoamylovorans	0.00069357	0.00040043	Leptotrichia_wadei	0.00050841	0.00026176	1013-28-CG33__uncultured_soil	0	0	ASV_6399	0.00047746	0.00027566
ASV_8384	0.00173947	0.00100428	Peptoniphilus_sp.	0.00136945	0.00079065	ASV_720	0.000662	0.00026681	Alkanibacter_metagenome	0.00047095	0.0002719	Abditibacterium_sp.	0	0	Rothia_aeria	0.00043065	0.00019293
ASV_720	0.00154619	0.00089269	Myxococcaceae_uncultured_bacterium	0.00131062	0.00075668	Sphingobacteriales_NS11-12_marine_groupBacteroidetes_bacterium	0.00065824	0.00038003	ASV_6292	0.00043472	0.00025099	Absconditabacteriales_(SR1)__metagenome	0	0	ASV_363	0.00032558	0.00018797
ASV_1571	0.00149282	0.00086188	Oikopleura_metagenome	0.00121729	0.0007028	ASV_7050	0.00063716	0.00036787	Beijerinckiaceae_uncultured_endolithic	0.00043472	0.00025099	Absconditabacteriales_(SR1)__uncultured_bacterium	0	0	Leptotrichia_wadei	0.00028647	0.0001654
Lautropia_metagenome	0.00140406	0.00081063	Collimonas_sp.	0.00118017	0.00068137	Chloroplast_Cucumis_sativus	0.00057924	0.00033442	SanguibacterSanguibacter_sp.	0.00043472	0.00025099	Absconditabacteriales_(SR1)_candidate_division	0	0	Prevotella_genomosp.	0.00026738	0.00015437
Nitrosomonadaceae_DSSD61_uncultured_bacterium	0.00140406	0.00081063	Methylopila_sp.	0.00118017	0.00068137	Varibaculum_sp.	0.00055028	0.0003177	Streptococcus_sobrinus	0.00043084	0.00024875	Absconditabacteriales_(SR1)_sp.	0	0	Obscuribacteraceae_metagenome	0.00026297	0.00015182
Thermomonas_sp.	0.00140406	0.00081063	ASV_366	0.00117955	0.00068102	Leptotrichia_wadei	0.0005371	0.00027226	Pseudomonas_pertucinogena	0.00039849	0.00023007	Absconditabacteriales_(SR1)_SR1_bacterium	0	0	Rhodococcus_erythropolis	0.00026017	0.00010973
Roseiflexaceae_uncultured__sp.	0.00125628	0.00072531	CCD24_sp.	0.00106513	0.00061495	Kocuria_marina	0.00052809	0.00025637	Psychrobacter_fozii	0.00039849	0.00023007	Absconditabacteriales_(SR1)_uncultured_candidate	0	0	Acetobacteraceae_uncultured_alpha_proteobacterium	0.00023967	8.6579E-05
Pirellula_uncultured_Planctomycetales	0.00124805	0.00072056	Gemmatimonas_phototrophica	0.00106513	0.00061495	Oligoflexales_uncultured__sp.	0.00051719	0.0002986	Oligoflexales_uncultured__soil	0.00036227	0.00020915	Absconditabacteriales_(SR1)_uncultured_Epsilonproteobacteria	0	0	Clostridia_vadinBB60_group_Clostridiales_bacterium	0.00022918	0.00013232
Rhodococcus_erythropolis	0.00120659	0.00065639	PirellulaPlanctomycetes_bacterium	0.00106513	0.00061495	Acholeplasma_morum	0.00049235	0.00028426	Rhynchosporium_secalis	0.00036227	0.00020915	Absconditabacteriales_(SR1)_uncultured_organism	0	0	Meiothermus_uncultured_bacterium	0.0002254	0.00013013
ASV_11530	0.00115964	0.00066952	Persicitalea_uncultured_bacterium	0.00094414	0.0005451	Alkanindiges_uncultured_gamma	0.00049235	0.00028426	ASV_14011	0.00032604	0.00018824	Absconditabacteriales_(SR1)_uncultured_prokaryote	0	0	ASV_1021	0.00019098	0.00011026
Arenicellaceae_uncultured_prokaryote	0.00109204	0.00063049	Lawsonella_uncultured_bacterium	0.00091296	0.0005271	ASV_1208	0.00049235	0.00028426	ASV_211	0.00032604	0.00018824	ABY1_sp.	0	0	Ureibacillus_sp.	0.00019098	0.00011026
ASV_709	0.00106301	0.00061373	Patescibacteria_uncultured__bacterium	0.00091296	0.0005271	Candidatus_Buchananbacteria_metagenome	0.00049235	0.00028426	ASV_3256	0.00032604	0.00018824	Acanthopleuribacter__uncultured_bacterium	0	0	Streptococcus_sobrinus	0.00017188	9.9237E-05
Peptoniphilus_duerdenii	0.00106301	0.00061373	Peptoniphilus_urinimassiliensis	0.00091296	0.0005271	Parasutterella_uncultured_organism	0.00049235	0.00028426	Gastranaerophilales_uncultured_cyanobacterium	0.00032604	0.00018824	Acetitomaculum__uncultured_bacterium	0	0	Leptotrichiaceae_uncultured_marine__metagenome	0.00016279	9.3986E-05
Saccharimonadales_TM7auncultured_compost	0.00096637	0.00055793	ASV_720	0.00078637	0.00045401	ASV_709	0.00044497	0.00016694	Brevundimonas_diminuta	0.00029972	0.00017304	Acetitomaculum__uncultured_rumen	0	0	Prevotella_aurantiaca	0.00016269	9.3928E-05
Chitinophagales_37-13__metagenome	0.00093604	0.00054042	Fibrobacteraceae_uncultured__bacterium	0.0007608	0.00043925	Cloacibacterium_sp.	0.00043628	0.00025188	Flavobacterium_aquatile	0.00028981	0.00016732	Acetitomaculum_sp.	0	0	ASV_366	0.00015027	8.6757E-05
Neisseriaceae_uncultured__bacterium	0.00093604	0.00054042	Rhodospirillales_uncultured_proteobacterium	0.0007608	0.00043925	Eikenella_sp.	0.00043628	0.00025188	Microscillaceae_uncultured_Flexibacteraceae	0.00028981	0.00016732	Acetivibrio_uncultured_bacterium	0	0	Microscillaceae_uncultured_Candidatus	0.00013557	7.8273E-05
ASV_13977	0.00086973	0.00050214	Lactobacillus_vaginalis	0.0007081	0.00040882	ASV_1071	0.00042509	0.00024543	Rhodobacteraceae_uncultured_Amaricoccus	0.00028098	0.00016223	Acetoanaerobium_uncultured_bacterium	0	0	Howardella_sp.	0.00013369	7.7184E-05
Jatrophihabitans_uncultured_bacterium	0.0008	0.0005	ASV_363	0.0006	0.0003	Tepidimonas_sp.	0.0003	0.0002	Rothia_kristinae	0.0002	0.0001	Acetobacter_sp.	0	0	ASV_17763	0.0001	6.6158

	6973	0214		5531	7834		8034	1959		8098	6223					1459	E-05
Pedosphaeraceae_uncultured__metagenome	0.00086973	0.00050214	Opitutaceae_IMCC26134_sp.	0.00065531	0.00037834	ASV_14953	0.00037613	0.00021716	Niastella_sp.	0.00026225	0.00015141	Acetobacteraceae_sp.	0	0	ASV_897	0.00011459	6.6158E-05
Lactobacillus_rhamnosus	0.00081683	0.00047159	Rhodococcus_erythropolis	0.00065531	0.00037834	DEV008_uncultured_Verrucomicrobia	0.00037613	0.00021716	Rothia_aeria	0.00025359	0.00014641	Acetobacteraceae_uncultured__bacterium	0	0	Mitochondria_Eukaryota_sp.	0.00011459	6.6158E-05
ASV_18478	0.00078003	0.00045035	Saccharimonadalesuncultured_Chloroflexi	0.00065531	0.00037834	Eggerthellaceae_DNF00809_uncultured_actinobacterium	0.00037613	0.00021716	ASV_1071	0.00024352	0.0001406	Acetobacteraceae_uncultured__metagenome	0	0	Pedomicrobium_sp.	0.00011459	6.6158E-05
Caenarcaniphilales_uncultured_cyanobacterium	0.00078003	0.00045035	Actinomyces_israelii	0.00062943	0.0003634	Luteolibacter_arcticus	0.00037613	0.00021716	ASV_12623	0.00020605	0.00011897	Acetobacteraceae_uncultured__sp.	0	0	ASV_7533	8.7656E-05	5.0608E-05
Eubacterium_ruminantium_group_sp.	0.00078003	0.00045035	Ruminococcus_gnavus_group_sp.	0.00062943	0.0003634	Alistipes_sp.	0.00032912	0.00019002	Chloroplast_uncultured_bacillariophyte	0.00020605	0.00011897	Acetobacteraceae_uncultured_alpha_proteobacterium	0	0	ASV_2103	7.6393E-05	4.4105E-05
Exiguobacterium_undae	0.00078003	0.00045035	Oligoflexus_uncultured_delta	0.00060864	0.0003514	Gammaproteobacteria_CHAB-XI-27_uncultured_bacterium	0.00032912	0.00019002	Serpentinicella_sp.	0.00018113	0.00010458	Acetobacterium_sp.	0	0	ASV_4143	7.6393E-05	4.4105E-05
Novosphingobium_capsulatum	0.00078003	0.00045035	Chloroplast_Virgulinella_fragilis	0.00055075	0.00031797	Lachnospiraceae_A2_sp.	0.00032912	0.00019002	Ferribacterium_uncultured_bacterium	0.00016859	9.7335E-05	Acetothermiia__uncultured_bacterium	0	0	Desulfomonile_sp.	6.7787E-05	3.9137E-05
Arcobacteraceae_uncultured__sp.	0.0007731	0.00044635	Planctomycetes_uncultured_Planctomycetaceae	0.00055075	0.00031797	Thermodesulfovibrionia_uncultured__bacterium	0.00032912	0.00019002	Leifsonia_sp.	0.00016859	9.7335E-05	Acholeplasma__uncultured_bacterium	0	0	Angustibacter_uncultured_bacterium	6.2611E-05	3.6149E-05
Blastocatella_uncultured_Acidobacteria	0.0007731	0.00044635	Roseiflexaceae_uncultured_Chloroflexi	0.00055075	0.00031797	ASV_12280	0.00031858	0.00018393	Mitochondria_Eukaryota_sp.	0.00016859	9.7335E-05	Acholeplasma_axanthum	0	0	ASV_3215	6.2611E-05	3.6149E-05
Lactobacillus_amylovorus	0.0007731	0.00044635	Solobacterium_moorei	0.00055075	0.00031797	ASV_12284	0.00031858	0.00018393	Kocuria_marina	0.00014986	8.6521E-05	Acholeplasma_brassicae	0	0	ASV_5620	6.2611E-05	3.6149E-05
ASV_16023	0.00067646	0.00039055	Agitococcus_lubricus_group_sp.	0.00052425	0.00030267	Candidatus_Berkiella_Ectothiorhodospira_sp.	0.00031858	0.00018393	ASV_19805	0.00014491	8.3662E-05	Acholeplasma_laidlawii	0	0	Brevundimonas_diminuta	5.7295E-05	3.3079E-05
Bacteroidetes_vadinHA17_uncultured_bacterium	0.00067646	0.00039055	ASV_1592	0.00052425	0.00030267	Rothia_aeria	0.00031858	0.00018393	Candidatus_Kaiserbacteria_groundwater__metagenome	0.00014491	8.3662E-05	Acholeplasma_morum	0	0	Cellvibrio_uncultured_Cellvibrio	5.7295E-05	3.3079E-05
Propioniciclava_metagenome	0.00067646	0.00039055	Candidatus_Uhrbacteria_sp.	0.00052425	0.00030267	Cellvibrio_uncultured_Cellvibrio	0.00030392	9.4873E-05	Saccharimonadalesuncultured_candidate	0.00014491	8.3662E-05	Acholeplasma_sp.	0	0	Actinomycetaceae_uncultured__rumen	5.4229E-05	3.1309E-05
Peptostreptococcus_uncultured_organism	0.00062905	0.00036318	Macellibacteroides_sp.	0.00052425	0.00030267	Slackia_exigua	0.00029085	0.00016792	Providencia_sp.	0.00013113	7.5705E-05	Acholeplasmataceae_EMP-G18_uncultured_Mollicutes	0	0	Alphaproteobacteria_uncultured__metagenome	5.4229E-05	3.1309E-05
Actinobacteria_uncultured__bacterium	0.00062403	0.00036028	Oligoflexales_uncultured__soil	0.00052425	0.00030267	Anaerococcus_prevotii	0.00028962	0.00016721	ASV_17766	0.00011239	6.489E-05	Acholeplasmauncultured_Acholeplasmataceae	0	0	ASV_346	5.4229E-05	3.1309E-05
Candidatus_Uhrbacteria_uncultured_Parcubacteria	0.00062403	0.00036028	Gaiellales_uncultured_microorganism	0.00047207	0.00027255	ASV_13091	0.00028962	0.00016721	Howardella_sp.	0.00011239	6.489E-05	Achromatium_sp.	0	0	Clostridioides_sp.	5.4229E-05	3.1309E-05
Planctomycetes_uncultured__metagenome	0.00062403	0.00036028	Ilumatobacteraceae_uncultured_actinobacterium	0.00047207	0.00027255	Bdellovibrionaceae_OM27_cladeuncultured_delta	0.00028962	0.00016721	I-8_uncultured_bacterium	0.00011239	6.489E-05	Acidaminobacter__uncultured_bacterium	0	0	Luteolibacter_algae	5.4229E-05	3.1309E-05
Gemmatimonas_uncultured_soil	0.00057982	0.00033476	Acetitomaculum__uncultured_rumen	0.00045648	0.00026355	Tannerella_forsythia	0.00028962	0.00016721	Peptostreptococcales-Tissierellales_sp.	0.00011239	6.489E-05	Acidaminococcaceae_uncultured__bacterium	0	0	ASV_19760	5.0089E-05	2.8919E-05
Peptoniphilus_sp.	0.00057982	0.00033476	Candidatus_Amesbacteria_sp.	0.00045648	0.00026355	Candidatus_Woesebacteria_metagenome	0.0002821	0.00016287	Slackia_exigua	0.00011239	6.489E-05	Acidibacter__metagenome	0	0	Oligoflexus_uncultured_bacterium	5.0089E-05	2.8919E-05
Prevotella_timonensis	0.00057982	0.00033476	Candidatus_Paracaedibacter_uncultured_bacterium	0.00045648	0.00026355	Clostridia_vadinBB60_group_sp.	0.0002821	0.00016287	ASV_720	0.00010868	6.2746E-05	Acidibacter__uncultured_bacterium	0	0	Pseudoxanthomonas_sp.	5.0089E-05	2.8919E-05
Xanthomonadaceae_sp.	0.00057982	0.00033476	Streptobacillus_uncultured_bacterium	0.00045648	0.00026355	Erysipelotrichaceae_uncultured__bacterium	0.0002821	0.00016287	Bacilli_RF39_uncultured_rumen	0.00010868	6.2746E-05	Acidibacter_sp.	0	0	ASV_256	4.0672E-05	2.3482E-05
Acholeplasma_laidlawii	0.00048319	0.00027897	Blastocatellia_11-24_uncultured_Acidobacteria	0.00039339	0.00022712	Spirosomaceae_sp.	0.0002821	0.00016287	Saccharimonadales_TM7uncultured_Candidatus	0.00010868	6.2746E-05	Acidimicrobiia_IMCC26256_metagenome	0	0	ASV_2649	4.0672E-05	2.3482E-05
ASV_18451	0.00048319	0.00027897	Howardella_sp.	0.00039339	0.00022712	Lactobacillus_rhamnosus	0.00027966	0.00016146	Chloroplast_Epipyxis_sp.	9.3661E-05	5.4075E-05	Acidimicrobiia_IMCC26256_sp.	0	0	Lysobacter_sp.	4.0672E-05	2.3482E-05
Candidatus_Amesbacteria_Microgenomates_bacterium	0.00048319	0.00027897	Pedobacter_terrae	0.00039339	0.00022712	ASV_1592	0.00027871	0.00012247	Sphingorhabdus_sp.	5.6197E-05	3.2445E-05	Acidimicrobiia_IMCC26256_uncultured_bacterium	0	0	Pseudarcobacter_uncultured_Arcobacter	4.0672E-05	2.3482E-05
Chitinophaga_terrae	0.00048319	0.00027897	Puniceicoccaceae_sp.	0.00039339	0.00022712	Bacillus_uncultured_bacterium	0.00026066	0.00015049	Candidatus_Azambacteria_uncultured_bacterium	3.7464E-05	2.163E-05	Acidimicrobiia_IMCC26256_uncultured_soil	0	0	Saccharimonadalesuncultured_Candidatus	4.0672E-05	2.3482E-05
Leptotrichia_metagenome	0.00048319	0.00027897	Streptococcus_sobrinus	0.00039339	0.00022712	Flavobacterium_soli	0.00026066	0.00015049	Flavobacterium_lindanitolerans	3.7464E-05	2.163E-05	Acidimicrobiia_IMCC26256uncultured_Aciditerrimonas	0	0	SB-5_uncultured_bacterium	4.0672E-05	2.3482E-05
Oligoflexus_metagenome	0.00048319	0.00027897	ASV_709	0.00039319	0.00022701	Streptococcus_sobrinus	0.00025729	0.00014855	Parcubacteria_uncultured_bacterium	3.7464E-05	2.163E-05	Acidimicrobiia_IMCC26256uncultured_actinobacterium	0	0	ASV_292	3.8196E-05	2.2053E-05
Pajaroellobacter_uncultured_bacterium	0.00048319	0.00027897	Chitinophagaceae_uncultured__bacterium	0.00039319	0.00022701	Aquicella_unidentified	0.00023508	0.00013573	009E01-B-SD-P15__uncultured_bacterium	0	0	Acidimicrobiia_IMCC26256uncultured_Actinomycetales	0	0	Nocardiopsis_sp.	3.8196E-05	2.2053E-05
Parasediminibacterium_sp.	0.00048319	0.00027897	Nakamurella_sp.	0.00039319	0.00022701	ASV_18432	0.00023508	0.00013573	1013-28-CG33__uncultured_soil	0	0	Acidimicrobiia_sp.	0	0	ASV_4563	3.7567E-05	2.1689E-05
Rhizorhapis_uncultured_bacterium	0.00048319	0.00027897	Patescibacteria_WWE3_sp.	0.00039319	0.00022701	Candidatus_Limnoluna_uncultured_bacterium	0.00023508	0.00013573	Abditibacterium_sp.	0	0	Acidimicrobiia_uncultured__bacterium	0	0	Gemmata_bacterium_enrichment	3.7567E-05	2.1689E-05

Romboutsia_sp.	0.00048319	0.00027897	Eubacterium_hallii_group_sp.	0.00031471	0.0001817	Gammaproteobacteria_CHAB-XI-28_uncultured_bacterium	0.00023508	0.00013573	Absconditabacteriales_(SR1)__metagenome	0	0	Acidimicrobiia_uncultured__metagenome	0	0	Paludibaculum_sp.	3.7567E-05	2.1689E-05
Shewanella_sp.	0.00048319	0.00027897	Parcubacteriauncultured_deep-sea	0.00031471	0.0001817	Gammaproteobacteria_CHAB-XI-30_uncultured_bacterium	0.00023508	0.00013573	Absconditabacteriales_(SR1)__uncultured_bacterium	0	0	Acidimicrobiia_uncultured__sp.	0	0	Acetobacteraceae_uncultured__sp.	2.7115E-05	1.5655E-05
Shinella_sp.	0.00048319	0.00027897	Polaromonas_sp.	0.00031471	0.0001817	Chlamydiales_cvE6Chlamydiales_bacterium	0.0002317	0.00013377	Absconditabacteriales_(SR1)_candidate_division	0	0	Acidimicrobiia_uncultured_Acidimicrobiia	0	0	ASV_2390	2.7115E-05	1.5655E-05
Cryptobacterium_sp.	0.00047883	0.00027645	Slackia_exigua	0.00031471	0.0001817	Saccharimonadales_TM7_uncultured_bacterium	0.0002317	0.00013377	Absconditabacteriales_(SR1)_sp.	0	0	Acidimicrobiia_uncultured_actinobacterium	0	0	Bacteroides_pyogenes	2.7115E-05	1.5655E-05
Acetobacteraceae_sp.	0.00046802	0.00027021	ASV_8009	0.00030432	0.0001757	Catenulispora_rubra	0.00020273	0.0001705	Absconditabacteriales_(SR1)_SR1_bacterium	0	0	Acidimicrobiia_uncultured_Actinomycetales	0	0	Candidatus_Woesebacteria_sp.	2.7115E-05	1.5655E-05
Incertae_Sedis_sp.	0.00046802	0.00027021	ASV_8275	0.00030432	0.0001757	ASV_256	0.00018807	0.00010858	Absconditabacteriales_(SR1)_uncultured_candidate	0	0	Acidimicrobiia_uncultured_bacterium_YC-ZSS-LKJ199	0	0	Caulobacter_metagenome	2.7115E-05	1.5655E-05
Leeia_uncultured_bacterium	0.00046802	0.00027021	Candidatus_Yanofskybacteria	0.00030432	0.0001757	ASV_292	0.00018807	0.00010858	Absconditabacteriales_(SR1)_uncultured_Epsilonproteobacteria	0	0	Acidiphilium__uncultured_bacterium	0	0	Flavobacteriales_NS9_marine_group_metagenome	2.7115E-05	1.5655E-05
Pedosphaeraceae_uncultured__sp.	0.00046802	0.00027021	ASV_21722	0.00026212	0.00015134	ASV_366	0.00018807	0.00010858	Absconditabacteriales_(SR1)_uncultured_organism	0	0	Acidiphilium_sp.	0	0	Peredibactermicrobial_mat	2.7115E-05	1.5655E-05
ASV_1592	0.00044592	0.0001451	Bosea_sp.	0.00026212	0.00015134	Candidatus_Falkowbacteria__uncultured_soil	0.00018807	0.00010858	Absconditabacteriales_(SR1)_uncultured_prokaryote	0	0	Acidisoma_sp.	0	0	Saccharimonadales_sp.	2.7115E-05	1.5655E-05
ASV_19746	0.00038655	0.00022317	Clostridia_sp.	0.00026212	0.00015134	Dialister_uncultured_bacterium	0.00018807	0.00010858	ABY1_sp.	0	0	Acidithiobacillaceae_KCM-B-112_sp.	0	0	Sellimonas_sp.	2.7115E-05	1.5655E-05
ASV_19752	0.00038655	0.00022317	Acetobacteraceae_uncultured_alpha_proteobacterium	0.00023604	0.00013627	Planctomycetes_WD2101_soil_group_sp.	0.00018807	0.00010858	Acanthopleuribacter__uncultured_bacterium	0	0	Acidithiobacillus_ferrooxidans	0	0	Gammaproteobacteria_uncultured_marine	2.5045E-05	1.4459E-05
Brevundimonas_diminuta	0.00038655	0.00022317	Atopobium_sp.	0.00023604	0.00013627	Terrimicrobiaceae_FukuN18_freshwater_group_uncultured_bacterium	0.00018807	0.00010858	Acetitomaculum__uncultured_bacterium	0	0	Acidobacteria_bacterium	0	0	LD29_sp.	2.5045E-05	1.4459E-05
BSV26_denitrifying_bacterium	0.00038655	0.00022317	Candidatus_Yanofskybacteria_uncultured_organism	0.00023604	0.00013627	ASV_5084	0.00017377	0.00010033	Acetitomaculum__uncultured_rumen	0	0	Acidobacteriaceae_(Subgroup_1)_sp.	0	0	ASV_1161	1.3557E-05	7.8273E-06
Murdochiella_asaccharolytica	0.00038655	0.00022317	Cellvibrio_uncultured_bacterium	0.00023604	0.00013627	Sericytochromatiauncultured_cyanobacterium	0.00017377	0.00010033	Acetitomaculum_sp.	0	0	Acidobacteriae_Elev-16S-1166_sp.	0	0	ASV_3568	1.2522E-05	7.2297E-06
Oligoflexales_uncultured__soil	0.00038655	0.00022317	Deinococcus_uncultured_bacterium	0.00023604	0.00013627	ASV_2180	0.00015196	4.7437E-05	Acetivibrio_uncultured_bacterium	0	0	Acidobacteriae_Elev-16S-1166_uncultured_bacterium	0	0	Lawsonella_uncultured_bacterium	1.2522E-05	7.2297E-06
ASV_21727	0.00031201	0.00018014	Eikenella_sp.	0.00023604	0.00013627	Actinomycetaceae_F0332unidentified	0.00014481	8.3606E-05	Acetoanaerobium_uncultured_bacterium	0	0	Acidobacteriae_sp.	0	0	Leptospira_wolffii	1.2522E-05	7.2297E-06
Candidatus_Berkiella_unidentified_marine	0.00031201	0.00018014	Omnitrophia_sp.	0.00023604	0.00013627	Aeromicrobium_sp.	0.00014105	8.1435E-05	Acetobacter_sp.	0	0	Acidocella__uncultured_bacterium	0	0	009E01-B-SD-P15__uncultured_bacterium	0	0
Candidatus_Yanofskybacteria_uncultured_deep-sea	0.00028991	0.00016738	Trabulsiella_uncultured_bacterium	0.00023604	0.00013627	ASV_363	0.00014105	8.1435E-05	Acetobacteraceae_sp.	0	0	Acidothermus__uncultured_bacterium	0	0	1013-28-CG33__uncultured_soil	0	0
Gaiella_sp.	0.00028991	0.00016738	ASV_21979	0.00015736	9.085E-05	Flexithrix_dorotheae	0.00014105	8.1435E-05	Acetobacteraceae_uncultured__bacterium	0	0	Acidothermus_sp.	0	0	Abditibacterium_sp.	0	0
Pedosphaeraceae_uncultured__bacterium	0.00028991	0.00016738	Gammaproteobacteria_sp.	0.00015736	9.085E-05	Gallicola_uncultured_bacterium	0.00014105	8.1435E-05	Acetobacteraceae_uncultured__metagenome	0	0	Acidovorax_sp.	0	0	Absconditabacteriales_(SR1)__metagenome	0	0
Rhodobacteraceae_sp.	0.00028991	0.00016738	009E01-B-SD-P15__uncultured_bacterium	0	0	Kiritimatiellae_WCHB1-41_sp.	0.00014105	8.1435E-05	Acetobacteraceae_uncultured__sp.	0	0	Acinetobacter__metagenome	0	0	Absconditabacteriales_(SR1)__uncultured_bacterium	0	0
Eikenella_sp.	0.00023472	0.00013552	1013-28-CG33__uncultured_soil	0	0	Legionella_uncultured_bacterium	0.00014105	8.1435E-05	Acetobacterium_sp.	0	0	Acinetobacter_baumannii	0	0	Absconditabacteriales_(SR1)_candidate_division	0	0
Absconditabacteriales_(SR1)_uncultured_prokaryote	0.00019327	0.00011159	Abditibacterium_sp.	0	0	Patescibacteria_CPR2_uncultured_Clostridium	0.00014105	8.1435E-05	Acetothermiia__uncultured_bacterium	0	0	Acinetobacter_kyonggiensis	0	0	Absconditabacteriales_(SR1)_sp.	0	0
ASV_21709	0.00019327	0.00011159	Absconditabacteriales_(SR1)__metagenome	0	0	Prevotellaceae_uncultured_Prevotella_sp.	0.00014105	8.1435E-05	Acholeplasma__uncultured_bacterium	0	0	Acinetobacter_sp.	0	0	Absconditabacteriales_(SR1)_SR1_bacterium	0	0
Bacteriovoracaceae_uncultured__bacterium	0.00019327	0.00011159	Absconditabacteriales_(SR1)__uncultured_bacterium	0	0	Psychrobacter_sp.	0.00014105	8.1435E-05	Acholeplasma_axanthum	0	0	Acinetobacter_towneri	0	0	Absconditabacteriales_(SR1)_uncultured_candidate	0	0
Candidatus_Nomurabacteria_sp.	0.00019327	0.00011159	Absconditabacteriales_(SR1)_candidate_division	0	0	Sandaracinaceae_uncultured__uncultured_soil	0.00014105	8.1435E-05	Acholeplasma_brassicae	0	0	Acremonium_fuci	0	0	Absconditabacteriales_(SR1)_uncultured_Epsilonproteobacteria	0	0
Ruminiclostridium_metagenome	0.00019327	0.00011159	Absconditabacteriales_(SR1)_sp.	0	0	Spirosomaceae_uncultured__metagenome	0.00014105	8.1435E-05	Acholeplasma_laidlawii	0	0	Actinobacillus_sp.	0	0	Absconditabacteriales_(SR1)_uncultured_organism	0	0
Gemmatimonas_phototrophica	0.00015601	9.007E-05	Absconditabacteriales_(SR1)_SR1_bacterium	0	0	Patescibacteria_CPR2_uncultured_Firmicutes	0.00011585	6.6885E-05	Acholeplasma_morum	0	0	Actinobacteria_0319-7L14_uncultured_actinobacterium	0	0	Absconditabacteriales_(SR1)_uncultured_prokaryote	0	0
ASV_292	0.00013144	7.5889E-05	Absconditabacteriales_(SR1)_uncultured_candidate	0	0	Phenylobacterium_sp.	0.00011585	6.6885E-05	Acholeplasma_sp.	0	0	Actinobacteria_PeM15_bacterium_rJ7	0	0	ABY1_sp.	0	0
Acidimicrobiia_IMCC26256_uncultured_bacterium	9.3888E-05	5.4206E-05	Absconditabacteriales_(SR1)_uncultured_Epsilonproteobacteria	0	0	Peptococcus_niger	0.00011187	6.4586E-05	Acholeplasmataceae_EMP-G18_uncultured_Mollicutes	0	0	Actinobacteria_PeM15_metagenome	0	0	Acanthopleuribacter__uncultured_bacterium	0	0

ASV_256	8.4499E-05	4.8786E-05	Absconditabacteriales_(SR1)_uncultured_organism	0	0	Acetobacteraceae_uncultured_alpha_proteobacterium	0.00010068	5.8127E-05	Acholeplasmauncultured_Acholeplasmataceae	0	0	Actinobacteria_PeM15_sp.	0	0	Acetitomaculum__uncultured_bacterium	0	0
C86_sp.	8.4499E-05	4.8786E-05	Absconditabacteriales_(SR1)_uncultured_prokaryote	0	0	Proteiniphilum_sp.	0.00010068	5.8127E-05	Achromatium_sp.	0	0	Actinobacteria_PeM15_uncultured_soil	0	0	Acetitomaculum__uncultured_rumen	0	0
Slackia_exigua	8.4499E-05	4.8786E-05	ABY1_sp.	0	0	ASV_4527	9.4034E-05	5.429E-05	Acidaminobacter__uncultured_bacterium	0	0	Actinobacteria_PeM15uncultured_actinobacterium	0	0	Acetitomaculum_sp.	0	0
Acetobacteraceae_uncultured_alpha_proteobacterium	5.6333E-05	3.2524E-05	Acanthopleuribacter__uncultured_bacterium	0	0	Bacteroidales_RF16_group_sp.	9.4034E-05	5.429E-05	Acidaminococcaceae_uncultured__bacterium	0	0	Actinobacteria_sp.	0	0	Acetivibrio_uncultured_bacterium	0	0
Cellvibrio_uncultured_Cellvibrio	5.6333E-05	3.2524E-05	Acetitomaculum__uncultured_bacterium	0	0	C0119__uncultured_soil	9.4034E-05	5.429E-05	Acidibacter__metagenome	0	0	Actinobacteria_uncultured__bacterium	0	0	Acetoanaerobium_uncultured_bacterium	0	0
Butyrivibrio_Eubacterium_sp.	4.6944E-05	2.7103E-05	Acetitomaculum_sp.	0	0	Collinsella_sp.	9.4034E-05	5.429E-05	Acidibacter__uncultured_bacterium	0	0	Actinobacteria_uncultured__metagenome	0	0	Acetobacter_sp.	0	0
Collimonas_sp.	4.6944E-05	2.7103E-05	Acetivibrio_uncultured_bacterium	0	0	Gracilibacteria_metagenome	9.4034E-05	5.429E-05	Acidibacter_sp.	0	0	Actinobacteria_uncultured__sp.	0	0	Acetobacteraceae_sp.	0	0
Solirubrobacterales_67-14__uncultured_bacterium	4.6944E-05	2.7103E-05	Acetoanaerobium_uncultured_bacterium	0	0	Phycisphaeraceae_SM1A02_metagenome	9.4034E-05	5.429E-05	Acidimicrobiia_IMCC26256_metagenome	0	0	Actinobacteria_uncultured_Actinomycetales_bacterium	0	0	Acetobacteraceae_uncultured__bacterium	0	0
Flavobacterium_uncultured_Flavobacteriaceae	3.7555E-05	2.1683E-05	Acetobacter_sp.	0	0	Sphingobacteriales_env.OPS_17_uncultured_soil	9.4034E-05	5.429E-05	Acidimicrobiia_IMCC26256_sp.	0	0	Actinobacteriota_MB-A2-108_metagenome	0	0	Acetobacteraceae_uncultured__metagenome	0	0
Kiritimatiellae_WCHB1-41uncultured_Verrucomicrobia	3.7555E-05	2.1683E-05	Acetobacteraceae_sp.	0	0	Turneriella_sp.	9.4034E-05	5.429E-05	Acidimicrobiia_IMCC26256_uncultured_bacterium	0	0	Actinobacteriota_MB-A2-108_sp.	0	0	Acetobacterium_sp.	0	0
Peptoclostridium_uncultured_organism	3.7555E-05	2.1683E-05	Acetobacteraceae_uncultured__bacterium	0	0	WPS-2hydrothermal_vent	9.4034E-05	5.429E-05	Acidimicrobiia_IMCC26256_uncultured_soil	0	0	Actinobacteriota_MB-A2-108_uncultured_bacterium	0	0	Acetothermiia__uncultured_bacterium	0	0
Anaerovoracaceae_Family_XIII_UCG-004Eubacterium_sp.	2.8166E-05	1.6262E-05	Acetobacteraceae_uncultured__metagenome	0	0	Methanobacterium_flexile	8.6886E-05	5.0164E-05	Acidimicrobiia_IMCC26256uncultured_Aciditerrimonas	0	0	Actinobacteriota_MB-A2-108uncultured_actinobacterium	0	0	Acholeplasma__uncultured_bacterium	0	0
Gracilibacteria_JGI_0000069-P22Gracilibacteria_bacterium	2.8166E-05	1.6262E-05	Acetobacteraceae_uncultured__sp.	0	0	Beijerinckiaceae_uncultured__uncultured_soil	7.8306E-05	4.521E-05	Acidimicrobiia_IMCC26256uncultured_actinobacterium	0	0	Actinomadura_sp.	0	0	Acholeplasma_axanthum	0	0
Leptotrichia_wadei	2.8166E-05	1.6262E-05	Acetobacterium_sp.	0	0	Niabella_uncultured_bacterium	7.8306E-05	4.521E-05	Acidimicrobiia_IMCC26256uncultured_Actinomycetales	0	0	Actinomyces__uncultured_bacterium	0	0	Acholeplasma_brassicae	0	0
Alphaproteobacteria_uncultured_Rhodospirillaceae_bacterium	1.8778E-05	1.0841E-05	Acetothermiia__uncultured_bacterium	0	0	Geodermatophilus_sp.	6.7119E-05	3.8751E-05	Acidimicrobiia_sp.	0	0	Actinomyces_dentalis	0	0	Acholeplasma_laidlawii	0	0
Anaerovoracaceae_Family_XIII_UCG-005Eubacterium_sp.	1.8778E-05	1.0841E-05	Acholeplasma__uncultured_bacterium	0	0	Candidatus_Woesebacteria_uncultured_Microgenomates	5.7924E-05	3.3442E-05	Acidimicrobiia_uncultured__bacterium	0	0	Actinomyces_graevenitzii	0	0	Acholeplasma_morum	0	0
Chitinophagaceae_sp.	1.8778E-05	1.0841E-05	Acholeplasma_axanthum	0	0	Acetoanaerobium_uncultured_bacterium	5.5933E-05	3.2293E-05	Acidimicrobiia_uncultured__metagenome	0	0	Actinomyces_israelii	0	0	Acholeplasma_sp.	0	0
Oligoflexia_0319-6G20_marine__metagenome	1.8778E-05	1.0841E-05	Acholeplasma_brassicae	0	0	Acidisoma_sp.	5.5933E-05	3.2293E-05	Acidimicrobiia_uncultured__sp.	0	0	Actinomyces_sp.	0	0	Acholeplasmataceae_EMP-G18_uncultured_Mollicutes	0	0
Tyzzerella_sp.	1.8778E-05	1.0841E-05	Acholeplasma_laidlawii	0	0	Bifidobacteriaceae_sp.	5.5933E-05	3.2293E-05	Acidimicrobiia_uncultured_Acidimicrobiia	0	0	Actinomyces_unidentified	0	0	Acholeplasmauncultured_Acholeplasmataceae	0	0
009E01-B-SD-P15__uncultured_bacterium	0	0	Acholeplasma_morum	0	0	Methylomonas_uncultured_bacterium	5.5933E-05	3.2293E-05	Acidimicrobiia_uncultured_actinobacterium	0	0	Actinomycetaceae_F0332_uncultured_bacterium	0	0	Achromatium_sp.	0	0
1013-28-CG33__uncultured_soil	0	0	Acholeplasma_sp.	0	0	ASV_6240	4.7017E-05	2.7145E-05	Acidimicrobiia_uncultured_Actinomycetales	0	0	Actinomycetaceae_F0332unidentified	0	0	Acidaminobacter__uncultured_bacterium	0	0
Abditibacterium_sp.	0	0	Acholeplasmataceae_EMP-G18_uncultured_Mollicutes	0	0	ASV_20280	4.4746E-05	2.5834E-05	Acidimicrobiia_uncultured_bacterium_YC-ZSS-LKJ199	0	0	Actinomycetaceae_uncultured__bacterium	0	0	Acidaminococcaceae_uncultured__bacterium	0	0
Absconditabacteriales_(SR1)__metagenome	0	0	Acholeplasmauncultured_Acholeplasmataceae	0	0	ASV_20283	4.4746E-05	2.5834E-05	Acidiphilium__uncultured_bacterium	0	0	Actinomycetaceae_uncultured__metagenome	0	0	Acidibacter__metagenome	0	0
Absconditabacteriales_(SR1)__uncultured_bacterium	0	0	Achromatium_sp.	0	0	Campylobacter_concisus	4.4746E-05	2.5834E-05	Acidiphilium_sp.	0	0	Actinomycetaceae_uncultured__rumen	0	0	Acidibacter__uncultured_bacterium	0	0
Absconditabacteriales_(SR1)_candidate_division	0	0	Acidaminobacter__uncultured_bacterium	0	0	Cellulosimicrobium_sp.	4.4746E-05	2.5834E-05	Acidisoma_sp.	0	0	Actinomycetaceae_uncultured__sp.	0	0	Acidibacter_sp.	0	0
Absconditabacteriales_(SR1)_sp.	0	0	Acidaminococcaceae_uncultured__bacterium	0	0	Chloroplast_Juniperus_virginiana	4.4746E-05	2.5834E-05	Acidithiobacillaceae_KCM-B-112_sp.	0	0	Actinomycetospora_sp.	0	0	Acidimicrobiia_IMCC26256_metagenome	0	0
Absconditabacteriales_(SR1)_SR1_bacterium	0	0	Acidibacter__metagenome	0	0	Corynebacterium_sp.	4.4746E-05	2.5834E-05	Acidithiobacillus_ferrooxidans	0	0	Actinoplanes_garbadinensis	0	0	Acidimicrobiia_IMCC26256_sp.	0	0
Absconditabacteriales_(SR1)_uncultured_candidate	0	0	Acidibacter__uncultured_bacterium	0	0	Hydrogenophilus_sp.	4.4746E-05	2.5834E-05	Acidobacteria_bacterium	0	0	Actinoplanes_sp.	0	0	Acidimicrobiia_IMCC26256_uncultured_bacterium	0	0
Absconditabacteriales_(SR1)_uncultured_Epsilonproteobacteria	0	0	Acidibacter_sp.	0	0	Oligoflexia_0319-6G20__uncultured_soil	4.4746E-05	2.5834E-05	Acidobacteriaceae_(Subgroup_1)_sp.	0	0	Actinotalea_sp.	0	0	Acidimicrobiia_IMCC26256_uncultured_soil	0	0
Absconditabacteriales_(SR1)_uncultured_organism	0	0	Acidimicrobiia_IMCC26256_metagenome	0	0	Spirosoma_uncultured_bacterium	4.4746E-05	2.5834E-05	Acidobacteriae_Elev-16S-1166_sp.	0	0	Actinotignum_sp.	0	0	Acidimicrobiia_IMCC26256uncultured_Aciditerrimonas	0	0

ABY1_sp.	0	0	Acidimicrobiia_IMCC26256_sp.	0	0	Candidatus_BabelaCandidatus_Babela	3.356E-05	1.9376E-05	Acidobacteriae_Elev-16S-1166_uncultured_bacterium	0	0	Adhaeribacter__metagenome	0	0	Acidimicrobiia_IMCC26256uncultured_actinobacterium	0	0
Acanthopleuribacter__uncultured_bacterium	0	0	Acidimicrobiia_IMCC26256_uncultured_bacterium	0	0	Candidatus_Falkowbacteria_uncultured_anaerobic	3.356E-05	1.9376E-05	Acidobacteriae_sp.	0	0	Adhaeribacter__uncultured_soil	0	0	Acidimicrobiia_IMCC26256uncultured_Actinomycetales	0	0
Acetitomaculum__uncultured_bacterium	0	0	Acidimicrobiia_IMCC26256_uncultured_soil	0	0	Hyphomicrobium_vulgare	3.356E-05	1.9376E-05	Acidocella__uncultured_bacterium	0	0	Adhaeribacter_sp.	0	0	Acidimicrobiia_sp.	0	0
Acetitomaculum__uncultured_rumen	0	0	Acidimicrobiia_IMCC26256uncultured_Aciditerrimonas	0	0	Legionella_beliardensis	3.356E-05	1.9376E-05	Acidothermus__uncultured_bacterium	0	0	Advenella_sp.	0	0	Acidimicrobiia_uncultured__bacterium	0	0
Acetitomaculum_sp.	0	0	Acidimicrobiia_IMCC26256uncultured_actinobacterium	0	0	Solirubrobacterales_67-14_uncultured_Rubrobacteria	3.356E-05	1.9376E-05	Acidothermus_sp.	0	0	Aenigmarchaeales_sp.	0	0	Acidimicrobiia_uncultured__metagenome	0	0
Acetivibrio_uncultured_bacterium	0	0	Acidimicrobiia_IMCC26256uncultured_Actinomycetales	0	0	Turicella_uncultured_bacterium	3.356E-05	1.9376E-05	Acidovorax_sp.	0	0	Aenigmarcheota_sp.	0	0	Acidimicrobiia_uncultured__sp.	0	0
Acetoanaerobium_uncultured_bacterium	0	0	Acidimicrobiia_sp.	0	0	Oligoflexales_uncultured__soil	2.8962E-05	1.6721E-05	Acinetobacter__metagenome	0	0	Aequorivita_sp.	0	0	Acidimicrobiia_uncultured_Acidimicrobiia	0	0
Acetobacter_sp.	0	0	Acidimicrobiia_uncultured__bacterium	0	0	Aerococcus_sp.	2.2373E-05	1.2917E-05	Acinetobacter_baumannii	0	0	Aerococcaceae_sp.	0	0	Acidimicrobiia_uncultured_actinobacterium	0	0
Acetobacteraceae_uncultured__bacterium	0	0	Acidimicrobiia_uncultured__metagenome	0	0	Parcubacteria_metagenome	2.2373E-05	1.2917E-05	Acinetobacter_kyonggiensis	0	0	Aerococcaceae_uncultured__bacterium	0	0	Acidimicrobiia_uncultured_Actinomycetales	0	0
